# Synthesis and Ring Expansion of Triflylated Cyclobutenyl MIDA Boronates

**DOI:** 10.1021/acs.orglett.6c02345

**Published:** 2026-06-25

**Authors:** Sara Gallardo, Mireia Toledano-Pinedo, Pablo Pastor, Cristina Aragoncillo, José M. Alonso, Hikaru Yanai, Pedro Almendros

**Affiliations:** ‡ Instituto de Química Orgánica General, 201430 IQOG, CSIC, Juan de la Cierva 3, 28006 Madrid, Spain; § Grupo de Lactamas y Heterociclos Bioactivos, Unidad Asociada al CSIC por el IQOG, Departamento de Química Orgánica, Facultad de Química, 16734 Universidad Complutense de Madrid, 28040 Madrid, Spain; ∥ School of Pharmacy, 13115 Tokyo University of Pharmacy and Life Sciences, 1432-1 Horinouchi, Hachioji, Tokyo 192-0392, Japan

## Abstract

A synthetic method for regioselectively building borylated bis­(triflyl)­cyclobutenes from BMIDA alkynes and *in situ* formed 1,1-bis­(triflyl)­ethylene has been developed. In addition, the conversion of the so-prepared borylated cyclobutenes into BMIDA-substituted naphthyl triflones has been accomplished by simple thermal treatment without using any catalyst. A sequence capable of directly affording functionalized naphthalenes from alkynes in a one-pot manner was also possible. In this way, a divergent approach to offering otherwise difficult-to-prepare cyclobutenyl boronates and ((triflyl)­naphthalen-1-yl)­boranes from the same precursor by slight modification of the reaction conditions has been discovered.

## Graphical abstract

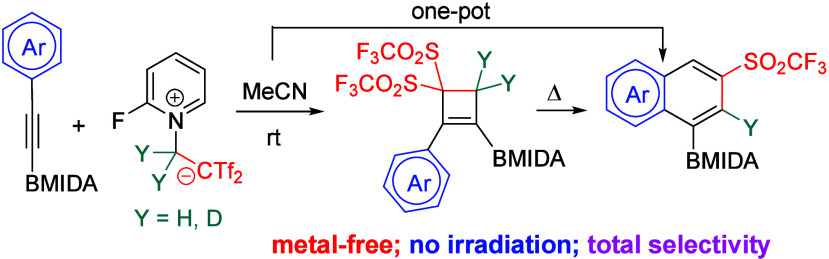

Alkenyl boronates are a particular type of organoboron compound that has been extensively used in organic synthesis, materials science, and medicinal chemistry (Scheme 1, top).^1^ On the other hand, cyclobutene derivatives are recognized as versatile building blocks in organic synthesis and also are pharmaceutically relevant compounds (Scheme 1, top).^2^ The synthesis of cyclobutanyl boronates is widely reported in the literature,^3^ but methods for the preparation of more strained cyclobutenyl boronates are rare. These previous protocols include the preparation of a sole example of the cyclobutene-derived boronate core from the reaction of an alkynyl pinacol ester with either a Fisher carbene complex (Scheme 1a)^4^ or ethylene catalyzed by a sophisticated cobalt salt (Scheme 1b).^5^ A recent strategy to prepare fused bicyclic cyclobutene-derived boronates started from alkyl-ended alkynyl Bpin (pin = pinacolato) compounds and maleimides and used photochemical conditions (Scheme 1c).^6^ Besides, borylated triflyl-decorated molecules are very appealing because they combine the relevant chemical and biological properties of fluorinated compounds with the organoboron scaffold.^7^ Consequently, the development of efficient synthetic strategies for unexplored hybrid cyclobutenyl triflylated boronates may allow access to new chemical space, but it remains a challenge for chemical methodology. Previously, Yanai described the convenient preparation of zwitterion **1** as a bis­[(trifluoromethyl)­sulfonyl]-ethylating reagent,^8^ and in the present work, we surveyed its reaction with BMIDA alkynes (MIDA = *N*-methyliminodiacetyl) to prepare (triflyl)­cyclobutene-derived boronates and a further ring expansion (Scheme 1d).

**Scheme 1:**
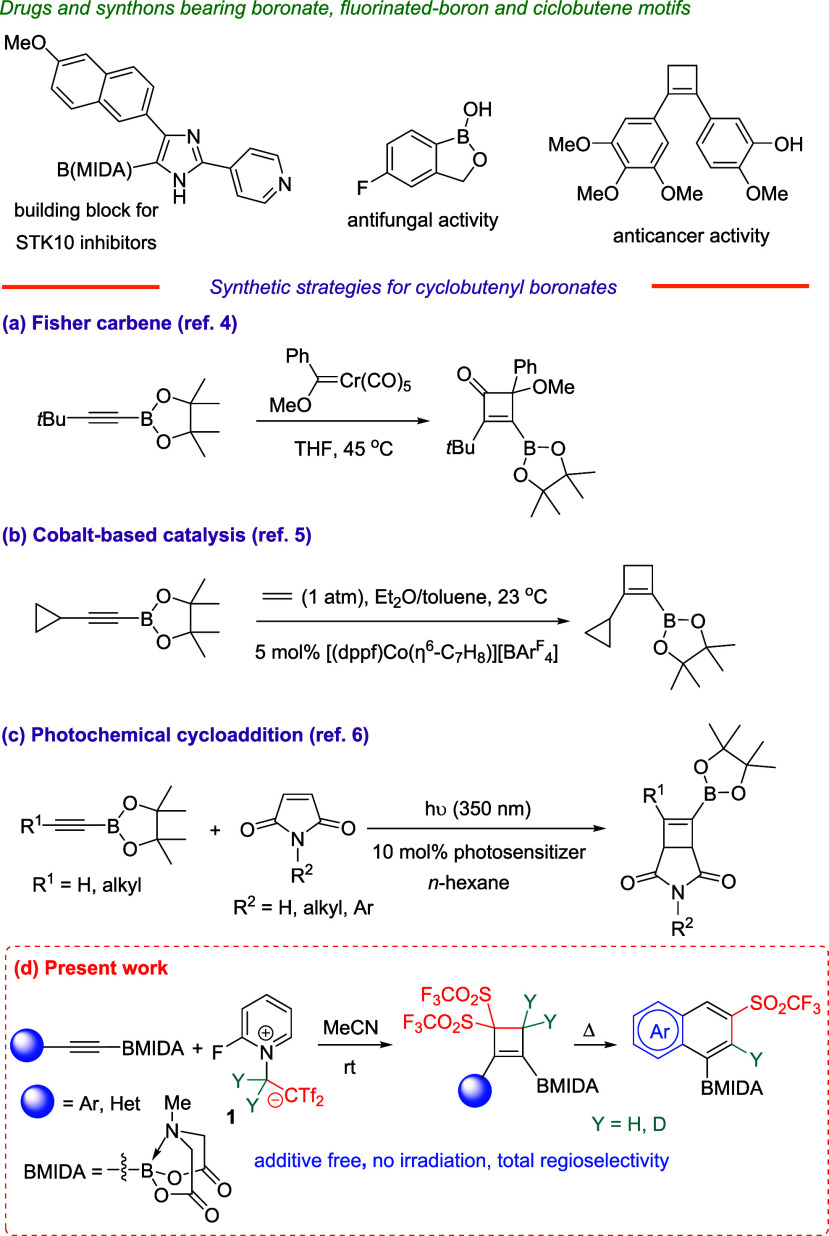
Prior Art and Current Strategy

It should be noted that the formation of borylated cyclobutenes from alkynyl boron compounds and Yanai’s reagent **1** is challenging because *in situ* formed 1,1-bis­(triflyl)­ethylene quickly reacts with compounds bearing heteroatoms, such as N, P, and As, giving rise to zwitterionic species.^9^ An initial test reaction between zwitterion **1** and **2a**-Bpin (1-ethynyl-4-methoxybenzene Bpin) resulted in a complex mixture, probably containing protodeboronation and C–B activation products (Scheme 2a). In contrast to commonly employed alkynyl Bpin boronates, which needed purification through distillation and storage under an inert atmosphere,^10^ BMIDA alkynes are moderately stable due to their lower Lewis acidity, which granted recrystallization and chromatography purification.^10^ Taking into account that the MIDA-protective group in BMIDA boronates functions as a useful stabilizer of easily altered C–B bonds, we decided to inspect the reaction of zwitterion **1** with 1-ethynyl-4-methoxybenzene BMIDA **2a**. Indeed, the initial problem was overcome through the use of BMIDA alkyne **2a** because the controlled preparation of BMIDA cyclobutene **3a** was smoothly accomplished (Scheme 2) while disfavoring the formation of side products with zwitterion **1**. The investigation of the effect of solvents revealed that acetonitrile was superior to all other solvents evaluated (chlorinated solvents, aromatic hydrocarbons, ethers, DMF, and DMSO) due to the good solubility of both BMIDA alkyne and zwitterion **1** at room temperature. Despite previous reports on the use of reagent **1** outlining that product identity was strongly solvent-dependent, resulting not in cyclobutene formation but rather intermolecular cyclization,^8e^ we only observed the genesis of BMIDA cyclobutenes. As previously reported by Yanai,^8a^ the reactivity of zwitterions related to **1** depends on the p*K*_aH_ values of the substituted pyridine moiety, and 2-fluoropyridine-based betaine **1** displays the higher reactivity.

**Scheme 2:**
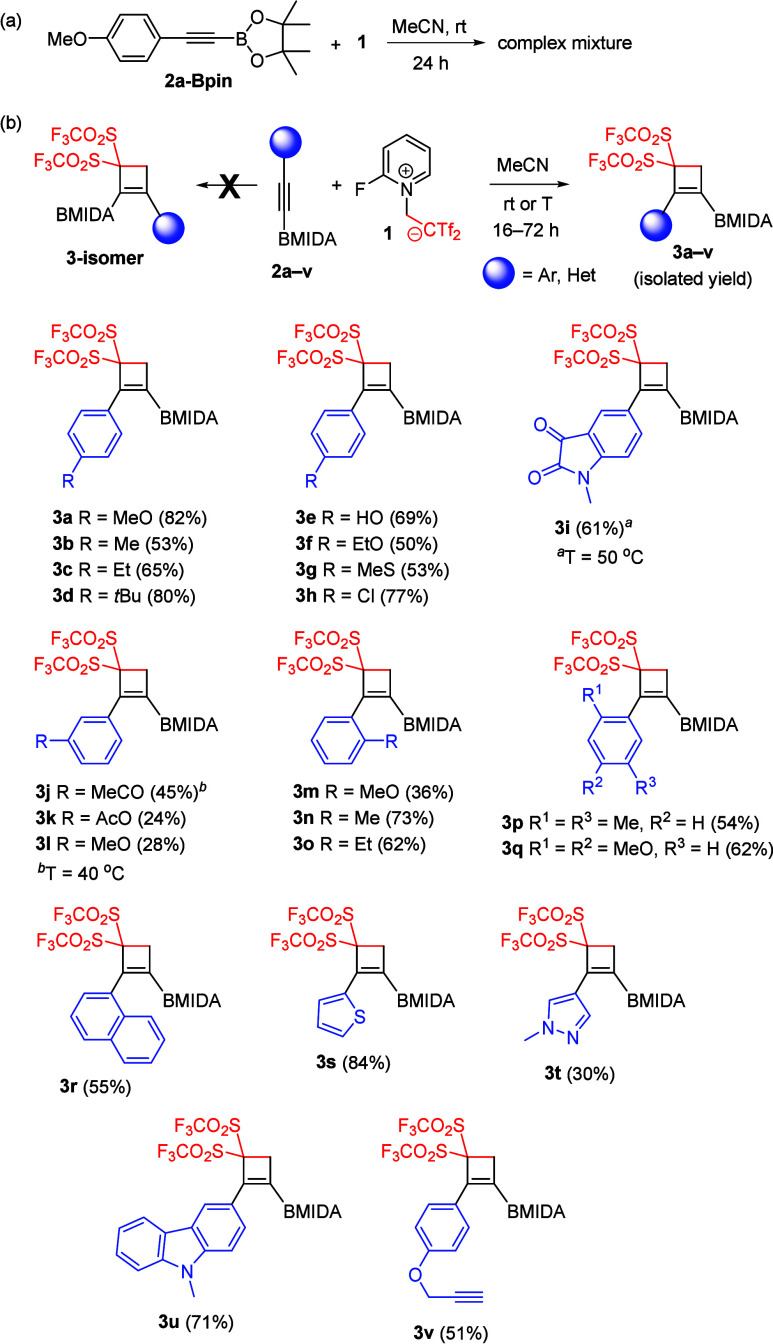
Controlled Synthesis of BMIDA Cyclobutenes **3**

With the optimized reaction conditions in hand, the scope of the fluorinated cyclobutenyl boronate formation was explored (Scheme 2b). It was encountered that the electronic characteristics of the substituents attached to the alkyne moiety exert a considerable influence on the reaction outcome. Electron-donating groups (Me, *t*Bu, HO, MeO, MeS, and OEt) and mild electron-withdrawing groups, such as chlorine, were well-accommodated, providing the required borylated cyclobutenes **3a**–**3h** in good yields. However, little conversion was detected at rt when the transformation was assayed in BMIDA alkynes **2i** and **2j** having electron-withdrawing substituents, suggesting an electronic influence for the success of the intermolecular cyclization reaction. Fortunately, total conversion was achieved when *T* was slightly increased to 50 °C (for the isatin nucleus, **2i**) and to 40 °C (for the acetyl substituent, **2j**). Different substitution patterns (*ortho*, *meta*, and *para*) at the substituted phenyl rings were smoothly engaged in the annulation reaction.

However, it appeared that the steric hindrance influences the reaction efficiency because *ortho*-substituted adduct **3m** was obtained in a diminished yield in comparison to its *para*-substituted counterpart **3a**.^11^ A 1-naphthyl ring was also suitable, furnishing the appropriate cyclobutenyl MIDA boronate **3r**. The cyclization reaction was not limited to BMIDA alkynes substituted with a phenyl ring. Also, heterocyclic nuclei, such as thiophene (**3s**), pyrazole (**3t**), and carbazole (**3u**), were tolerated. Noteworthy, the process exhibited an exquisite regioselectivity toward the formation of cyclobutene-derived boronates **3**, avoiding competitive regioisomeric reactions. Besides, total chemoselectivity toward the alkynyl-BMIDA moiety was procured starting from alkyne **2v**. Indeed, the coupling of **2v** with zwitterion **1** delivered cyclobutenyl boronate **3v**, in which the terminal alkyne remained untouched. Additionally, cyclobutenyl boron compounds **3** are air-stable compounds that can be easily handled. Alkyl-substituted alkynes related to **2** are not good partners, as was made clear by the failure of the reaction between (4-phenylbut-1-yn-1-yl)­BMIDA and betaine **1**.

Because deuterated derivatives have displayed notorious applications in drug discovery and mechanistic studies,^12^ some BMIDA alkynes **2** were selected to react with zwitterion **1**-*d*_2_ ^13^ to prove the viability of our protocol for the preparation of deuterated cyclobutenyl MIDA boronates. In the event, deuterated strained cycles **3**-*d*_2_ were successfully forged (Scheme 3). In the four cases, the reaction exclusively leads to the formation of fluorinated [2π + 2π]-cycloadduct **3**-*d*_2_ rather than the regioisomeric **3**-isomer-*d*_2_ product.

**Scheme 3:**
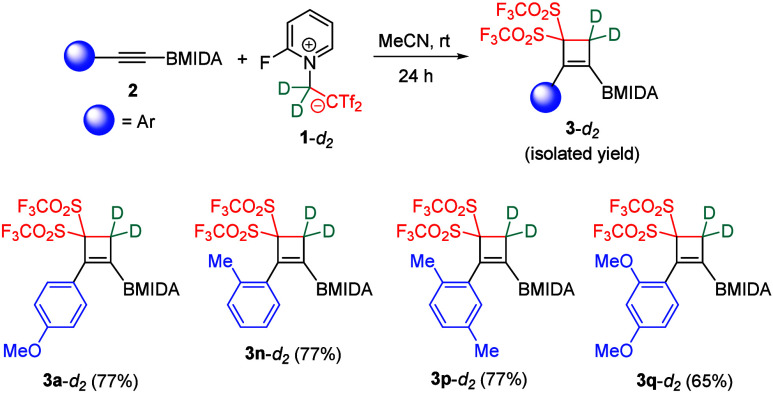
Synthesis of Labeled BMIDA Cyclobutenes 3-*d*_2_

Yanai previously reported the dynamic behavior of betaine **1**,^8b^ which quickly (sub-milliseconds) dissociated in solution to provide Tf_2_C=CH_2_ and 2-fluoropyridine. Besides, the Tf_2_C*H*R moiety exhibits a strong carbon acid character, but Tf_2_C-derived carbanions are weak nucleophiles. With both facts taken into consideration, an initial nucleophilic attack from the anionic carbon in zwitterion **1** onto alkynes **2** is highly unlikely. A feasible reaction pathway is shown in Scheme 4. Initially, highly electrophilic alkene 1,1-bis­[(trifluoromethyl)­sulfonyl]­ethene (Tf_2_C=CH_2_) is liberated *in situ* from zwitterionic reagent **1**. Ensuring nucleophilic attack of BMIDA alkynes **2** toward Tf_2_C=CH_2_ should result in the formation of zwitterionic intermediate **I**. Subsequent cyclization should forge triflylated cyclobutenyl MIDA boronate **3**. The regiochemical outcome should be imparted by the higher stability of intermediate **I** in comparison to intermediate **II**. It may be presumed that species **I** is capable of greater stabilization of the carbocation because the positive charge is close to the electron-rich aromatic ring. Because of the less efficient support of the BMIDA moiety in putative intermediate **II**, functionalized cyclobutenes **3** are solely constructed instead of **3**-isomer regioisomers.

**Scheme 4:**
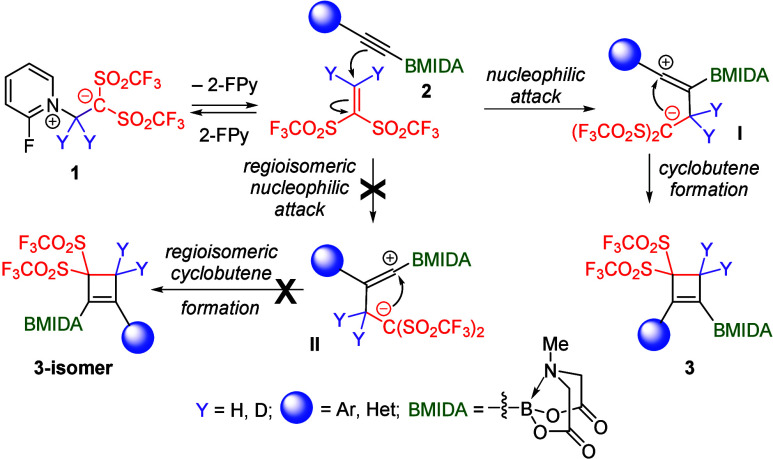
Rationalization for the Controlled Formation of Triflylated Cyclobutenyl MIDA Boronates **3**

A scale-up reaction of BMIDA alkyne **2a** and betaine **1** was performed under the optimized conditions, providing BMIDA cyclobutene **3a** with comparable results (Scheme 5a). To manifest the further usefulness of our procedure, post-synthetic transformations were conducted. Preliminary results of the BMIDA cyclobutene coupling were not very rewarding. The reaction of cyclobutene **3a** with 4-iodoanisole, both catalyzed by Pd­(OAc)_2_ and Pd­(PPh_3_)_4_, in the presence of SPhos and K_2_CO_3_, respectively, resulted in complex reaction mixtures. Similarly, the treatment of cyclobutene **3a** with oxone occurred with decomposition. The thermal treatment of borylated bis­(triflyl)­cyclobutene **3a** proceeded smoothly. Surprisingly, the expected adduct arising from a [2,3]-sigmatropic rearrangement of the allylic trifluoromethanesulfonate moiety was not attained.^14^ To our delight, the obtained product was ring-expanded BMIDA-substituted naphthyl triflone **4a** (Scheme 5b). With the both the novelty of the transformation as well as the interest of the obtained products taken into account,^15^ the process was explored to a greater extent (Scheme 5b). It is noteworthy that the electronic nature of the arene ring at the cyclobutene nucleus did not exert any influence on the efficiency of the ring-expansion reaction. Indeed, both electron-donating and electron-withdrawing substituents were tolerated, which should discard an electrophilic aromatic substitution-type reaction. We also surveyed the reactivity of cyclobutenyl boronates **3**, having substituents at different positions on the benzene ring. *meta*-Substituted BMIDA cyclobutenes, such as **3k** and **3l**, were profitable for the ring enlargement reaction, yielding functionalized naphthalenes **4k** and **4l**. However, difficulties were encountered in *ortho*-substituted molecules, such as **3m** and **3n**, probably because of steric hindrance, leading to unproductive decomposition of the starting material. We were pleased to observe that hetaryl derivative **3s** was well-accommodated for the ring enlargement strategy, providing target benzannulation product **4s** in a fair yield.^16^ In order to broaden the scope and probe the versatility of our protocol, we introduced labeled derivative **3a**-*d*_2_ as a precursor. As expected, required naphthyl triflone **4a**-*d* was obtained (Scheme 5b), providing convenient access to otherwise difficult-to-prepare deuterated functionalized arenes. This sequential transformation converts alkynes into borylated organofluorine naphthalenes, but it was also possible to have a sequence capable of directly affording functionalized naphthalenes **4** from alkynes in a one-pot manner (Scheme 5b). The bicyclic structures of naphthalenes **4** were established by NMR two-dimensional techniques, such as ^1^H–^1^H COSY, ^1^H–^1^H TOCSY, ^1^H–^13^C HMBC, and ^1^H–^13^C HSQC in compound **4a**, ^1^H–^13^C HMBC and ^1^H–^13^C HSQC in compound **4a**-*d*, ^1^H–^1^H COSY in compound **4e**, ^1^H–^13^C HMBC and ^1^H–^13^C HSQC in compound **4h**, ^1^H–^13^C HMBC, ^1^H–^13^C HSQC, and NOESY in compound **4l**, and ^1^H–^13^C HMBC and ^1^H–^13^C HSQC in compound **4s** (see the Supporting Information).

**Scheme 5:**
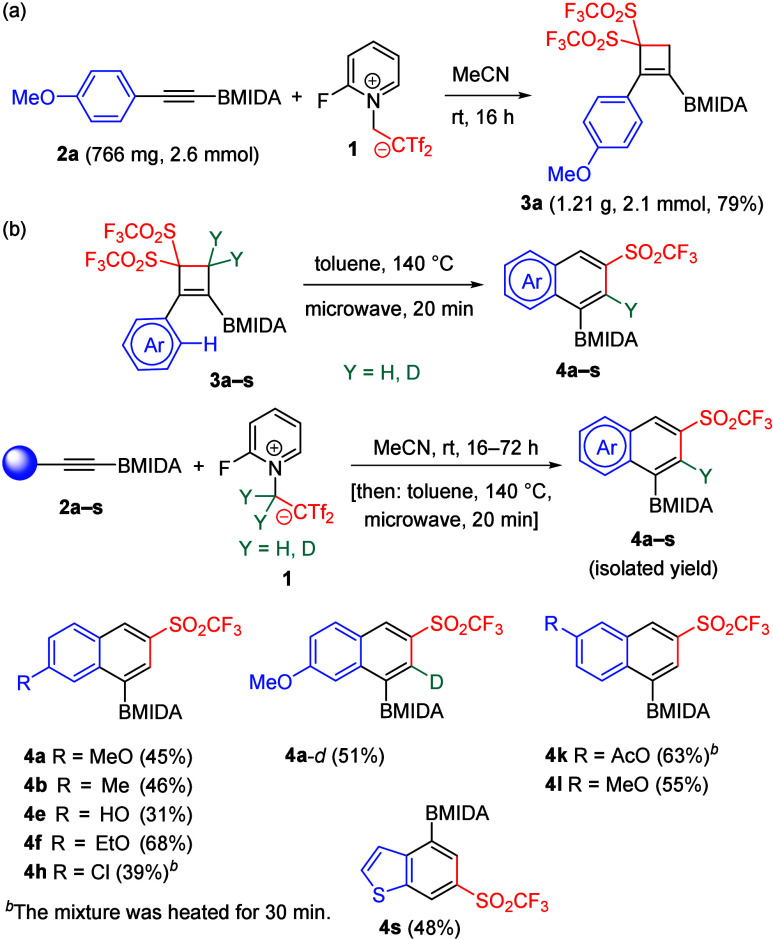
Scalability and Synthesis of BMIDA-Functionalized Naphthyl Triflones **4**

To procure a mechanistic understanding, we ran control experiments in the presence of the radical scavengers 2,2,6,6-tetramethylpiperidin-4-oxy (TEMPO) and 2,6-di-*tert*-butyl hydroxytoluene (BHT). When either TEMPO or BHT was incorporated into the standard reactions of **3a** or **3b**, the generation of **4a** or **4b** was frustrated (Scheme 6a), pointing to a radical path. Besides, the free radical capture experiment using **3b** and TEMPO resulted in the detection (by LC–MS analysis of the crude reaction mixture) of an intermediate trapped by two molecules of TEMPO, showing a mass-to-charge ratio (*m*/*z*) of 898.30 [M + Na]^+^ (see the Supporting Information). No significant kinetic isotope effect was observed (KIE = 1.04) when BMIDA cyclobutene **3a** and deuterated analogue **3a**-*d*_2_ were independently subjected to the standard reaction conditions in parallel experiments (Scheme 6b). This result suggests that cleavage of the C­(sp^3^)–H bond is unlikely to be involved in the rate-determining step. A conceivable mechanism for the generation of fused arenes **4** from cyclobutenyl boronates **3** is depicted in Scheme 6c. Initially, [2,3]-sigmatropic rearrangement to generate trifluoromethyl sulfinate cyclobutenyl ester intermediate **A** should occur.^17^ If intermediate **A** suffers homolytic cleavage of one of the single bonds of the cyclobutene ring, diradical intermediate **B** may be formed. The driving force for the formation of diradical **B** must be the great stabilizing effect imparted by the BMIDA group on α-carbon-centered radicals, as recently reported.^18^ The vinyl radical in species **B** should launch a translocation^19^ through 1,3-hydrogen abstraction to form aryl radical **C**, which should experience delocalization over the ring. Subsequent cyclization to generate neutral bicycle **D** should be followed by release of trifluoromethanesulfinic acid with accompanying formation of final products **4**.

**Scheme 6:**
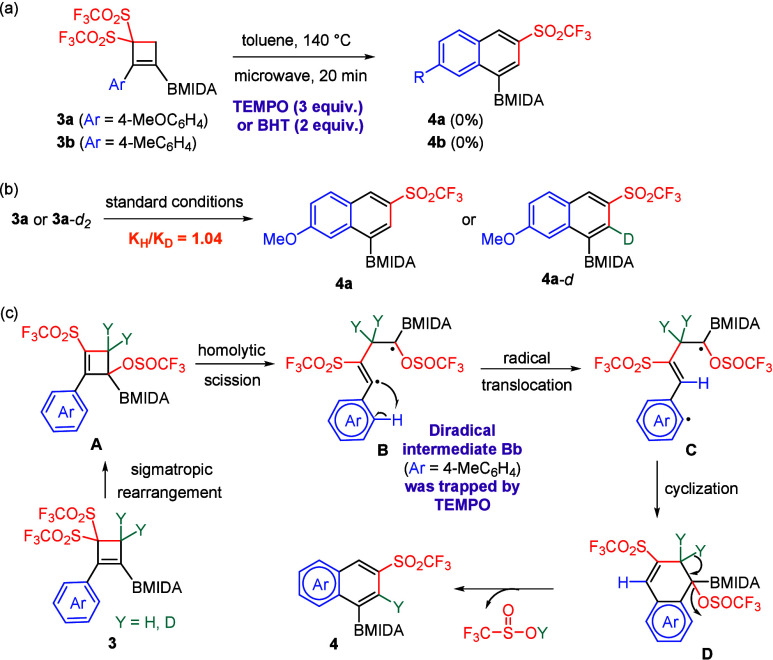
Possible Pathway for the Generation of Arenes **4**

To summarize, this work offered a convenient strategy for the controlled synthesis of triflylated cyclobutenyl MIDA boronates from readily available starting materials using a simple approach under mild and straightforward conditions. Interestingly, this protocol abolishes the need for a metal or photocatalyst, making it appropriate for large-scale applications. In addition, highly functionalized four-membered cyclobutenes can be further transformed into value-added compounds, namely, ring-expanded BMIDA-substituted naphthyl triflones. The one-pot conversion of BMIDA alkynes into borylated (triflyl)­naphthalenes was also viable.

## Supplementary Material



## Data Availability

The data underlying this study are available in the published article and its Supporting Information.

## Notes

Experimental procedures, characterization data of new compounds, copies of NMR spectra, and computational details (PDF)
