# Supplementary material for: Synthesis and Ring Expansion of Triflylated Cyclobutenyl MIDA Boronates
Source: Org Lett. 2026 Jun 25;28(27):8690–5. doi: 10.1021/acs.orglett.6c02345 (PMC13366694; doi:10.1021/acs.orglett.6c02345)
Supplement: Supplementary file 1 [file ol6c02345_si_001.pdf]

Supplementary Information for the paper

# Synthesis and Ring Expansion of Triflylated Cyclobutenyl MIDA Boronates

Sara Gallardo,[†,‡,a] Mireia Toledano-Pinedo,[‡,a] Pablo Pastor,[†] Cristina Aragoncillo,*[,†] José M. Alonso,*[,†] Hikaru Yanai,[§] and Pedro Almendros*[,‡]

[†]Grupo de Lactamas y Heterociclos Bioactivos, Unidad Asociada al CSIC por el IQOG, Departamento de Química Orgánica, Facultad de Química, Universidad Complutense de Madrid, 28040-Madrid, Spain

[‡]Instituto de Química Orgánica General, IQOG, CSIC, Juan de la Cierva 3, 28006-Madrid, Spain

[§]School of Pharmacy, Tokyo University of Pharmacy and Life Sciences, 1432-1 Horinouchi, Hachioji, Tokyo 192-0392, Japan

[a]These authors have equally contributed to this work

E-mail: caragoncillo@quim.ucm.es; josalo08@ucm.es; palmendros@iqog.csic.es

## Table of Contents

General Methods S2

Experimental Section S3–S38

Trapping experiment with TEMPO S39–S39

$^{1}H$ NMR, $^{13}C$ NMR, $^{11}B$ NMR, $^{19}F$ NMR, and Bidimensional Spectra S40–S135

DFT Calculations S136–S140

**General Methods**: $^{1}$H NMR and $^{13}$C NMR spectra were recorded on a Bruker Avance AVIII-700 with cryoprobe, Bruker AMX-500, Bruker Avance III HD-400, Bruker Avance-300, or Varian VRX-300S. NMR spectra were recorded in $CDCl_3$ solutions, except otherwise stated. Chemical shifts are given in ppm relative to TMS ($^{1}$H, 0.0 ppm), or $CDCl_3$ ($^{1}$H, 7.27 ppm; $^{13}$C, 76.9 ppm), or acetone-$d_6$ ($^{1}$H, 2.05 ppm; $^{13}$C, 206.3 ppm), or $CD_3CN$ ($^{1}$H, 1.94 ppm; $^{13}$C, 118.2 ppm), or DMSO-$d_6$ ($^{1}$H, 2.50 ppm; $^{13}$C, 39.5 ppm). Chemical shifts in $^{19}$F are given in ppm relative to (trifluoromethyl)benzene ($C_6H_5CF_3$) in $CDCl_3$ ($^{19}$F, –63.7 ppm). Chemical shifts in $^{11}$B are given in ppm relative to $BF_3{\cdot}OEt_2$ in $CDCl_3$ ($^{11}$B, 32.09 ppm). Structural assignments were made with additional information from NOESY, gCOSY, gTOCSY, gHSQC, and gHMBC experiments. Low and high resolution mass spectra were taken on an AGILENT 6520 Accurate-Mass QTOF LC/MS spectrometer using the electronic impact (EI) or electrospray modes (ES) unless otherwise stated. IR spectra were recorded on a FT/IR4X JASCO spectrometer. Microwave irradiation was carried out in a Monowave 300 from Anton Paar GmbH. The reaction temperatures during microwave heating were measured with an internal infrared sensor. Syntheses in the microwave reactor were conducted in sealed reaction vessels. For light-promoted reactions, the reaction vessel (borosilicate glass) was placed about 2 cm from a Kessil PR160L lamp (427 nm) and stirred (a fan was used to dissipate the heat generated by the lamp). Column chromatography was carried out using silica gel 60, 0.04-0.06 mm, for flash chromatography (230-400 mesh ASTM) provided by Scharlau. For reactions that require heating, a heating-on block was used. All commercially available compounds were used without further purification.

Pyridinium salt **1** was synthesized according to a literature procedure: Yanai, H.; Takahashi, Y.; Fukaya, H.; Dobashi, Y.; Matsumoto, T. *Chem. Commun.* **2013**, *49*, 10091.

To a solution of $Tf_2CH_2$ or $Tf_2D_2$ (281 mg, 1.00 mmol) in 1,2-dichloroethane (6.0 mL), paraformaldehyde (90% purity, 73.0 mg, 2.19 mmol) and 2-fluoropyridine (172 μL, 2.00 mmol) were added at room temperature. After being stirred for 8 h at 60 °C, the reaction mixture was concentrated under reduced pressure. The resulting residue was washed with $CHCl_3$ (1.0 mL x 3) to give zwitterion **1** in 91% yield (356 mg, 0.915 mmol).

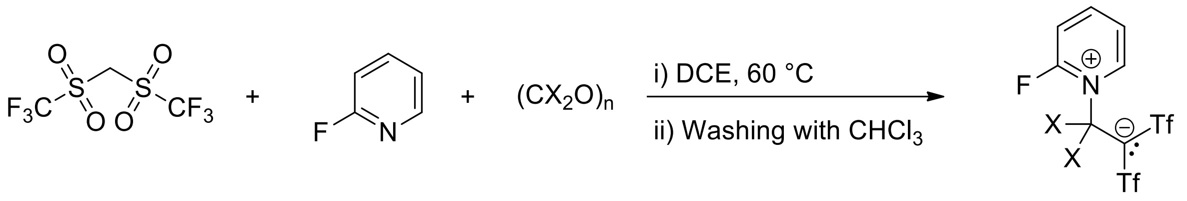


**1** (X=H); 91 %

**1-*d*2** (X=D); 86%

**Scheme S1**. Synthesis of Yanai's reagent **1**.

**BMIDA-alkynes (MIDA = *N*-methyliminodiacetyl) 2a, 2b, 2h, 2j, 2l, 2n, and 2s were synthesized as described in the literature. 2a, 2b, 2l, 2n, and 2s** (Struble, J. R.; Lee, S. J.; Burke, M. D. *Tetrahedron* **2010**, *66*, 4710–4718)**, 2h** (Fan, W.-X.; Li, J.-L.; Lv, W.-X.; Yang, L.; Li, Q.; Wang, H. *Chem. Commun.* **2020**, *56*, 82–85; Chen, Z.-H.; Su, X.-X.; Li, Q.; Wu, J.-Q.; Ou, T.-M.; Wang, H. *Org. Lett.* **2023**, *25*, 1099–1103)**, and 2j** (Halford-McGuff, J. M.; Cordes, D. B.; Watson, A. J. B. *Chem. Commun.* **2023**, *59*, 7759–7762).

**Novel BMIDA-alkynes 2c–g, 2i, 2k, 2m, 2o–r, and 2t–v were prepared using the following general procedure.**

An oven-dried microwave vial equipped with a stir bar was charged with $PdCl_2(PPh_3)_2$ (5 mol %), CuI (10 mol %), the aryl iodide **II** (1.16 equiv.) and THF (2 mL) at room

temperature. The solution was stirred for 5 minutes under argon atmosphere. Subsequently, a solution of ethynyl boronate **I** (80 mg, 0.442 mmol) in $Et_3N$ (0.4 mL) and MeCN (0.6 mL) was added dropwise via syringe under argon atmosphere at room temperature. The reaction mixture was stirred at 50ºC for 5 hours using microwave irradiation, until completed consumption of **I** (followed by TLC). The reaction mixture was cooled to room temperature, diluted with EtOAc (10 mL), filtered through a celite pad and concentrated under reduced pressure. The resulting crude was purified by column chromatography to afford the desired alkynyl MIDA boronate **2**.

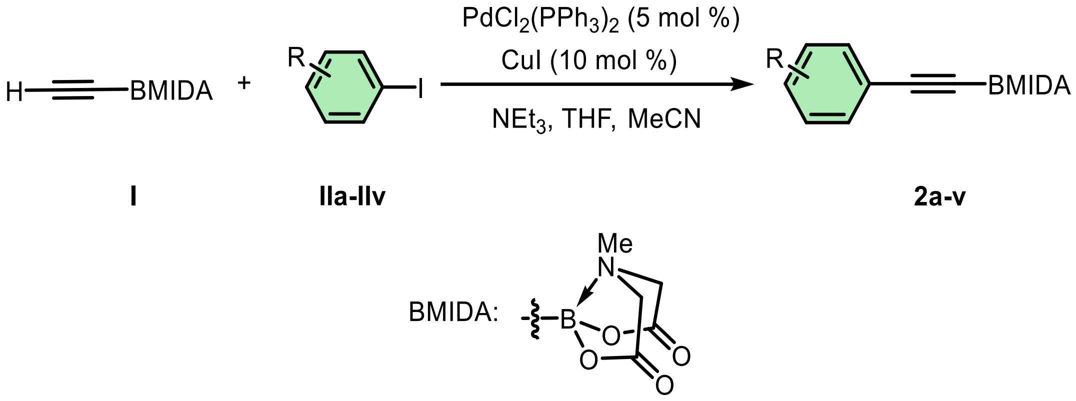


**Scheme S2.** Synthesis of alkynyl MIDA boronates **2.**

**Alkynyl MIDA Boronate 2c**

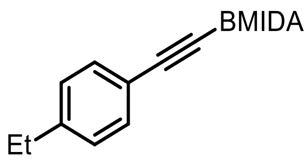


Following the general procedure and starting from 60 mg (0.33 mmol) of ethynyl boronate **I**, 55.2 mg (0.19 mmol) of compound **2c** (59%) were obtained as a colorless solid, after purification on column chromatography using *n*-hexane/AcOEt (1:3 → 1:8) as eluent; m.p. 228-230 ºC; $^1$H NMR (300 MHz, acetone-$d_6$, 25ºC): δ 7.40 (2H, d, *J* = 8.3 Hz, $CH_{Ar}$), 7.22 (2H, d, *J* = 8.5 Hz, $CH_{Ar}$), 4.32 (2H, d, *J* = 16.9 Hz, $CH_2$), 4.16 (2H, d, *J* = 16.9 Hz, $CH_2$), 3.31 (3H, s, $NCH_3$), 2.64 (2H, q, *J* = 7.6 Hz, $CH_2$), 1.20 (3H, t, *J* = 7.6

Hz, $CH_3$); $^{13}C\ \{^1H\}$ NMR (75 MHz, acetone-$d_6$, 25ºC): δ 168.6 (C=O), 146.0 ($C_{Ar}$), 132.6 ($CH_{Ar}$), 128.8 ($CH_{Ar}$), 121.3 ($C_{Ar}$), 62.4 (OC-$\underline{C}H_2$), 48.5 ($NCH_3$), 29.1 ($CH_2$), 15.8 ($CH_3$); HRMS (ESI-TOF) m/z: $[M-H]^-$ calcd for $C_{15}H_{15}BNO_4$: 284.1102; found 284.1108; IR ($cm^{-1}$): ν 1772 (C=O), 1568, 1204.

**Alkynyl MIDA Boronate 2d**

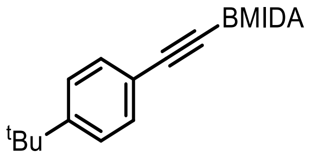


Following the general procedure and starting from 80 mg (0.44 mmol) of ethynyl boronate **I**, 78.0 mg (0.25 mmol) of compound **2d (**56%) were obtained as a colorless solid, after purification on column chromatography using *n*-hexane/AcOEt (1:9 → 0:1) as eluent; m.p. 257-259 ºC; $^1H$-RMN (300 MHz, acetone-$d_6$, 25ºC): δ 7.42 (4H, s, $CH_{Ar}$), 4.32 (2H, d, $J$ = 17.0 Hz, $CH_2$), 4.16 (2H, d, $J$ = 17.0 Hz, $CH_2$), 3.31 (3H, s, $NCH_3$), 1.30 (9H, s, $C(CH_3)_3$); $^{13}C\ \{^1H\}$ NMR (75 MHz, acetone-$d_6$, 25ºC): δ 168.6 (C=O), 152.7 ($C_{Ar}$), 132.4 ($CH_{Ar}$), 126.2 ($CH_{Ar}$), 121.1 ($C_{Ar}$), 62.4 (OC-$\underline{C}H_2$), 48.5 ($NCH_3$), 35.3 ($\underline{C}(CH_3)_3$), 31.4 ($C(\underline{C}H_3)_3$); HRMS (ESI-TOF) m/z: $[M-H]^-$ calcd for $C_{17}H_{19}BNO_4$: 312.1416; found 312.1420; IR ($cm^{-1}$): ν 1708 (C=O), 1416, 1020.

**Alkynyl MIDA Boronate 2e**

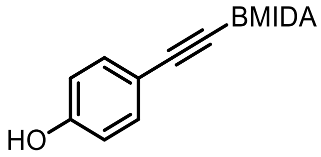


Following the general procedure and starting from 60 mg (0.33 mmol) of ethynyl boronate **I**, 48.6 mg (0.18 mmol) of compound **2e** (54%) were obtained as a yellow solid, after purification on column chromatography using *n*-hexane/AcOEt (1:10 → 0:1) as

eluent; m.p. 197-199 ºC; $^1$H NMR (300 MHz, acetone-d$_6$, 25ºC): δ 8.80 (1H, s, OH), 7.34 (2H, d, *J* = 8.6 Hz, CH$_{Ar}$), 6.82 (2H, d, *J* = 8.7 Hz, CH$_{Ar}$), 4.30 (2H, d, *J* = 16.9 Hz, CH$_2$), 4.14 (2H, d, *J* = 17.0 Hz, CH$_2$), 3.29 (3H, s, NCH$_3$); $^{13}$C {$^1$H} NMR (75 MHz, acetone-d$_6$, 25ºC): δ 168.6 (C=O), 158.9 (C$_{Ar}$), 134.2 (CH$_{Ar}$), 116.3 (CH$_{Ar}$), 114.8 (C$_{Ar}$), 62.3 (OC-$\underline{C}$H$_2$), 48.4 (NCH$_3$); HRMS (ESI-TOF) m/z: [M+H]$^+$ calcd for $C_{13}H_{13}BNO_5$: 274.0884; found 274.0879; IR (cm$^{-1}$): ν 1605 (C=O), 1514, 1280.

**Alkynyl MIDA Boronate 2f**

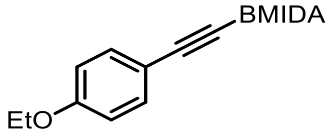


Following the general procedure and starting from 60 mg (0.33 mmol) of ethynyl boronate **I**, 76.9 mg (0.26 mmol) of compound **2f** (77%) were obtained as an orange solid, after purification on column chromatography using *n*-hexane/AcOEt (1:3 → 1:8) as eluent; m.p. 208-210 ºC; $^1$H NMR (300 MHz, acetone-d$_6$, 25ºC): δ 7.41 (2H, d, *J* = 8.9 Hz, CH$_{Ar}$), 6.90 (2H, d, *J* = 8.9 Hz, CH$_{Ar}$), 4.31 (2H, d, *J* = 16.9 Hz, CH$_2$), 4.15 (2H, d, *J* = 16.9 Hz, CH$_2$), 4.06 (2H, q, *J* = 7.0 Hz, CH$_2$), 3.30 (3H, s, NCH$_3$), 1.36 (3H, t, CH$_3$); $^{13}$C {$^1$H} NMR (75 MHz, acetone-d$_6$, 25ºC): δ 168.6 (C=O), 160.3 (C$_{Ar}$), 134.1 (CH$_{Ar}$), 115.8 (C$_{Ar}$), 115.3 (CH$_{Ar}$), 64.2 (CH$_2$), 62.3 (OC-$\underline{C}$H$_2$), 48.4 (NCH$_3$), 15.0 (CH$_3$); HRMS (ESI-TOF) m/z: [M+H]$^+$ calcd for $C_{15}H_{17}BNO_5$: 302.1197; found 302.1193; IR (cm$^{-1}$): ν 1765 (C=O), 1508, 1241.

**Alkynyl MIDA Boronate 2g**

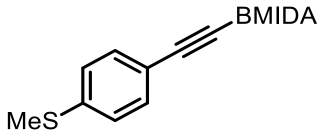

Following the general procedure and starting from 60 mg of ethynyl boronate **I**, 60 mg (0.2 mmol) of compound **2g** (61%) were obtained as a colorless oil, after purification on column chromatography using *n*-hexane/AcOEt (1:3 → 0:1) as eluent; $^{1}$H NMR (300 MHz, acetone-d$_6$, 25ºC): δ 7.41 (2H, d, *J* = 8.7 Hz, CH$_{Ar}$), 7.25 (2H, d, *J* = 8.7 Hz, CH$_{Ar}$), 4.32 (2H, d, *J* = 16.9 Hz, CH$_2$), 4.16 (2H, d, *J* = 17.0 Hz, CH$_2$), 3.31 (3H, s, NCH$_3$), 2.50 (3H, s, SCH$_3$); $^{13}$C {$^{1}$H} NMR (75 MHz, acetone-d$_6$, 25ºC): δ 168.6 (C=O), 141.1 (C$_{Ar}$), 132.9 (CH$_{Ar}$), 126.4 (CH$_{Ar}$), 120.0 (C$_{Ar}$), 62.4 (OC-$\underline{C}$H$_2$), 48.5 (NCH$_3$), 14.9 (CH$_3$); HRMS (ESI-TOF) m/z: [M+H]$^+$ calcd for $C_{14}H_{15}BNO_4S$: 304.0812; found 304.0806; IR (cm$^{-1}$): ν 1773 (C=O), 1279.

**Alkynyl MIDA Boronate 2i**

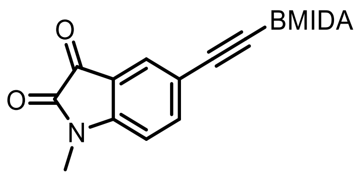


Following the general procedure and starting from 55 mg (0.30 mmol) of ethynyl boronate **I**, 69.0 mg (0.2 mmol) of compound **2i** (66%) were obtained as a red solid; m.p. 208-210 ºC; $^{1}$H-RMN (300 MHz, DMSO-d$_6$, 25ºC): δ 7.78 (1H, dd, *J* = 8.2, 1.8 Hz, CH$_{Ar}$), 7.63 (1H, d, *J* = 1.7 Hz, CH$_{Ar}$), 7.16 (1H, d, *J* = 8.2 Hz, CH$_{Ar}$), 4.32 (2H, d, *J* = 17.2 Hz, CH$_2$), 4.13 (2H, d, *J* = 17.1 Hz, CH$_2$), 3.15 (3H, s, NCH$_3$), 3.08 (3H, s, NCH$_3$); $^{13}$C {$^{1}$H} NMR (75 MHz, DMSO-d$_6$, 25ºC): δ 182.6 (C=O), 168.7 (C=O), 158.3 (C=O), 151.0 (C$_{Ar}$), 140.8 (CH$_{Ar}$), 126.9 (CH$_{Ar}$), 117.7 (C$_{Ar}$), 116.9 (C$_{Ar}$), 110.9 (CH$_{Ar}$), 98.1 (Ar-$\underline{C}_{sp}$), 61.4 (OC-$\underline{C}$H$_2$), 47.9 (NCH$_3$), 26.2 (NCH$_3$); HRMS (ESI-TOF) m/z: [M+H]$^+$ calcd for $C_{16}H_{14}BN_2O_6$: 341.0942; found 341.0942; IR (cm$^{-1}$): ν 1734 (C=O), 1619 (C=O), 1587, 1326.

**Alkynyl MIDA Boronate 2k**

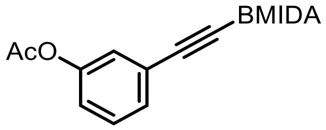


Following the general procedure and starting from 80 mg (0.44 mmol) of ethynyl boronate **I**, 85.0 mg (0.27 mmol) of compound **2k** (61%) were obtained as a colorless solid, after purification on column chromatography using *n*-hexane/AcOEt (1:5 → 0:1) as eluent; m.p. 187-190 ºC; $^{1}$H NMR (300 MHz, acetone-$d_6$, 25ºC): δ 7.38 (2H, m, $CH_{Ar}$), 7.25 (1H, m, $CH_{Ar}$), 7.15 (1H, m, $CH_{Ar}$), 4.33 (2H, d, *J* = 17.0 Hz, $CH_2$), 4.18 (2H, d, *J* = 16.9 Hz, $CH_2$), 3.32 (3H, s, $NCH_3$), 2.26 (3H, s, $OCOC\underline{H}_3$); $^{13}$C {$^{1}$H} NMR (75 MHz, acetone-$d_6$, 25ºC): δ 169.5 (OC=O), 168.5 (C=O), 151.8 ($C_{Ar}$), 130.4 ($CH_{Ar}$), 129.8 ($CH_{Ar}$), 125.9 ($CH_{Ar}$), 125.0 ($C_{Ar}$), 123.4 ($CH_{Ar}$), 62.4 (OC-$\underline{C}H_2$), 48.5 ($NCH_3$), 20.9 ($\underline{C}H_3CO_2$); HRMS (ESI-TOF) m/z: $[M+Na]^+$ calcd for $C_{15}H_{14}BNO_6Na$: 338.0809; found 338.0805; IR ($cm^{-1}$): ν 1771 (C=O), 1710 (C=O), 1465, 1287.

**Alkynyl MIDA Boronate 2m**

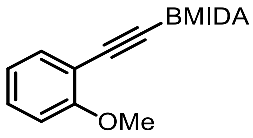


Following the general procedure and starting from 60 mg (0.33 mmol) of ethynyl boronate **I**, 41 mg (0.14 mmol) of compound **2m** (43%) were obtained as a colorless oil, after purification on column chromatography using *n*-hexane/AcOEt (1:1 → 1:7) as eluent; $^{1}$H NMR (300 MHz, acetone-$d_6$, 25ºC): δ 7.42 (1H, ddd, *J* = 7.6, 1.8, 0.5 Hz, $CH_{Ar}$), 7.35 (1H, ddd, *J* = 8.4, 7.4, 1.8 Hz, $CH_{Ar}$), 7.02 (1H, dd, *J* = 8.4, 1.0 Hz, $CH_{Ar}$), 6.92 (1H, td, *J* = 7.5, 1.0 Hz, $CH_{Ar}$), 4.32 (2H, d, *J* = 16.9 Hz, $CH_2$), 4.15 (2H, d, *J* = 16.9 Hz, $CH_2$), 3.85 (3H, s, $OCH_3$), 2.84 (3H, s, $NCH_3$); $^{13}$C {$^{1}$H} NMR (75 MHz, acetone-$d_6$, 25ºC): δ 168.6 (C=O), 161.5 ($C_{Ar}$), 134.2 ($CH_{Ar}$), 131.0 ($CH_{Ar}$), 121.1 ($CH_{Ar}$), 113.1

($C_{Ar}$), 111.8 ($CH_{Ar}$), 62.3 (OC-$\underline{C}H_2$), 56.0 ($OCH_3$), 48.5 ($NCH_3$); HRMS (ESI-TOF) m/z: $[M+Na]^+$ calcd for $C_{14}H_{14}BNO_5Na$: 310.0860; found 310.0857; IR ($cm^{-1}$): ν 1706 (C=O), 1261.

**Alkynyl MIDA Boronate 2o**

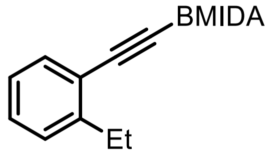


Following the general procedure and starting from 60 mg (0.33 mmol) of ethynyl boronate **I**, 47.7 mg (0.17 mmol) of compound **2o** (51%) were obtained as a colorless solid, after purification on column chromatography using *n*-hexane/AcOEt (1:3 → 1:8) as eluent; m.p. 173-175 ºC; $^1$H NMR (300 MHz, acetone-$d_6$, 25ºC): δ 7.46 (1H, m, $CH_{Ar}$), 7.28 (2H, m, $CH_{Ar}$), 7.18 (1H, m, $CH_{Ar}$), 4.34 (2H, d, $J$ = 17.0 Hz, $CH_2$), 4.18 (2H, d, $J$ = 16.9 Hz, $CH_2$), 3.34 (3H, s, $NCH_3$), 2.82 (2H, q, $CH_2$), 1.22 (3H, t, $J$ = 7.6 Hz, $CH_3$); $^{13}$C {$^1$H} NMR (75 MHz, acetone-$d_6$, 25ºC): δ 168.6 (C=O), 147.1 ($C_{Ar}$), 133.4 ($CH_{Ar}$), 129.8 ($CH_{Ar}$), 128.9 ($CH_{Ar}$), 126.6 ($CH_{Ar}$), 123.0 ($C_{Ar}$), 62.4 (OC-$\underline{C}H_2$), 48.5 ($NCH_3$), 28.2 ($CH_2$), 15.3 ($\underline{C}H_3$); HRMS (ESI-TOF) m/z: $[M-H]^-$ calcd for $C_{15}H_{15}BNO_4$: 284.1102; found 284.1099; IR ($cm^{-1}$): ν 1706 (C=O), 1222.

**Alkynyl MIDA Boronate 2p**

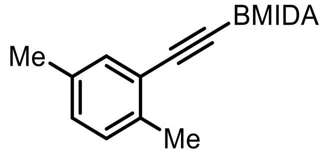


Following the general procedure and starting from 60 mg (0.33 mmol) of ethynyl boronate **I**, 66.2 mg (0.23 mmol) of compound **2p** (70%) were obtained as a colorless solid, after purification on column chromatography using *n*-hexane/AcOEt (1:2 → 0:1)

as eluent; m.p. 190-193 ºC; $^1$H NMR (300 MHz, acetone-$d_6$, 25ºC): δ 7.26 (1H, m, $CH_{Ar}$), 7.13 (1H, m, $CH_{Ar}$), 7.09 (1H, m, $CH_{Ar}$), 4.33 (2H, d, *J* = 17.0 Hz, $CH_2$), 4.17 (2H, d, *J* = 16.9 Hz, $CH_2$), 3.33 (3H, s, $NCH_3$), 2.37 (3H, s, $CH_3$), 2.26 (3H, s, $CH_3$); $^{13}$C {$^1$H} NMR (75 MHz, acetone-$d_6$, 25ºC): δ 168.6 (C=O), 137.9 ($C_{Ar}$), 135.9 ($C_{Ar}$), 133.4 ($CH_{Ar}$), 130.3 ($CH_{Ar}$), 130.2 ($CH_{Ar}$), 123.5 ($C_{Ar}$), 62.4 (OC-$\underline{C}H_2$), 48.6 ($NCH_3$), 20.7 ($CH_3$), 20.3 ($CH_3$); HRMS (ESI-TOF) m/z: $[M-H]^-$ calcd for $C_{15}H_{15}BNO_4$: 284.1102; found 284.1100; IR ($cm^{-1}$): ν 1761 (C=O), 1283.

**Alkynyl MIDA Boronate 2q**

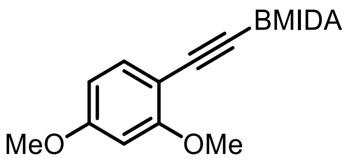


Following the general procedure and starting from 80 mg (0.44 mmol) of ethynyl boronate **I**, 86.0 mg (0.27 mmol) of compound **2q** (61%) were obtained as a green solid, after purification on column chromatography using *n*-hexane/AcOEt (1:7 → 0:1) as eluent; m.p. 183-185 ºC; $^1$H-RMN (300 MHz, acetone-$d_6$, 25ºC): δ 7.33 (1H, d, *J* = 8.4 Hz, $CH_{Ar}$), 6.56 (1H, d, *J* = 2.4 Hz, $CH_{Ar}$), 6.51 (1H, dd, *J* = 8.4, 2.4 Hz, $CH_{Ar}$), 4.30 (2H, d, *J* = 16.9 Hz, $CH_2$), 4.12 (2H, d, *J* = 16.9 Hz, $CH_2$), 3.84 (3H, s, $OCH_3$), 3.82 (3H, s, $OCH_3$), 3.30 (3H, s, $NCH_3$); $^{13}$C {$^1$H} NMR (75 MHz, acetone-$d_6$, 25ºC): δ 168.7 (C=O), 162.8 ($C_{Ar}$), 162.5 ($C_{Ar}$), 135.0 ($CH_{Ar}$), 106.0 ($CH_{Ar}$), 105.5 ($C_{Ar}$), 99.0 ($CH_{Ar}$), 62.3 (OC-$\underline{C}H_2$), 56.1 ($OCH_3$), 55.8 ($OCH_3$), 48.5 ($NCH_3$); HRMS (ESI-TOF) m/z: $[M+Na]^+$ calcd for $C_{15}H_{16}BNO_6Na$: 340.0966; found 340.0960; IR ($cm^{-1}$): ν 1772 (C=O), 1282.

**Alkynyl MIDA Boronate 2r**

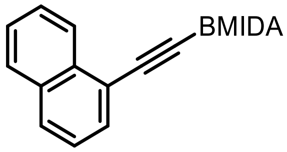


Following the general procedure and starting from 60 mg (0.33 mmol) of ethynyl boronate **I**, 54.0 mg (0.18 mmol) of compound **2r** (54%) were obtained as a colorless solid, after purification on column chromatography using *n*-hexane/AcOEt (1:3 → 1:7) as eluent; m.p. 211-213 ºC; $^1$H-RMN (300 MHz, acetone-$d_6$, 25ºC): δ 8.37 (1H, d, $J$ = 8.0 Hz, $CH_{Ar}$), 7.96 (2H, d, $J$ = 7.5 Hz, $CH_{Ar}$), 7.76 (1H, dd, $J$ = 7.1, 1.2 Hz, $CH_{Ar}$), 7.60 (2H, m, $CH_{Ar}$), 7.50 (1H, dd, $J$ = 8.3, 7.2 Hz, $CH_{Ar}$), 4.39 (2H, d, $J$ = 17.0 Hz, $CH_2$), 4.26 (2H, d, $J$ = 17.0 Hz, $CH_2$), 3.43 (3H, s, $NCH_3$); $^{13}$C {$^1$H} NMR (75 MHz, acetone-$d_6$, 25ºC): δ 168.6 (C=O), 134.2 ($C_{Ar}$), 134.1 ($C_{Ar}$), 131.8 ($CH_{Ar}$), 130.0 ($CH_{Ar}$), 129.3 ($CH_{Ar}$), 127.9 ($CH_{Ar}$), 127.5 ($CH_{Ar}$), 126.8 ($CH_{Ar}$), 126.2 ($CH_{Ar}$), 121.5 ($C_{Ar}$), 98.3 (Ar-$\underline{C}_{sp}$), 62.5 (OC-$\underline{C}H_2$), 48.7 ($CH_3$); HRMS (ESI-TOF) m/z: $[M+Na]^+$ calcd for $C_{17}H_{14}BNO_4Na$: 330.0911; found 330.0911; IR (cm$^{-1}$): ν 1702 (C=O), 1229.

**Alkynyl MIDA Boronate 2t**

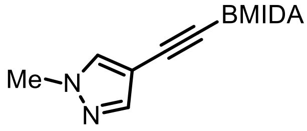


Following the general procedure and starting from 60 mg (0.33 mmol) of ethynyl boronate **I**, 42 mg (0.16 mmol) of compound **2t** (49%) were obtained as a yellow oil, after purification on column chromatography using *n*-hexane/AcOEt (1:1 → 0:1) as eluent; $^1$H NMR (300 MHz, acetone-$d_6$, 25ºC): δ 7.81 (1H, s, $CH_{Ar}$), 7.52 (1H, s, $CH_{Ar}$), 4.29 (2H, d, $J$ = 17.0 Hz, $CH_2$), 4.12 (2H, d, $J$ = 16.9 Hz, $CH_2$), 3.87 (3H, s, $NCH_3$), 3.25 (3H, s, $NCH_3$); $^{13}$C {$^1$H} NMR (75 MHz, acetone-$d_6$, 25ºC): δ 168.6 (C=O), 142.3 ($CH_{Ar}$), 134.3 ($CH_{Ar}$), 103.6 ($C_{Ar}$), 62.3 (OC-$\underline{C}H_2$), 48.4 ($NCH_3$), 39.1 ($NCH_3$); HRMS (ESI-TOF) m/z:

$[M-H]^-$ calcd for $C_{11}H_{11}BN_3O_4$: 260.0850; found 260.0842; IR ($cm^{-1}$): ν 1771 (C=O), 1288.

**Alkynyl MIDA Boronate 2u**

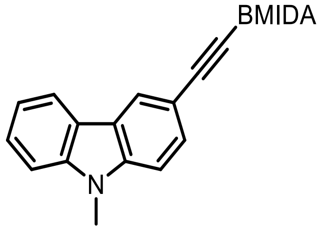


Following the general procedure and starting from 60 mg (0.33 mmol) of ethynyl boronate **I**, 75.9 mg (0.21 mmol) of compound **2u** (64%) were obtained as a colorless solid, after purification on column chromatography using *n*-hexane/AcOEt (1:3) as eluent; m.p. 218-220 ºC; $^1$H NMR (300 MHz, acetone-d$_6$, 25ºC): δ 8.30 (1H, m, $CH_{Ar}$), 8.18 (1H, m, $CH_{Ar}$), 7.59 (1H, m, $CH_{Ar}$), 7.55 (2H, m, $CH_{Ar}$), 7.51 (1H, m, $CH_{Ar}$), 7.25 (1H, m, $CH_{Ar}$), 4.34 (2H, d, *J* = 16.9 Hz, $CH_2$), 4.19 (2H, d, *J* = 16.9 Hz, $CH_2$), 3.92 (3H, s, $NCH_3$), 3.36 (3H, s, $NCH_3$); $^{13}$C {$^1$H} NMR (75 MHz, acetone-d$_6$, 25ºC): δ 168.7 (C=O), 142.4 ($C_{Ar}$), 141.7 ($C_{Ar}$), 130.2 ($CH_{Ar}$), 127.2 ($CH_{Ar}$), 124.9 ($CH_{Ar}$), 123.4 ($C_{Ar}$), 123.0 ($C_{Ar}$), 121.2 ($CH_{Ar}$), 120.3 ($CH_{Ar}$), 114.0 ($C_{Ar}$), 110.0 ($CH_{Ar}$), 109.8 ($CH_{Ar}$), 62.3 (OC-$\underline{C}H_2$), 48.5 ($NCH_3$), 28.5 ($NCH_3$); HRMS (ESI-TOF) m/z: $[M-H]^-$ calcd for $C_{20}H_{16}BN_2O_4$: 359.1212; found 359.1218; IR ($cm^{-1}$): ν 1705 (C=O), 1280, 1070.

**Alkynyl MIDA Boronate 2v**

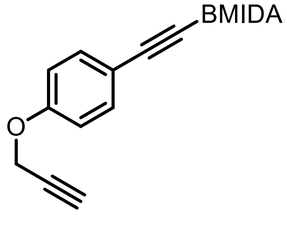

Adapting a reported procedure (Li, J.; Yang, F.; Ma, Y-T.; K. Ji; *Adv. Synth. Catal.* **2019**, *361*, 2148) and starting from 50 mg (0.18 mmol) of the alkynyl boronate **2e,** 32.6 mg (0.1 mmol) of compound **2v** (57%) were obtained as a colorless solid, after purification on column chromatography using *n*-hexane/AcOEt (1:1 → 0:1) as eluent; m.p. 190-193 ºC; $^{1}$H NMR (300 MHz, acetone-d$_6$, 25ºC): δ 7.45 (2H, d, *J*=9.0 Hz, CH$_{Ar}$), 7.00 (2H, d, *J* = 9.0 Hz, CH$_{Ar}$), 4.82 (2H, d, *J* = 2.4 Hz, OCH$_2$), 4.31 (2H, d, *J* = 16.9 Hz, CH$_2$), 4.15 (2H, d, *J* = 17.0 Hz, CH$_2$), 3.3 (3H, s, NCH$_3$), 3.10 (1H, t, *J* = 2.4 Hz, C≡C<u>H</u>); $^{13}$C {$^{1}$H} NMR (75 MHz, acetone-d$_6$, 25ºC): δ 168.6 (C=O), 158.8 (C$_{Ar}$), 134.0 (CH$_{Ar}$), 116.8 (C$_{Ar}$), 115.8 (CH$_{Ar}$), 79.3 (C≡C), 77.3 (C≡C), 62.3 (OC-<u>C</u>H$_2$), 56.4 (CH$_2$), 48.4 (NCH$_3$); HRMS (ESI-TOF) m/z: [M+Na]$^+$ calcd for $C_{16}H_{14}BNO_5Na$: 334.0860; found 334.0855; IR (cm$^{-1}$): ν 1709 (C=O), 1263.

**General procedure for the synthesis of BMIDA-cyclobutenes 3.**

The corresponding BMIDA-alkyne **2** (1 equiv.), zwitterion **1** or **1**-*d$_2$* (1.05 equiv.) and acetonitrile (8 mL/mmol) were added to an oven-dried round bottom flask. The reaction mixture was stirred at room temperature under argon atmosphere until disappearance of the starting alkyne **2** as monitored by TLC. Then, the mixture was concentrated under reduced pressure. Chromatography of the residue eluting with *n*-hexane/ethyl acetate mixtures gave analytically pure compounds. Spectroscopic and analytical data for bis(triflyl)-decorated BMIDA-cyclobutenes **3** follow.

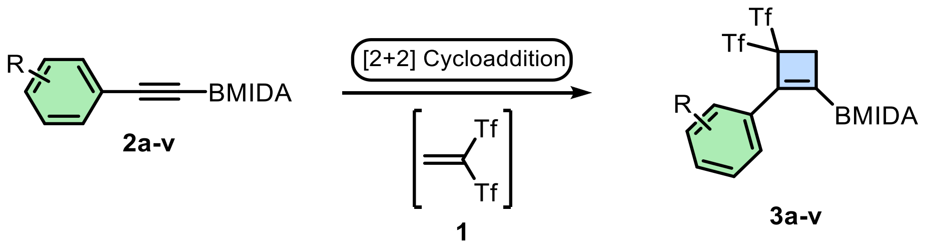


**Scheme S3.** Synthesis of BMIDA cyclobutenes **3**.

**MIDA boronate cyclobutene 3a**

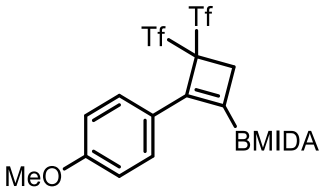


Following the general procedure and starting from 38.3 mg (0.13 mmol) of alkyne **2a**, 63 mg (0.11 mmol) of compound **3a** (82 %) were obtained as a brown solid, after purification on column chromatography using *n*-hexane/AcOEt (1:4 → 0:1) as eluent; m.p. 153-155 ºC; $^{1}$H-RMN (300 MHz, acetone-$d_6$, 25ºC): δ 7.63 (2H, d, $J$ = 8.9 Hz, $CH_{Ar}$), 6.98 (2H, d, $J$ = 8.9 Hz, $CH_{Ar}$), 4.39 (2H, d, $J$ = 17.1 Hz, $CH_2$), 4.12 (2H, d, $J$ = 17.1 Hz, $CH_2$), 3.85 (3H, s, $OCH_3$), 3.55 (2H, s, $CH_2$), 3.20 (3H, s, $NCH_3$); $^{13}$C {$^{1}$H} NMR (75 MHz, $CDCl_3$, 25ºC): δ 166.8 (C=O), 161.3 ($C_{Ar}$), 147.2 (B-C=$\underline{C}$), 129.8 ($CH_{Ar}$), 123.6 ($C_{Ar}$), 119.9 (q, $J$ = 331.1 Hz, $CF_3$), 114.7 ($CH_{Ar}$), 88.4 ($CTf_2$), 62.3 (OC-$\underline{C}H_2$), 55.5 ($OCH_3$), 47.3 ($NCH_3$), 35.7 ($CH_2$); $^{19}$F NMR (282 MHz, acetone-$d_6$, 25ºC): δ –71.0 (3F, s, $CF_3$); HRMS (ESI-TOF) m/z: $[M-H]^-$ calcd for $C_{18}H_{15}BF_6NO_9S_2$: 578.0194; found 578.0186; IR ($cm^{-1}$): ν 1772 (C=O), 1378, 1194, 1100, 1048.

**MIDA boronate cyclobutene 3b**

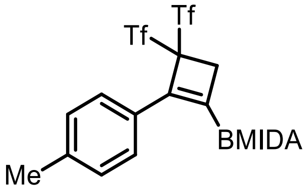


Following the general procedure and starting from 25 mg (0.09 mmol) of alkyne **2b**, 27 mg (0.05 mmol) of compound **3b** (53%) were obtained as a yellow solid, after purification on column chromatography using *n*-hexane/AcOEt (2:5) as eluent; m.p. 97-100 ºC; $^{1}$H NMR (300 MHz, acetone-$d_6$, 300 MHz, 25ºC): δ 7.55 (2H, d, $J$ = 8.3 Hz, $CH_{Ar}$), 7.25 (2H, d, $J$ = 7.9 Hz, $CH_{Ar}$), 4.39 (2H, d, $J$ = 17.1 Hz, $CH_2$), 4.11 (2H, d, $J$ = 17.1 Hz, $CH_2$),

3.57 (2H, s, $CH_2$), 3.20 (3H, s, $NCH_3$), 2.36 (3H, s, $CH_3$); $^{13}C$ {$^1H$} NMR (75 MHz, acetone-$d_6$, 25ºC): δ 168.3 (C=O), 146.2 (B-C=$\underline{C}$), 141.1($C_{Ar}$), 130.1 ($CH_{Ar}$), 129.4 ($C_{Ar}$), 128.9 ($CH_{Ar}$), 120.7 (q, $J$ = 330.7 Hz, $CF_3$), 89.1 ($CTf_2$), 63.3 (OC-$\underline{C}H_2$), 48.1 ($NCH_3$), 36.2 ($CH_2$), 21.3 ($CH_3$); $^{19}F$ NMR (282 MHz, acetone-$d_6$, 25ºC): δ –71.0 (3F, s, $CF_3$); HRMS (ESI-TOF) m/z: $[M-H]^-$ calcd for $C_{18}H_{15}BF_6NO_8S_2$: 562.0245; found 562.0238; IR ($cm^{-1}$): ν 1776 (C=O), 1378, 1198, 1102, 1055.

**MIDA boronate cyclobutene 3c**

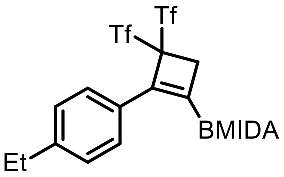


Following the general procedure and starting from 31.5 mg (0.11 mmol) of alkyne **2c**, 41.2 mg (0.07 mmol) of compound **3c** (65%) were obtained as a yellow oil, after purification on column chromatography using *n*-hexane/AcOEt (1:3) as eluent; $^1H$ NMR (300 MHz, acetone-$d_6$, 25ºC): δ 7.60 (2H, d, $J$ = 8.3 Hz, $CH_{Ar}$), 7.29 (2H, d, $J$ = 8.6 Hz, $CH_{Ar}$), 4.39 (2H, d, $J$ = 17.1 Hz, $CH_2$), 4.12 (2H, d, $J$ = 17.1 Hz, $CH_2$), 3.57 (2H, s, $CH_2$), 3.20 (3H, s, $NCH_3$), 2.68 (2H, q, $J$ = 7.6 Hz, $CH_2$), 1.20 (3H, t, $J$ = 7.6 Hz, $CH_3$); $^{13}C$ {$^1H$} NMR (75 MHz, acetone-$d_6$, 25ºC): δ 168.3 (C=O), 147.3 ($C_{Ar}$), 146.2 (B-C=$\underline{C}$), 129.6 ($C_{Ar}$), 129.0 ($CH_{Ar}$), 128.9 ($CH_{Ar}$), 120.8 (q, $J$ = 331.0 Hz, $\underline{C}F_3$), 89.0 ($CTf_2$), 63.3 (OC-$\underline{C}H_2$), 48.1 ($NCH_3$), 36.2 ($CH_2$), 15.5 ($CH_3$); $^{19}F$ NMR (282 MHz, acetone-$d_6$, 25ºC): δ –71.0 (3F, s, $CF_3$); HRMS (ESI-TOF) m/z: $[M+Na]^+$ calcd for $C_{19}H_{18}BF_6NNaO_8S_2$: 600.0363; found 600.0370; IR ($cm^{-1}$): ν 1768 (C=O), 1378, 1190, 1099, 1049.

**MIDA boronate cyclobutene 3d**

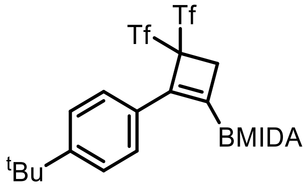


Following the general procedure and starting from 30.3 mg (0.1 mmol) of alkyne **2d**, 46.6 mg (0.08 mmol) of compound **3d** (80%) were obtained as a colorless solid, after purification on column chromatography using *n*-hexane/AcOEt (1:2 → 0:1) as eluent; m.p. 168-170 ºC; $^{1}$H-RMN (300 MHz, acetone-d$_6$, 25ºC): δ 7.65 (2H, d, *J* = 8.5 Hz, CH$_{Ar}$), 7.48 (2H, d, *J* = 8.7 Hz, CH$_{Ar}$), 4.40 (2H, d, *J* = 17.2 Hz, CH$_2$), 4.15 (2H, d, *J* = 17.2 Hz, CH$_2$), 3.58 (2H, s, CH$_2$), 3.21 (3H, s, NCH$_3$), 1.33 (9H, s, C(CH$_3$)$_3$); $^{13}$C {$^{1}$H} NMR (75 MHz, acetone-d$_6$, 25ºC): δ 168.3 (C=O), 154.0 (C$_{Ar}$), 145.9 (B-C=<u>C</u>), 129.3 (C$_{Ar}$), 128.7 (CH$_{Ar}$), 126.3 (CH$_{Ar}$), 120.8 (q, *J* = 330.8 Hz, CF$_3$), 88.9 (CTf$_2$), 63.3 (OC-<u>C</u>H$_2$), 48.0 (NCH$_3$), 36.3 (<u>C</u>(CH$_3$)$_3$), 35.4 (CH$_2$), 31.3 (C(<u>C</u>H$_3$)$_3$); $^{19}$F NMR (282 MHz, acetone-d$_6$, 25ºC): δ –71.0 (3F, s, CF$_3$); HRMS (ESI-TOF) m/z: [M+Na]$^+$ calcd for $C_{21}H_{22}BF_6NO_8S_2Na$: 628.0680; found 628.0685; IR (cm$^{-1}$): ν 1780 (C=O), 1380, 1195, 1101, 1048.

**MIDA boronate cyclobutene 3e**

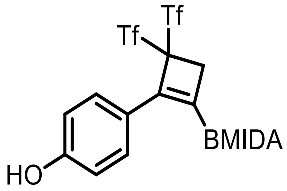


Following the general procedure and starting from 24.2 mg (0.09 mmol) of alkyne **2e**, 34.4 mg (0.06 mmol) of compound **3e** (69%) were obtained as a brown oil, after purification on column chromatography using *n*-hexane/AcOEt (1:2 → 1:8) as eluent; $^{1}$H NMR (300 MHz, acetone-d$_6$, 25ºC): δ 7.54 (2H, d, *J* = 8.8 Hz, CH$_{Ar}$), 6.88 (2H, d, *J* = 8.8 Hz, CH$_{Ar}$), 4.38 (2H, d, *J* = 17.2 Hz, CH$_2$), 4.11 (2H, d, *J* = 17.1 Hz, CH$_2$), 3.53 (2H, s,

$CH_2$), 3.19 (3H, s, $NCH_3$); $^{13}C\{^1H\}$ NMR (75 MHz, acetone-$d_6$, 25ºC): δ 168.4 (C=O), 159.9 ($C_{Ar}$), 146.2 (B-C=<u>C</u>), 130.8 ($CH_{Ar}$), 123.6 ($C_{Ar}$), 120.8 (q, *J* = 330.8 Hz, <u>C</u>$F_3$), 116.3 ($CH_{Ar}$), 89.1 ($CTf_2$), 63.3 (OC-<u>C</u>$H_2$), 48.0 ($NCH_3$), 36.0 ($CH_2$); $^{19}F$ NMR (282 MHz, acetone-$d_6$, 25ºC): δ –71.0 (3F, s, $CF_3$); HRMS (ESI-TOF) m/z: $[M+Na]^+$ calcd for $C_{17}H_{14}BF_6NNaO_9S_2$: 588.0005; found 588.0016; IR ($cm^{-1}$): ν 1773 (C=O), 1377, 1194, 1099, 1042.

**MIDA boronate cyclobutene 3f**

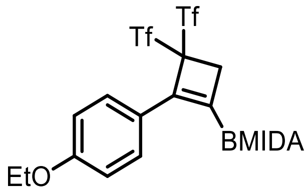


Following the general procedure and starting from 34.2 mg (0.11 mmol) of alkyne **2f**, 32.6 mg (0.05 mmol) of compound **3f** (50%) were obtained as a yellow oil, after purification on column chromatography using *n*-hexane/AcOEt (1:3) as eluent; $^1H$ NMR (300 MHz, acetone-$d_6$, 25ºC): δ 7.61 (2H, d, *J* = 8.9 Hz, $CH_{Ar}$), 6.96 (2H, d, *J* = 8.9 Hz, $CH_{Ar}$), 4.38 (2H, d, *J* = 17.1 Hz, $CH_2$), 4.13 (2H, d, *J* = 17.1 Hz, $CH_2$), 4.10 (2H, q, *J* = 7.1 Hz, $CH_2$), 3.54 (2H, s, $CH_2$), 3.19 (3H, s, $NCH_3$), 1.38 (3H, t, $CH_3$); $^{13}C\{^1H\}$ NMR (75 MHz, acetone-$d_6$, 25ºC): δ 168.4 (C=O), 161.2 ($C_{Ar}$), 145.9 (B-C=<u>C</u>), 130.7 ($CH_{Ar}$), 124.4 ($C_{Ar}$), 120.8 (q, *J* = 331.0 Hz, <u>C</u>$F_3$), 115.2 ($CH_{Ar}$), 89.1 ($CTf_2$), 64.3 ($CH_2$), 63.3 (OC-<u>C</u>$H_2$), 48.0 ($NCH_3$), 36.1 ($CH_2$), 15.0 ($CH_3$); $^{19}F$ NMR (282 MHz, acetone-$d_6$, 25ºC): δ –71.0 (3F, s, $CF_3$); HRMS (ESI-TOF) m/z: $[M+Na]^+$ calcd for $C_{19}H_{18}BF_6NO_9S_2Na$: 616.0316 ; found 616.0308; IR ($cm^{-1}$): ν 1758 (C=O), 1377, 1186, 1097, 1036.

**MIDA boronate cyclobutene 3g**

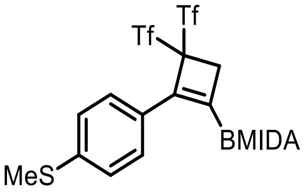


Following the general procedure and starting from 24 mg (0.08 mmol) of alkyne **2g**, 24.7 mg (0.042 mmol) of compound **3g** (53%) were obtained as a green oil, after purification on column chromatography using *n*-hexane/AcOEt (1:3 → 1:8) as eluent; $^{1}$H NMR (300 MHz, acetone-d$_6$, 25ºC): δ 7.61 (2H, d, *J* = 8.6 Hz, $CH_{Ar}$), 7.30 (2H, d, *J* = 8.8 Hz, $CH_{Ar}$), 4.40 (2H, d, *J* = 17.1 Hz, $CH_2$), 4.15 (2H, d, *J* = 17.2 Hz, $CH_2$), 3.58 (2H, s, $CH_2$), 3.21 (3H, s, $NCH_3$), 2.54 (3H, s, $SCH_3$); $^{13}$C {$^{1}$H} NMR (75 MHz, acetone-d$_6$, 25ºC): δ 168.3 (C=O), 145.6 (B-C=$\underline{C}$), 142.9 ($C_{Ar}$), 129.3 ($CH_{Ar}$), 128.2 ($C_{Ar}$), 126.1 ($CH_{Ar}$), 120.8 (q, *J* = 330.8 Hz, $\underline{C}F_3$), 89.0 ($CTf_2$), 63.3 (OC-$\underline{C}H_2$), 48.1 ($NCH_3$), 36.3 ($CH_2$), 14.6 ($CH_3$); $^{19}$F NMR (282 MHz, acetone-d$_6$, 25ºC): δ –71.0 (3F, s, $CF_3$); HRMS (ESI-TOF) m/z: $[M-H]^-$ calcd for $C_{18}H_{15}BF_6NO_8S_3$: 593.9966; found 593.9965; IR (cm$^{-1}$): ν 1759 (C=O), 1375, 1186, 1097, 1037.

**MIDA boronate cyclobutene 3h**

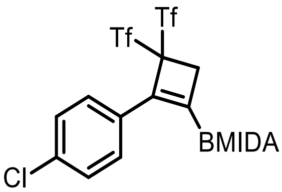


Following the general procedure and starting from 29.7 mg (0.103 mmol) of alkyne **2h**, 44.5 mg (0.08 mmol) of compound **3h** (77%) were obtained as a colorless oil, after purification on column chromatography using *n*-hexane/AcOEt (1:3 → 1:3) as eluent; $^{1}$H NMR (300 MHz, acetone-d$_6$, 25ºC): δ 7.64 (2H, d, *J* = 8.7 Hz, $CH_{Ar}$), 7.49 (2H, d, *J* = 8.8 Hz, $CH_{Ar}$), 4.40 (2H, d, *J* = 17.1 Hz, $CH_2$), 4.16 (2H, d, *J* = 17.2 Hz, $CH_2$), 3.62 (2H, s, $CH_2$), 3.24 (3H, s, $NCH_3$); $^{13}$C {$^{1}$H} NMR (75 MHz, acetone-d$_6$, 25ºC): δ 168.2 (C=O),

145.0 (B-C=C), 136.5 ($C_{Ar}$), 130.9 ($CH_{Ar}$), 129.6 ($CH_{Ar}$), 120.7 (q, *J* = 331.0 Hz, $CF_3$), 89.1 ($CTf_2$), 63.3 (OC-$CH_2$), 48.1 ($NCH_3$), 36.4 ($CH_2$); $^{19}$F NMR (282 MHz, acetone-$d_6$, 25ºC): δ –70.9 (3F, s, $CF_3$); HRMS (ESI-TOF) m/z: $[M-H]^-$ calcd for $C_{17}H_{12}BClF_6NO_8S_2$: 581.9699; found 581.9693; IR ($cm^{-1}$): ν 1738 (C=O), 1384, 1168, 1105, 1033.

**MIDA boronate cyclobutene 3i**

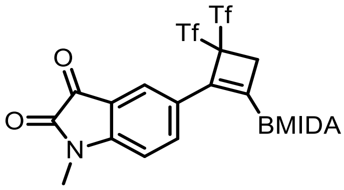


Following the general procedure and starting from 37.7 mg (0.11 mmol) of alkyne **2i**, 43 mg (0.07 mmol) of compound **3i** (61%) were obtained as a yellow solid, after purification on column chromatography using $Et_2O$/AcOEt (1:10 → 0:1) as eluent; m.p. 162-165 ºC; $^1$H-RMN (300 MHz, acetone-$d_6$, 25ºC): δ 7.93 (1H, dd, *J* = 8.3, 2.0 Hz, $CH_{Ar}$), 7.80 (1H, d, *J* = 2.0 Hz, $CH_{Ar}$), 7.23 (1H, d, *J* = 8.3 Hz, $CH_{Ar}$), 4.42 (2H, d, *J* = 17.1 Hz, $CH_2$), 4.20 (2H, d, *J* = 17.2 Hz, $CH_2$), 3.63 (s, 2H, $CH_2$), 3.29 (3H, s, $CH_3$), 3.26 (3H, s, $CH_3$); $^{13}$C {$^1$H} NMR (75 MHz, acetone-$d_6$, 25ºC): δ 183.7 (C=O), 168.2 (C=O), 158.9 (C=O), 153.6 ($C_{Ar}$), 144.6 (B-C=C), 139.2 ($CH_{Ar}$), 127.0 ($C_{Ar}$), 124.6 ($CH_{Ar}$), 120.7 (q, *J* = 330.6 Hz, $CF_3$), 118.3 ($C_{Ar}$), 111.4 ($CH_{Ar}$), 89.2 ($CTf_2$), 63.2 (OC-$CH_2$), 48.1 ($NCH_3$), 36.3 ($CH_2$), 26.5 ($CH_3$); $^{19}$F NMR (282 MHz, acetone-$d_6$, 25ºC): δ –70.9 (3F, s, $CF_3$); HRMS (ESI-TOF) m/z: $[M+Na]^+$ calcd for $C_{20}H_{15}BF_6N_2O_{10}S_2Na$: 655.0061; found 655.0050; IR ($cm^{-1}$): ν 1760 (C=O), 1736 (C=O), 1620 (C=O), 1381, 1192, 1105, 1024.

**MIDA boronate cyclobutene 3j**

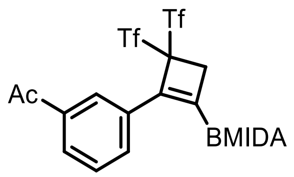


Following the general procedure and starting from 14.6 mg (0.05 mmol) of alkyne **2j**, 12.9 mg (0.02 mmol) of compound **3j** (45%) were obtained as a yellow oil, after purification on column chromatography using *n*-hexane/AcOEt (1:3) as eluent; $^1$H-RMN (300 MHz, acetone-d$_6$, 25ºC): δ 8.30 (1H, t, *J* = 1.8 Hz, CH$_{Ar}$), 8.05 (1H, ddd, *J* = 7.8, 1.7, 1.1 Hz, CH$_{Ar}$), 7.88 (1H, ddd, *J* = 7.8, 1.9, 1.1 Hz, CH$_{Ar}$), 7.61 (1H, td, *J* = 7.8, 0.6 Hz, CH$_{Ar}$), 4.42 (2H, d, *J* = 17.1 Hz, CH$_2$), 4.17 (2H, d, *J* = 17.1 Hz, CH$_2$), 3.66 (2H, s, CH$_2$), 3.25 (s, 3H, NCH$_3$), 2.62 (3H, s, OC-CH$_3$); $^{13}$C {$^1$H} NMR (176 MHz, acetone-d$_6$, 25ºC): δ 197.3 (CH$_3$<u>C</u>O), 168.3 (C=O), 145.3 (B-C=<u>C</u>), 138.3 (C$_{Ar}$), 133.1 (CH$_{Ar}$), 132.3 (C$_{Ar}$), 130.1 (CH$_{Ar}$), 129.9 (CH$_{Ar}$), 129.3 (CH$_{Ar}$), 120.7 (q, *J* = 330.6 Hz, CF$_3$), 89.0 (CTf$_2$), 63.6 (OC-<u>C</u>H$_2$), 48.1 (NCH$_3$), 36.4 (CH$_2$), 26.8 (OC-CH$_3$); $^{19}$F NMR (282 MHz, acetone-d$_6$, 25ºC): δ –70.9 (3F, s, CF$_3$); $^{11}$B-RMN (160 MHz, acetone-d$_6$, 25ºC): δ 7.9; HRMS (ESI-TOF) m/z: [M-H]$^-$ calcd for $C_{19}H_{15}BF_6NO_9S_2$: 590.0195; found 590.0190; IR (cm$^{-1}$): ν 1772 (C=O), 1685 (C=O), 1381, 1212, 1104, 1056.

**MIDA boronate cyclobutene 3k**

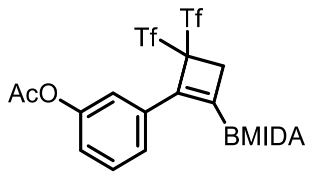


Following the general procedure and starting from 30 mg (0.1 mmol) of alkyne **2k**, 14 mg (0.02 mmol) of compound **3k** (24%) were obtained as a colorless solid, after purification on column chromatography using *n*-hexane/AcOEt (1:3 → 1:4) as eluent; m.p. 183-186 ºC; $^1$H-RMN (300 MHz, acetone-d$_6$, 25ºC): δ 7.60 –7.43 (3H, m, CH$_{Ar}$), 7.24 (1H, ddd, *J* = 7.6, 2.3, 1.5 Hz, CH$_{Ar}$), 4.40 (2H, d, *J* = 17.2 Hz, CH$_2$), 4.11 (2H, d, *J*

= 17.1 Hz, $CH_2$), 3.61 (2H, s, $CH_2$), 3.17 (3H, s, $NCH_3$), 2.27 (3H, s, $OCOCH_3$); $^{13}C\ \{^1H\}$ NMR (75 MHz, acetone-$d_6$, 25ºC): δ 169.8 (C=O), 168.3 (C=O), 151.9 ($C_{Ar}$), 144.8 (B-C=C), 133.2 ($C_{Ar}$), 130.8 ($CH_{Ar}$), 126.2 ($CH_{Ar}$), 124.4 ($CH_{Ar}$), 122.2 ($CH_{Ar}$), 120.7 (q, $J$ = 330.9 Hz, $CF_3$), 88.7 ($CTf_2$), 63.5 (OC-$CH_2$), 48.1 ($NCH_3$), 36.6 ($CH_2$), 20.9 ($CH_3CO_2$). $^{19}F$ NMR (282 MHz, acetone-$d_6$, 25ºC): δ –70.9 (3F, s, $CF_3$). HRMS (ESI-TOF) m/z: $[M+H]^+$ calcd for $C_{19}H_{17}BF_6NO_{10}S_2$: 608.0289; found 608.0296; IR ($cm^{-1}$): ν 1776 (C=O), 1742 (C=O), 1376, 1186, 1109, 1016.

**MIDA boronate cyclobutene 3l**

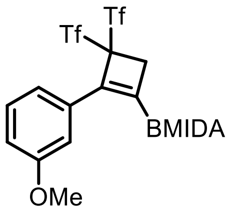


Following the general procedure and starting from 19.5 mg (0.07 mmol) of alkyne **2l**, 10.9 mg (0.02 mmol) of compound **3l** (28%) were obtained as a yellow solid, after purification on column chromatography using *n*-hexane/AcOEt (2:5) as eluent; m.p. 143-145 ºC; $^1H$ NMR (700 MHz, acetone-$d_6$, 25ºC): δ 7.35 (1H, t, $J$ = 8.0 Hz, $CH_{Ar}$), 7.28 (1H, t, $J$ = 7.3 Hz, $CH_{Ar}$), 7.25 (1H, dt, $J$ = 7.8, 1.2 Hz, $CH_{Ar}$), 7.01 (1H, ddd, $J$ = 8.3, 2.5, 1.0 Hz, $CH_{Ar}$), 4.40 (2H, d, $J$ = 17.1 Hz, $CH_2$), 4.14 (2H, d, $J$ = 17.1 Hz, $CH_2$), 3.83 (3H, s, $OCH_3$), 3.60 (2H, s, $CH_2$). 3.20 (3H, s, $NCH_3$); $^{13}C\ \{^1H\}$ NMR (176 MHz, acetone-$d_6$, 25ºC): δ 168.4 (C=O), 160.6 ($C_{Ar}$), 146.0 (B-C=C), 133.3 ($C_{Ar}$), 130.6 ($CH_{Ar}$), 121.1 ($CH_{Ar}$), 120.3 (q, $J$ = 330.7 Hz, $CF_3$), 117.3 ($CH_{Ar}$), 114.6 ($C_{Ar}$), 113.7 ($CH_{Ar}$), 89.0 ($CTf_2$), 63.3 (OC-$CH_2$), 55.6 ($OCH_3$), 48.1 ($NCH_3$), 36.3 ($CH_2$); $^{19}F$ NMR (282 MHz, acetone-$d_6$, 25ºC): δ –70.9 (3F, s, $CF_3$); HRMS (ESI-TOF) m/z: $[M+Na]^+$ calcd for $C_{18}H_{16}BF_6NO_9S_2Na$: 602.0159; found 602.0167; IR ($cm^{-1}$): ν 1771 (C=O), 1379, 1208, 1102, 1054.

**MIDA boronate cyclobutene 3m**

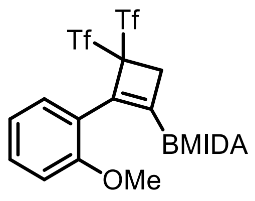


Following the general procedure and starting from 21.1 mg (0.07 mmol) of alkyne **2m**, 14.9 mg (0.026 mmol) of compound **3m** (36%) were obtained as a yellow solid, after purification on column chromatography using *n*-hexane/AcOEt (1:3) as eluent; m.p. 108-110 ºC; $^{1}H$ NMR (700 MHz, acetonitrile-$d_3$, 25ºC): δ 7.41 (1H, ddd, *J* = 8.7, 7.5, 1.8, Hz, $CH_{Ar}$), 7.32 (1H, dd, *J* = 7.6, 1.6 Hz, $CH_{Ar}$), 6.99 (1H, d, *J* = 8.7 Hz, $CH_{Ar}$), 6.97 (1H, dd, *J* = 7.5, 1.0 Hz, $CH_{Ar}$), 4.00 (2H, d, *J* = 17.0 Hz, $CH_2$), 3.76 (3H, s, $OCH_3$), 3.74 (2H, d, *J* = 17.1 Hz, $CH_2$), 3.50 (2H, s, $CH_2$), 3.05 (3H, s, $NCH_3$); $^{13}C$ {$^{1}H$} NMR (176 MHz, acetonitrile-$d_3$, 25ºC): δ 168.5 (C=O), 158.2 ($C_{Ar}$), 144.4 (B-C=<u>C</u>), 132.7 ($CH_{Ar}$), 131.4 ($CH_{Ar}$), 121.6 ($C_{Ar}$), 121.3 ($CH_{Ar}$), 120.8 (q, *J* = 330.7 Hz, <u>C</u>$F_3$), 112.3 ($CH_{Ar}$), 90.8 ($CTf_2$), 63.1 (OC-<u>C</u>$H_2$), 56.0, 48.3 ($NCH_3$), 36.1 ($CH_2$); $^{19}F$ NMR (282 MHz, acetonitrile-$d_3$, 25ºC): δ –70.42 (3F, s, $CF_3$); HRMS (ESI-TOF) m/z: $[M-H]^-$ calcd for $C_{18}H_{15}BF6NO_9S_2$: 578.0194; found 578.0200; IR ($cm^{-1}$): ν 1778 (C=O), 1382, 1196, 1103, 1052.

**MIDA boronate cyclobutene 3n**

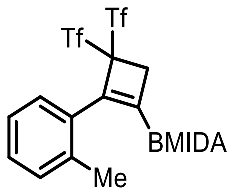


Following the general procedure and starting from 20 mg (0.07 mmol) of alkyne **2n**, 29 mg (0.05 mmol) of compound **3n** (73%) were obtained as a colorless solid, after

purification on column chromatography using *n*-hexane/AcOEt (1:1 → 1:2) as eluent; m.p. 134-137 ºC; $^1$H NMR (700 MHz, acetone-d$_6$, 25ºC): δ 7.42 (1H, dd, *J* = 7.7, 1.4, Hz, $CH_{Ar}$), 7.31 (1H, td, *J* = 7.5, 1.4, Hz, $CH_{Ar}$), 7.27 (1H, dd, *J* = 7.7, 1.5, Hz, $CH_{Ar}$), 7.21 (1H, td, *J* = 7.5, 1.5, Hz, $CH_{Ar}$), 4.26 (2H, d, *J* = 17.0 Hz, $CH_2$), 3.86 (2H, d, *J* = 17.0 Hz, $CH_2$), 3.62 (2H, s, $CH_2$), 3.24 (3H, s, $NCH_3$), 2.46 (3H, s, $CH_3$); $^{13}$C {$^1$H} NMR (176 MHz, acetone-d$_6$, 25ºC): δ 168.0 (C=O), 145.9 (B-C=C), 138.9 ($C_{Ar}$), 132.2 ($C_{Ar}$), 131.6 ($CH_{Ar}$), 130.5 ($CH_{Ar}$), 128.9 ($CH_{Ar}$), 126.1 ($CH_{Ar}$), 120.6 (q, *J* = 330.5 Hz, $CF_3$), 91.2 ($CTf_2$), 62.5 (OC-$CH_2$), 47.8 ($NCH_3$), 35.3 ($CH_2$), 20.0 ($CH_3$); $^{19}$F NMR (282 MHz, acetone-d$_6$, 25ºC): δ –70.5 (3F, s, $CF_3$); HRMS (ESI-TOF) m/z: $[M-H]^-$ calcd for $C_{18}H_{15}BF_6NO_8S_2$: 562.0245; found 562.0248; IR (cm$^{-1}$): ν 1782 (C=O), 1381, 1197, 1102, 1058.

**MIDA boronate cyclobutene 3o**

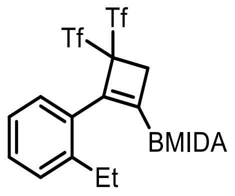


Following the general procedure and starting from 34 mg (0.12 mmol) of alkyne **2o**, 43 mg (0.07 mmol) of compound **3o** (62%) were obtained as a colorless oil, after purification on column chromatography using *n*-hexane/AcOEt (1:3 → 1:5) as eluent; $^1$H NMR (300 MHz, acetone-d$_6$, 25ºC): δ 7.43 (1H, m, $CH_{Ar}$), 7.38 (2H, m, $CH_{Ar}$), 7.21 (1H, m, $CH_{Ar}$), 4.25 (2H, d, *J* = 17.1 Hz, $CH_2$), 3.86 (2H, d, *J* = 17.1 Hz, $CH_2$), 3.61 (2H, s, $CH_2$), 3.23 (3H, s, $NCH_3$), 2.87 (2H, q, *J* = 7.6 Hz, $CH_2$), 1.26 (3H, t, *J* = 7.6 Hz, $CH_3$); $^{13}$C {$^1$H} NMR (75 MHz, acetone-d$_6$, 25ºC): δ 167.9 (C=O), 146.0 (B-C=C), 144.6 ($C_{Ar}$), 131.7 ($C_{Ar}$), 130.7 ($CH_{Ar}$), 129.4 ($CH_{Ar}$), 128.9 ($CH_{Ar}$), 126.0 ($CH_{Ar}$), 91.4 ($CTf_2$), 62.6 (OC-$CH_2$), 47.8 ($NCH_3$), 35.3 ($CH_2$), 25.5 ($CH_2$), 14.6 ($CH_3$); $^{19}$F NMR (282 MHz, acetone-d$_6$, 25ºC): δ –70.4 (3F, s, $CF_3$); HRMS (ESI-TOF) m/z: $[M+Na]^+$ calcd for

$C_{19}H_{18}BF_6NNaO_8S_2$: 600.0363; found 600.0372; IR (cm$^{-1}$): ν 1787 (C=O), 1381, 1196, 1103, 1055.

**MIDA boronate cyclobutene 3p**

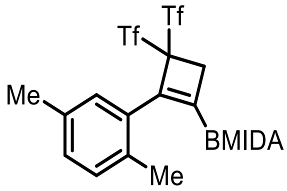


Following the general procedure and starting from 20 mg (0.07 mmol) of alkyne **2p**, 22 mg (0.04 mmol) of compound **3p** (54%) were obtained as a colorless solid, after purification on column chromatography using *n*-hexane/AcOEt (1:1 → 1:2) as eluent; m.p. 152-155 ºC; $^{1}$H NMR (300 MHz, acetone-d$_6$, 25ºC): δ 7.25 (1H, m, CH$_{Ar}$), 7.13 (2H, m, CH$_{Ar}$), 4.26 (2H, d, $J$ = 17.0 Hz, CH$_2$), 3.86 (2H, d, $J$ = 17.0 Hz, CH$_2$), 3.60 (2H, s, CH$_2$), 3.23 (3H, s, NCH$_3$), 2.40 (3H, s, CH$_3$), 2.27 (3H, s, CH$_3$); $^{13}$C {$^{1}$H} NMR (176 MHz, acetone-d$_{6,}$ 25ºC): δ 168.0 (C=O), 146.0 (B-C=C), 135.7 (C$_{Ar}$), 135.4 (C$_{Ar}$), 132.1 (C$_{Ar}$), 131.6 (CH$_{Ar}$), 131.3 (CH$_{Ar}$), 129.3 (CH$_{Ar}$), 120.6 (q, $J$ = 330.3 Hz, CF$_3$), 91.2 (CTf$_2$), 62.5 (OC-CH$_2$), 47.8 (NCH$_3$), 35.2 (CH$_2$), 20.9 (CH$_3$), 19.5 (CH$_3$); $^{19}$F NMR (282 MHz, acetone-d$_6$, 25ºC): δ –70.5 (3F, s, CF$_3$); HRMS (ESI-TOF) m/z: [M-H]$^-$ calcd for $C_{19}H_{17}BF_6NO_8S_2$: 576.0402; found 576.0406; IR (cm$^{-1}$): ν 1794 (C=O), 1382, 1196, 1104, 1058.

**MIDA boronate cyclobutene 3q**

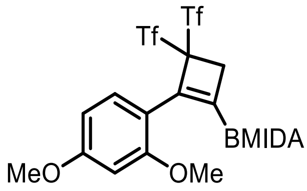

Following the general procedure and starting from 24.4 mg (0.08 mmol) of alkyne **2q**, 29 mg (0.05 mmol) of compound **3q** (62 %) were obtained as a yellow oil, after purification on column chromatography using *n*-hexane/AcOEt (1:4 → 0:1) as eluent; $^{1}$H-RMN (300 MHz, acetone-d$_6$, 25ºC): δ 7.29 (1H, dd, *J* = 8.1, 0.7 Hz, CH$_{Ar}$), 6.56 (1H, d, *J* = 2.5 Hz, CH$_{Ar}$), 6.53 (1H, dd, *J* = 8.2, 2.4 Hz, CH$_{Ar}$), 4.31 (2H, d, *J* = 17.0 Hz, CH$_2$), 3.98 (2H, d, *J* = 17.0 Hz, CH$_2$), 3.83 (3H, s, OCH$_3$), 3.81 (3H, s, OCH$_3$), 3.50 (2H, s, CH$_2$), 3.30 (3H, s, NCH$_3$); $^{13}$C {$^{1}$H} NMR (75 MHz, acetone-d$_6$, 25ºC): δ 168.3 (C=O), 163.4 (C$_{Ar}$), 159.5 (C$_{Ar}$), 144.1 (B-C=<u>C</u>), 132.3 (CH$_{Ar}$), 120.8 (q, *J* = 328.7 Hz, CF$_3$), 114.2 (C$_{Ar}$), 105.7 (CH$_{Ar}$), 99.1 (CH$_{Ar}$), 90.8 (CTf$_2$), 63.2 (OC-<u>C</u>H$_2$), 55.8 (OCH$_3$), 55.7 (OCH$_3$), 48.1 (NCH$_3$), 35.7 (CH$_2$); $^{19}$F NMR (282 MHz, acetone-d$_6$, 25ºC): δ –70.7 (3F, s, CF$_3$); HRMS (ESI-TOF) m/z: [M-H]$^-$ calcd for $C_{19}H_{17}BF_6NO_{10}S_2$: 608.0300; found 608.0305; IR (cm$^{-1}$): ν 1776 (C=O), 1381, 1209, 1104, 1050.

**MIDA boronate cyclobutene 3r**

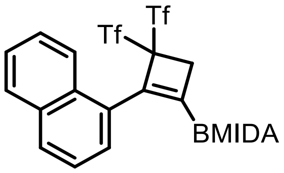


Following the general procedure and starting from 49.8 mg (0.16 mmol) of alkyne **2r**, 53.5 mg (0.09 mmol) of compound **3r** (55%) were obtained as a colorless oil, after purification on column chromatography using *n*-hexane/AcOEt (1:4 → 0:1) as eluent; $^{1}$H-RMN (300 MHz, acetone-d$_6$, 25ºC): δ 8.29 (1H, d, *J* = 7.5 Hz, CH$_{Ar}$), 7.99 (1H, d, *J* = 8.3 Hz, CH$_{Ar}$), 7.93 (1H, dd, *J* = 7.2, 2.3 Hz, CH$_{Ar}$), 7.69 (1H, dd, *J* = 7.3, 1.3 Hz, CH$_{Ar}$), 7.60–7.51 (3H, m, CH$_{Ar}$), 4.19 (2H, d, *J* = 17.1 Hz, CH$_2$), 3.88 (2H, d, *J* = 17.1 Hz, CH$_2$), 3.76 (2H, s, CH$_2$), 3.31 (3H, s, CH$_3$); $^{13}$C {$^{1}$H} NMR (75 MHz, acetone-d$_6$, 25ºC): δ 167.7 (C=O), 145.0 (B-C=<u>C</u>), 134.5 (C$_{Ar}$), 132.2 (C$_{Ar}$), 131.3 (CH$_{Ar}$), 130.6

($C_{Ar}$), 129.1 ($CH_{Ar}$), 127.7 ($CH_{Ar}$), 127.5 ($CH_{Ar}$), 127.3 ($CH_{Ar}$), 126.7 ($CH_{Ar}$), 125.5 ($CH_{Ar}$), 120.6 (q, $J$ = 330.6 Hz, $CF_3$), 91.4 ($CTf_2$), 62.6 (OC-$\underline{C}H_2$), 48.0 ($CH_3$), 35.7 ($CH_2$); $^{19}$F NMR (282 MHz, acetone-$d_6$, 25ºC): δ –70.6 (3F, s, $CF_3$); HRMS (ESI-TOF) m/z: $[M-H]^-$ calcd for $C_{21}H_{15}BF_6NO_8S_2$: 598.0246; found 598.0242; IR ($cm^{-1}$): ν 1737 (C=O), 1338, 1188, 1120, 1040.

**MIDA boronate cyclobutene 3s**

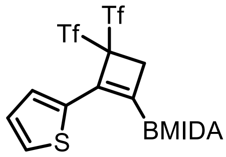


Following the general procedure and starting from 25 mg (0.1 mmol) of alkyne **2s**, 44 mg (0.08 mmol) of compound **3s** (84%) were obtained as a brown solid, after purification on column chromatography using *n*-hexane/AcOEt (1:2 → 1:4) as eluent; m.p. 128-130 ºC; $^1$H NMR (700 MHz, acetone-$d_6$, 25ºC): δ 7.73 (1H, dd, $J$= 5.1, 1.1 Hz, $CH_{Ar}$), 7.61 (1H, dd, $J$= 3.8, 1.1 Hz, $CH_{Ar}$), 7.16 (1H, dd, $J$ = 5.1, 3.8 Hz, $CH_{Ar}$), 4.47 (2H, d, $J$ = 17.2 Hz, $CH_2$), 4.28 (2H, d, $J$ = 17.2 Hz, $CH_2$), 3.60 (2H, s, $CH_2$), 3.24 (3H, s, $NCH_3$); $^{13}$C {$^1$H} NMR (176 MHz, acetone-$d_6$, 25ºC): δ 168.6 (C=O), 138.0 (B-C=$\underline{C}$), 133.3 ($C_{Ar}$), 131.6 ($CH_{Ar}$), 130.9 ($CH_{Ar}$), 128.5 ($CH_{Ar}$), 120.7 (q, $J$ = 331.3 Hz, $\underline{C}F_3$), 87.8 ($CTf_2$), 63.8 (OC-$\underline{C}H_2$), 48.4 ($NCH_3$), 37.2 ($CH_2$); $^{19}$F NMR (282 MHz, acetone-$d_6$, 25ºC): δ –71.5 (3F, s, $CF_3$); HRMS (ESI-TOF) m/z: $[M-H]^-$ calcd for $C_{15}H_{11}BF_6NO_8S_3$: 553.9652; found 553.9662; IR ($cm^{-1}$): ν 1763 (C=O), 1373, 1192, 1104, 1052.

**MIDA boronate cyclobutene 3t**

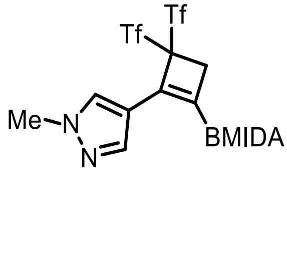

Following the general procedure and starting from 23 mg (0.09 mmol) of alkyne **2t**, 14 mg (0.02 mmol) of compound **3t** (30%) were obtained as a yellow oil, after purification on column chromatography using AcOEt as eluent; $^{1}$H NMR (300 MHz, acetone-d$_6$, 25ºC): δ 7.93 (1H, s, CH$_{Ar}$), 7.82 (1H, s, CH$_{Ar}$), 4.44 (2H, d, *J* = 17.1 Hz, CH$_2$), 4.26 (2H, d, *J* = 17.1 Hz, CH$_2$), 3.92 (3H, s, CH$_3$), 3.53 (2H, s, CH$_2$), 3.22 (3H, s, NCH$_3$); $^{13}$C {$^{1}$H} NMR (75 MHz, acetone-d$_6$, 25ºC): δ 168.6 (C=O), 139.7 (CH$_{Ar}$), 137.0 (B-C=$\underline{C}$), 132.0 (CH$_{Ar}$), 120.8 (q, *J* = 330.7 Hz, $\underline{C}$F$_3$), 114.0 (C$_{Ar}$), 87.8 (CTf$_2$), 63.3 (OC-$\underline{C}$H$_2$), 47.9 (NCH$_3$), 39.3, 37.0 (CH$_2$); $^{19}$F NMR (282 MHz, acetone-d$_6$, 25ºC): δ –71.6 (3F, s, CF$_3$); HRMS (ESI-TOF) m/z: [M+H]$^+$ calcd for $C_{15}H_{15}BF_6N_3O_8S_2$: 554.0295; found 554.0282; IR (cm$^{-1}$): ν = 1769 (C=O), 1377, 1191, 1100, 1046.

**MIDA boronate cyclobutene 3u**

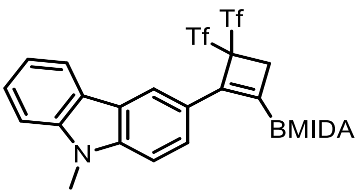


Following the general procedure and starting from 31.4 mg (0.09 mmol) of alkyne **2u**, 40.2 mg (0.06 mmol) of compound **3u** (71%) were obtained as a yellow solid, after purification on column chromatography using *n*-hexane/AcOEt (1:3) as eluent; m.p. 138-140 ºC; $^{1}$H NMR (300 MHz, acetone-d$_6$, 25ºC): δ 8.54 (1H, dd, *J* = 1.8, 0.6 Hz, CH$_{Ar}$), 8.15 (1H, ddd, *J* = 7.8, 1.2, 0.8 Hz, CH$_{Ar}$), 7.85 (1H, dd, *J* = 8.7, 1.9 Hz, CH$_{Ar}$), 7.61 (1H, ddd, *J* = 3.6, 0.8, Hz, CH$_{Ar}$), 7.59 (1H, d, *J* = 0.6 Hz, CH$_{Ar}$), 7.55 (1H, m, CH$_{Ar}$), 7.27 (1H, ddd, *J* = 8.0, 6.9, 1.2 Hz, CH$_{Ar}$), 4.42 (2H, d, *J* = 17.2 Hz, CH$_2$), 4.14 (2H, d, *J* = 17.1 Hz, CH$_2$), 3.96 (3H, s, NCH$_3$), 3.61 (2H, s, CH$_2$), 3.20 (3H, s, NCH$_3$); $^{13}$C {$^{1}$H} NMR (75 MHz, acetone-d$_6$, 25ºC): δ 168.6 (C=O), 146.9 (B-C=$\underline{C}$), 142.6 (C$_{Ar}$), 142.4 (C$_{Ar}$), 127.4 (CH$_{Ar}$), 126.5 (CH$_{Ar}$), 123.5 (C$_{Ar}$), 123.4 (C$_{Ar}$), 121.4 (CH$_{Ar}$), 121.0 (CH$_{Ar}$), 120.5

($CH_{Ar}$), 118.7 ($C_{Ar}$), 110.1 ($CH_{Ar}$), 109.8 ($CH_{Ar}$), 89.1 ($CTf_2$), 63.5 (OC-$\underline{C}H_2$), 48.2 ($NCH_3$), 36.2 ($CH_2$), 29.5 ($NCH_3$); $^{19}$F NMR (282 MHz, acetone-d$_6$, 25ºC): δ –71.0 (3F, s, $CF_3$); HRMS (ESI-TOF) m/z: $[M+Na]^+$ calcd for $C_{24}H_{19}BF_6N_2NaO_8S_2$: 675.0472; found 675.0482; IR (cm$^{-1}$): ν 1769 (C=O), 1283, 1150, 1071, 1024.

**MIDA boronate cyclobutene 3v**

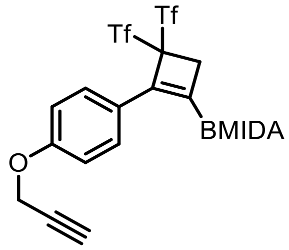


Following the general procedure and starting from 50 mg (0.15 mmol) of alkyne **2v**, 51 mg (0.09 mmol) of compound **3v** (51%) were obtained as a yellow oil, after purification on column chromatography using *n*-hexane/AcOEt (1:3) as eluent; $^1$H NMR (300 MHz, acetone-d$_6$, 25ºC): δ 7.66 (2H, d, $J$ = 8.9 Hz, $CH_{Ar}$), 7.00 (2H, d, $J$ = 8.9 Hz, $CH_{Ar}$), 4.86 (2H, d, $J$ = 2.4, $CH_2$), 4.39 (2H, d, $J$ = 17.2 Hz, $CH_2$), 4.15 (2H, d, $J$ = 17.2 Hz, $CH_2$), 3.56 (2H, s, $CH_2$), 3.21 (3H, s, $NCH_3$), 3.12 (1H, t, $J$ = 2.4 Hz, C≡C$\underline{H}$); $^{13}$C {$^1$H} NMR (75 MHz, acetone-d$_6$, 25ºC): δ 168.4 (C=O), 159.8 ($C_{Ar}$), 145.7 (B-C=$\underline{C}$), 130.6 ($CH_{Ar}$), 125.2 ($C_{Ar}$), 115.6 ($CH_{Ar}$), 89.0 ($CTf_2$), 79.3 (C≡CH), 77.4 (C≡CH), 63.3 (OC-$\underline{C}H_2$), 56.4 ($CH_2$), 48.0 ($NCH_3$), 36.1 ($CH_2$); $^{19}$F NMR (282 MHz, acetone-d$_6$, 25ºC): δ –71.0 (3F, s, $CF_3$); HRMS (ESI-TOF) m/z: $[M-H]^-$ calcd for $C_{20}H_{15}BF_6NO_9S_2$: 602.0195; found 602.0197.

**MIDA boronate cyclobutene 3a-*d*$_2$**

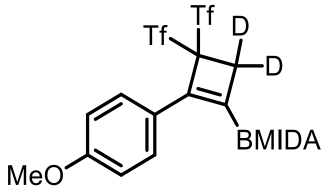


Following the general procedure and starting from 30 mg (0.1 mmol) of alkyne **2a**, 46.5 mg (0.08 mmol) of compound **3a**-*d₂* (77%) were obtained as a yellow solid, after purification on column chromatography using *n*-hexane/AcOEt (1:1 → 1:3) as eluent; m.p. 117-120 ºC; $^{1}$H-RMN (300 MHz, acetone-$d_6$, 25ºC): δ 7.63 (2H, d, *J* = 8.9 Hz, $CH_{Ar}$), 6.98 (2H, d, *J* = 8.9 Hz, $CH_{Ar}$), 4.39 (2H, d, *J* = 17.2 Hz, $CH_2$), 4.12 (2H, d, *J* = 17.2 Hz, $CH_2$), 3.85 (3H, s, $OCH_3$), 3.19 (3H, s, $NCH_3$); $^{13}$C {$^{1}$H} NMR (75 MHz, acetone-$d_6$, 25ºC): δ 168.4 (C=O), 161.9 ($C_{Ar}$), 146.0 (B-C=<u>C</u>), 130.6 ($CH_{Ar}$), 124.5 ($C_{Ar}$), 120.8 (q, *J* = 330.9 Hz, $CF_3$), 114.8 ($CH_{Ar}$), 88.9 ($CTf_2$), 79.2 ($CD_2$), 63.3 (OC-<u>C</u>$H_2$), 55.7 ($OCH_3$), 48.0 ($NCH_3$); $^{19}$F NMR (282 MHz, acetone-$d_6$, 25ºC): δ –71.0 (3F, s, $CF_3$); HRMS (ESI-TOF) m/z: $[M+Na]^+$ calcd for $C_{18}H_{14}D_2BF_6NNaO_9S_2$: 604.0281; found 604.0273; IR ($cm^{-1}$): ν 1750 (C=O), 1342, 1176, 1097, 1039.

**MIDA boronate cyclobutene 3n-*d₂***

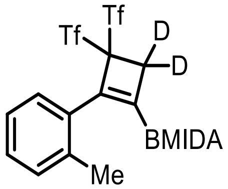


Following the general procedure and starting from 25 mg (0.09 mmol) of alkyne **2n**, 40 mg (0.07 mmol) of compound **3n**-*d₂* (77%) were obtained as a beige solid, after purification on column chromatography using *n*-hexane/AcOEt (1:1 → 1:5) as eluent; m.p. 146-149 ºC; $^{1}$H NMR (300 MHz, acetone-$d_6$, 25ºC): δ 7.42 (1H, m, $CH_{Ar}$), 7.29 (2H, m, $CH_{Ar}$), 7.21 (1H, m, $CH_{Ar}$), 4.26 (2H, d, *J* = 17.0 Hz, $CH_2$), 3.86 (2H, d, *J* = 17.0 Hz, $CH_2$), 3.23 (3H, s, $NCH_3$), 2.46 (3H, s, $CH_3$); $^{13}$C {$^{1}$H} NMR (75 MHz, acetone, 25ºC) δ

168.0 (C=O), 146.1 (B-C=C), 138.9 ($C_{Ar}$), 132.2 ($C_{Ar}$), 131.7 ($CH_{Ar}$), 130.6 ($CH_{Ar}$), 129.0 ($CH_{Ar}$), 128.1 ($C_{Ar}$), 126.2 ($CH_{Ar}$), 120.7 (q, $J$ = 330.4 Hz, $CF_3$), 91.1 ($CTf_2$), 62.6 (OC-$CH_2$), 47.8 ($NCH_3$), 20.0 ($CH_3$); $^{19}$F NMR (282 MHz, acetone-$d_6$, 25ºC): δ –70.5 (3F, s, $CF_3$); HRMS (ESI-TOF) m/z: $[M+H]^+$ calcd for $C_{18}H_{15}D_2BF_6NO_8S_2$: 566.0516; found 566.0524; IR ($cm^{-1}$): ν 1789 (C=O), 1383, 1197, 1106, 1055.

**MIDA boronate cyclobutene 3p-*d₂***

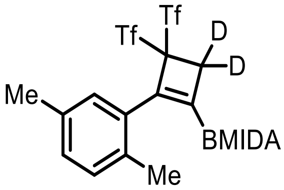


Following the general procedure and starting from 23 mg (0.08 mmol) of alkyne **2p**, 36.2 mg (0.06 mmol) of compound **3p**-$d_2$ (77%) were obtained as a colorless solid, after purification on column chromatography using *n*-hexane/AcOEt (1:1 → 1:5) as eluent; m.p. 160-163 ºC; $^1$H NMR (300 MHz, acetone-$d_6$, 25ºC): δ 7.25 (1H, m, $CH_{Ar}$), 7.13 (2H, m, $CH_{Ar}$), 4.25 (2H, d, $J$ = 17.0 Hz, $CH_2$), 3.86 (2H, d, $J$ = 17.0 Hz, $CH_2$), 3.23 (3H, s, $NCH_3$), 2.40 (3H, s, $CH_3$), 2.27 (3H, s, $CH_3$); $^{13}$C {$^1$H} NMR (75 MHz, acetone-$d_6$, 25ºC): δ 168.0 (C=O), 146.2 (B-C=C), 135.7 ($C_{Ar}$), 135.5 ($C_{Ar}$), 132.1 ($C_{Ar}$), 131.6 ($CH_{Ar}$), 131.3 ($CH_{Ar}$), 129.3 ($CH_{Ar}$), 120.7 (q, $J$ = 330.3 Hz, $CF_3$), 91.1 ($CTf_2$), 62.6 (OC-$CH_2$), 47.8 ($NCH_3$), 20.9 ($CH_3$), 19.5 ($CH_3$); $^{19}$F NMR (282 MHz, acetone-$d_6$, 25ºC): δ –70.5 (3F, s, $CF_3$); HRMS (ESI-TOF) m/z: $[M+Na]^+$ calcd for $C_{19}H_{16}D_2BF_6NO_8S_2Na$: 602.0492; found 602.0482; IR ($cm^{-1}$): ν 1773 (C=O), 1337, 1190, 1107, 1052.

**MIDA boronate cyclobutene 3q-*d₂***

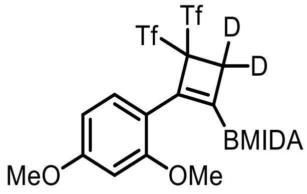


Following the general procedure and starting from 32.9 mg (0.1 mmol) of alkyne **2q**, 41.3 mg (0.07 mmol) of compound **3q**-*d2* (65%) were obtained as a yellow oil, after purification on column chromatography using *n*-hexane/AcOEt (1:4 → 0:1) as eluent; $^{1}$H-RMN (300 MHz, acetone-$d_6$, 25ºC): δ 7.29 (1H, dd, *J* = 8.2, 0.6 Hz, $CH_{Ar}$), 6.56 (1H, d, *J* = 2.2 Hz, $CH_{Ar}$), 6.54 (1H, dd, *J* = 8.2, 2.3 Hz, $CH_{Ar}$), 4.31 (2H, d, *J* = 17.1 Hz, $CH_2$), 3.99 (2H, d, *J* = 17.0 Hz, $CH_2$), 3.83 (3H, s, $OCH_3$), 3.81 (3H, s, $OCH_3$), 3.30 (3H, s, $NCH_3$); $^{13}$C {$^{1}$H} NMR (75 MHz, acetone-$d_6$, 25ºC): δ 168.3 (C=O), 163.4 ($C_{Ar}$), 159.5 ($C_{Ar}$), 144.2 (B-C=<u>C</u>), 132.3 ($CH_{Ar}$), 120.8 (q, *J* = 330.9 Hz, $CF_3$), 114.2 ($C_{Ar}$), 105.7 ($CH_{Ar}$), 99.1 ($CH_{Ar}$), 63.2 (OC-<u>C</u>H2), 55.8 ($OCH_3$), 55.7 ($OCH_3$), 48.1 ($NCH_3$); $^{19}$F NMR (282 MHz, acetone-$d_6$, 25ºC): δ –70.7 (3F, s, $CF_3$); HRMS (ESI-TOF) m/z: $[M+Na]^+$ calcd for $C_{19}H_{16}D_2BF_6NNaO_{10}S_2$: 634.0391; found 634.0384; IR ($cm^{-1}$): ν 1768 (C=O), 1382, 1186, 1104, 1046.

**General procedure for the synthesis of BMIDA-naphthalenes 4.**

*Method A:* The corresponding cyclobutene **3** (1 equiv.), toluene (27.9 mL/mmol) and acetonitrile (5.6 ml/mmol) were added to an oven-dried microwave equipped with a stir bar. The reaction was stirred at room temperature under argon atmosphere for 5 minutes and heated at 140ºC for 20 minutes using microwave irradiation, until completed consumption of **3** (as monitored by TLC). The reaction mixture was cooled to room temperature and was concentrated under reduced pressure. Chromatography of the residue eluting with *n*-hexane/ethyl acetate mixtures gave analytically pure compounds **4**.

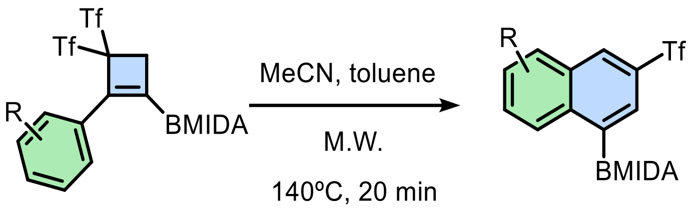


**3a-s** **4a-s**

**Scheme S4.** Synthesis of BMIDA naphthalenes **4**.

*Method B (one-pot):* The corresponding BMIDA-alkyne **2** (1 equiv.), zwitterion **1** or **1-***$d_2$* (1.05 equiv.) and acetonitrile (5.6 ml/mmol) were added to an oven-dried microwave and the solution was stirred at room temperature until disappearance of the starting alkyne **2** as monitored by TLC. Next, toluene (27.9 mL/mmol) was added and the reaction mixture was heated at 140ºC for 20 minutes using microwave irradiation. The mixture was cooled to room temperature and was concentrated under reduced pressure. Chromatography of the residue eluting with *n*-hexane/ethyl acetate mixtures gave analytically pure compounds. Spectroscopic and analytical data for triflyl-decorated BMIDA-naphthalenes **4** follow.

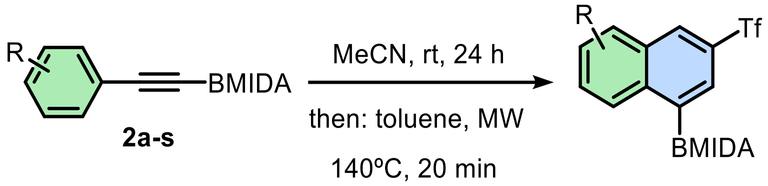


**2a-s** **4a-s**

**Scheme S5.** One-pot synthesis of BMIDA naphthalenes **4**.

**MIDA boronate naphthalene 4a**

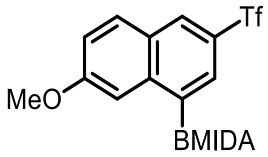


Following the general procedure and starting from 11 mg (0.04 mmol) of alkyne **2a**, 8.6 mg (0.02 mmol) of compound **4a** (45%) were obtained as an orange oil, after purification on column chromatography using *n*-hexane/AcOEt (1:2 → 1:4) as eluent; $^1$H-RMN (300

MHz, acetone-$d_6$, 25ºC): δ 8.30 (1H, d, *J* = 8.6 Hz, $CH_{Ar}$), 7.29 (1H, s, $CH_{Ar}$), 7.02 (1H, s, $CH_{Ar}$), 6.93 (1H, d, *J* = 2.5 Hz, $CH_{Ar}$), 6.79 (1H, dd, *J* = 8.7, 2.5 Hz, $CH_{Ar}$), 4.39 (2H, d, *J* = 17.1 Hz, $CH_2$), 4.25 (2H, d, *J* = 17.1 Hz, $CH_2$), 3.89 (3H, s, $OCH_3$), 3.11 (s, 3H, $NCH_3$); $^{13}$C {$^1$H} NMR (176 MHz, acetone-$d_6$, 25ºC): δ 168.8 (C=O), 164.6 ($C_{Ar}$), 162.8 ($C_{Ar}$), 151.3 ($CH_{Ar}$), 148.9 ($C_{Ar}$), 131.4 ($CH_{Ar}$), 125.9 ($C_{Ar}$), 121.2 (q, *J* = 326.4 Hz, $CF_3$), 116.0 ($CH_{Ar}$), 111.8 ($CH_{Ar}$), 110.8 ($CH_{Ar}$), 62.2 (OC-$\underline{C}H_2$), 56.2 (O$\underline{C}H_3$), 47.5 (N$\underline{C}H_3$); $^{19}$F NMR (282 MHz, acetone-$d_6$, 25ºC): δ –79.7 (3F, s, $CF_3$); HRMS (ESI-TOF) m/z: $[M+Na]^+$ calcd for $C_{17}H_{15}BF_3NNaO_7S$: 468.0510; found 468.0509; IR ($cm^{-1}$): ν 1772 (C=O), 1572, 1338, 1211, 1118, 1027.

**MIDA boronate naphthalene 4a-*d***

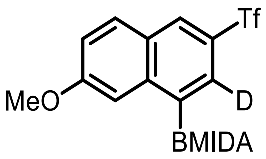


Following the general procedure and starting from 11 mg (0.04 mmol) of alkyne **2a**, 8.7 mg of compound **4a-*d*** (51 %) were obtained as an orange oil, after purification on column chromatography using *n*-hexane/AcOEt (1:2 → 2:5) as eluent; $^1$H-RMN (700 MHz, acetone-$d_6$, 25ºC): δ 8.30 (1H, d, *J* = 8.6 Hz, $CH_{Ar}$), 7.02 (1H, d, *J* = 1.1 Hz, $CH_{Ar}$), 6.93 (1H, d, *J* = 2.5 Hz, $CH_{Ar}$), 6.79 (1H, dd, *J* = 8.7, 2.5 Hz, $CH_{Ar}$), 4.38 (2H, d, *J* = 17.1 Hz, $CH_2$), 4.24 (2H, d, *J* = 17.1 Hz, $CH_2$), 3.89 (3H, s, $OCH_3$), 3.11 (s, 3H, $NCH_3$); $^{13}$C {$^1$H} NMR (176 MHz, acetone-$d_6$, 25ºC): δ 168.8 (C=O), 164.6 ($C_{Ar}$), 162.8 ($C_{Ar}$), 148.9 ($C_{Ar}$), 131.4 ($CH_{Ar}$), 125.9 ($C_{Ar}$), 121.2 (q, *J* = 326.4 Hz, $CF_3$), 116.0 ($CH_{Ar}$), 111.8 ($CH_{Ar}$), 110.8 ($CH_{Ar}$), 62.2 (OC-$\underline{C}H_2$), 56.2 ($OCH_3$), 47.5 ($NCH_3$); $^{19}$F NMR (282 MHz, acetone-$d_6$, 25ºC): δ –79.7 (3F, s, $CF_3$); HRMS (ESI-TOF) m/z: $[M+Na]^+$ calcd for

$C_{17}H_{14}DBF_3NNaO_7S$: 469.0569; found 469.0578; IR (cm$^{-1}$): ν 1771 (C=O), 1567, 1347, 1202, 1116.

**MIDA boronate naphthalene 4b**

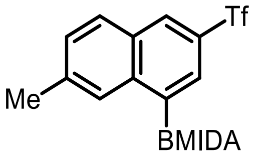


Following the general procedure and starting from 11 mg (0.04 mmol) of alkyne **2b**, 6.9 mg (0.02 mmol) of compound **4b** (46%) were obtained as a yellow oil, after purification on column chromatography using *n*-hexane/AcOEt (1:1 → 1:2) as eluent; $^1$H-RMN (700 MHz, acetone-d$_6$, 25ºC): δ 8.23 (1H, d, *J* = 7.9 Hz, $CH_{Ar}$), 7.33 (1H, s, $CH_{Ar}$), 7.17 (1H, d, *J* = 1.7 Hz, $CH_{Ar}$), 7.13 (1H, s, $CH_{Ar}$), 7.11 (1H, dd, *J* = 7.8, 1.7 Hz, $CH_{Ar}$), 4.38(2H, d, *J* = 17.0 Hz, $CH_2$), 4.25 (2H, d, *J* = 17.1 Hz, $CH_2$), 3.11 (s, 3H, $NCH_3$), 2.36 (3H, s, $CH_3$); $^{13}$C {$^1$H} NMR (176 MHz, acetone-d$_6$, 25ºC): δ 168.8 (C=O), 163.1 ($C_{Ar}$), 152.3 ($CH_{Ar}$), 146.7 ($C_{Ar}$), 144.3 ($C_{Ar}$), 131.3 ($C_{Ar}$), 129.5 ($CH_{Ar}$), 129.0 ($CH_{Ar}$), 124.3 ($CH_{Ar}$), 121.1 (q, *J* = 325.8 Hz, $CF_3$), 117.7 ($CH_{Ar}$), 62.2 (OC-$\underline{C}H_2$), 47.5 (O$\underline{C}H_3$), 21.6 ($\underline{C}H_3$); $^{19}$F NMR (282 MHz, acetone-d$_6$, 25ºC): δ –79.7 (3F, s, $CF_3$); HRMS (ESI-TOF) m/z: $[M+Na]^+$ calcd for $C_{17}H_{15}BF_3NO_6SNa$: 452.0561; found 452.0558; IR (cm$^{-1}$): ν 1771 (C=O), 1261, 1029.

**MIDA boronate naphthalene 4e**

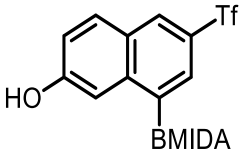


Following the general procedure and starting from 8 mg (0.03 mmol) of alkyne **2e**, 4.0 mg (0.01 mmol) of compound **4e** (31%) were obtained as a yellow oil, after purification

on column chromatography using *n*-hexane/AcOEt (1:2 → 1:3) as eluent; $^{1}$H-RMN (700 MHz, acetone-d$_6$, 25ºC): δ 9.36 (1H, s, O<u>H</u>), 8.21 (1H, d, *J* = 8.4 Hz, CH$_{Ar}$), 7.25 (1H, s, CH$_{Ar}$), 6.97 (1H, s, CH$_{Ar}$), 6.82 (1H, d, *J* = 2.3 Hz, CH$_{Ar}$), 6.67 (1H, d, *J* = 8.6, 2.5 Hz, CH$_{Ar}$), 4.38 (2H, d, *J* = 17.1 Hz, CH$_2$), 4.25 (2H, d, *J* = 17.1 Hz, CH$_2$), 3.10 (s, 3H, NCH$_3$); $^{13}$C {$^{1}$H} NMR (176 MHz, acetone-d$_6$, 25ºC): δ 168.8 (C=O), 163.0 (C$_{Ar}$), 162.8 (C$_{Ar}$), 151.3 (CH$_{Ar}$), 149.3 (C$_{Ar}$), 131.7 (CH$_{Ar}$), 124.9 (C$_{Ar}$), 120.4 (q, *J* = 327.2 Hz, CF$_3$), 115.3 (CH$_{Ar}$), 113.8 (CH$_{Ar}$), 111.7 (CH$_{Ar}$), 62.2 (OC-<u>C</u>H$_2$), 47.5 (N<u>C</u>H$_3$); $^{19}$F NMR (282 MHz, acetone-d$_6$, 25ºC): δ –79.8 (3F, s, CF$_3$); HRMS (ESI-TOF) m/z: [M-H]$^-$ calcd for C$_{16}$H$_{12}$BF$_3$NO$_7$S: 430.0388; found 430.0385; IR (cm$^{-1}$): ν 1771 (C=O), 1261, 1095, 1029.

**MIDA boronate naphthalene 4f**

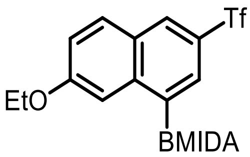


Following the general procedure and starting from 12 mg (0.04 mmol) of alkyne **2f**, 12.4 mg (0.03 mmol) of compound **4f** (68%) were obtained as an orange oil, after purification on column chromatography using *n*-hexane/AcOEt (1:2) as eluent; $^{1}$H-RMN (300 MHz, acetone-d$_6$, 25ºC): δ 8.28 (1H, d, *J* = 8.6 Hz, CH$_{Ar}$), 7.29 (1H, d, *J* = 0.9 Hz, CH$_{Ar}$), 7.01 (1H, d, *J* = 0.9 Hz, CH$_{Ar}$), 6.92 (1H, d, *J* = 2.5 Hz, CH$_{Ar}$), 6.78 (1H, dd, *J* = 8.7, 2.5 Hz, CH$_{Ar}$), 4.38 (2H, d, *J* = 17.1 Hz, CH$_2$), 4.24 (2H, d, *J* = 17.1 Hz, CH$_2$), 4.15 (2H, q, *J* = 7.0 Hz, CH$_2$), 3.11 (s, 3H, NCH$_3$), 1.39 (3H, t, *J* = 7.0 Hz, CH$_3$); $^{13}$C {$^{1}$H} NMR (75 MHz, acetone-d$_6$, 25ºC): δ 168.8 (C=O), 164.0 (C$_{Ar}$), 162.8 (C$_{Ar}$), 151.4 (CH$_{Ar}$), 149.0 (C$_{Ar}$), 131.4 (CH$_{Ar}$), 125.7 (C$_{Ar}$), 120.3 (q, *J* = 326.4 Hz, CF$_3$), 115.9 (CH$_{Ar}$), 112.4 (CH$_{Ar}$), 111.1 (CH$_{Ar}$), 64.8 (CH$_2$), 62.3 (OC-<u>C</u>H$_2$), 47.6 (NCH$_3$), 14.9 (CH$_3$); $^{19}$F NMR (282 MHz, acetone-d$_6$, 25ºC): δ –79.7 (3F, s, CF$_3$); HRMS (ESI-TOF) m/z: [M+Na]$^+$ calcd for

$C_{18}H_{17}BF_3NNaO_7S$: 482.0666; found 482.0662; IR ($cm^{-1}$): ν 1780 (C=O), 1572, 1340, 1210, 1117.

**MIDA boronate naphthalene 4h**

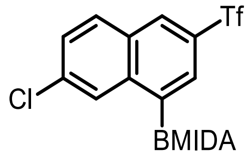


Following the general procedure and starting from 11.6 mg (0.04 mmol) of alkyne **2h**, 7.5 mg (0.02 mmol) of compound **4h** (39%) were obtained as a yellow oil, after purification on column chromatography using *n*-hexane/AcOEt (1:3 → 1:5) as eluent; $^1$H-RMN (700 MHz, acetone-d$_6$, 25ºC): δ 8.33 (1H, d, *J* = 8.3 Hz, $CH_{Ar}$), 7.42 (1H, d, *J* = 2.1 Hz, $CH_{Ar}$), 7.40 (1H, s, $CH_{Ar}$), 7.36 (1H, dd, *J* = 8.3, 2.1 Hz, $CH_{Ar}$), 7.28 (1H, s, $CH_{Ar}$), 4.42 (2H, d, *J* = 17.1 Hz, $CH_2$), 4.28 (2H, d, *J* = 17.1 Hz, $CH_2$), 3.15 (s, 3H, $NCH_3$); $^{13}$C {$^1$H} NMR (176 MHz, acetone-d$_6$, 25ºC): δ 168.8 (C=O), 161.4 ($C_{Ar}$), 150.9 ($CH_{Ar}$), 148.3 ($C_{Ar}$), 138.8 ($C_{Ar}$), 132.4 ($C_{Ar}$), 130.4 ($CH_{Ar}$), 128.1 ($CH_{Ar}$), 123.5 ($CH_{Ar}$), 121.0 (q, *J* = 326.4 Hz, $CF_3$), 119.7 ($CH_{Ar}$), 62.3 (OC-$\underline{C}H_2$), 47.6 ($NCH_3$); $^{19}$F NMR (282 MHz, acetone-d$_6$, 25ºC): δ –79.6 (3F, s, $CF_3$); HRMS (ESI-TOF) m/z: $[M+Na]^+$ calcd for $C_{16}H_{12}BClF_3NNaO_6S$: 472.0011; found 472.0003; IR ($cm^{-1}$): ν 1771 (C=O), 1334, 1284, 1212, 1117, 1046.

**MIDA boronate naphthalene 4k**

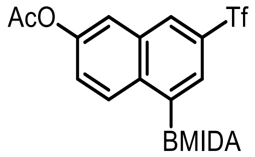


Following the general procedure and starting from 6.3 mg (0.02 mmol) of alkyne **2k**, 6.9 mg (0.01 mmol) of compound **4k** (63%) were obtained as an orange oil, after purification

on column chromatography using *n*-hexane/AcOEt (1:2 → 1:4) as eluent; $^{1}$H-RMN (700 MHz, acetone-$d_6$, 25ºC): δ 7.40 (1H, s, $CH_{Ar}$), 7.37 (1H, d, *J* = 8.0 Hz, $CH_{Ar}$), 7.26 (1H, s, $CH_{Ar}$), 7.20 (1H, dd, *J* = 8.0, 2.1 Hz, $CH_{Ar}$), 4.39 (2H, d, *J* = 17.1 Hz, $CH_2$), 4.26 (2H, d, *J* = 17.1 Hz, $CH_2$), 3.14 (s, 3H, $NCH_3$), 2.29 (s, 3H, $CH_3$); $^{13}$C {$^{1}$H} NMR (176 MHz, acetone-$d_6$, 25ºC): δ 169.8 (C=O), 168.8 (C=O), 162.3 ($C_{Ar}$), 151.9 ($CH_{Ar}$), 151.7 ($C_{Ar}$), 143.6 ($C_{Ar}$), 135.1 ($C_{Ar}$), 126.3 ($CH_{Ar}$), 123.9 ($CH_{Ar}$), 123.5 ($CH_{Ar}$), 121.0 (q, *J* = 326.4 Hz, $CF_3$), 119.5 ($CH_{Ar}$), 62.2 (OC-$\underline{C}H_2$), 47.5 ($NCH_3$), 20.9 (OC$\underline{C}H_3$); $^{19}$F NMR (282 MHz, acetone-$d_6$, 25ºC): δ –79.6 (3F, s, $CF_3$); HRMS (ESI-TOF) m/z: $[M+Na]^+$ calcd for $C_{18}H_{15}BF_3NNaO_8S$: 496.0456; found 496.0449; IR ($cm^{-1}$): ν 1752 (C=O), 1369, 1213, 1118.

**MIDA boronate naphthalene 4l**

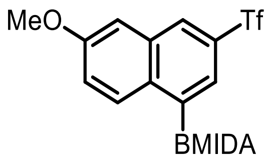


Following the general procedure and starting from 11.5 mg (0.04 mmol) of alkyne **2l**, 8.9 mg (0.02 mmol) of compound **4l** (55%) were obtained as a red oil, after purification on column chromatography using *n*-hexane/AcOEt (1:2) as eluent; $^{1}$H-RMN (700 MHz, acetone-$d_6$, 25ºC): δ 8.06 (1H, d, *J* = 2.3 Hz, $CH_{Ar}$), 7.32 (1H, s, $CH_{Ar}$), 7.24 (1H, d, *J* = 8.1 Hz, $CH_{Ar}$), 7.16 (1H, s, $CH_{Ar}$), 6.95 (1H, dd, *J* = 8.2, 2.4 Hz, $CH_{Ar}$), 4.37 (2H, d, *J* = 17.1 Hz, $CH_2$), 4.24 (2H, d, *J* = 17.1 Hz, $CH_2$), 3.83 (3H, s, $OCH_3$), 3.11 (s, 3H, $NCH_3$); $^{13}$C {$^{1}$H} NMR (176 MHz, acetone-$d_6$, 25ºC): δ 168.8 (C=O), 163.5 ($C_{Ar}$), 161.2 ($C_{Ar}$), 153.0 ($CH_{Ar}$), 138.6 ($C_{Ar}$), 135.9 ($C_{Ar}$), 123.9 ($CH_{Ar}$), 121.1 (q, *J* = 326.4 Hz, $CF_3$), 118.3 ($CH_{Ar}$), 118.3 ($CH_{Ar}$), 115.9 ($CH_{Ar}$), 62.2 (OC-$\underline{C}H_2$), 56.1 ($OCH_3$), 47.5 ($NCH_3$); $^{19}$F NMR (282 MHz, acetone-$d_6$, 25ºC): δ –79.6 (3F, s, $CF_3$); HRMS (ESI-TOF) m/z:

$[M+Na]^+$ calcd for $C_{17}H_{15}BF_3NNaO_7S$: 468.0510; found 468.0515; IR ($cm^{-1}$): ν 1772 (C=O), 1366, 1262, 1212, 1117, 1034.

**MIDA boronate benzothiophene 4s**

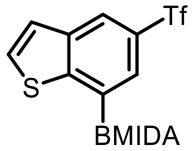


Following the general procedure and starting from 10.5 mg (0.04 mmol) of alkyne **2s**, 7.3 mg (0.03 mmol) of compound **4s** (48%) were obtained as a red oil, after purification on column chromatography using *n*-hexane/AcOEt (1:2) as eluent; $^1$H-RMN (300 MHz, acetone-$d_6$, 25ºC): δ 7.84 (1H, dd, *J* = 4.9, 0.5 Hz, $CH_{Ar}$), 7.17 (1H, d, *J* = 0.7 Hz, $CH_{Ar}$), 7.03 (1H, s, $CH_{Ar}$), 7.01 (1H, d, *J* = 4.9 Hz, $CH_{Ar}$), 4.35 (2H, d, *J* = 17.0 Hz, $CH_2$), 4.22 (2H, d, *J* = 17.0 Hz, $CH_2$), 3.16 (s, 3H, $NCH_3$); $^{13}$C {$^1$H} NMR (176 MHz, acetone-$d_6$, 25ºC): δ 168.9 (C=O), 158.0 ($C_{Ar}$), 157.4 ($C_{Ar}$), 145.7 ($CH_{Ar}$), 140.1 ($CH_{Ar}$), 132.7 ($C_{Ar}$), 132.3 ($C_{Ar}$), 121.3 ($CH_{Ar}$), 121.0 (q, *J* = 326.4 Hz, $CF_3$), 115.0 ($CH_{Ar}$), 62.2 (OC-$\underline{C}H_2$), 47.6 ($NCH_3$); $^{19}$F NMR (282 MHz, acetone-$d_6$, 25ºC): δ –80.0 (3F, s, $CF_3$); HRMS (ESI-TOF) m/z: $[M+H]^+$ calcd for $C_{14}H_{12}BF_3NO_6S_2$: 422.0148; found 422.0152; IR ($cm^{-1}$): ν 1770 (C=O), 1580, 1334, 1212, 1195, 1160, 1117.

# Radical trapping experiment with TEMPO

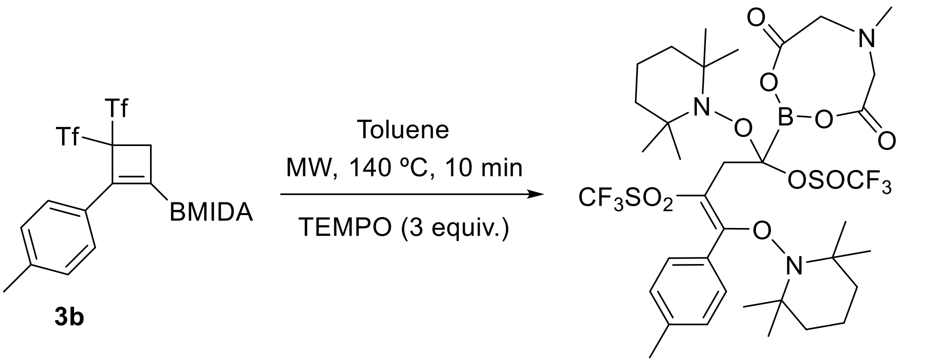


m/z: 875.31

Found: 898.30 $[M + Na]^+$

Determined by LC-MS

trapping of **B** with TEMPO

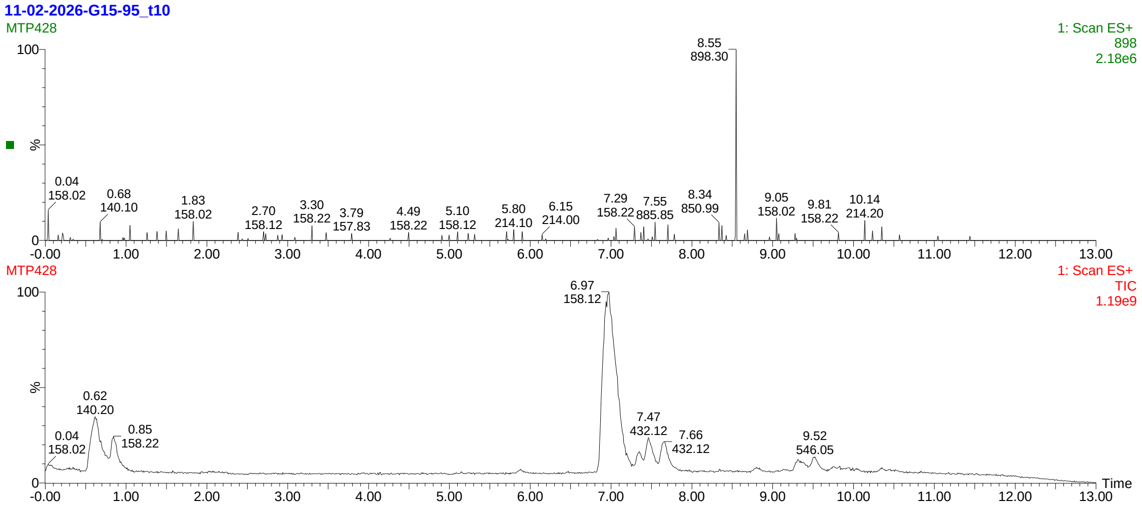


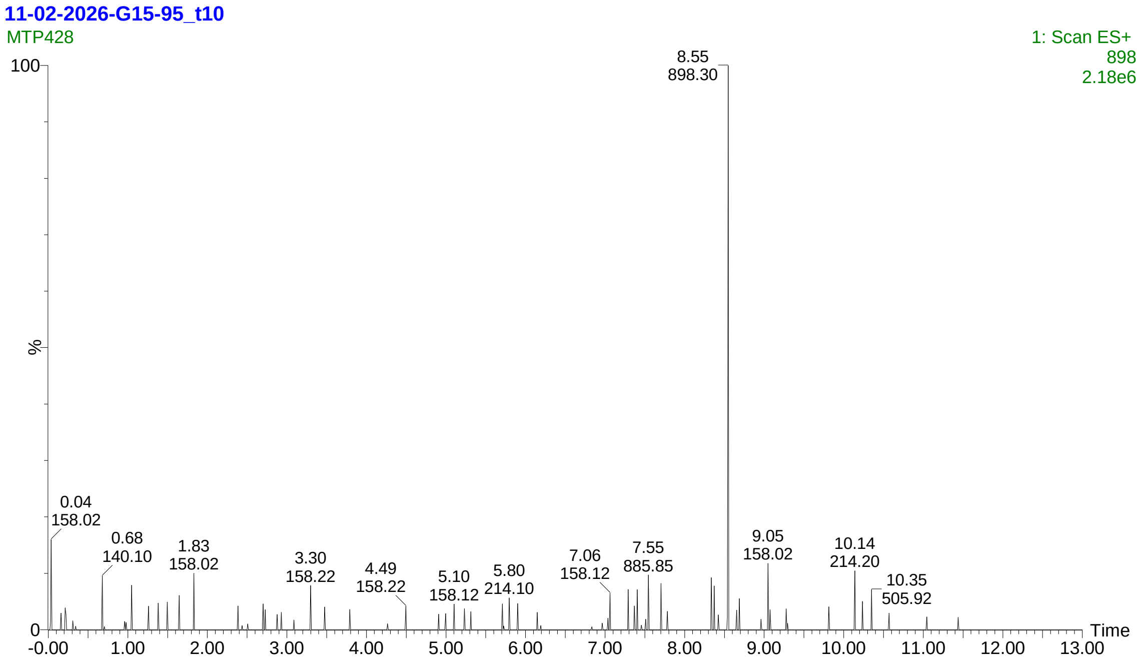

$^{1}$H NMR of compound **2c** (300 MHz, acetone-$d_6$, 25ºC)

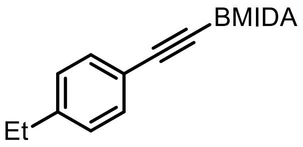


**2c**

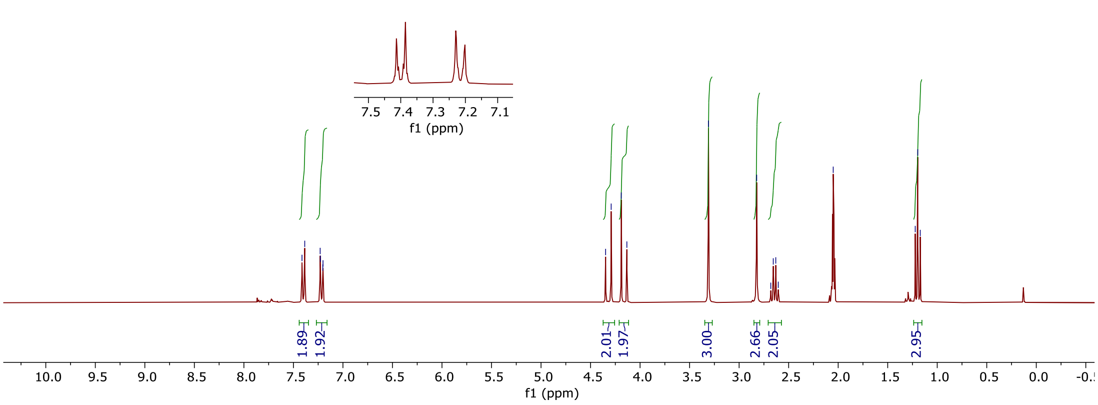


$^{13}$C NMR of compound **2c** (75 MHz, acetone-$d_6$, 25ºC)

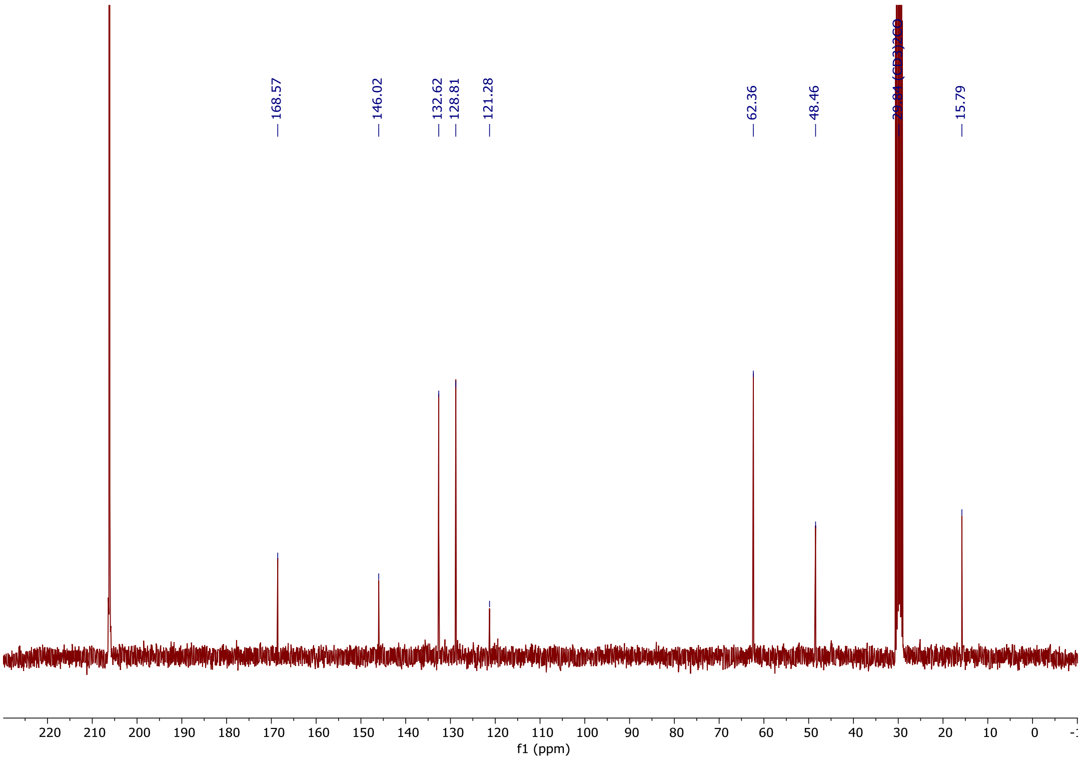

$^{1}$H NMR of compound **2d** (300 MHz, acetone-$d_6$, 25ºC)

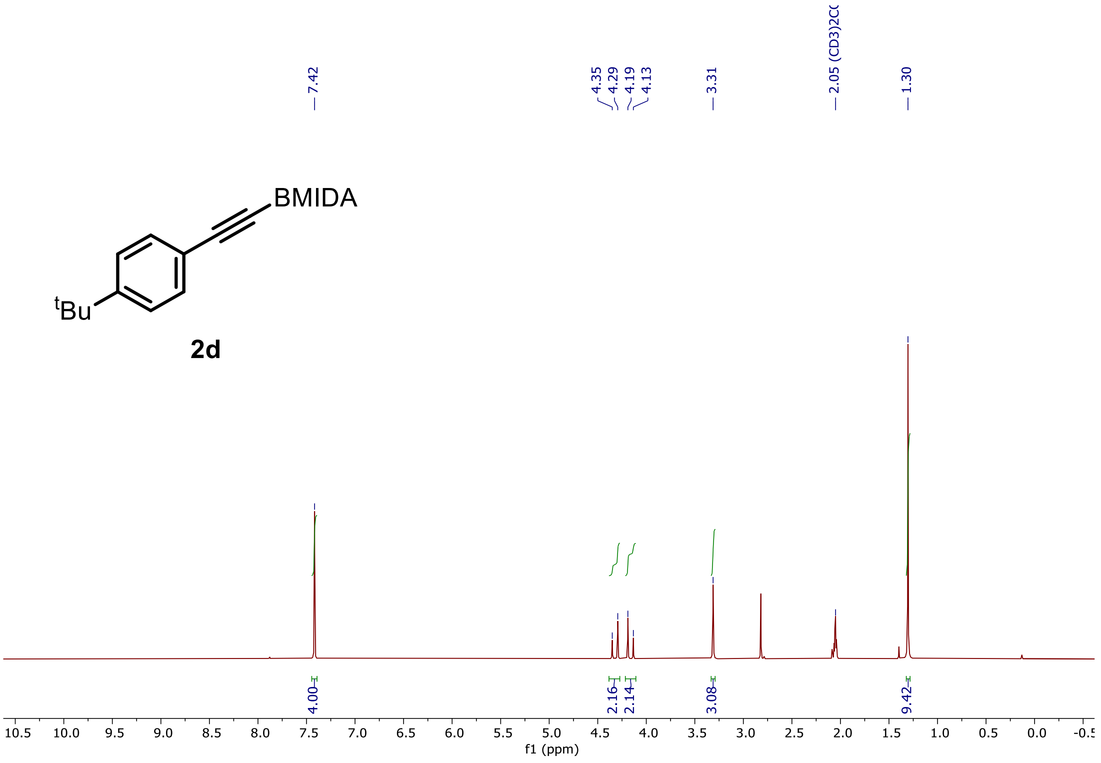


$^{13}$C NMR of compound **2d** (75 MHz, acetone-$d_6$, 25ºC)

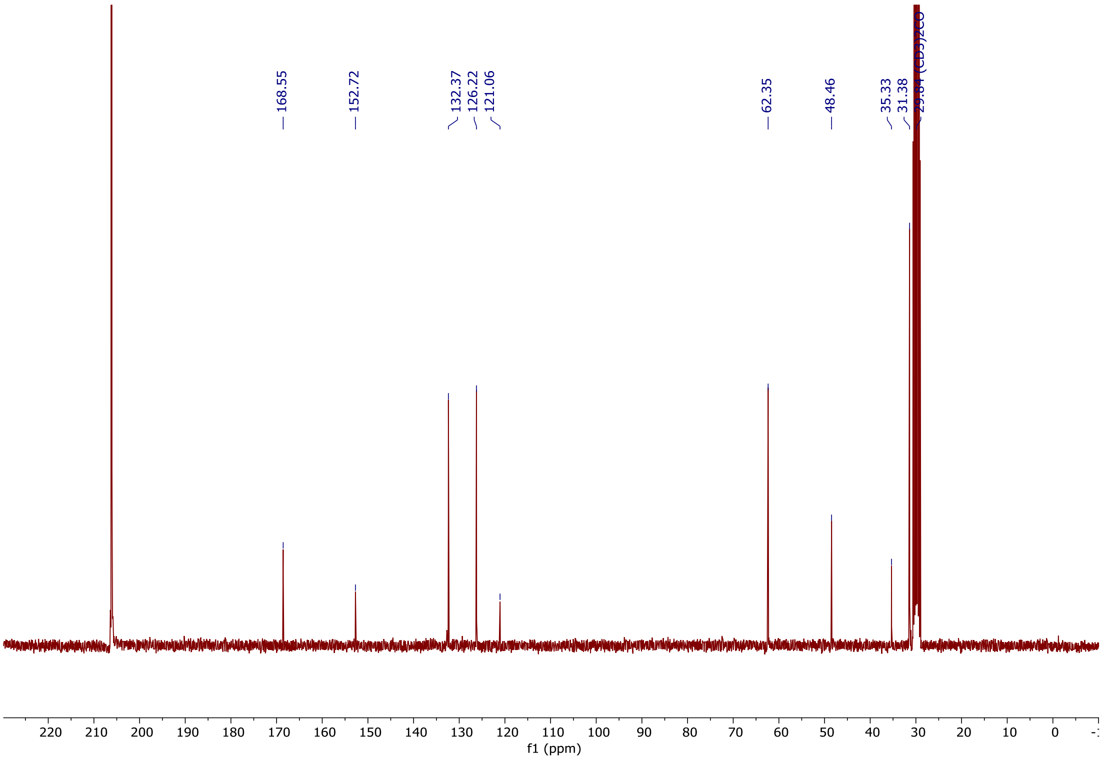

$^{1}$H NMR of compound **2e** (300 MHz, acetone-$d_6$, 25ºC)

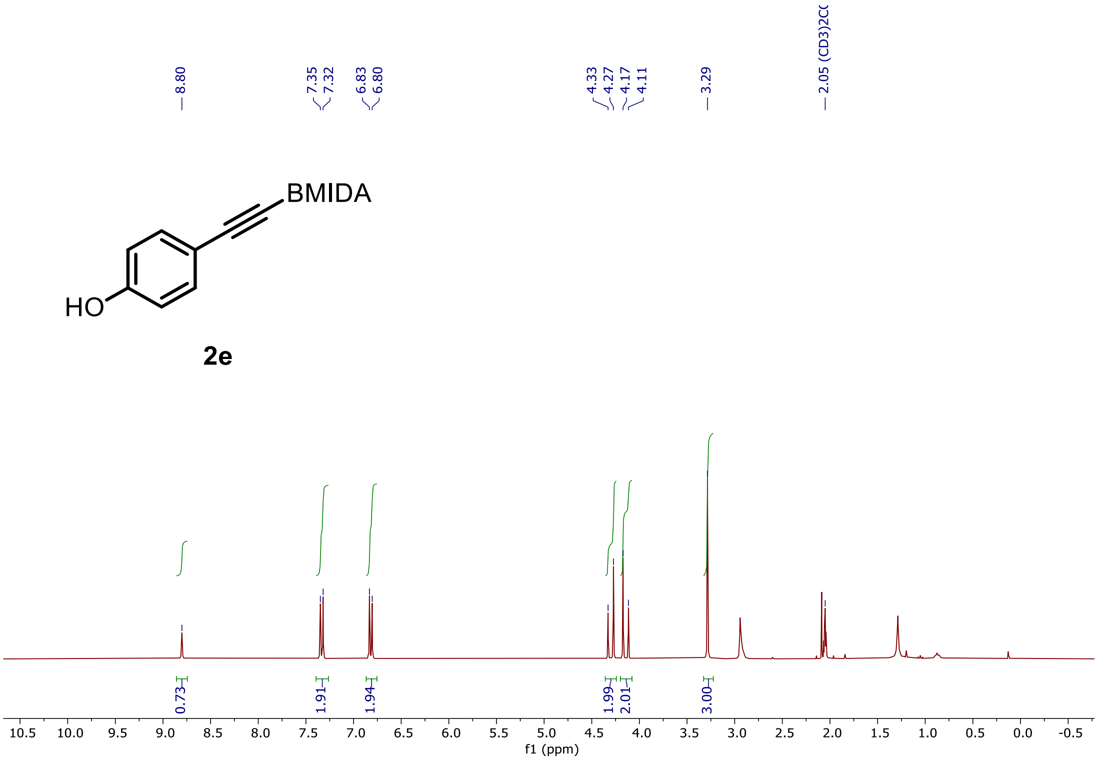


$^{13}$C NMR of compound **2e** (75 MHz, acetone-$d_6$, 25ºC)

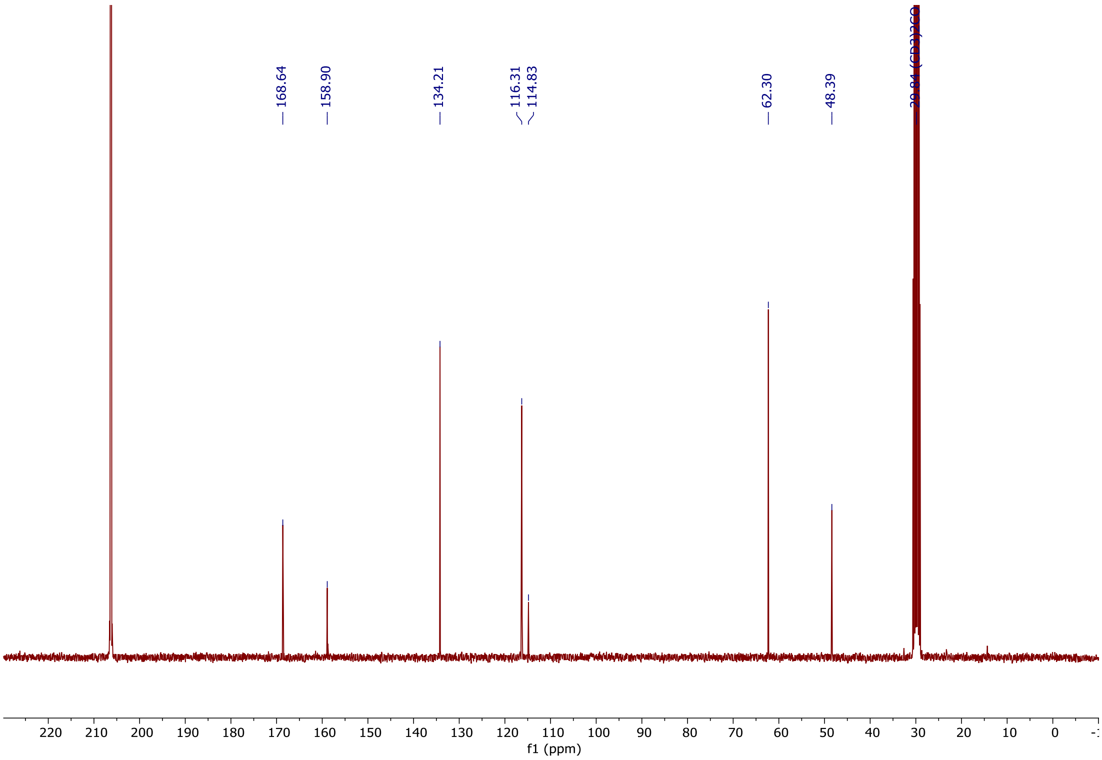

$^{1}$H NMR of compound **2f** (300 MHz, acetone-$d_6$, 25ºC)

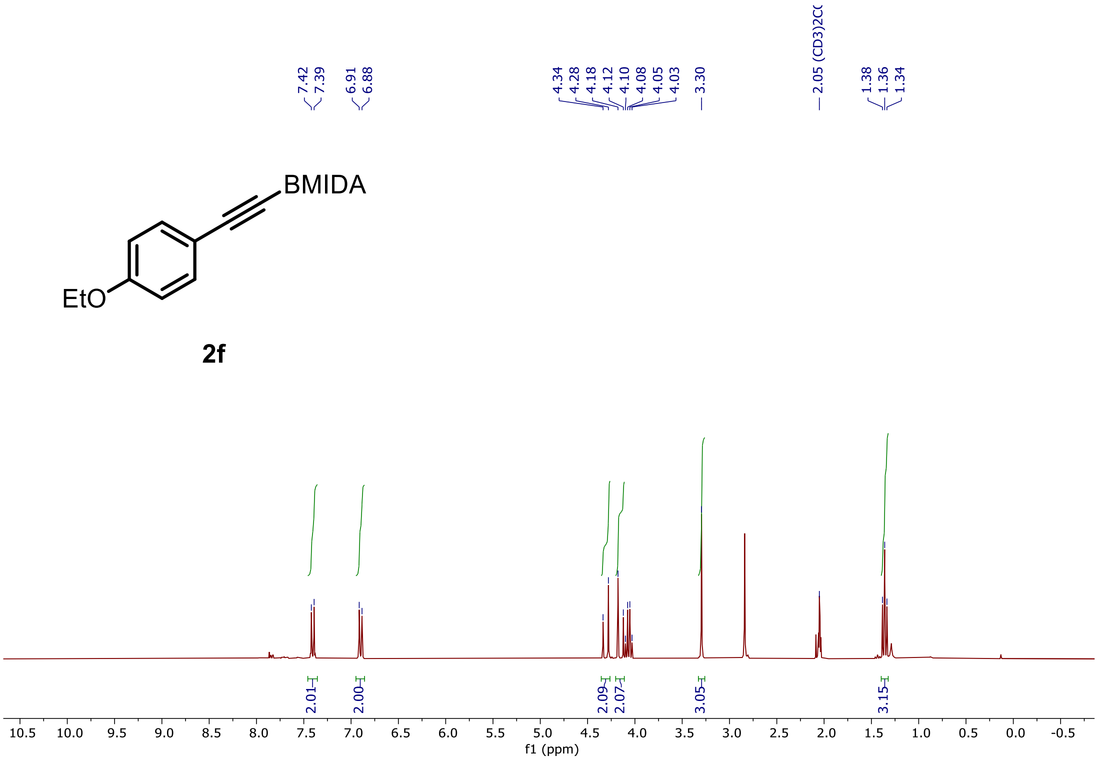


**2f**

$^{13}$C NMR of compound **2f** (75 MHz, acetone-$d_6$, 25ºC)

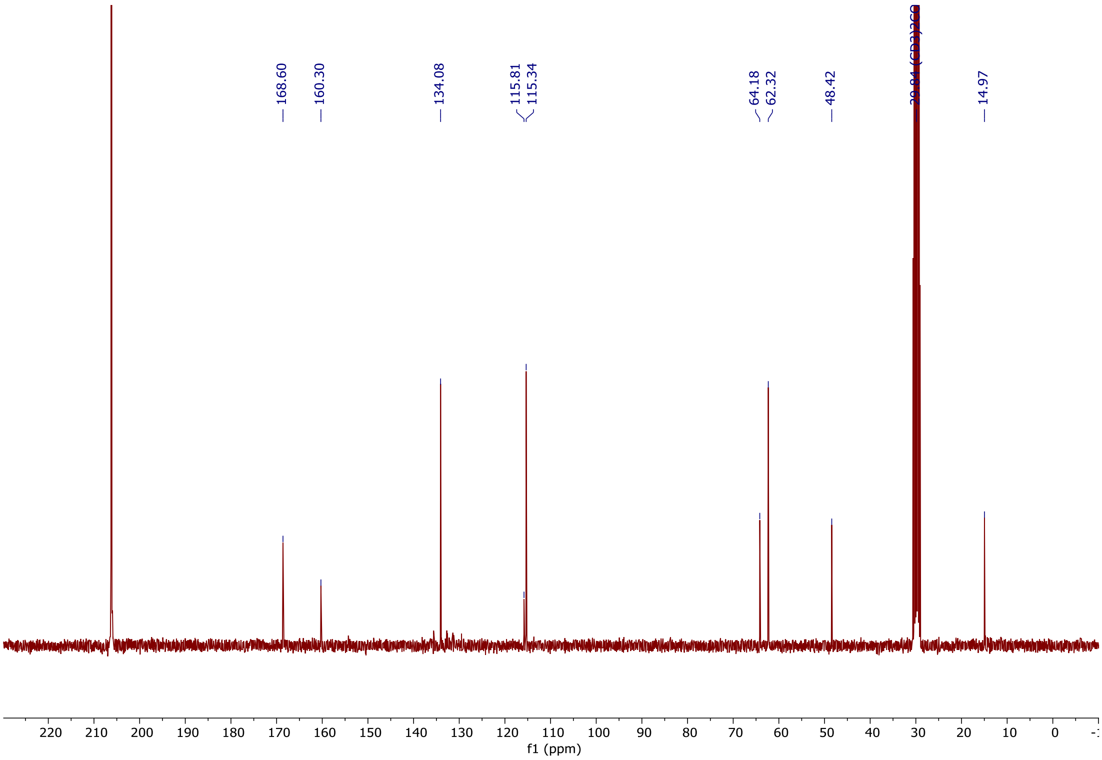

$^{1}H$ NMR of compound **2g** (300 MHz, acetone-$d_6$, 25ºC)

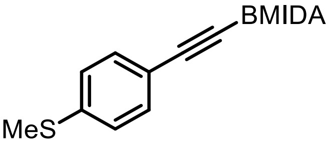


**2g**

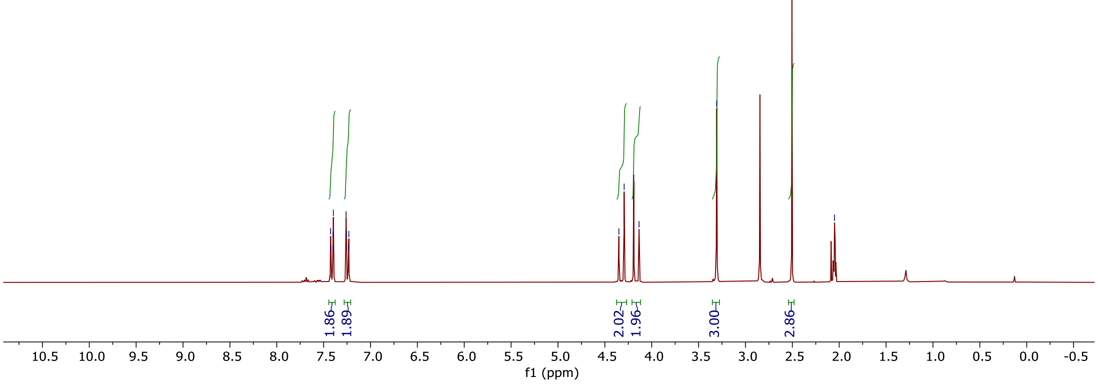


$^{13}C$ NMR of compound **2g** (75 MHz, acetone-$d_6$, 25ºC)

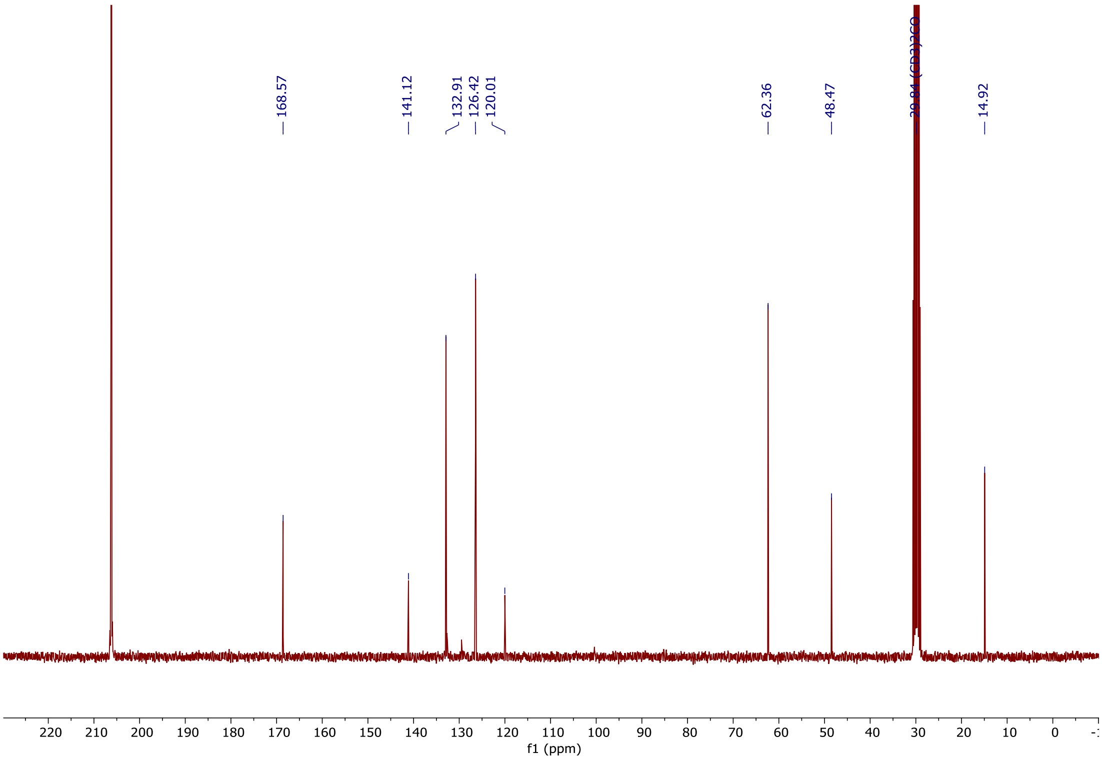

$^{1}H$ NMR of compound **2i** (300 MHz, DMSO-d$_6$, 25ºC)

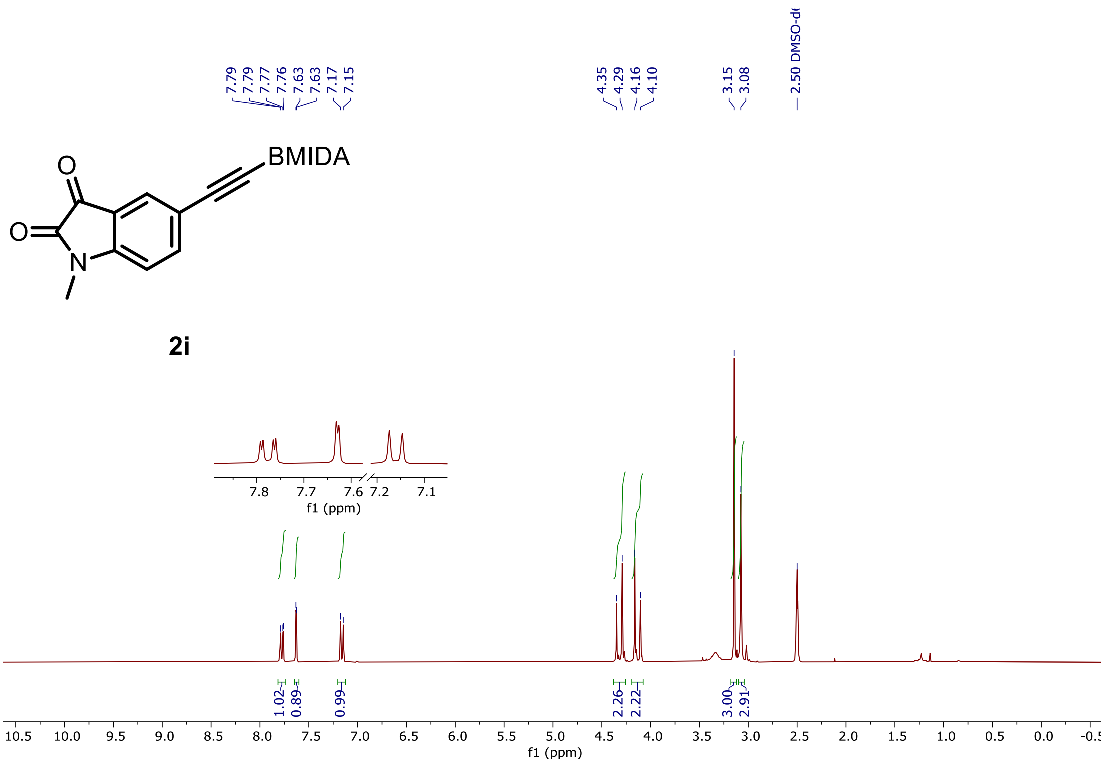

$^{13}C$ NMR of compound **2i** (75 MHz, DMSO-d$_6$, 25ºC)

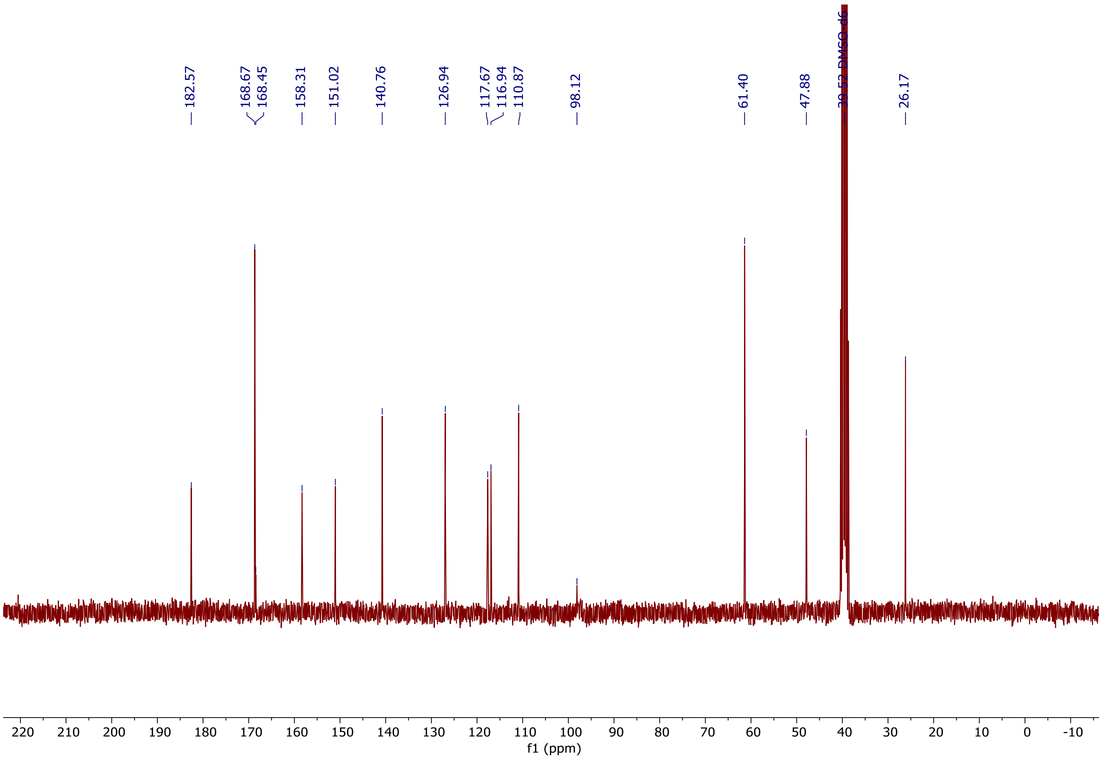

$^{1}$H NMR of compound **2k** (300 MHz, acetone-$d_6$, 25ºC)

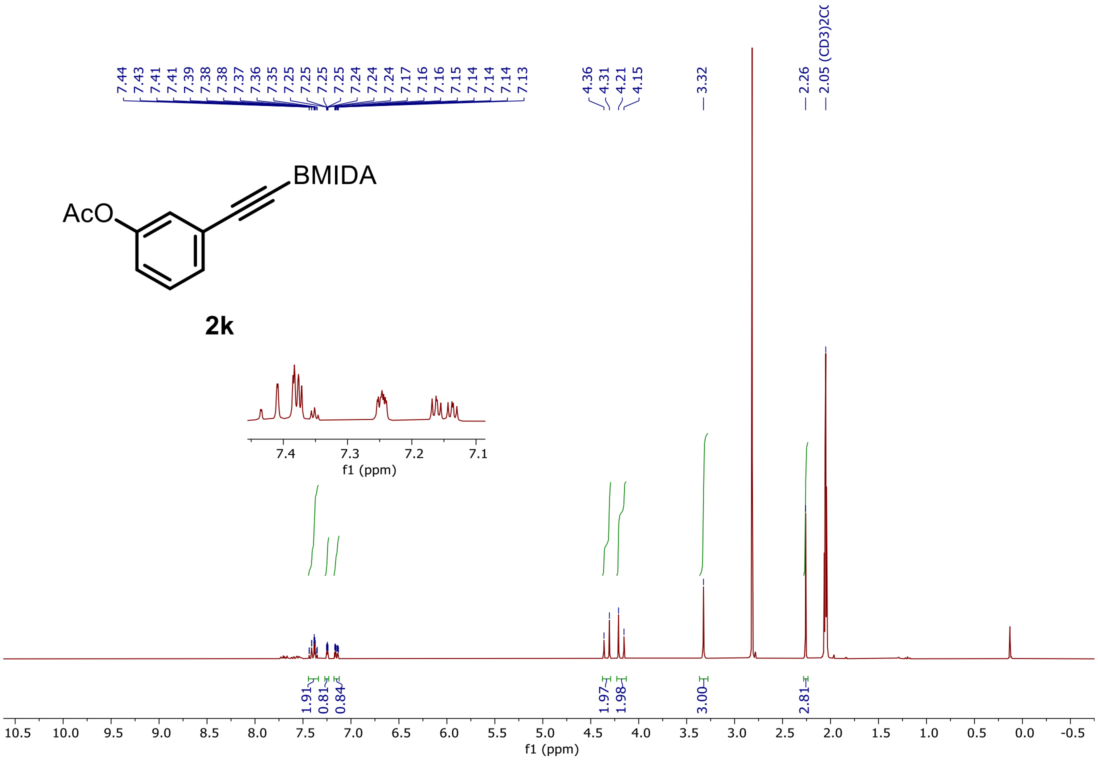


$^{13}$C NMR of compound **2k** (75 MHz, acetone-$d_6$, 25ºC)

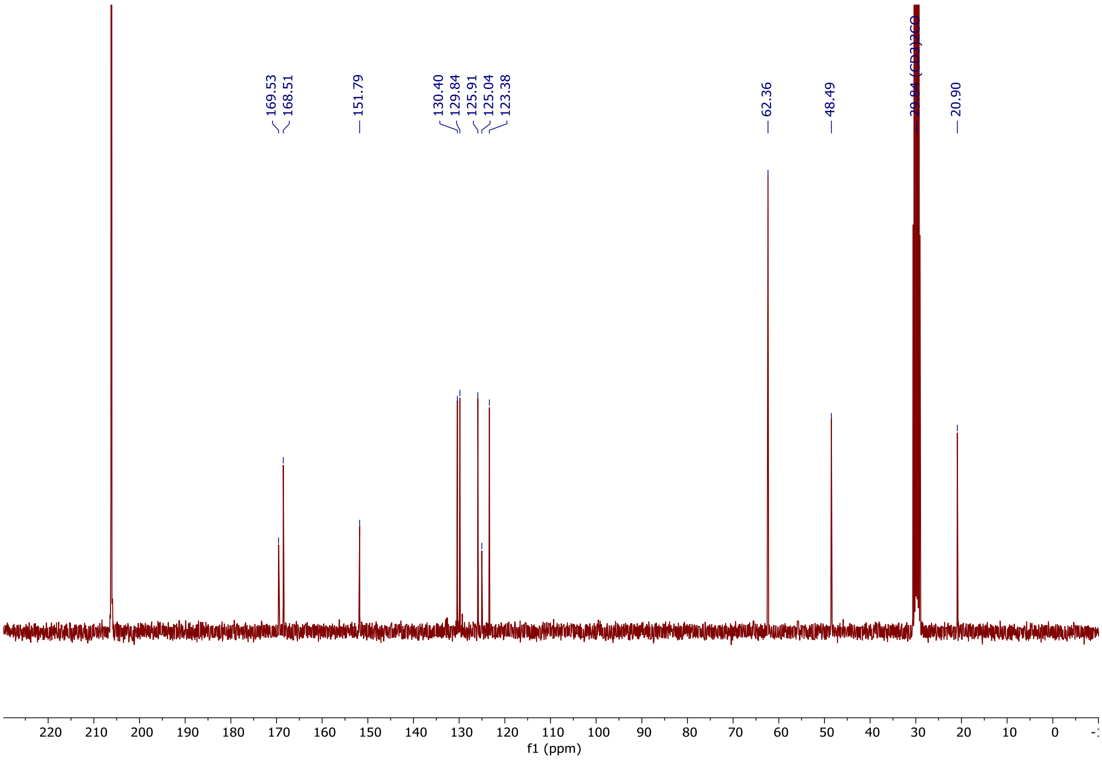

$^{1}$H NMR of compound **2m** (300 MHz, acetone-$d_6$, 25ºC)

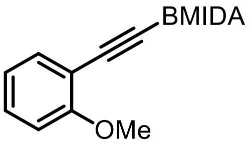


**2m**

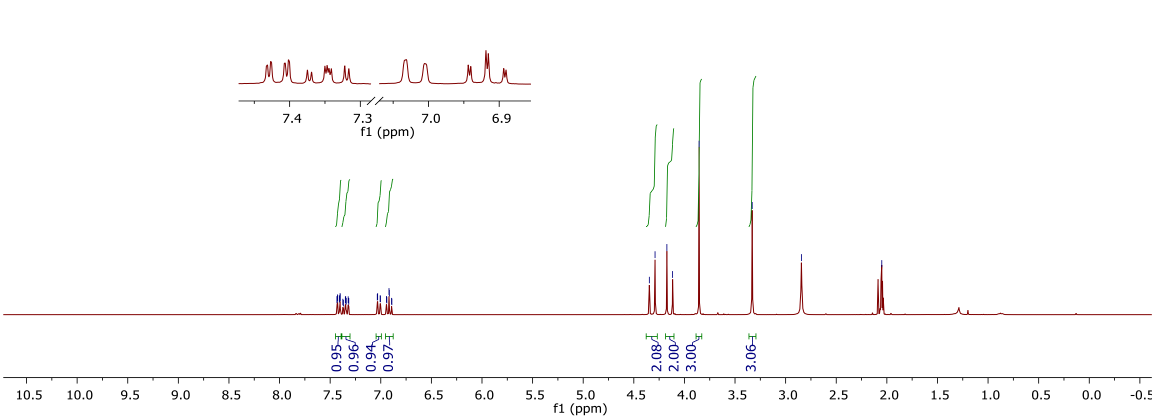


$^{13}$C NMR of compound **2m** (75 MHz, acetone-$d_6$, 25ºC)

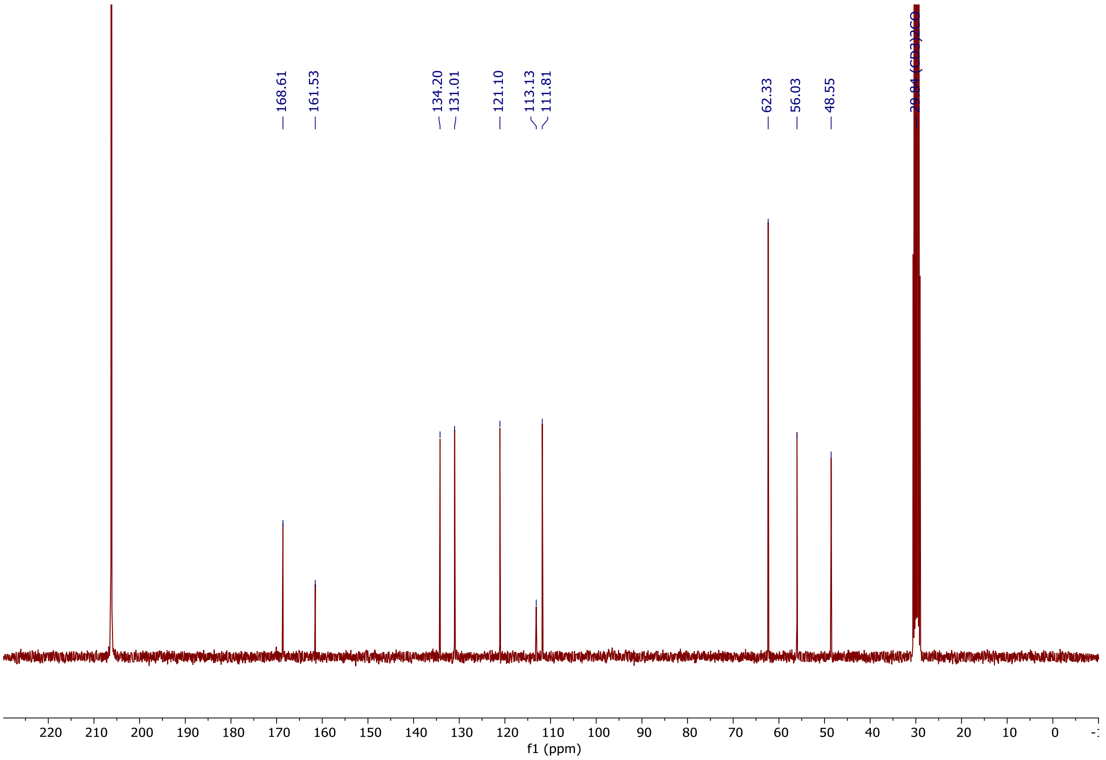

$^{1}$H NMR of compound **2o** (300 MHz, acetone-d$_6$, 25ºC)

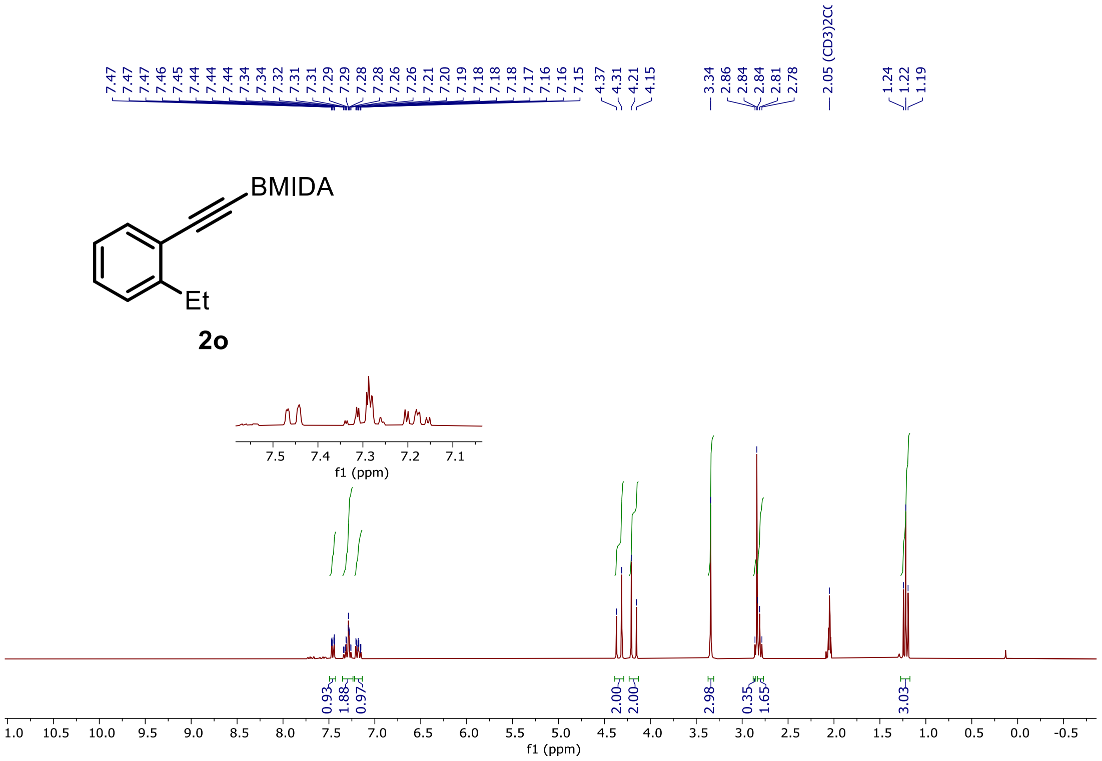


$^{13}$C NMR of compound **2o** (75 MHz, acetone-d$_6$, 25ºC)

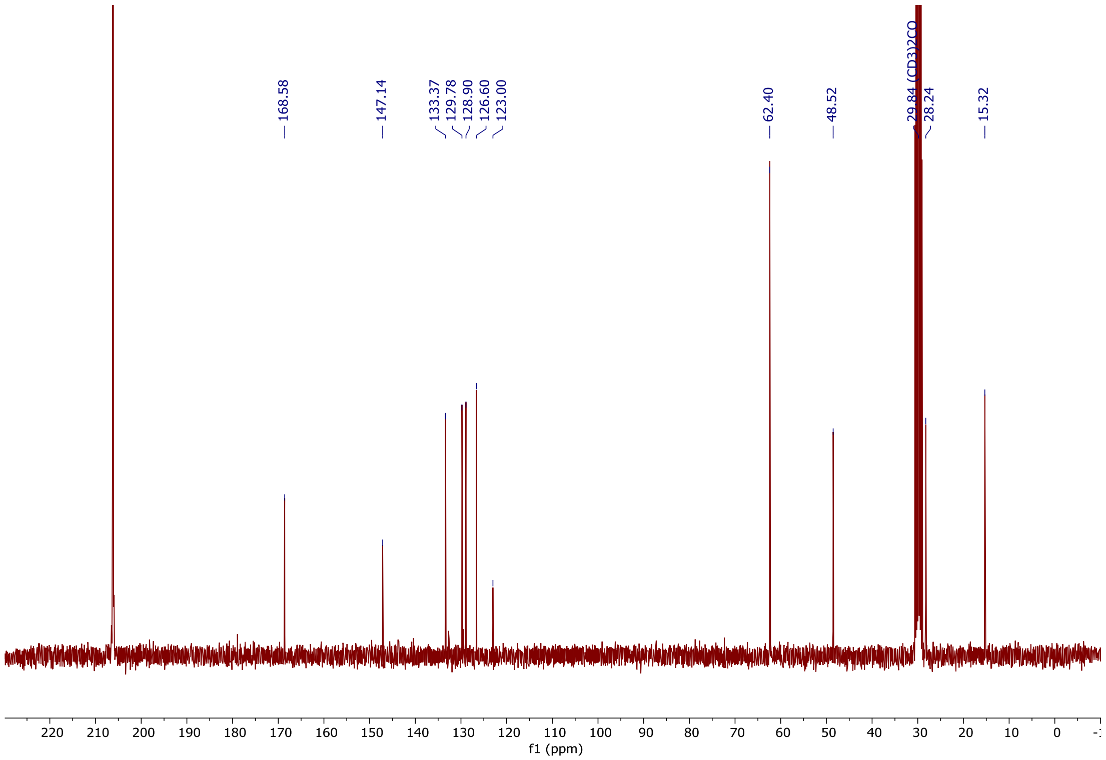

$^{1}$H NMR of compound **2p** (300 MHz, acetone-d$_6$, 25ºC)

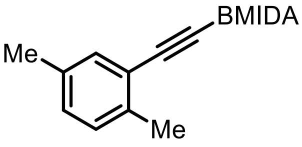


**2p**

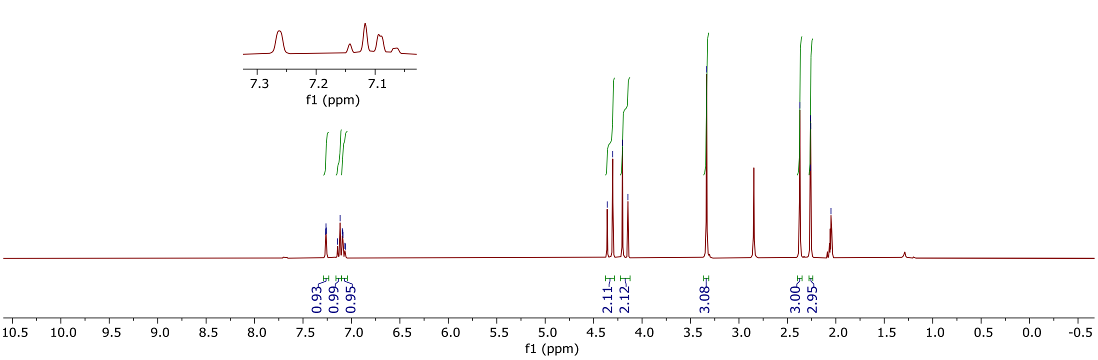


$^{13}$C NMR of compound **2p** (75 MHz, acetone-d$_6$, 25ºC)

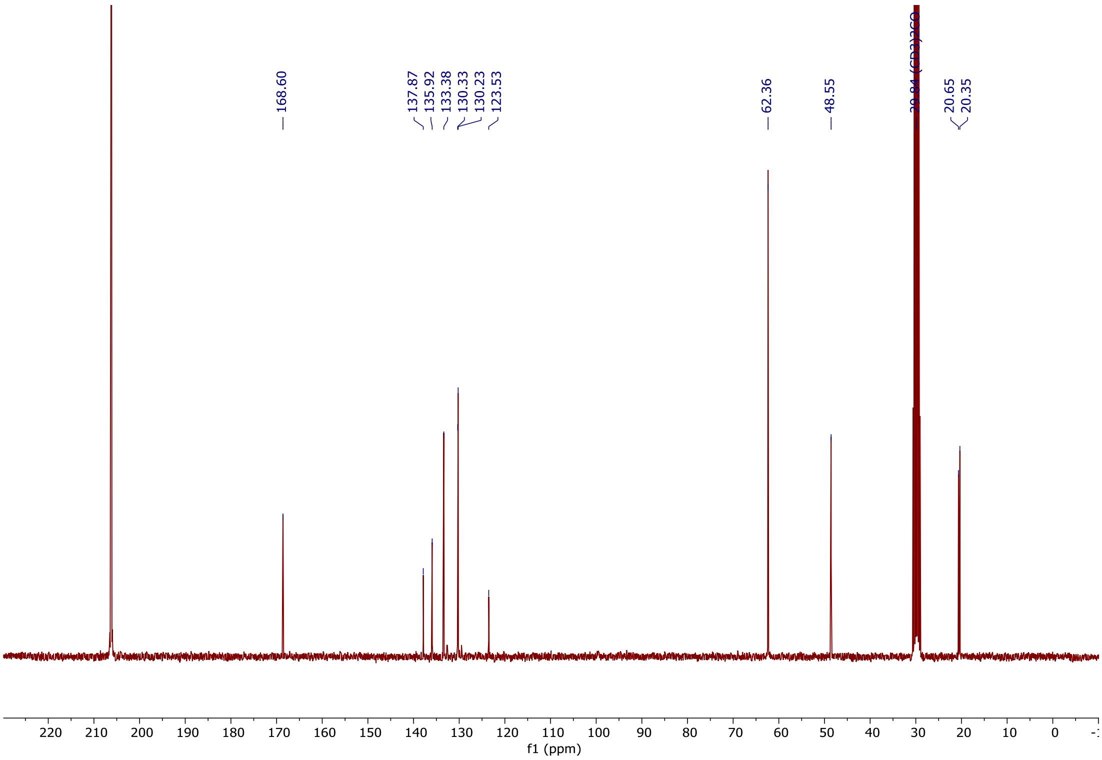

$^{1}H$ NMR of compound **2q** (300 MHz, acetone-$d_6$, 25ºC)

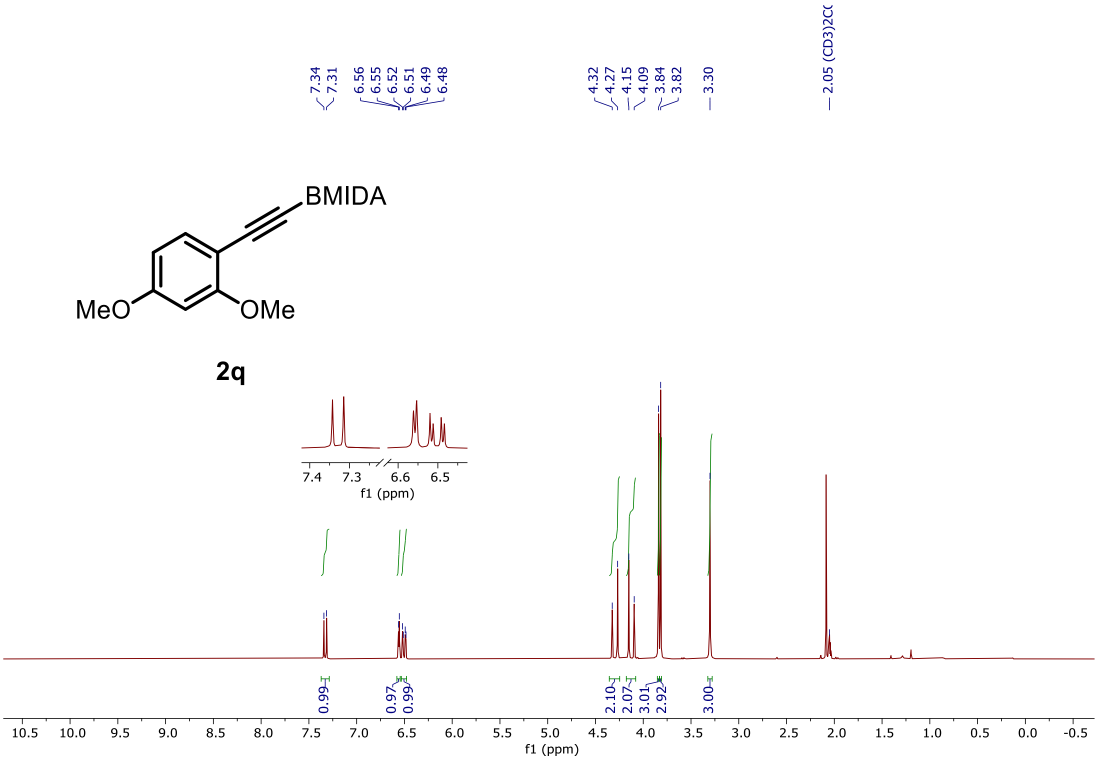


$^{13}C$ NMR of compound **2q** (75 MHz, acetone-$d_6$, 25ºC)

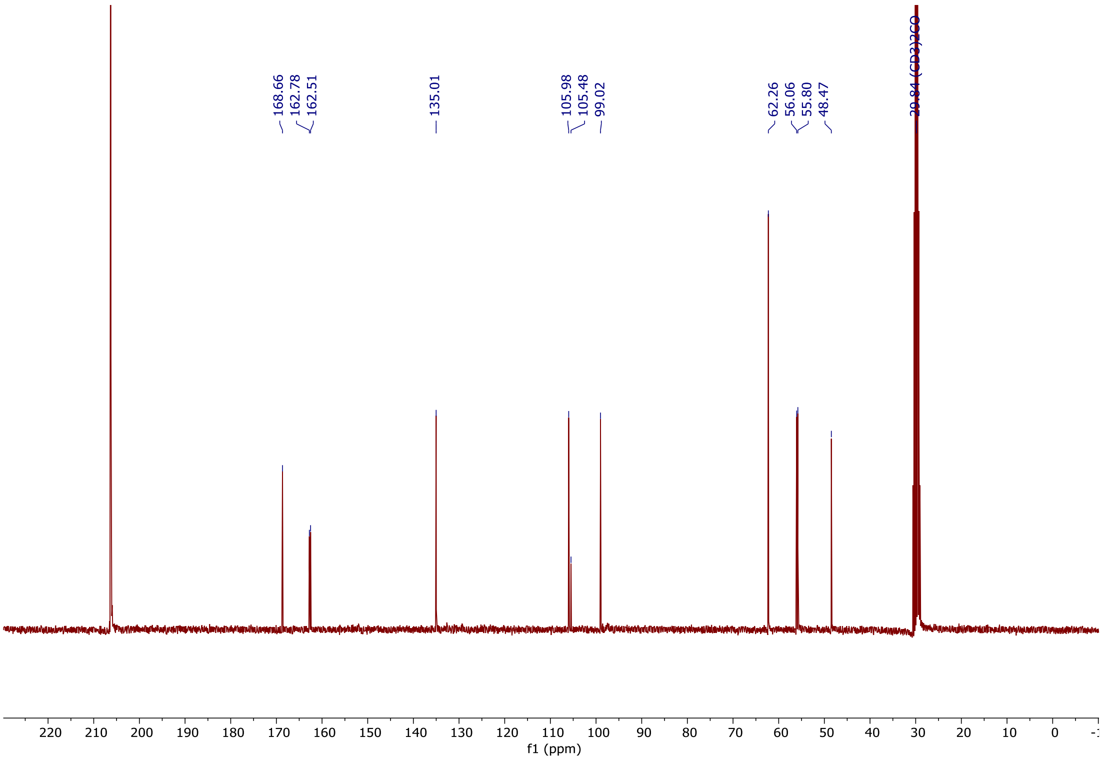

$^{1}$H NMR of compound **2r** (300 MHz, acetone-d$_6$, 25ºC)

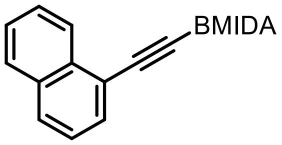


**2r**

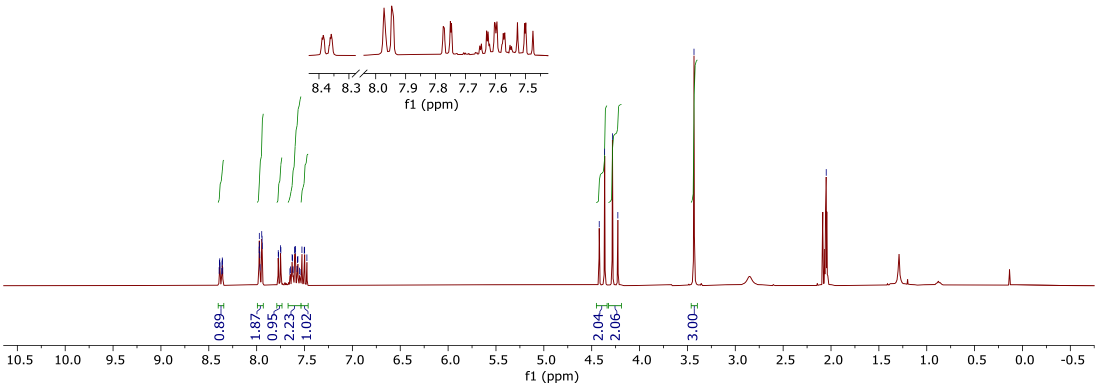


$^{13}$C NMR of compound **2r** (75 MHz, acetone-d$_6$, 25ºC)

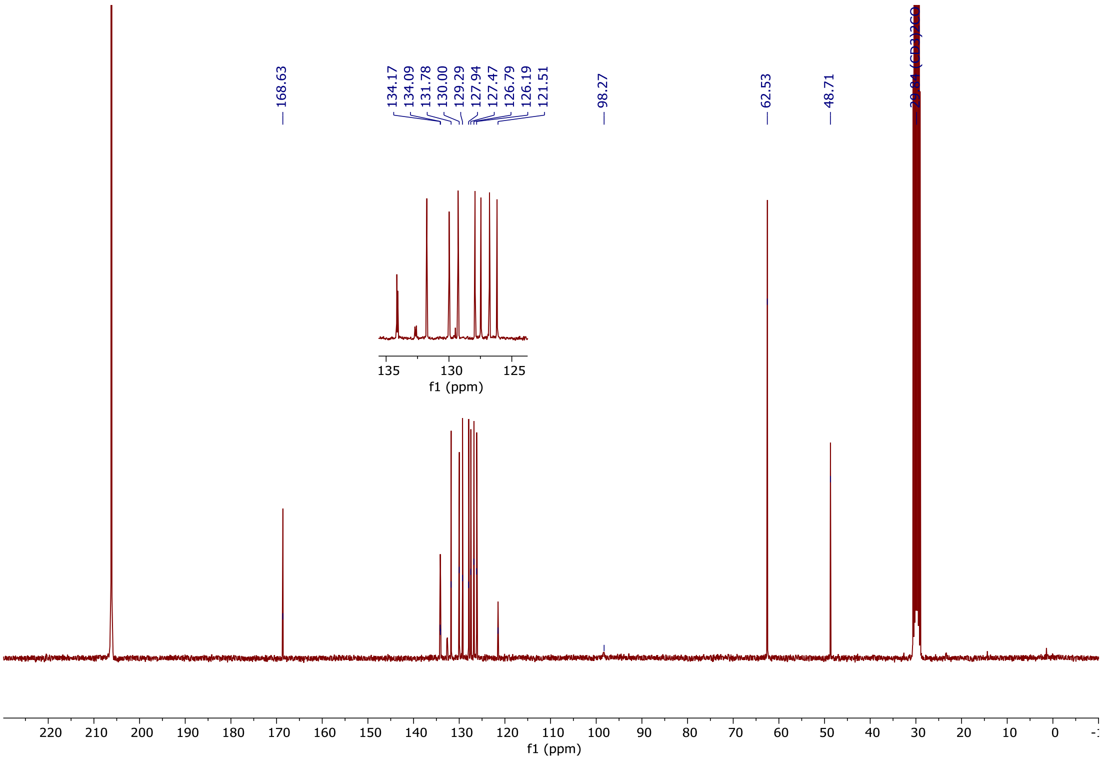

$^{1}H$ NMR of compound **2t** (300 MHz, acetone-$d_6$, 25ºC)

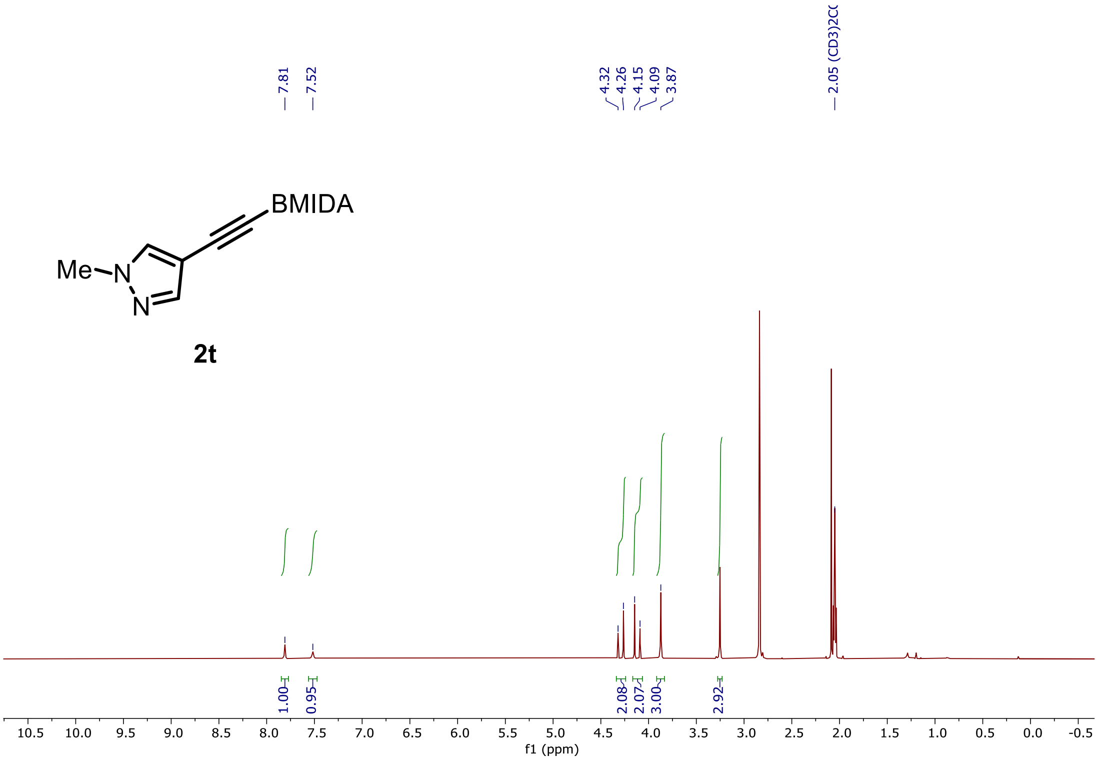


$^{13}C$ NMR of compound **2t** (75 MHz, acetone-$d_6$, 25ºC)

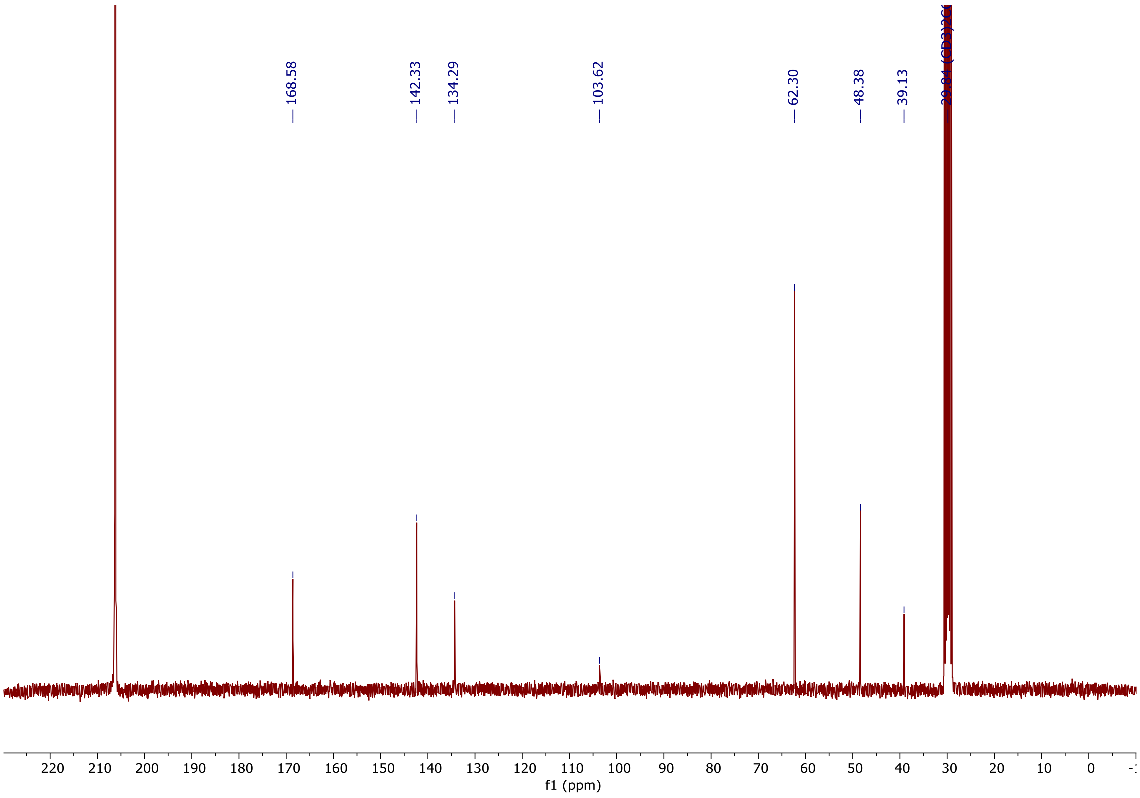

$^{1}$H NMR of compound **2u** (300 MHz, acetone-d$_6$, 25ºC)

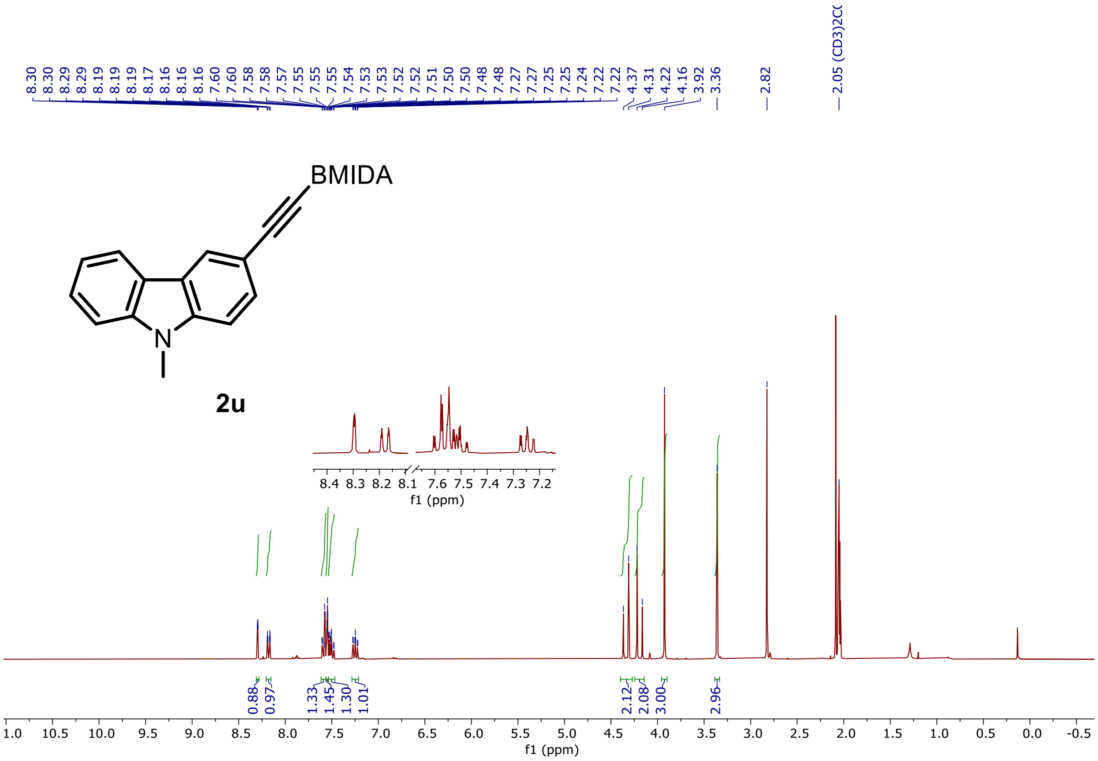


$^{13}$C NMR of compound **2u** (75 MHz, acetone-d$_6$, 25ºC)

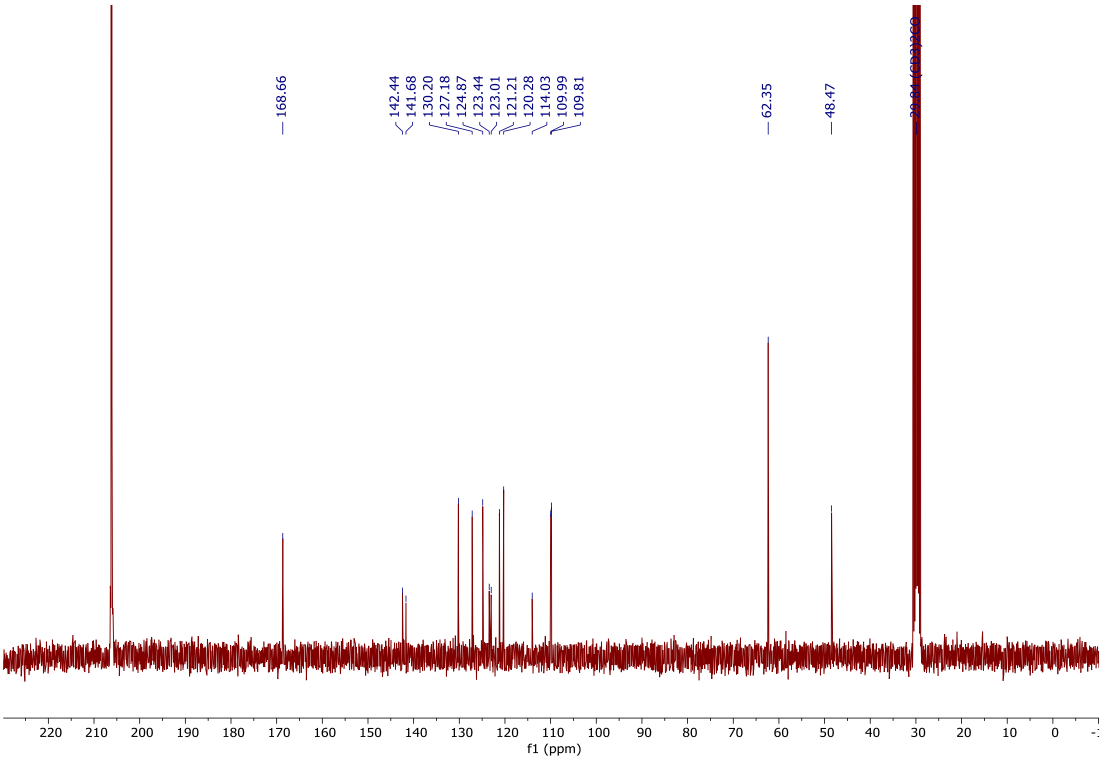

$^{1}$H NMR of compound **2v** (300 MHz, acetone-d$_6$, 25ºC)

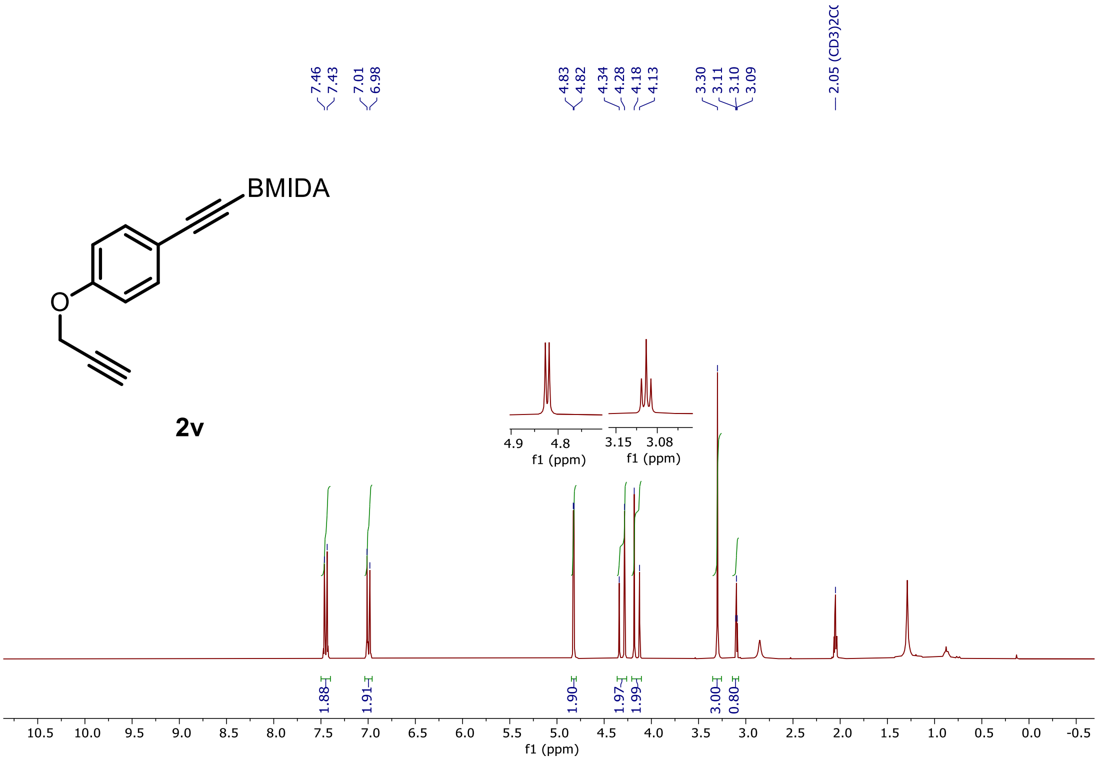

$^{13}$C NMR of compound **2v** (75 MHz, acetone-d$_6$, 25ºC)

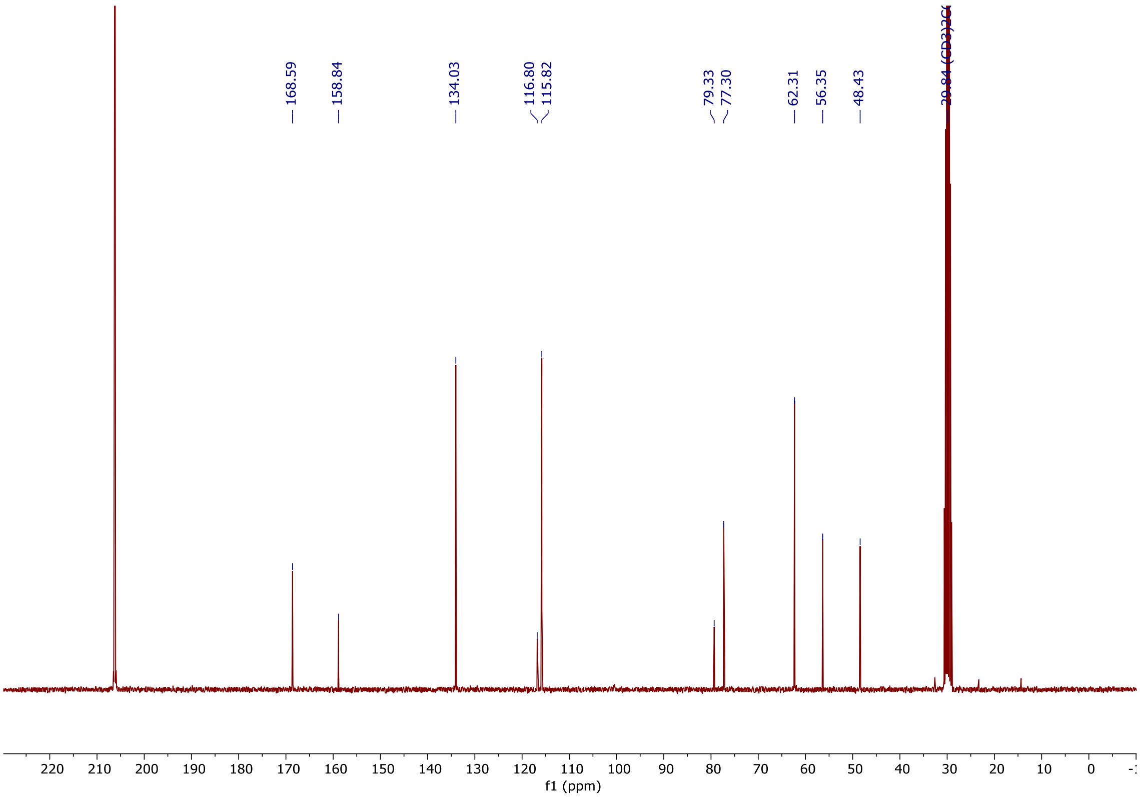

$^{1}H$ NMR of compound **3a** (300 MHz, acetone-$d_6$, 25ºC)

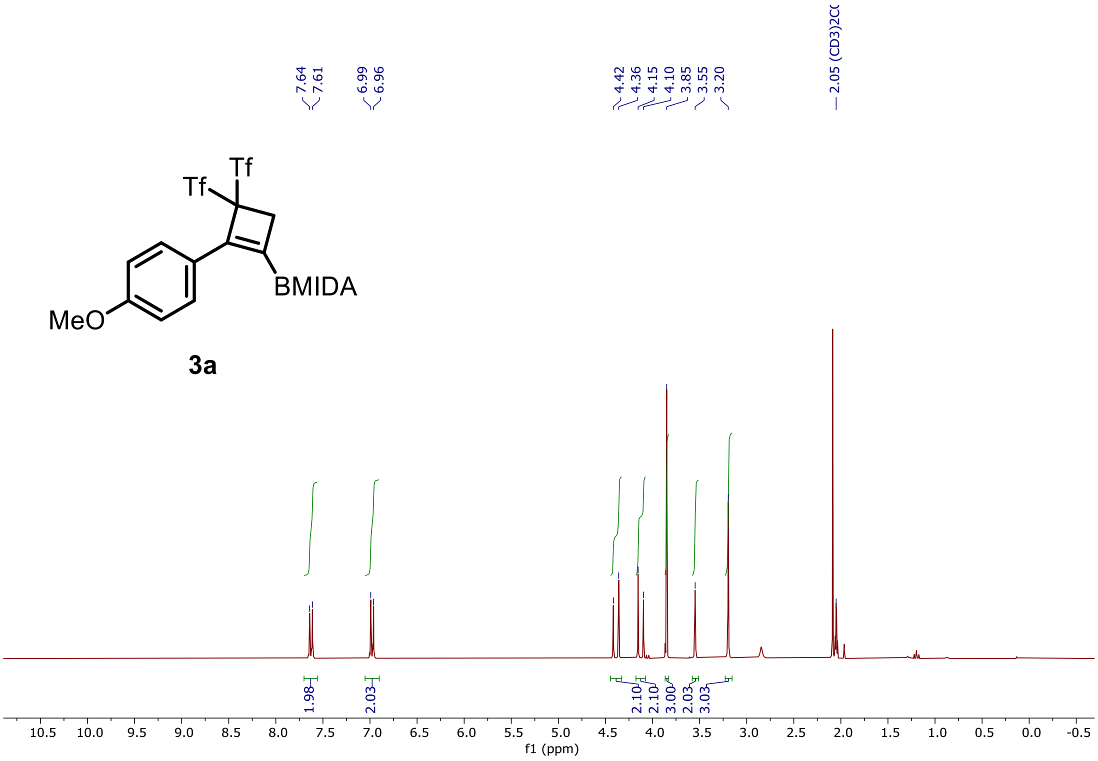


$^{13}C$ NMR of compound **3a** (75 MHz, $CDCl_3$, 25ºC)

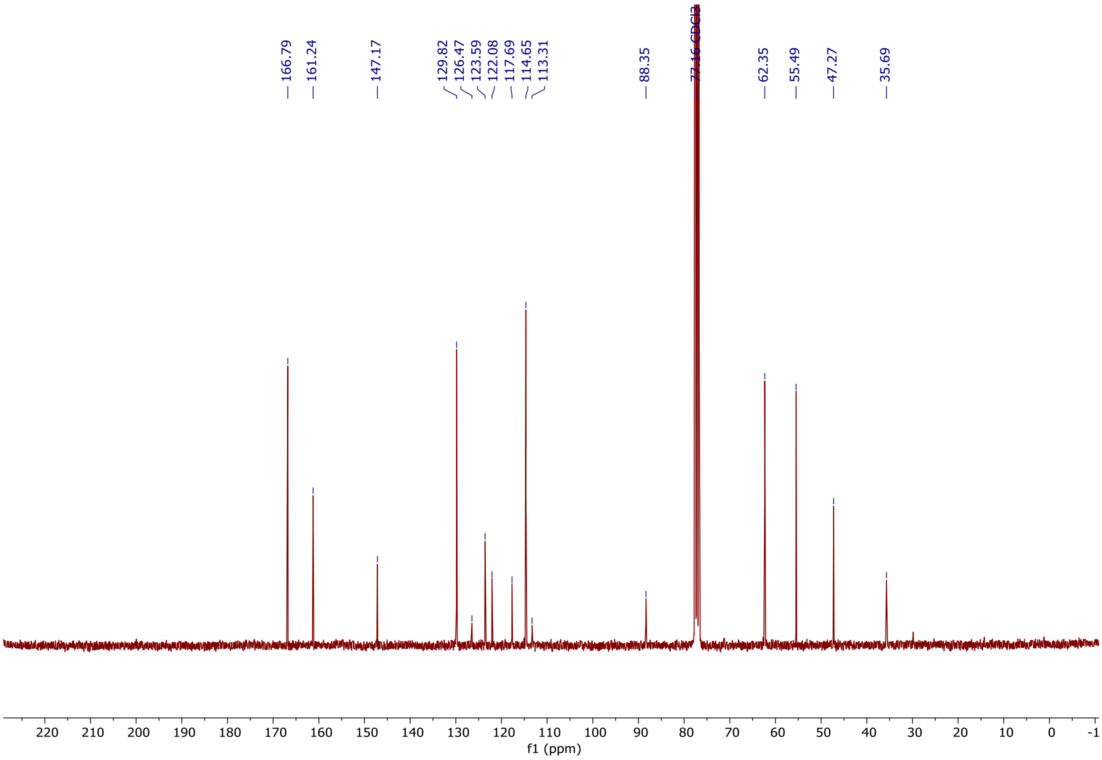

$^{19}$F NMR of compound **3a** (282 MHz, acetone-d$_6$, 25ºC)

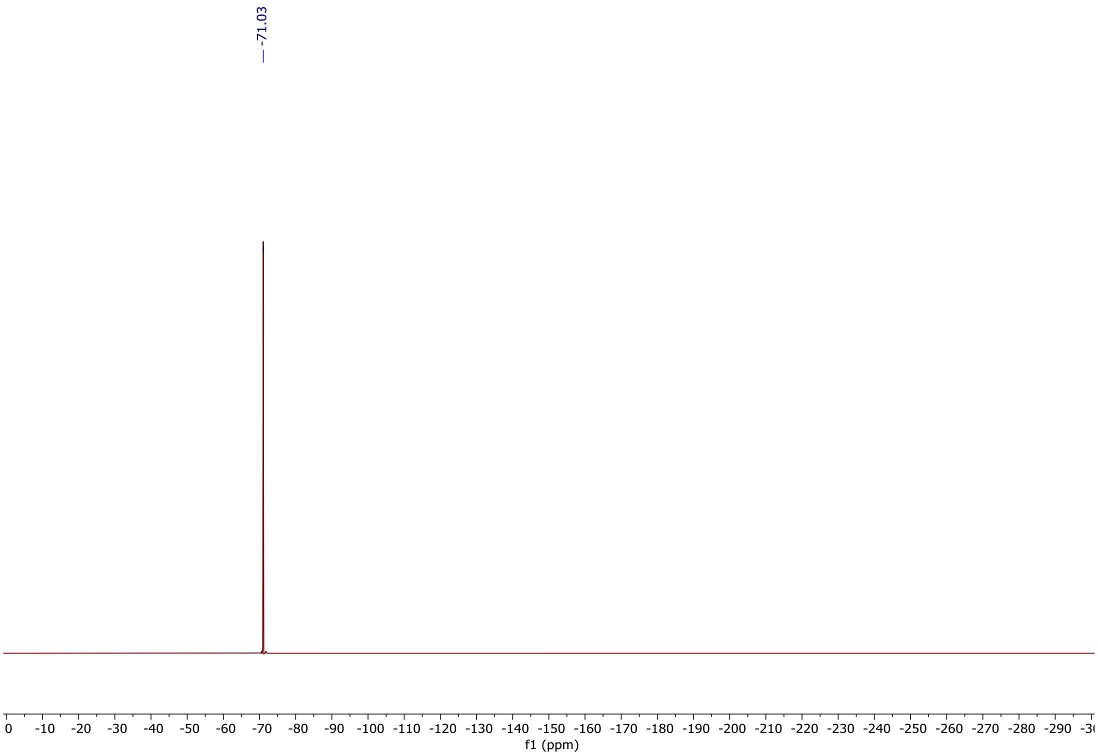

$^{1}$H NMR of compound **3b** (300 MHz, acetone-d$_6$, 25ºC)

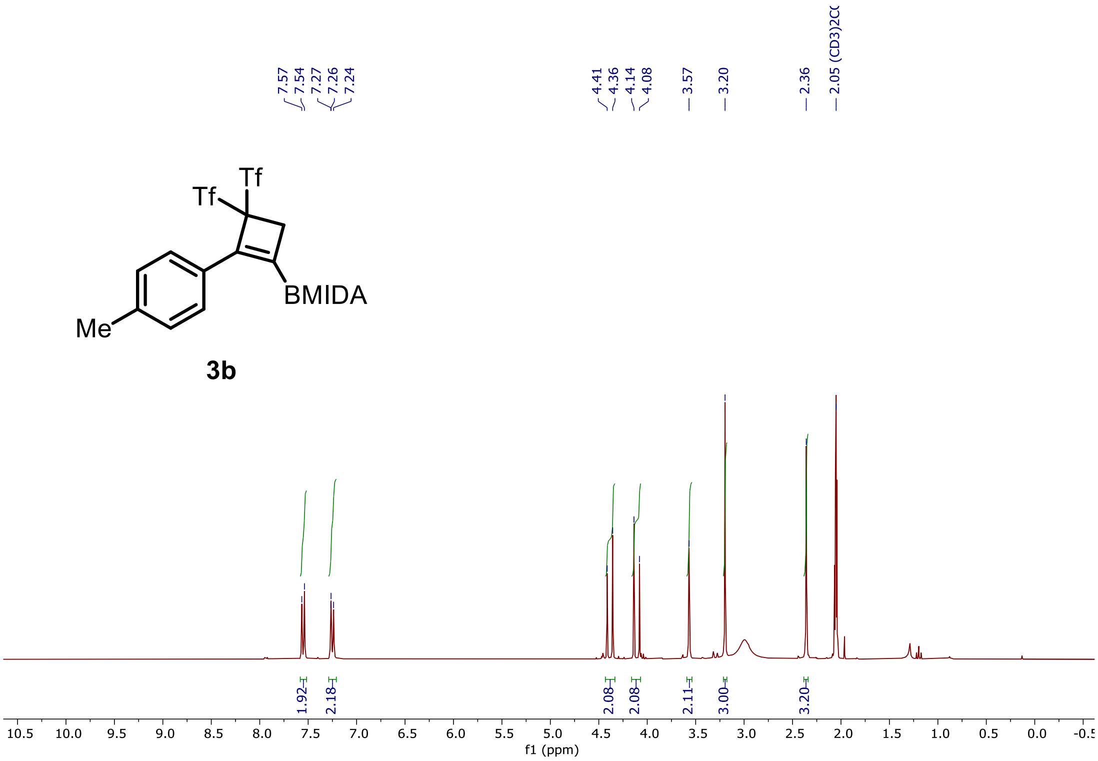


$^{13}$C NMR of compound **3b** (75 MHz, acetone-d$_6$, 25ºC)

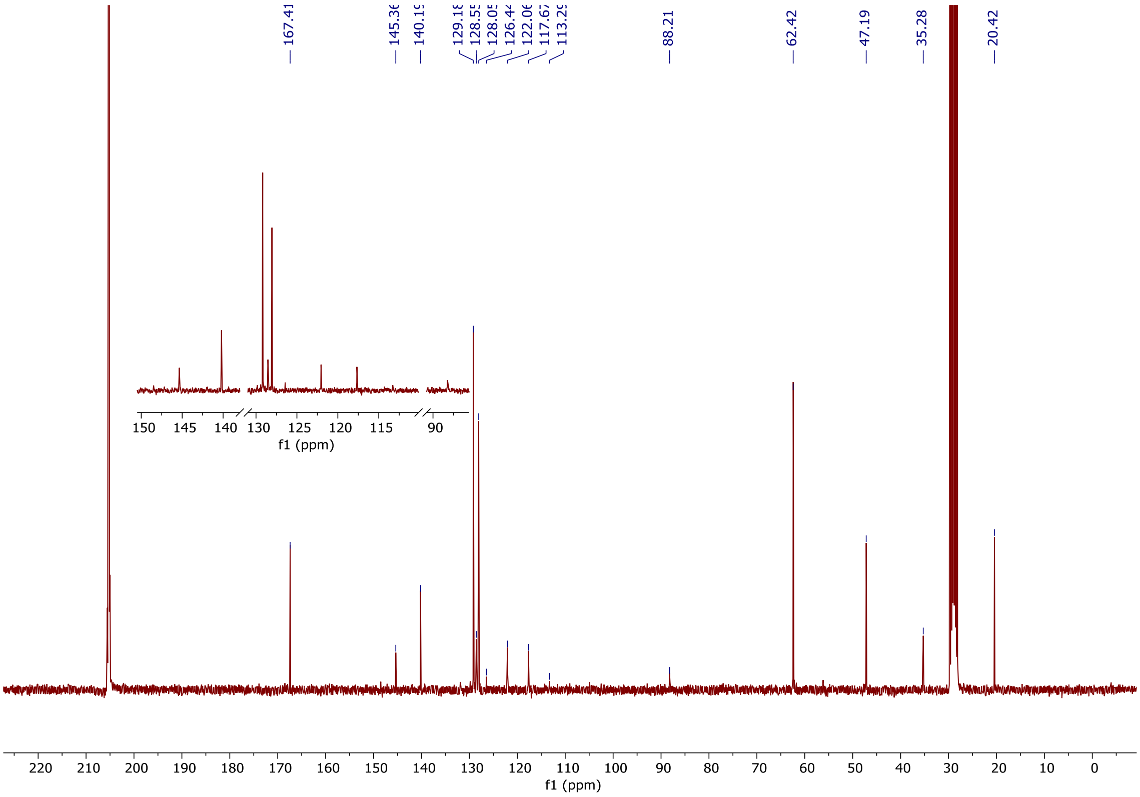

$^{19}F$ NMR of compound **3b** (282 MHz, acetone-$d_6$, 25ºC)

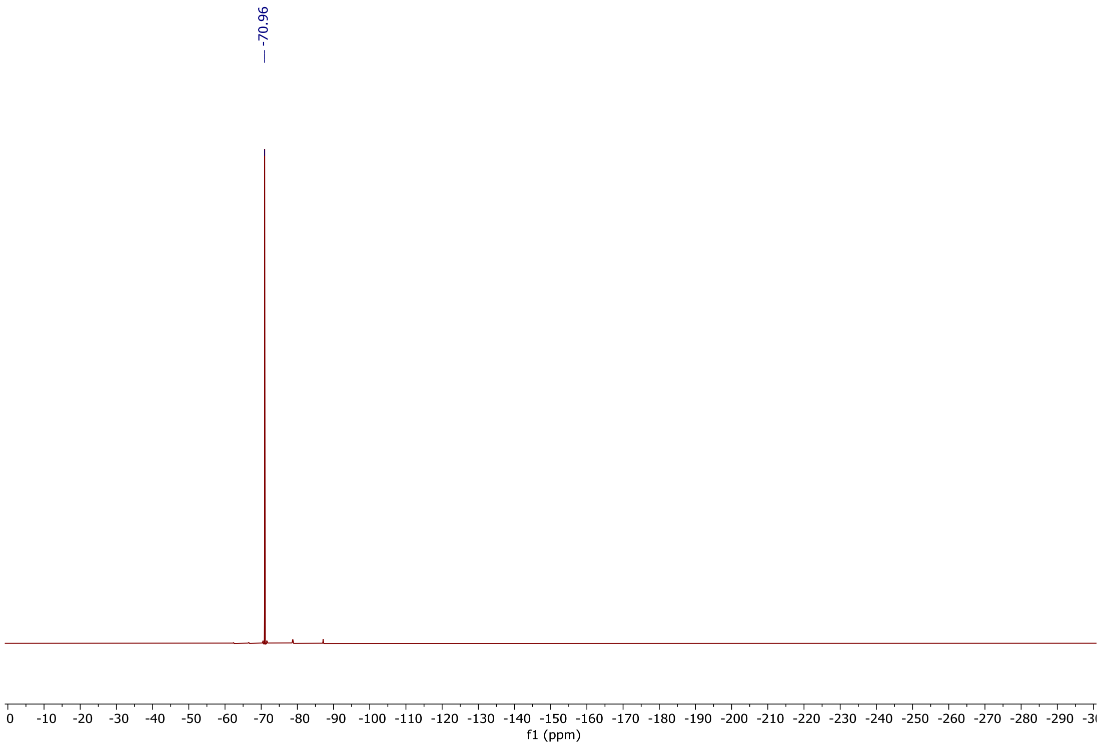

$^{1}$H NMR of compound **3c** (300 MHz, acetone-d$_6$, 25ºC)

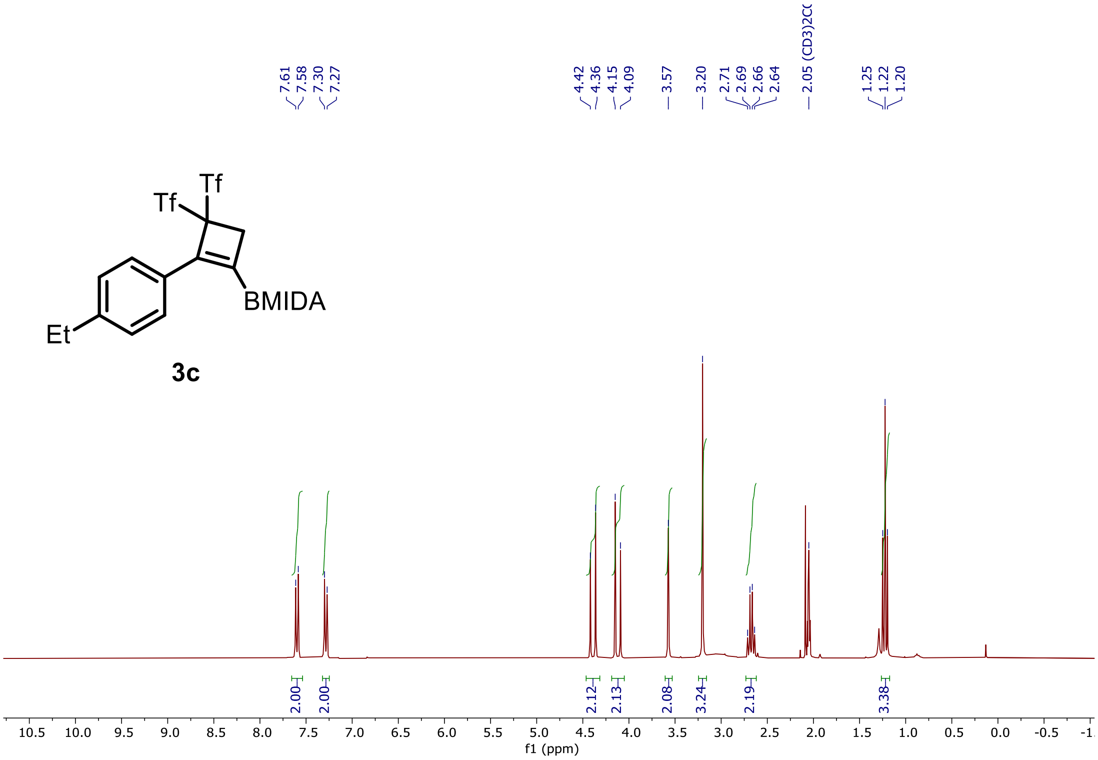


$^{13}$C NMR of compound **3c** (75 MHz, acetone-d$_6$, 25ºC)

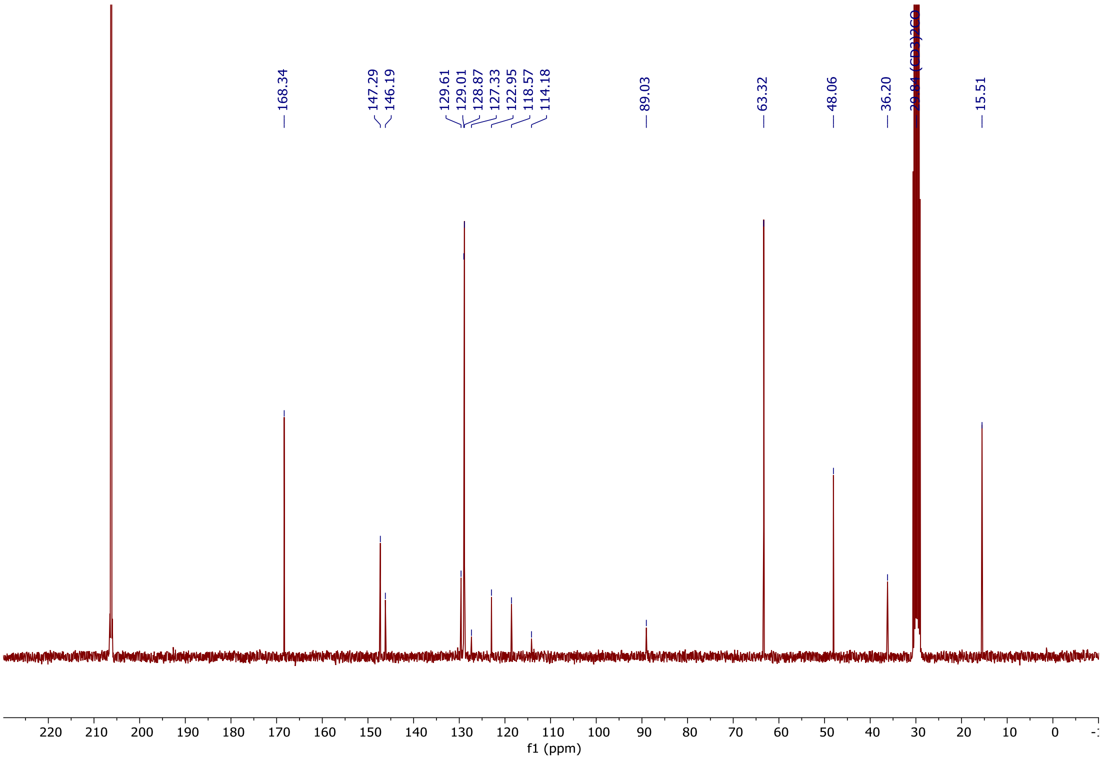

$^{19}F$ NMR of compound **3c** (282 MHz, acetone-$d_6$, 25ºC)

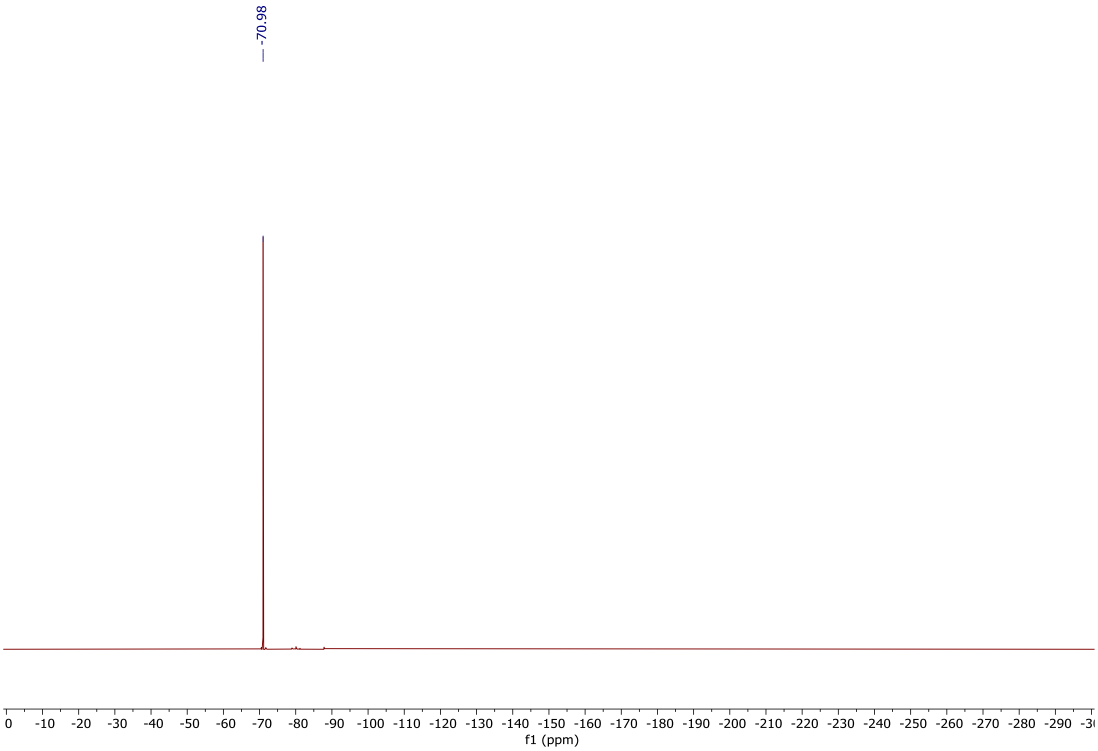

$^{1}H$ NMR of compound **3d** (300 MHz, acetone-$d_6$, 25ºC)

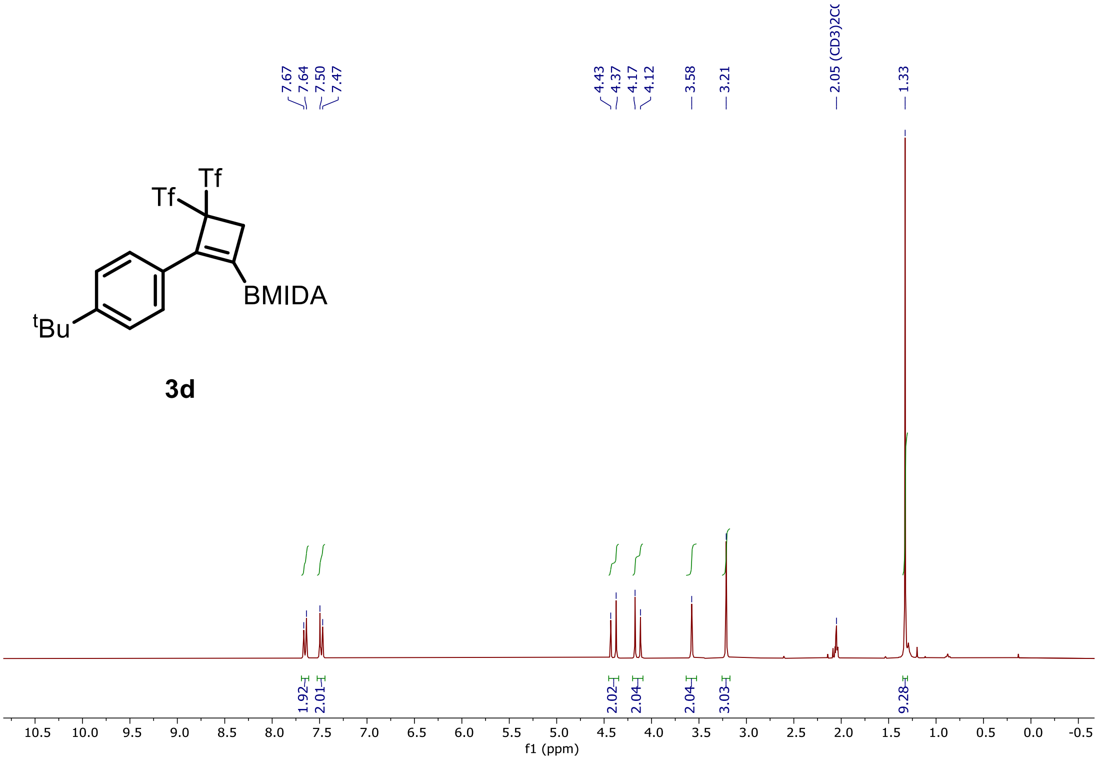


$^{13}C$ NMR of compound **3d** (75 MHz, acetone-$d_6$, 25ºC)

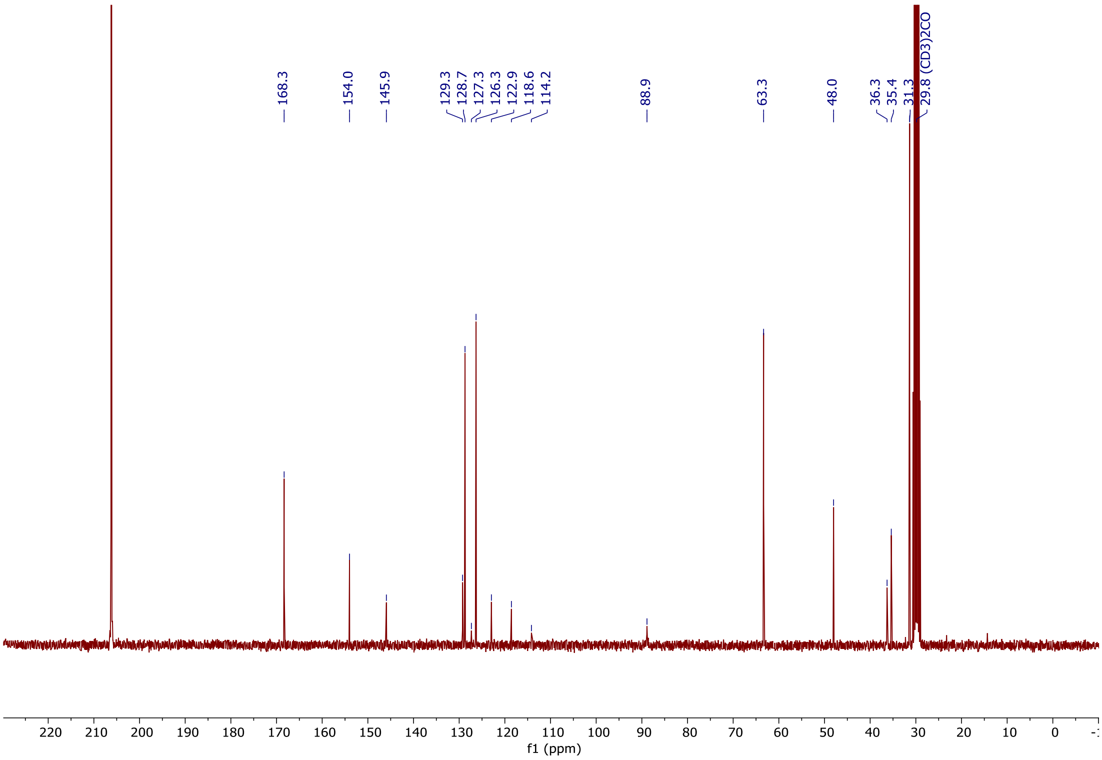

$^{19}F$ NMR of compound **3d** (282 MHz, acetone-$d_6$, 25ºC)

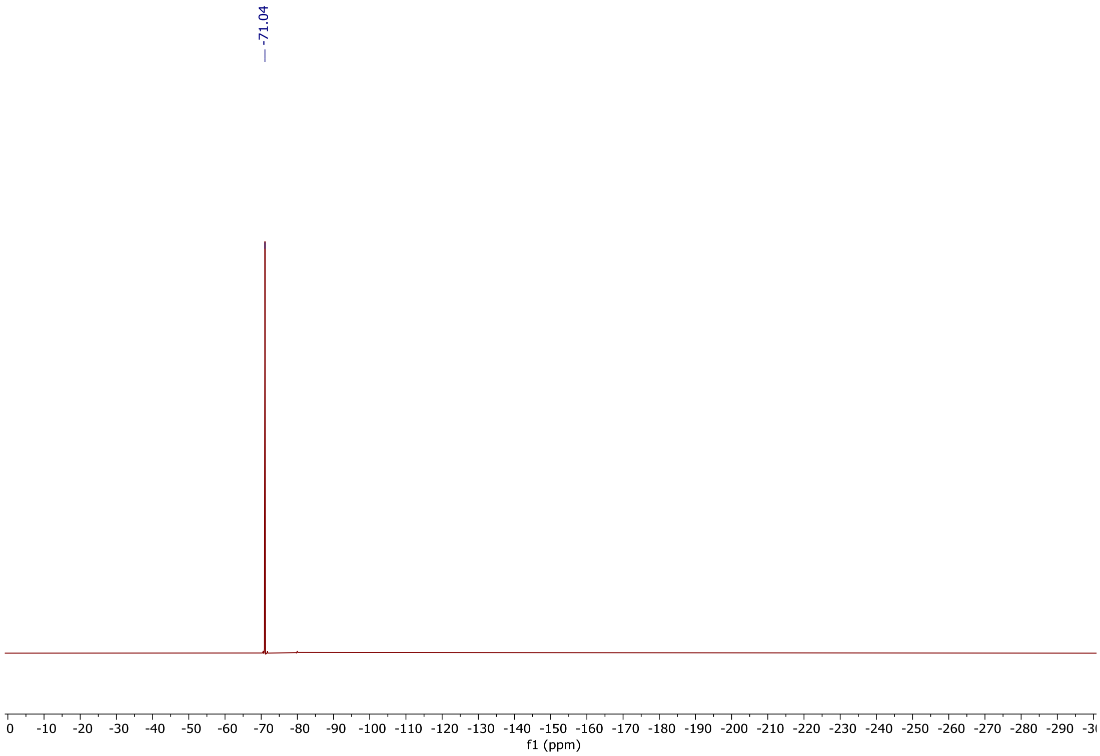

$^{1}H$ NMR of compound **3e** (300 MHz, acetone-d$_6$, 25ºC)

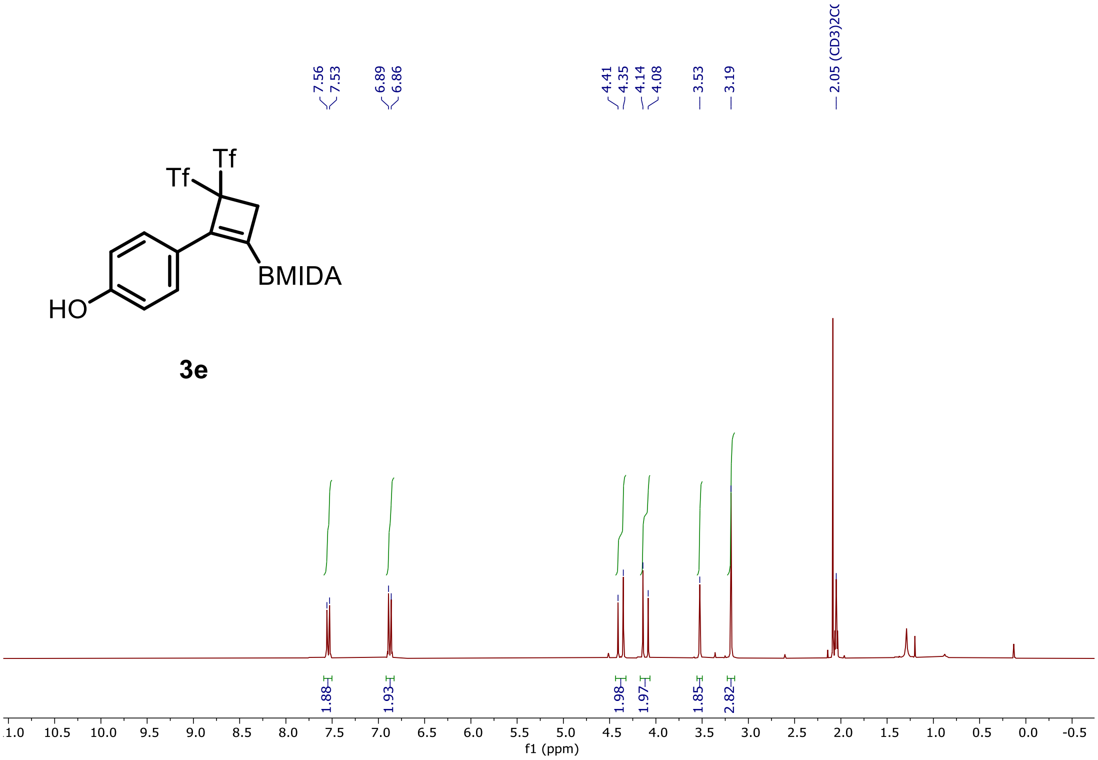


$^{13}C$ NMR of compound **3e** (75 MHz, acetone-d$_6$, 25ºC)

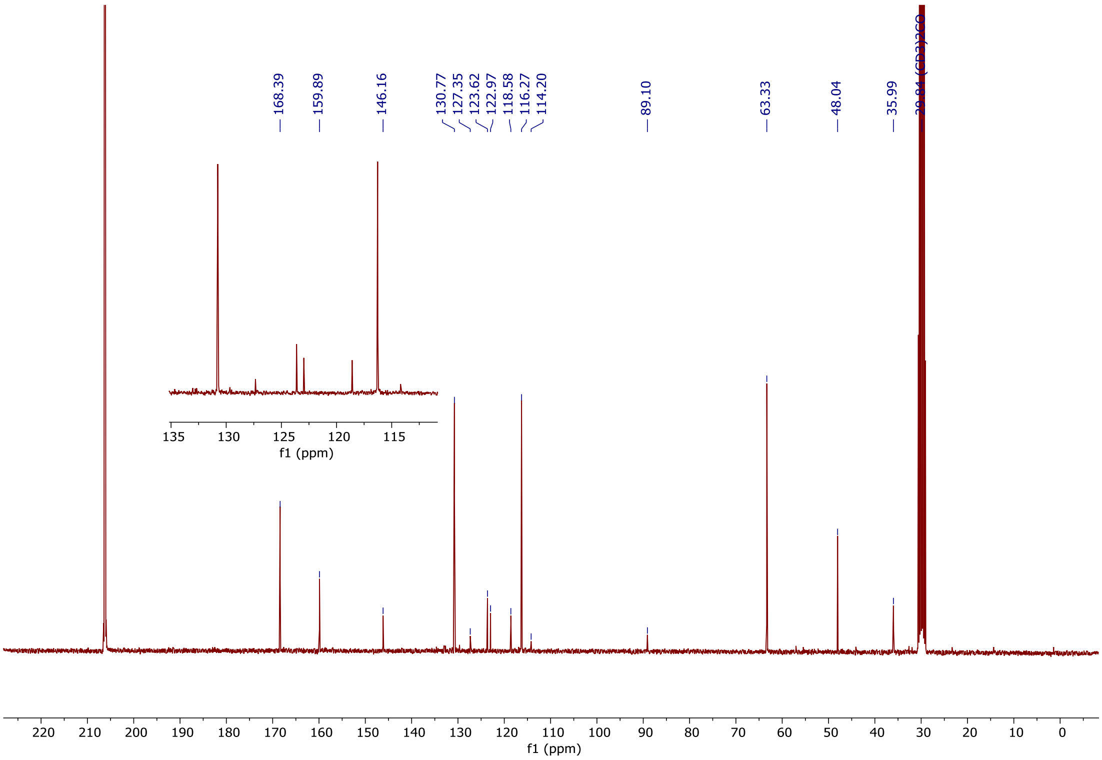

$^{19}F$ NMR of compound **3e** (282 MHz, acetone-$d_6$, 25ºC)

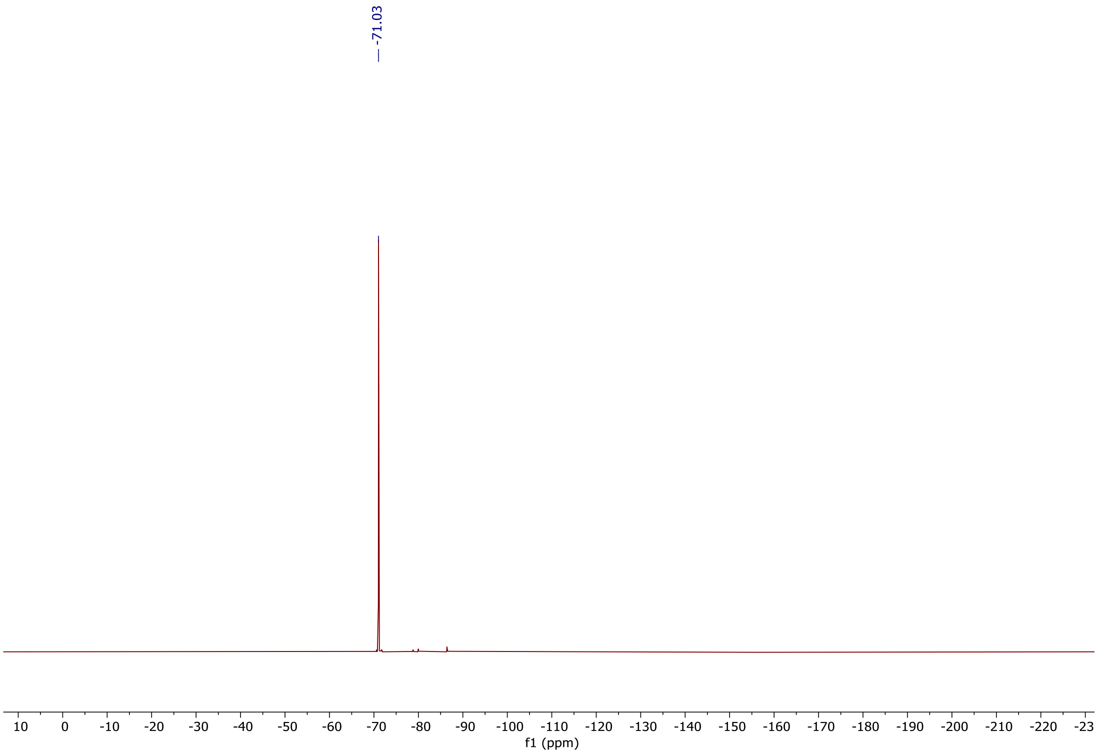

$^{1}H$ NMR of compound **3f** (300 MHz, acetone-$d_6$, 25ºC)

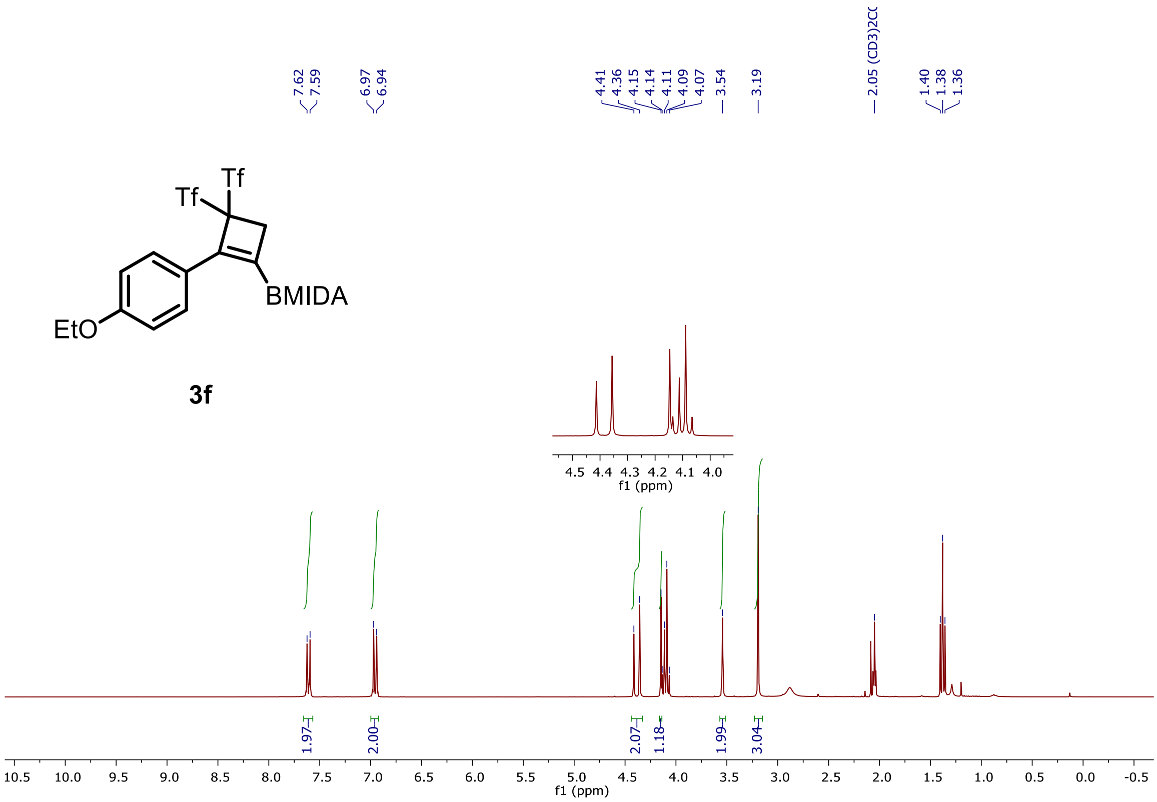

$^{13}C$ NMR of compound **3f** (75 MHz, acetone-$d_6$, 25ºC)

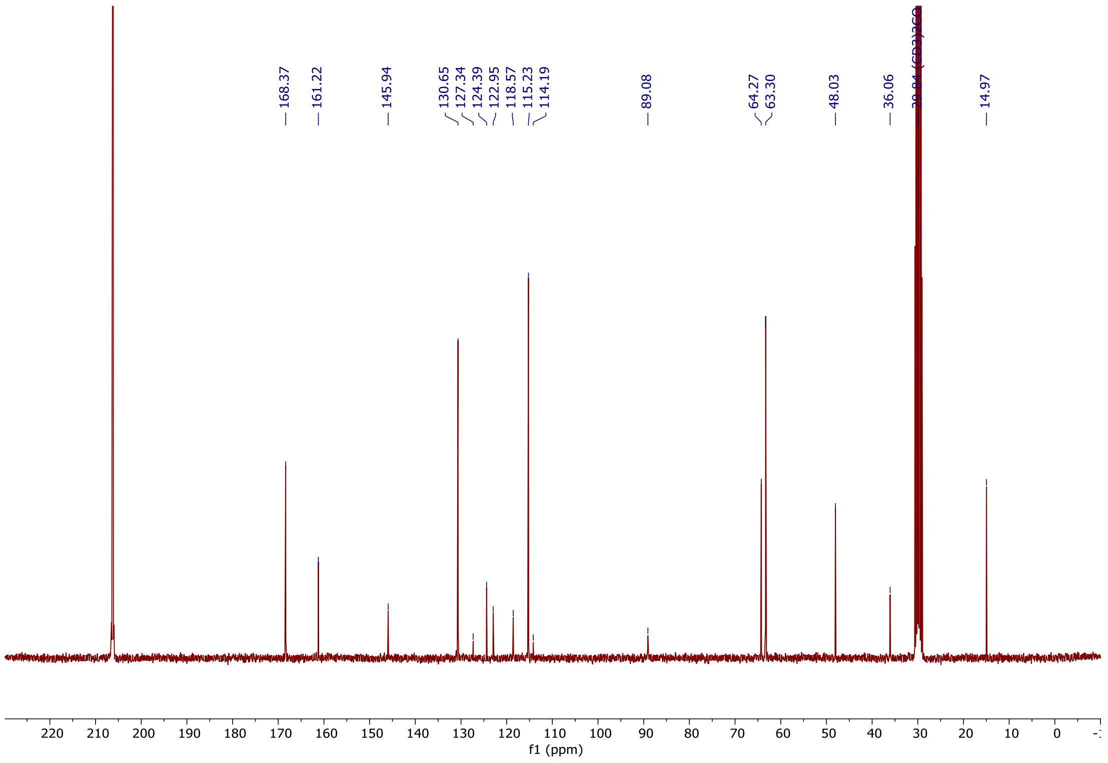

$^{19}F$ NMR of compound **3f** (282 MHz, acetone-$d_6$, 25ºC)

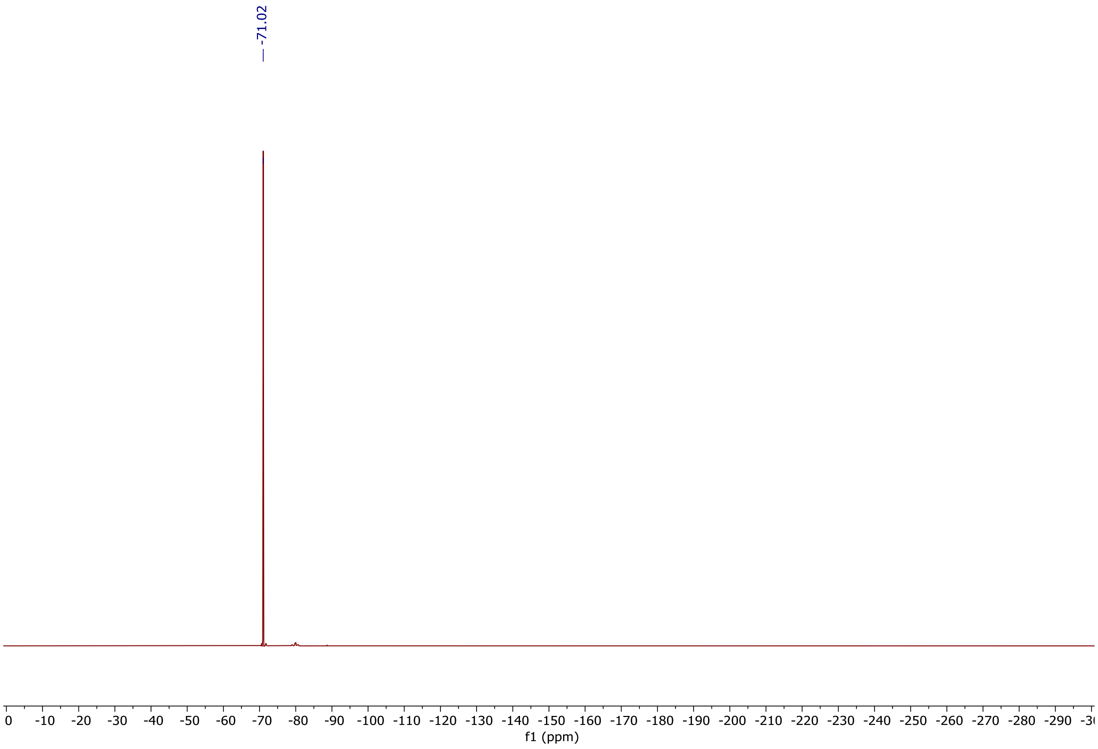

$^{1}$H NMR of compound **3g** (300 MHz, acetone-d$_6$, 25ºC)

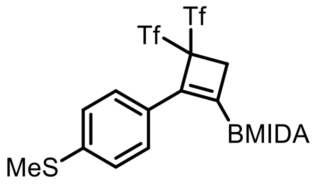


**3g**

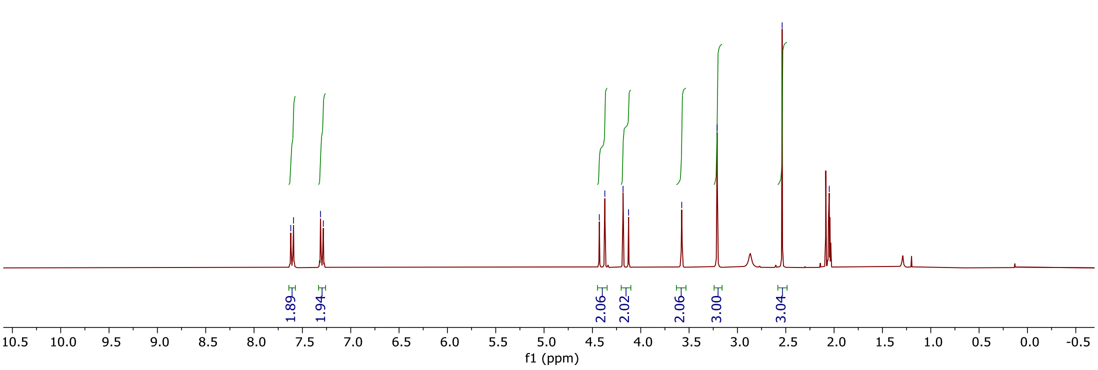


$^{13}$C NMR of compound **3g** (75 MHz, acetone-d$_6$, 25ºC)

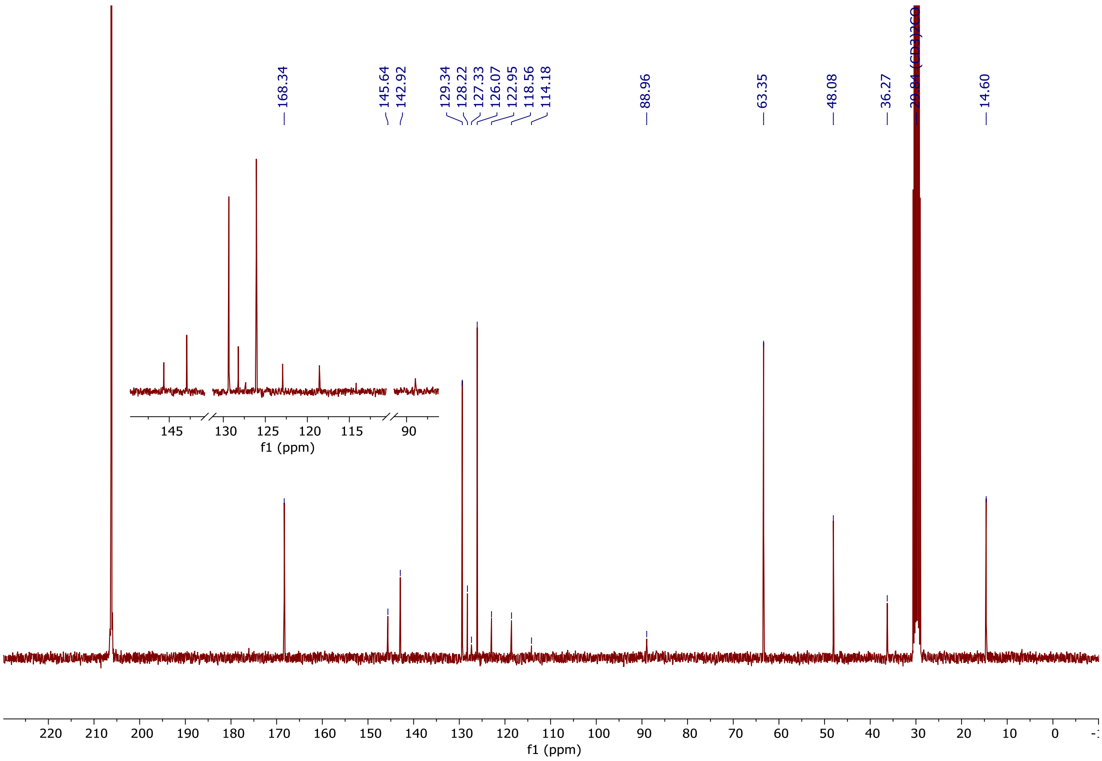

$^{19}$F NMR of compound **3g** (282 MHz, acetone-d$_6$, 25ºC)

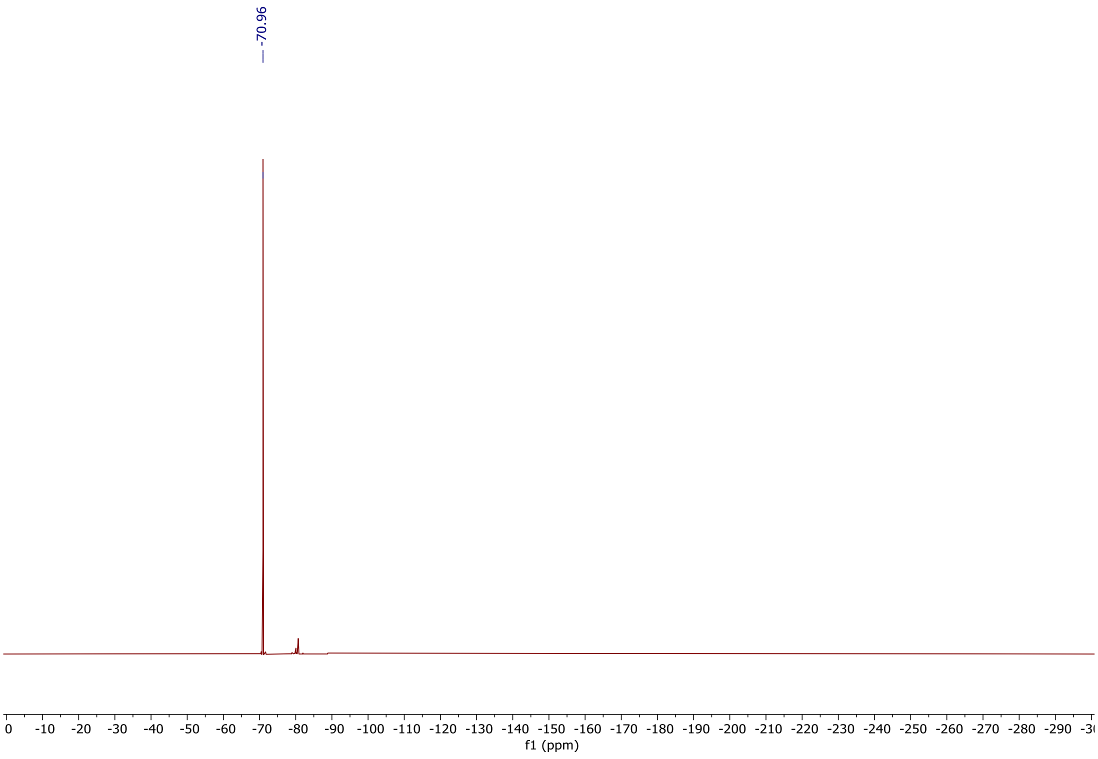

$^{1}H$ NMR of compound **3h** (300 MHz, acetone-$d_6$, 25ºC)

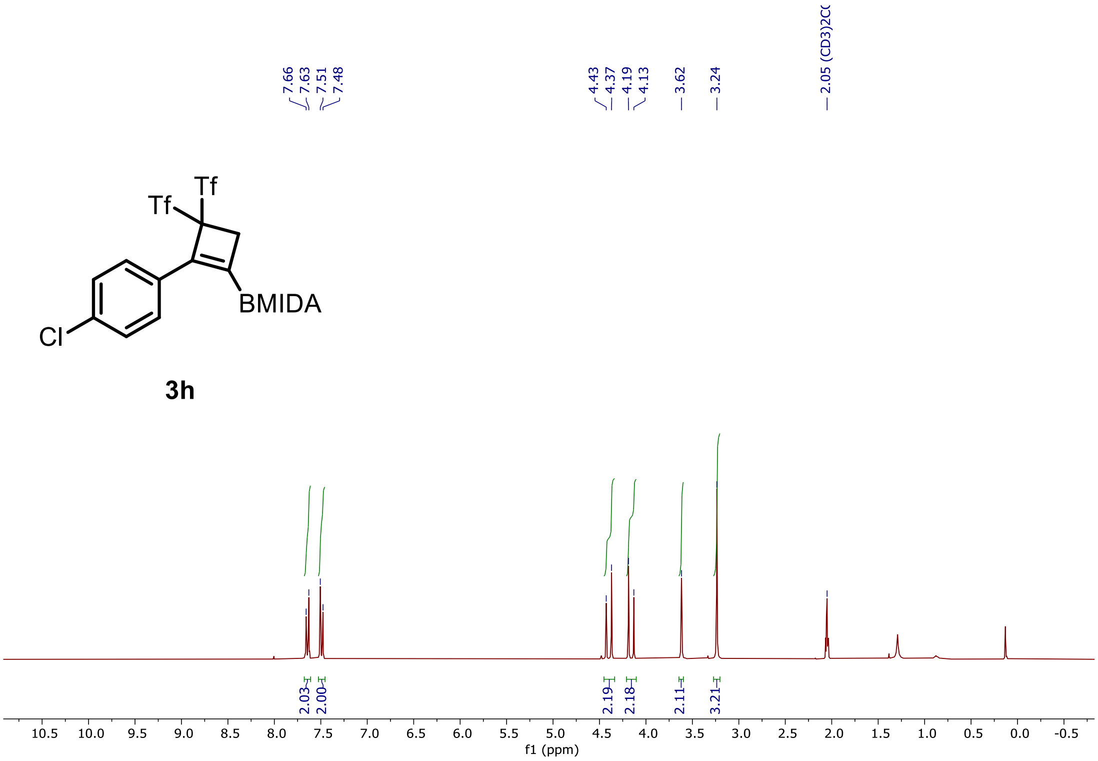


$^{13}C$ NMR of compound **3h** (75 MHz, acetone-$d_6$, 25ºC)

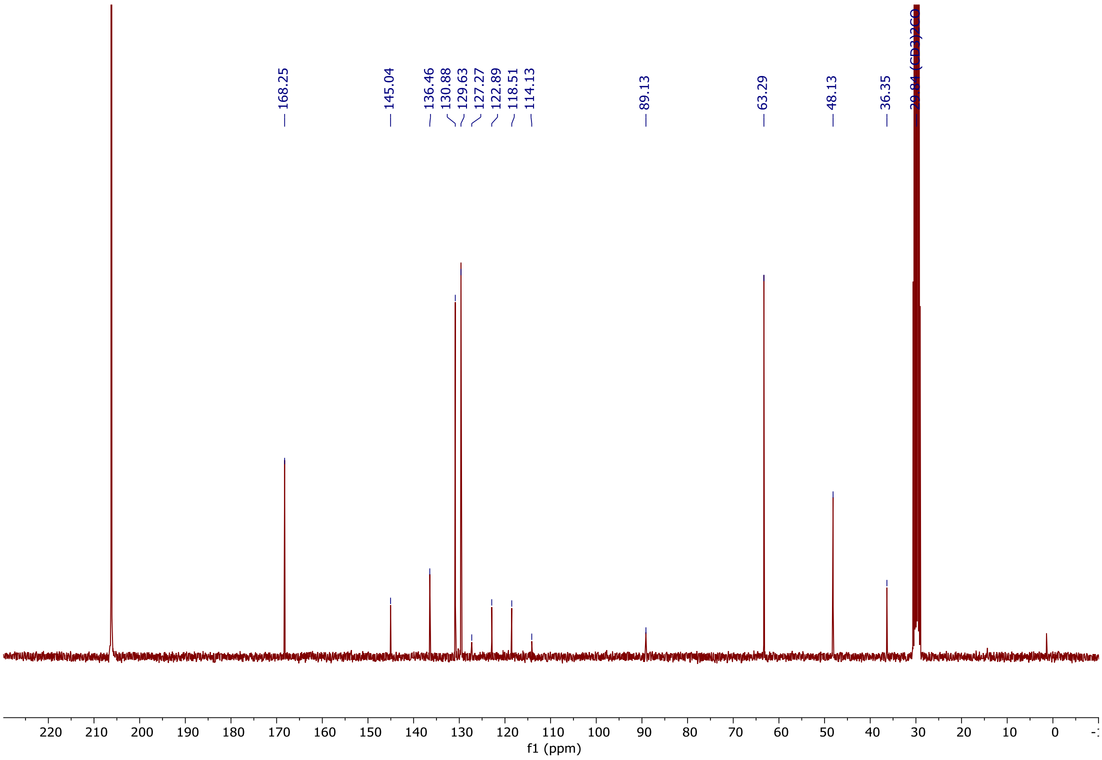

$^{19}F$ NMR of compound **3h** (282 MHz, acetone-$d_6$, 25ºC)

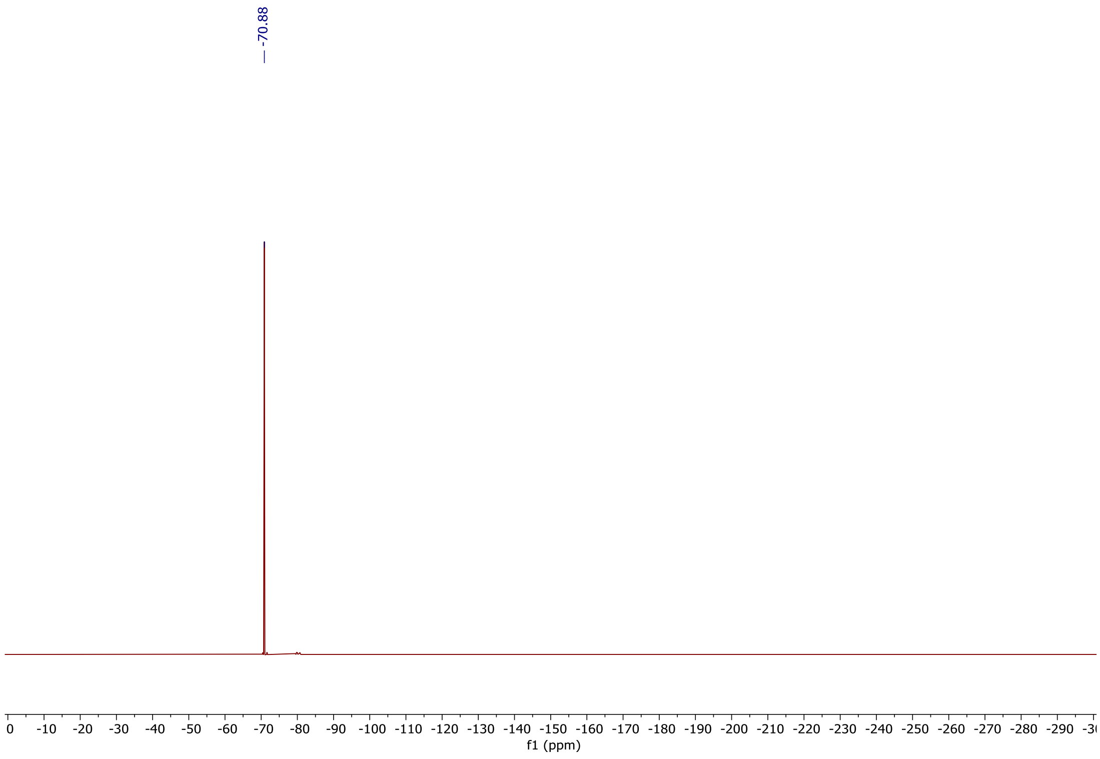

$^{1}$H NMR of compound **3i** (300 MHz, acetone-d$_6$, 25ºC)

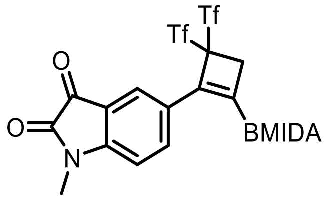

**3i**

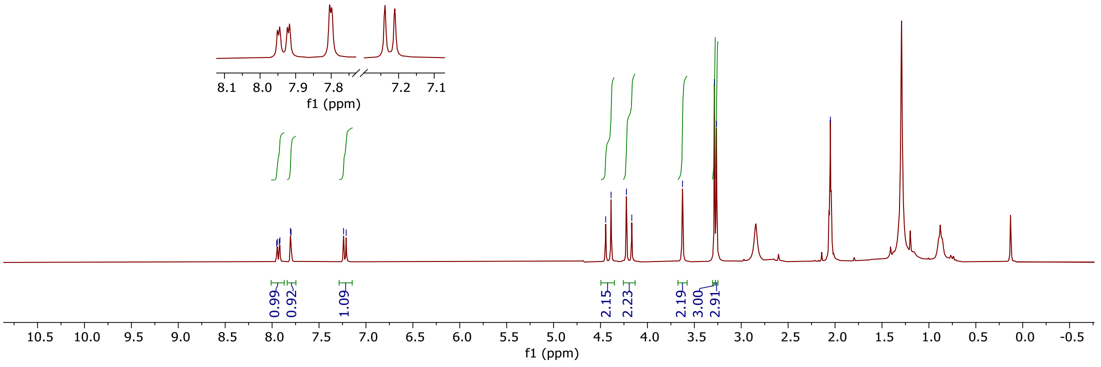

$^{13}$C NMR of compound **3i** (75 MHz, acetone-d$_6$, 25ºC)

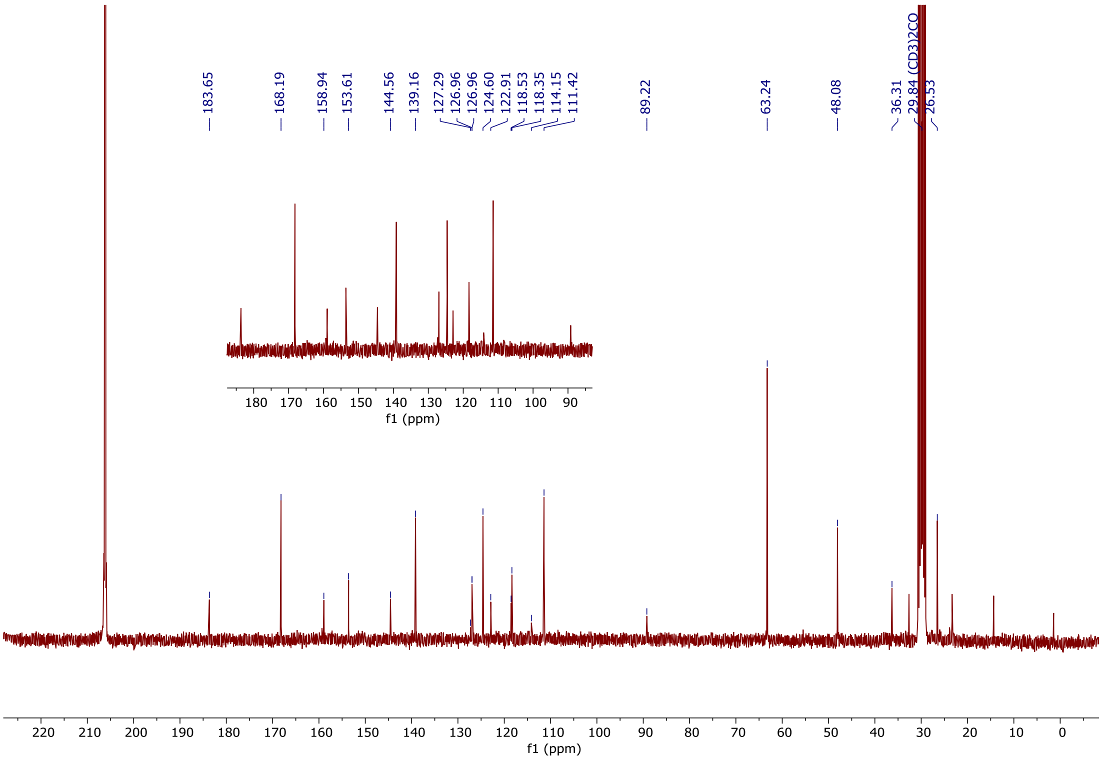

$^{19}F$ NMR of compound **3i** (282 MHz, acetone-$d_6$, 25ºC)

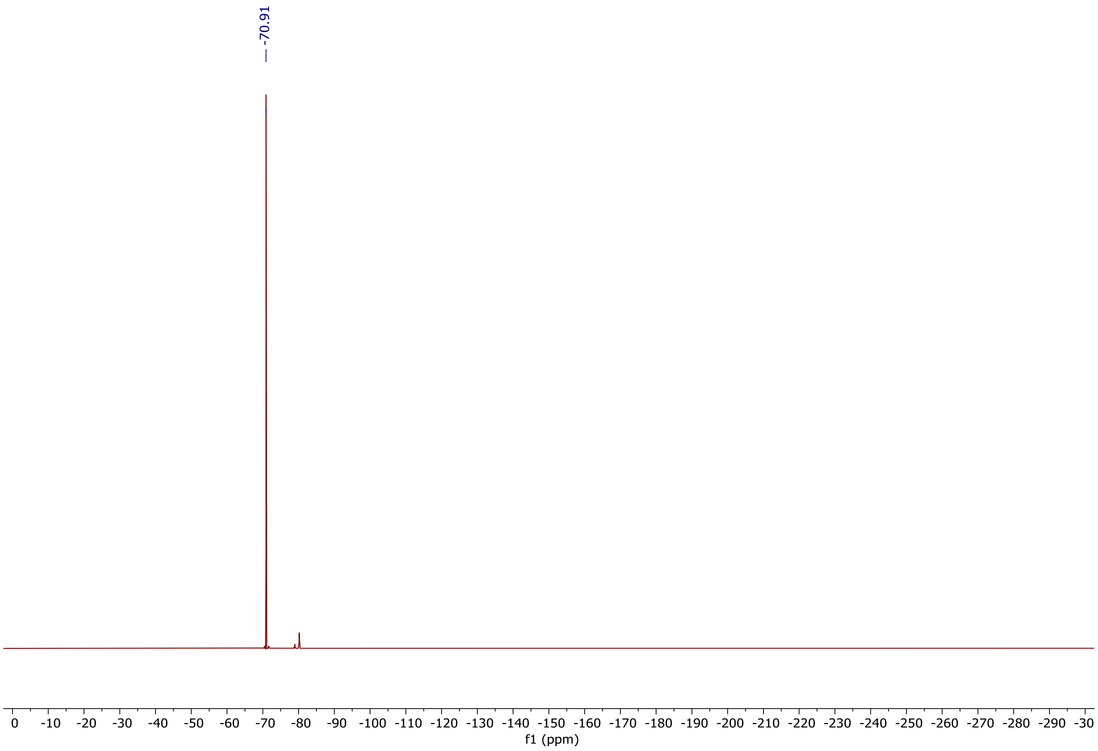

$^{1}H$ NMR of compound **3j** (300 MHz, acetone-$d_6$, 25ºC)

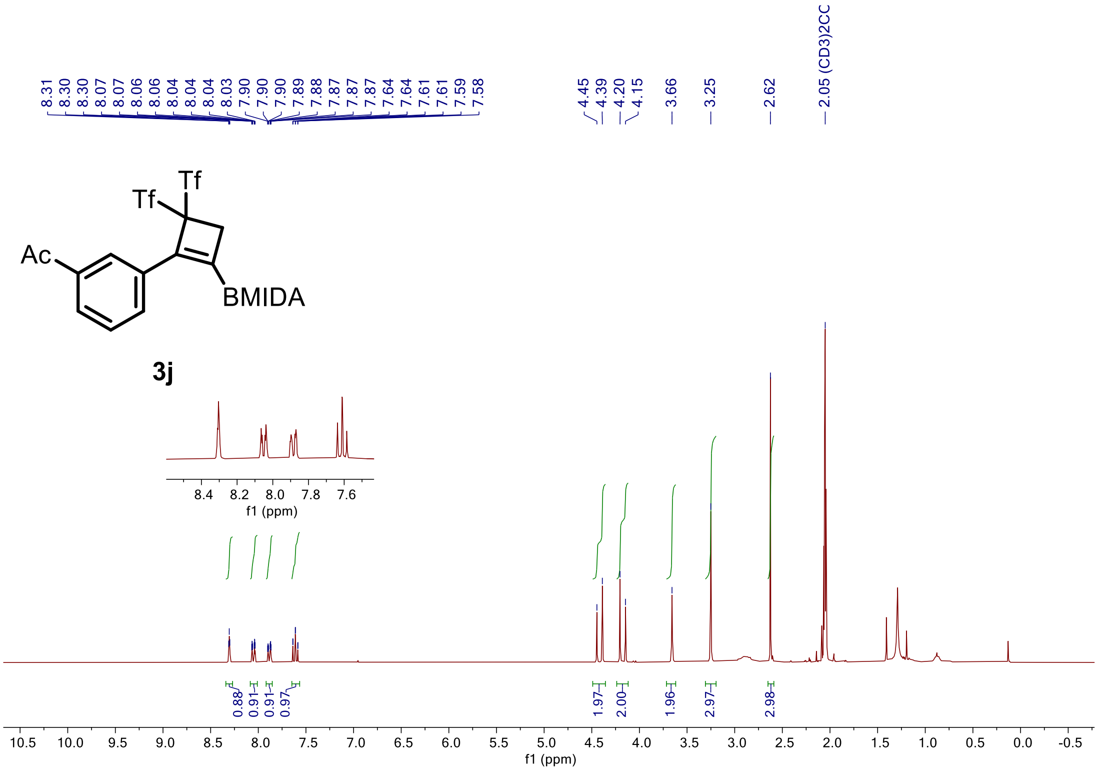


$^{13}C$ NMR of compound **3j** (176 MHz, acetone-$d_6$, 25ºC)

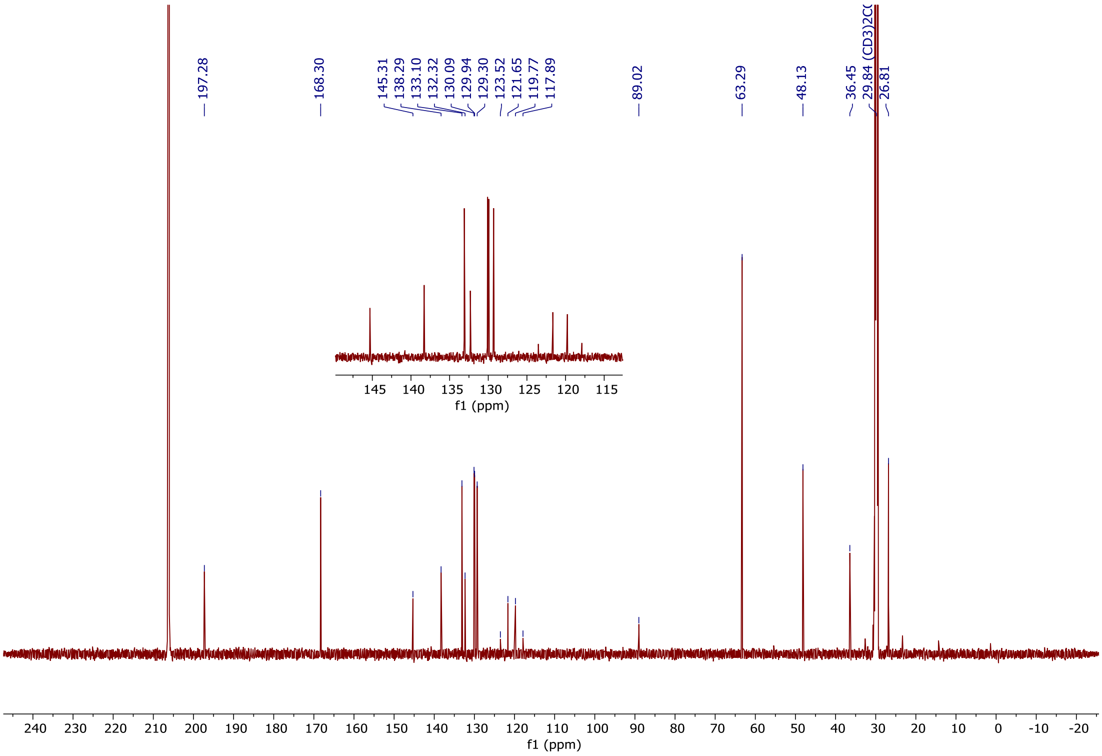

$^{19}F$ NMR of compound **3j** (282 MHz, acetone-$d_6$, 25ºC)

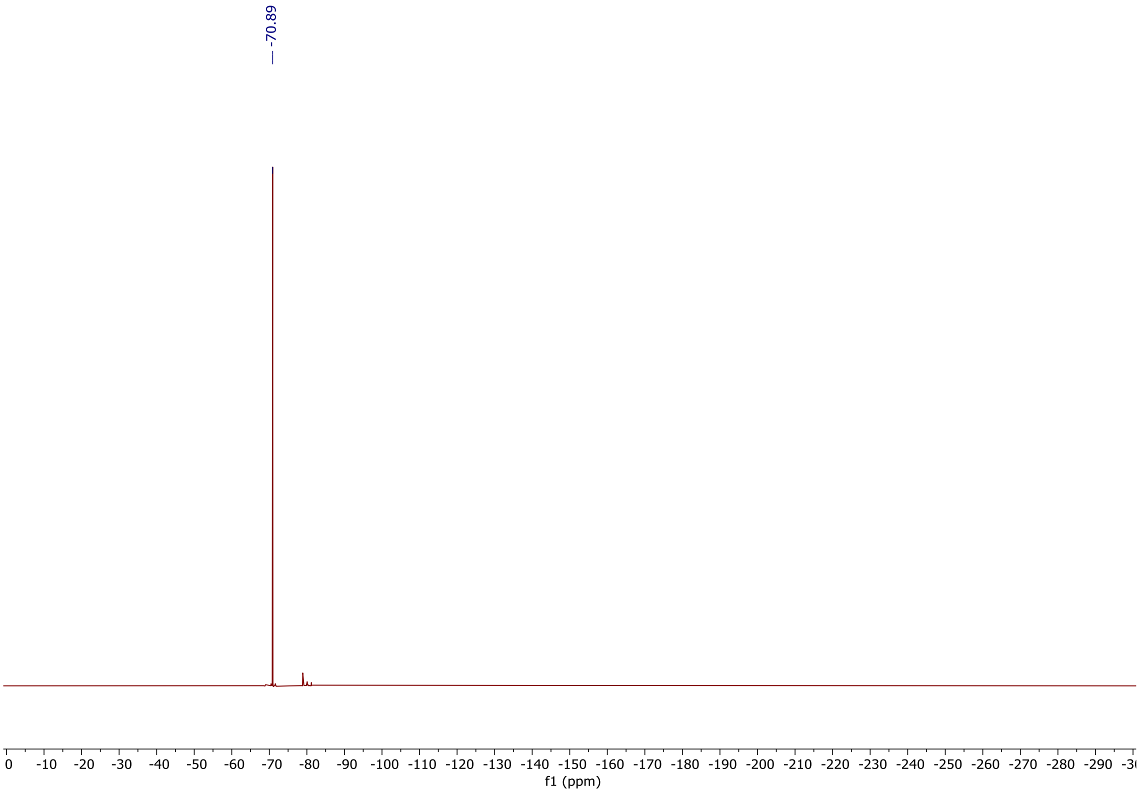


$^{11}B$ NMR of compound **3j** (160 MHz, acetone-$d_6$, 25ºC)

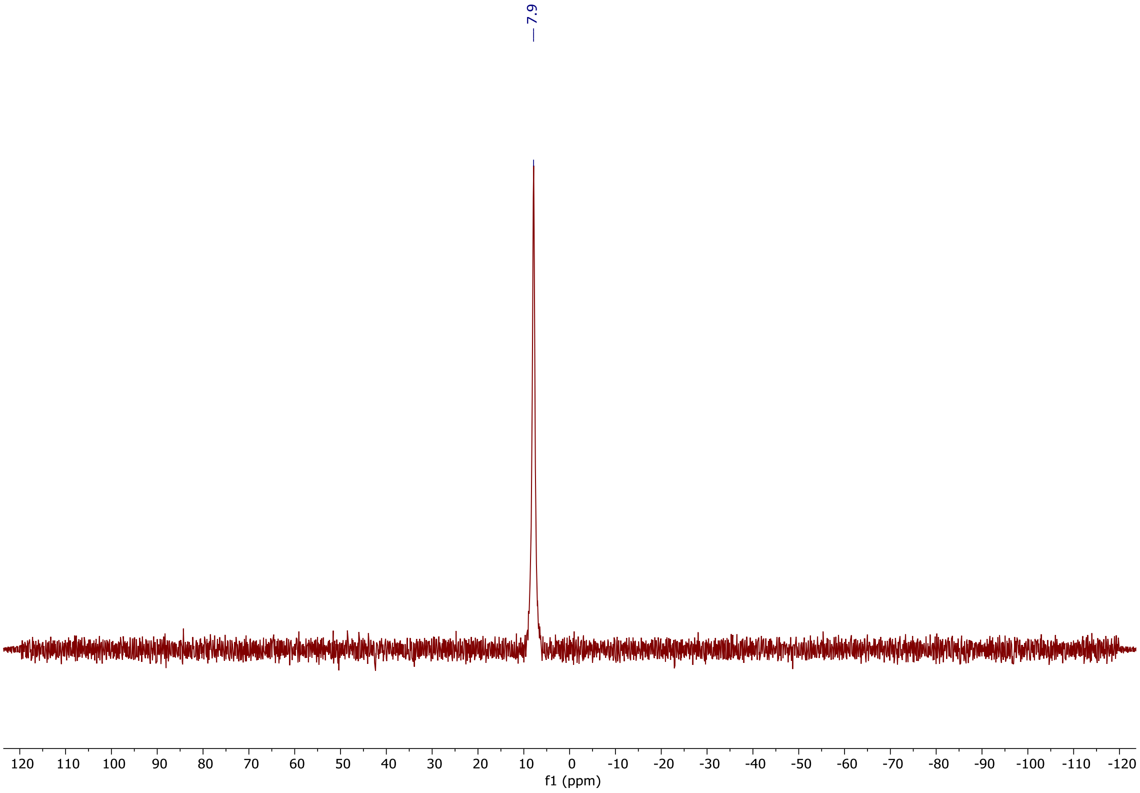

NOE NMR of compound **3j** (300 MHz, acetone-$d_6$, 25ºC): irradiation = 8.23 ppm

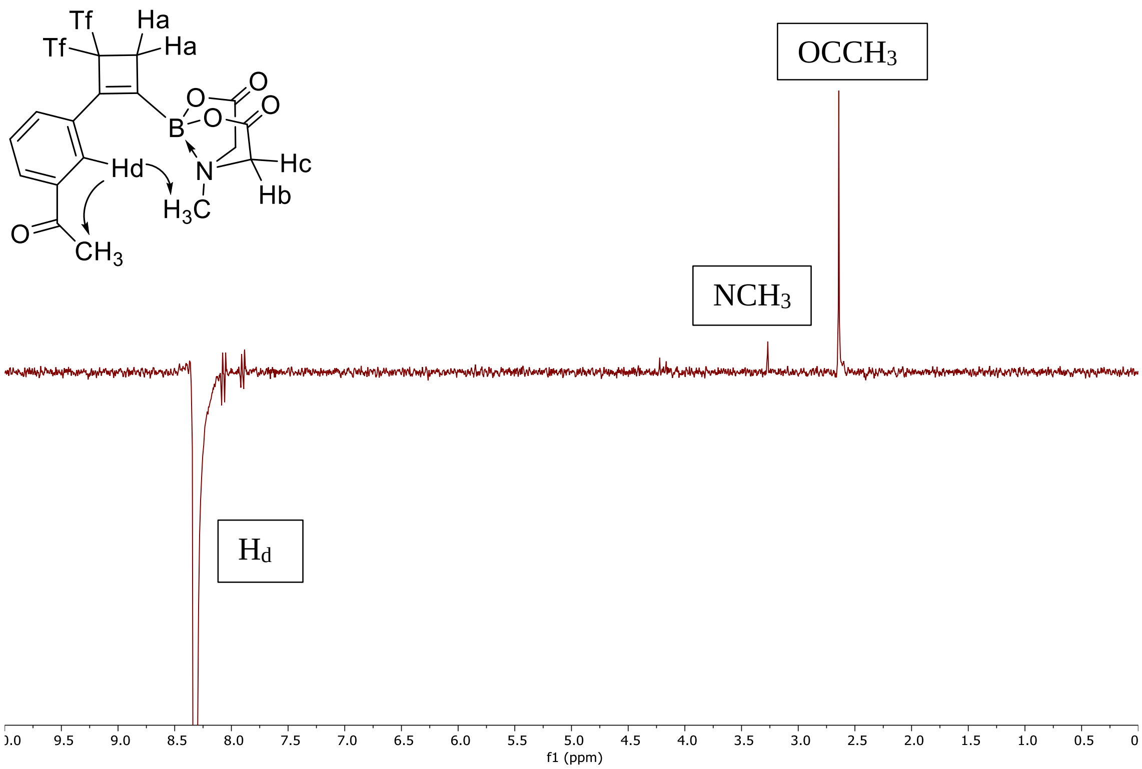


NOE NMR of compound **3j** (300 MHz, acetone-$d_6$, 25ºC): irradiation = 3.27 ppm

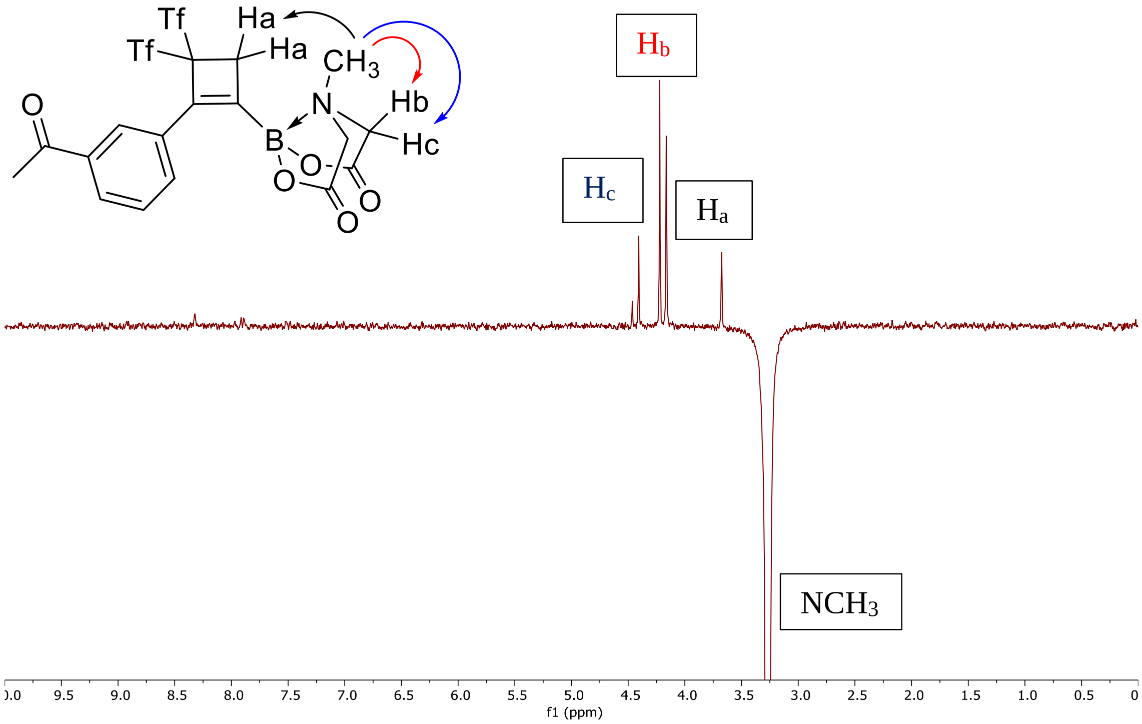

NOE NMR of compound **3j** (300 MHz, acetone-d$_6$, 25ºC): irradiation = 4.44 ppm

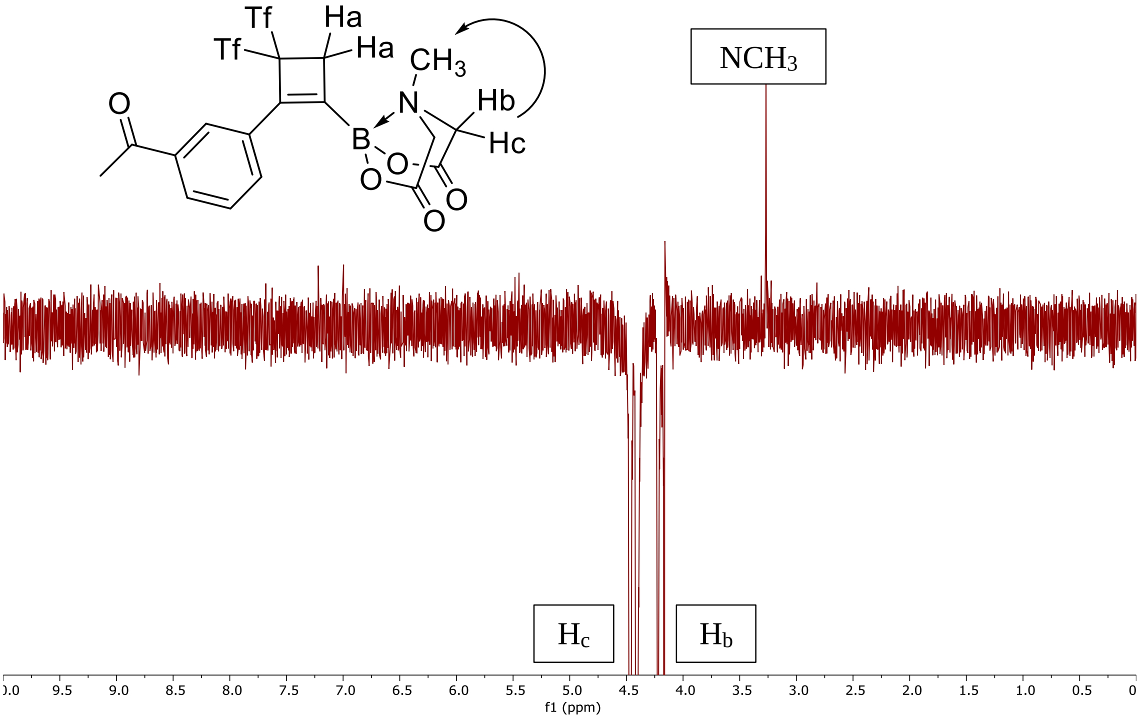


NOE NMR of compound **3j** (300 MHz, acetone-d$_6$, 25ºC): irradiation = 3.67 ppm

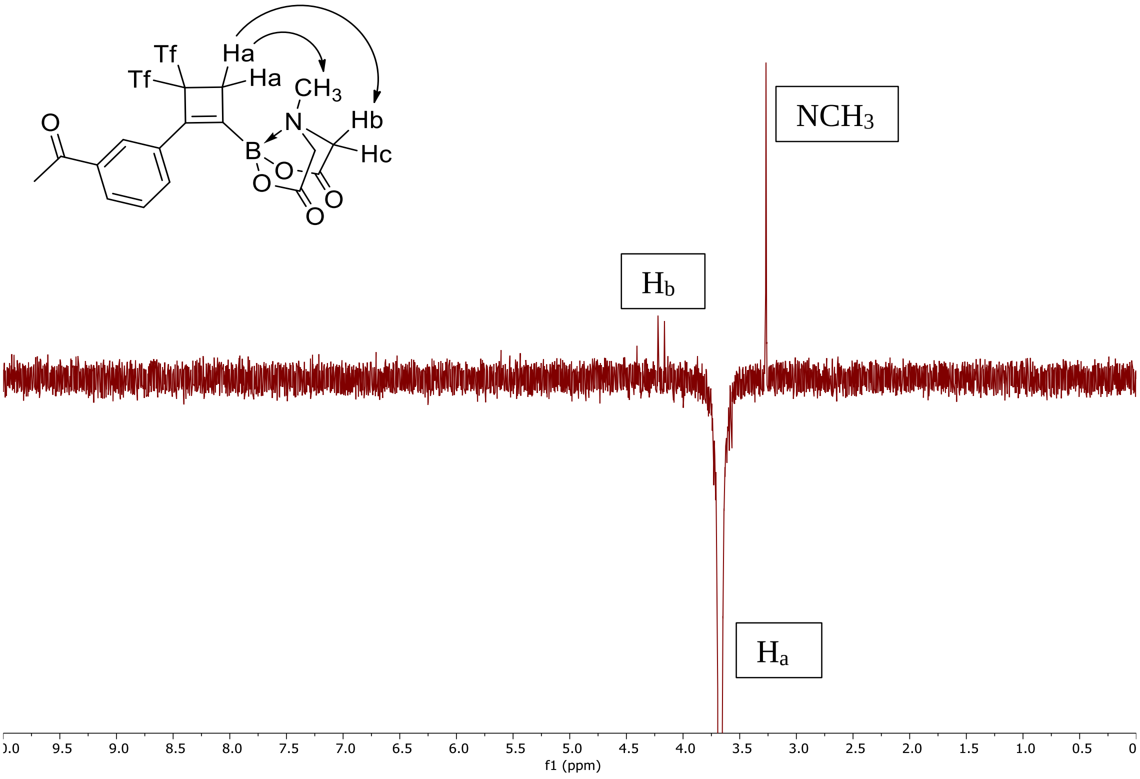

$^1H$-$^1H$ COSY NMR of compound **3j** (300 MHz, acetone-d$_6$, 25ºC)

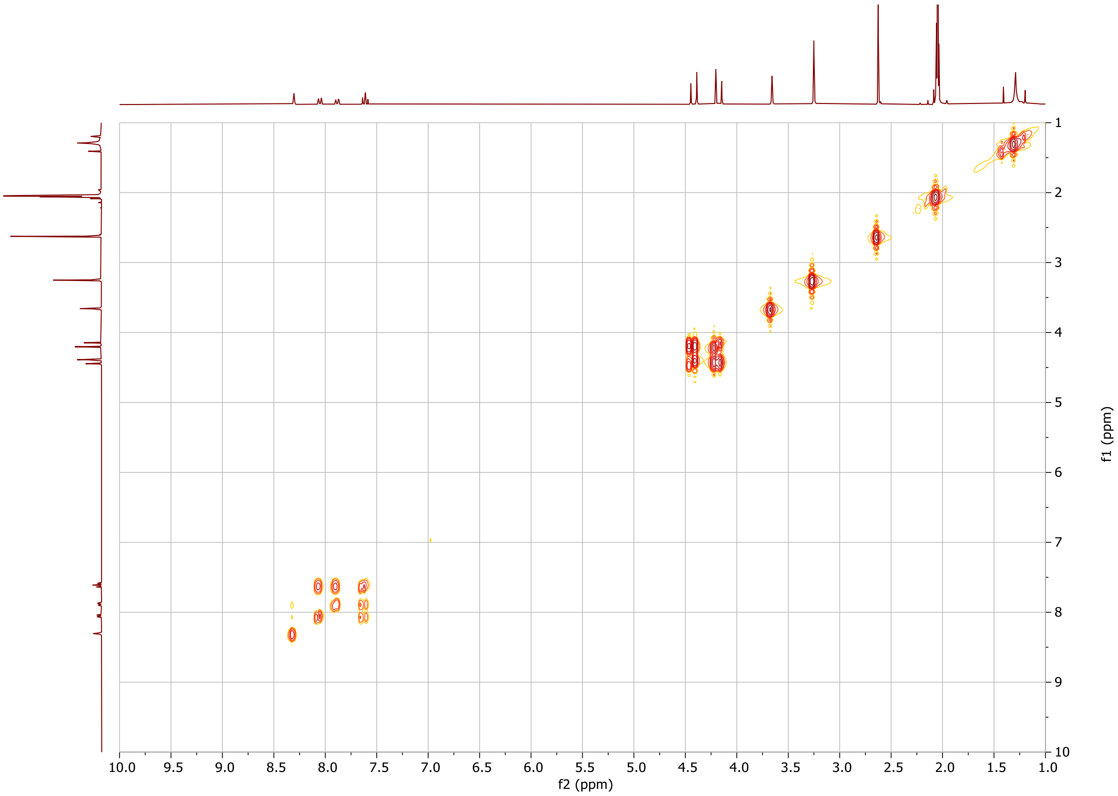

$^{1}$H NMR of compound **3k** (300 MHz, acetone-$d_6$, 25ºC)

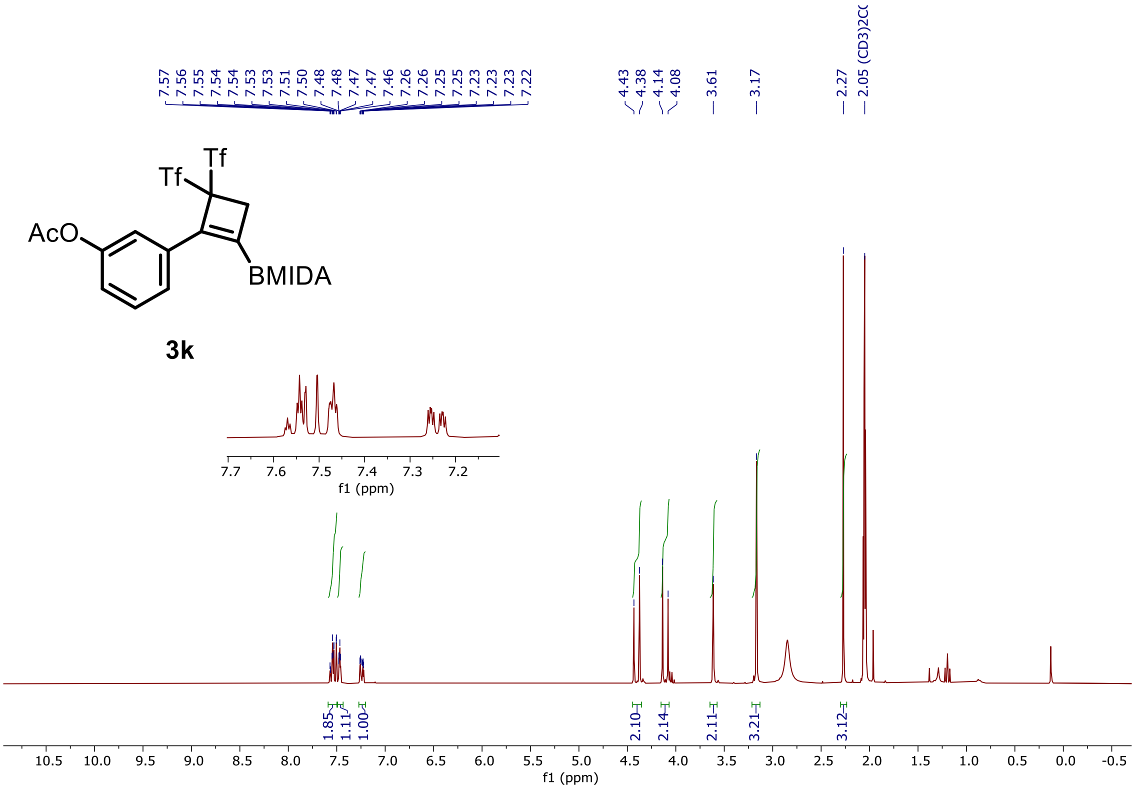


$^{13}$C NMR of compound **3k** (75 MHz, acetone-$d_6$, 25ºC)

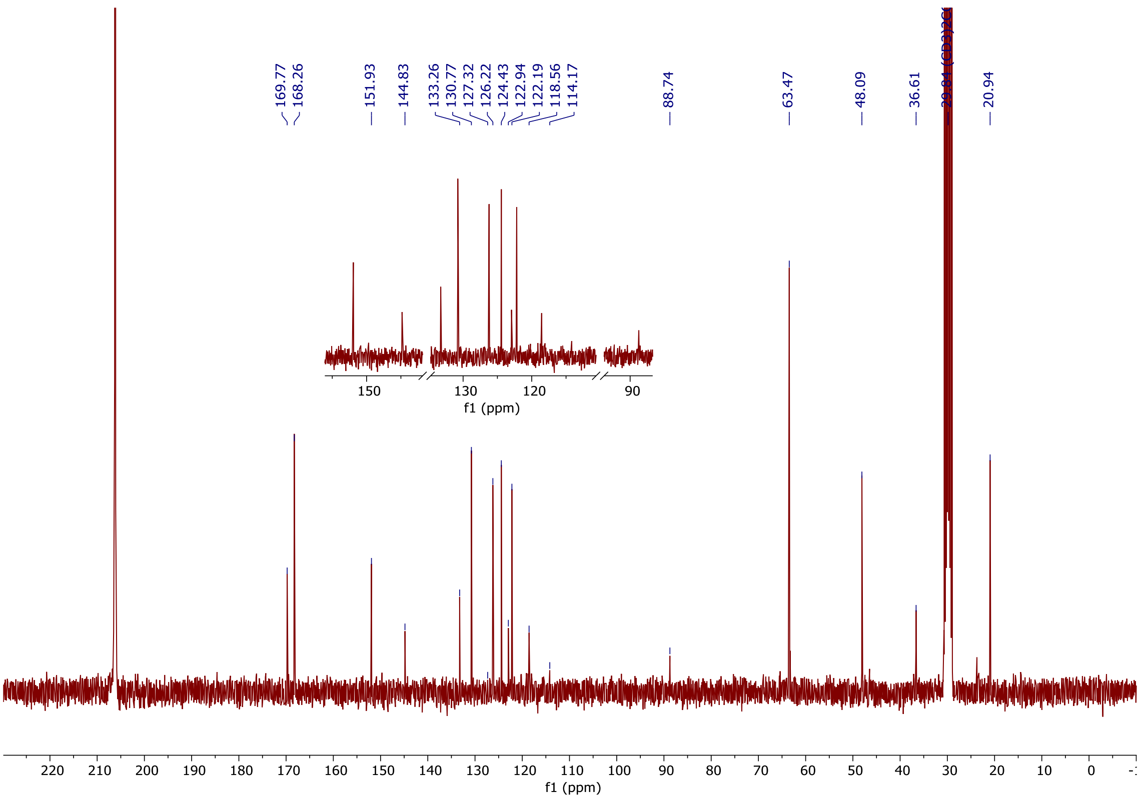

$^{19}$F NMR of compound **3k** (282 MHz, acetone-$d_6$, 25ºC)

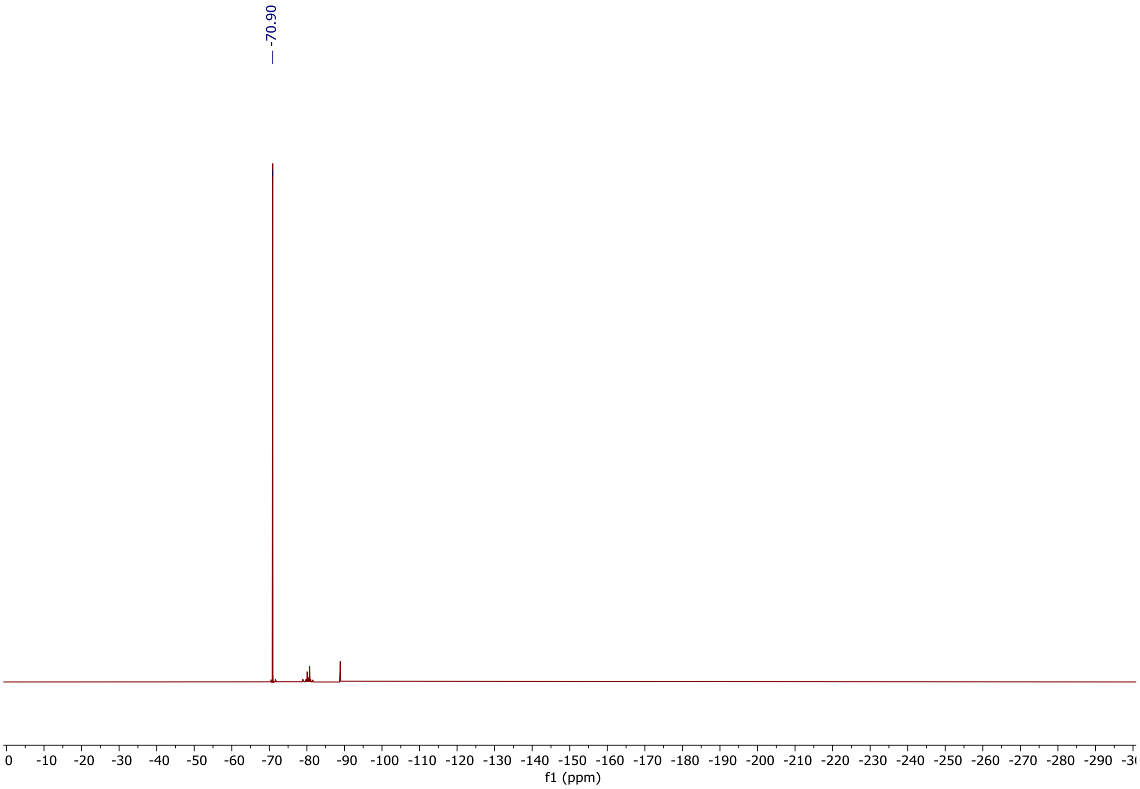

$^{1}H$ NMR of compound **3l** (700 MHz, acetone-d$_6$, 25ºC)

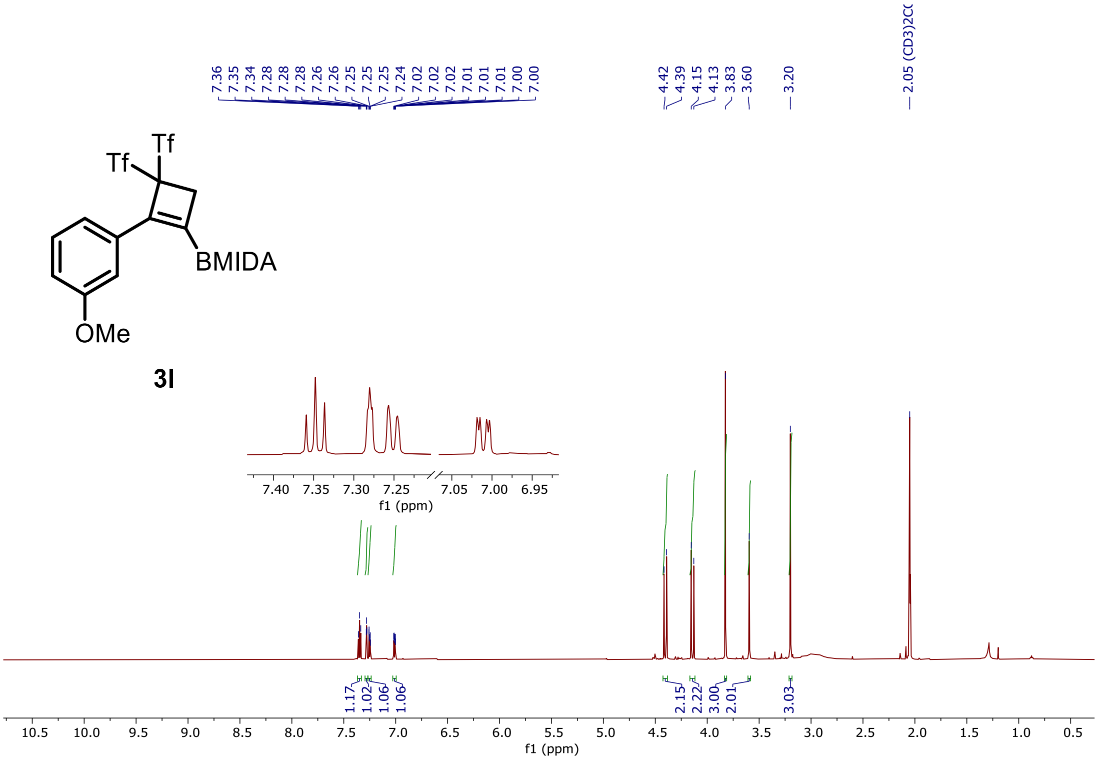


$^{13}C$ NMR of compound **3l** (176 MHz, acetone-d$_6$, 25ºC)

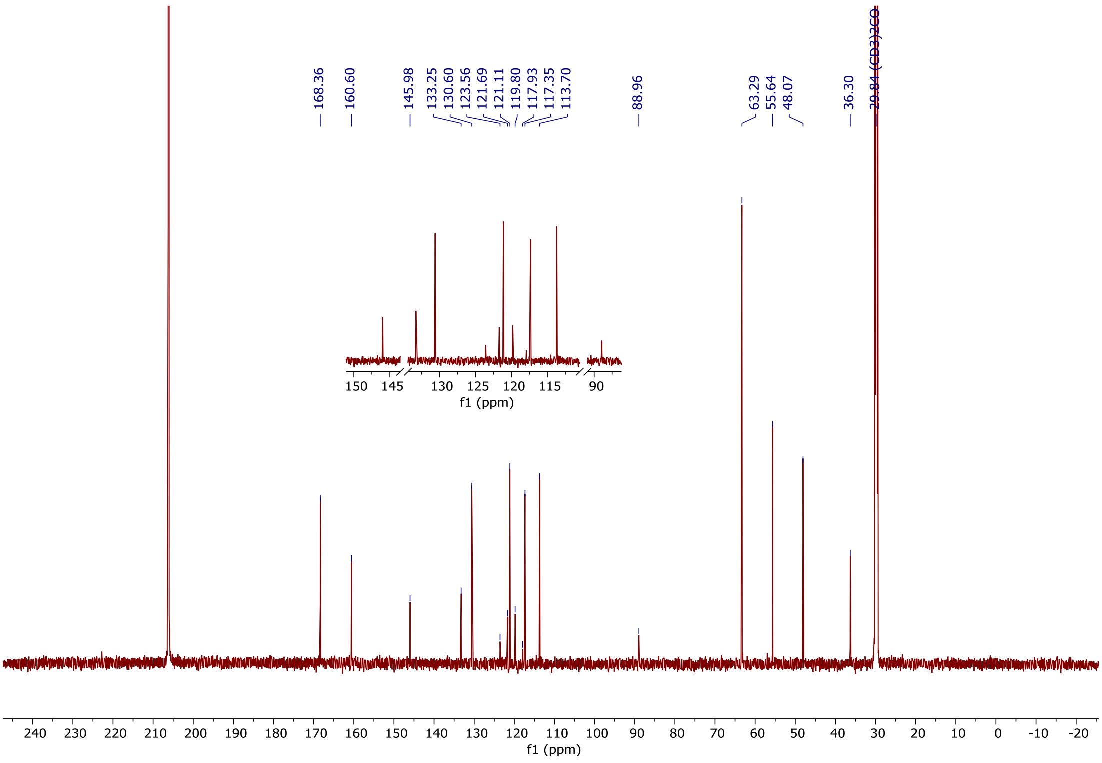

$^{19}F$ NMR of compound **3l** (282 MHz, acetone-d$_6$, 25ºC)

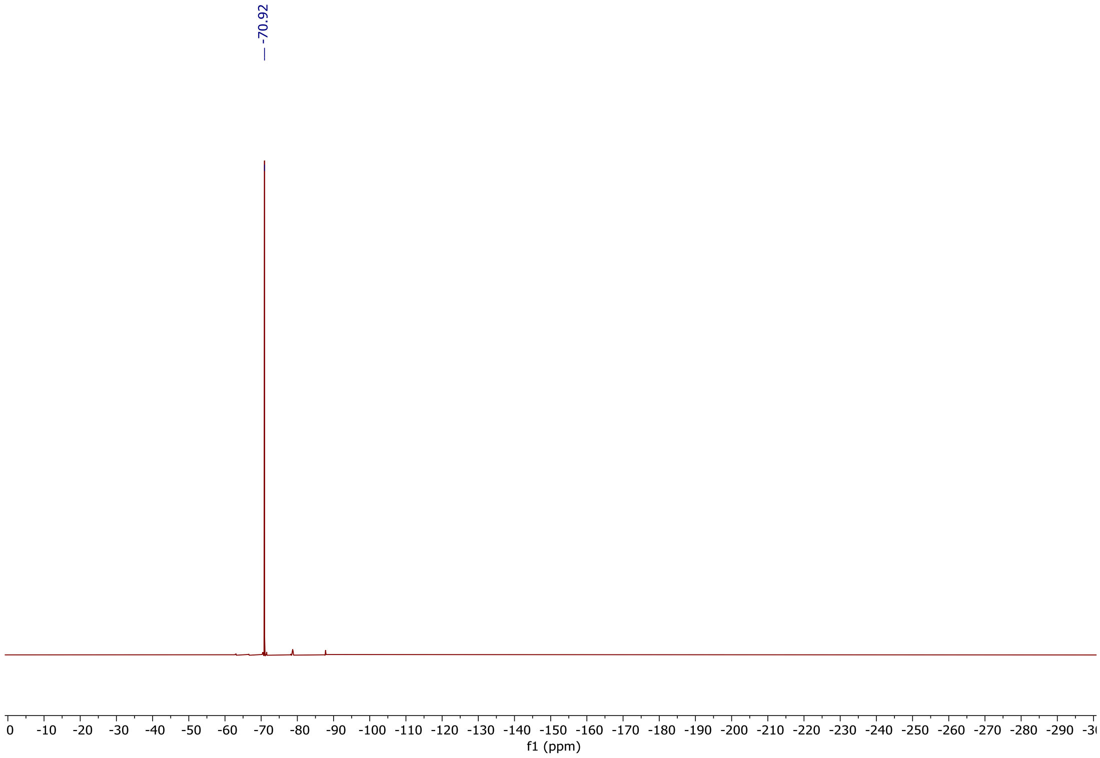

## $^{1}$H NMR of compound **3m** (700 MHz, acetonitrile-d$_3$, 25ºC)

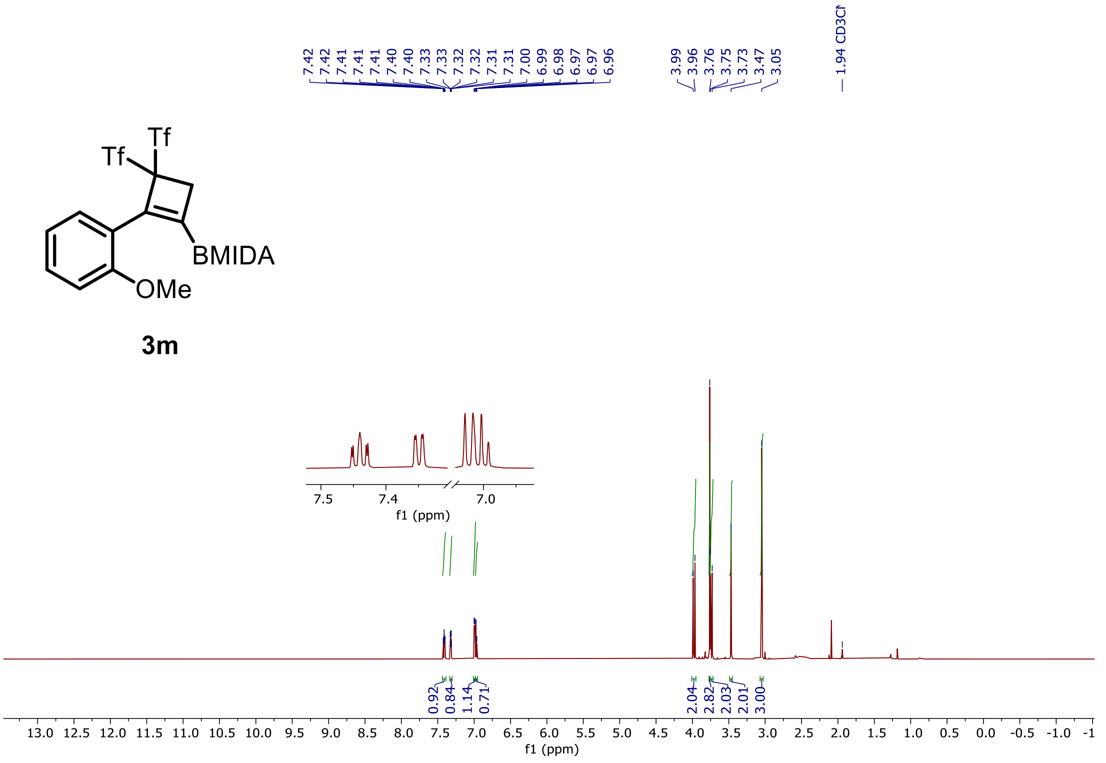


## $^{13}$C NMR of compound **3m** (176 MHz, acetonitrile-d$_3$, 25ºC)

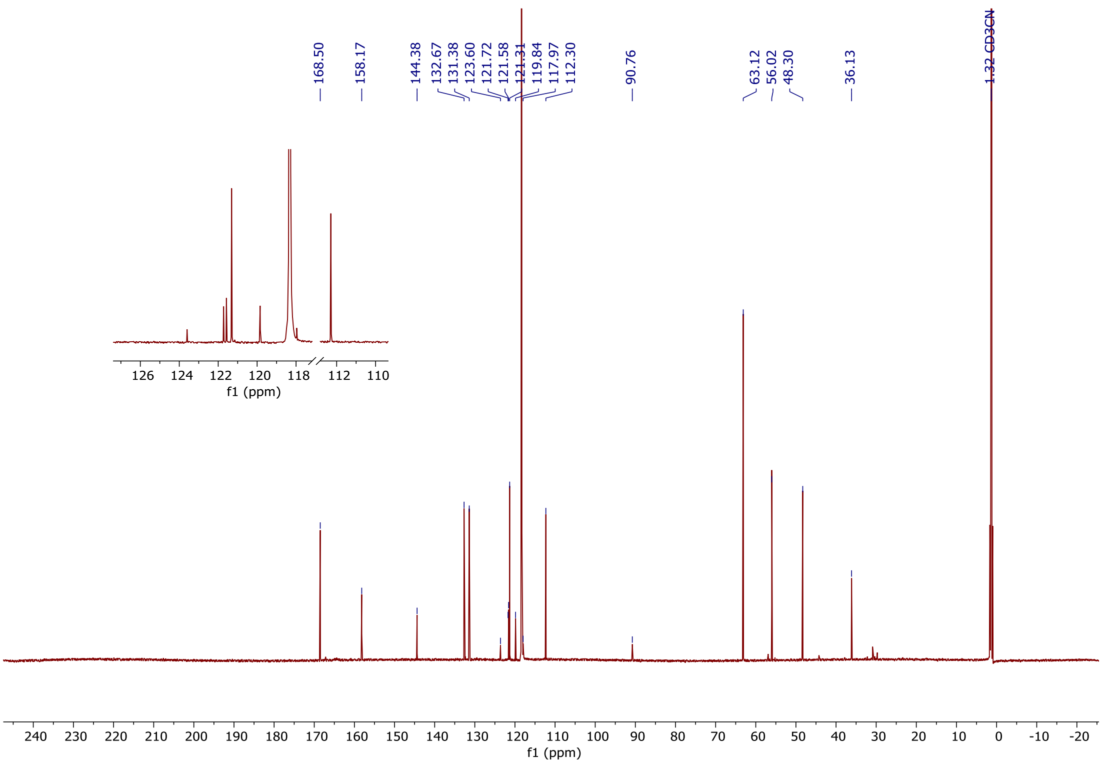

$^{19}$F NMR of compound **3m** (282 MHz, acetonitrile-d$_3$, 25ºC)

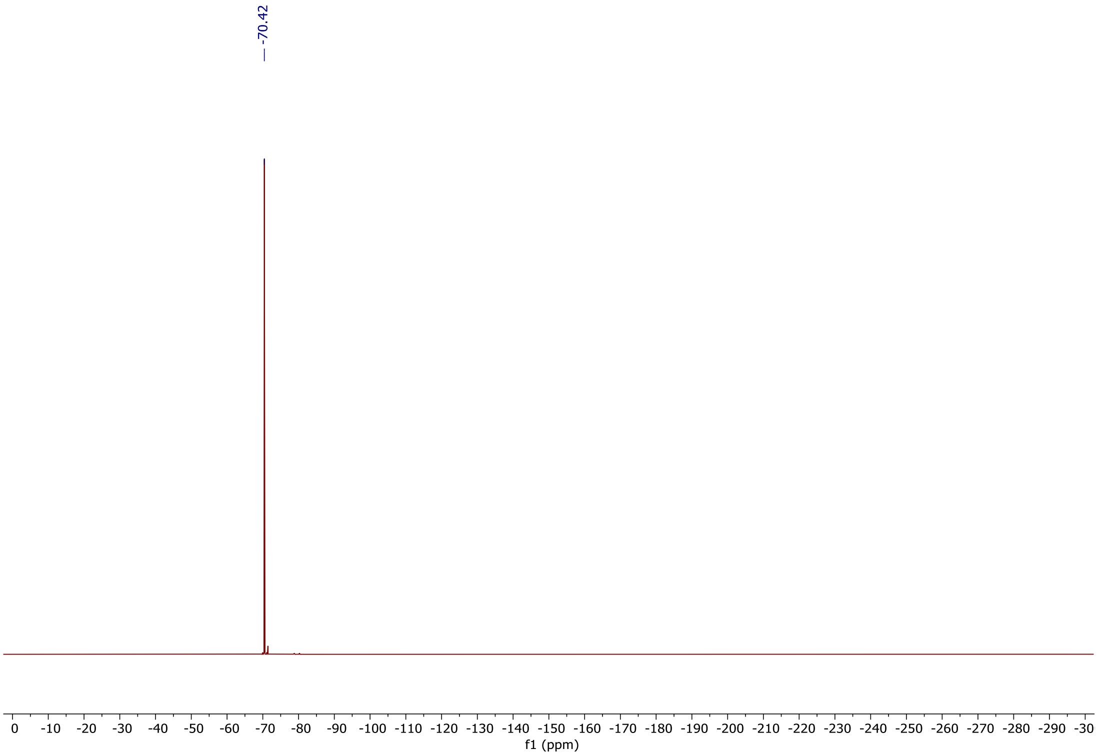


HSQC NMR of compound **3m** (176 MHz /700 MHz, acetonitrile-d$_3$, 25ºC)

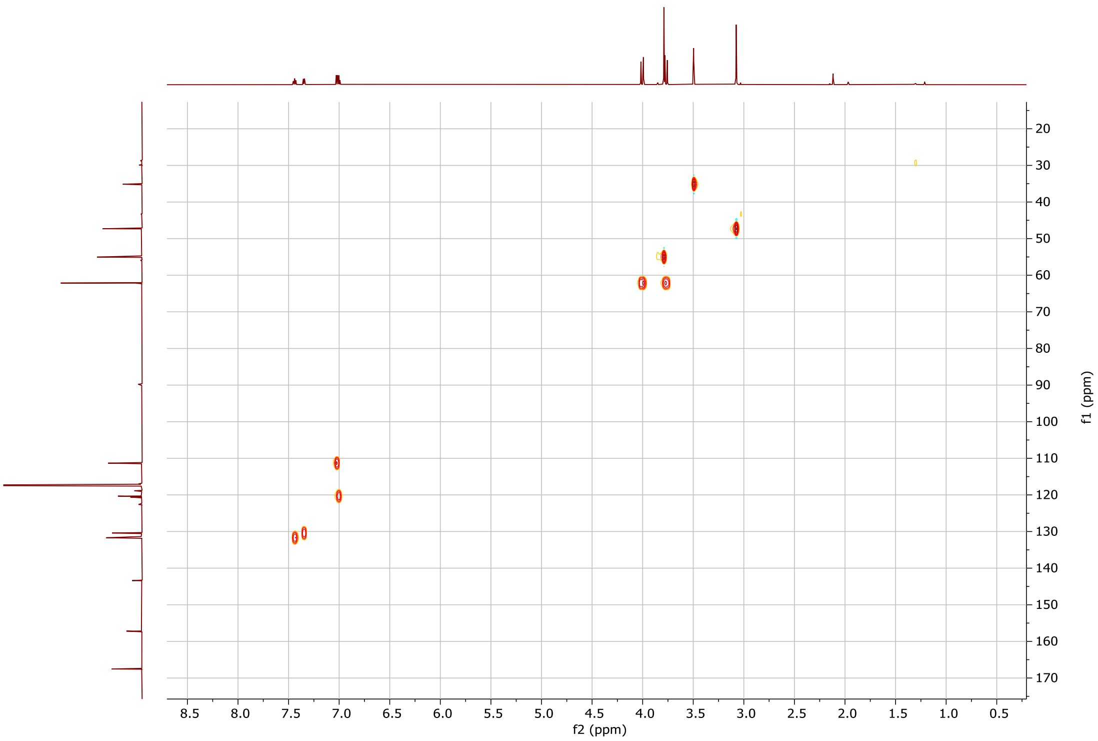

$^{1}H$ NMR of compound **3n** (700 MHz, acetone-$d_6$, 25ºC)

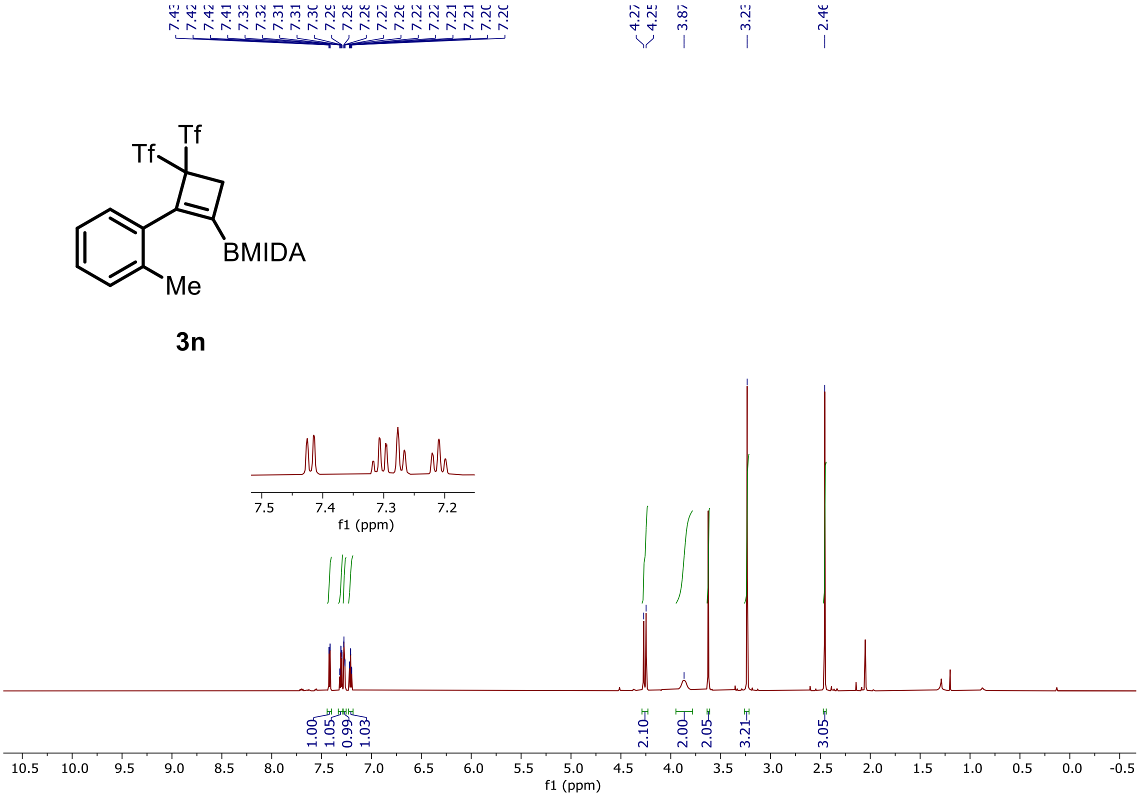


$^{13}C$ NMR of compound **3n** (176 MHz, acetone-$d_6$, 25ºC)

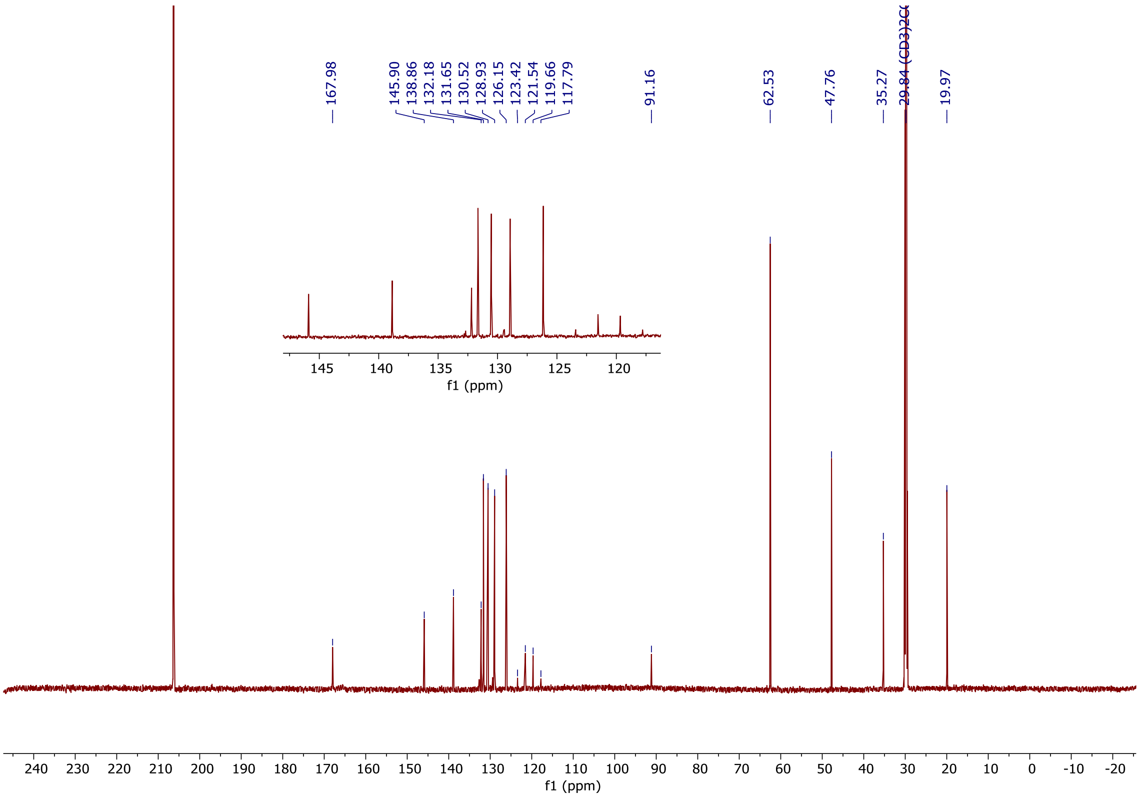

$^{19}F$ NMR of compound **3n** (282 MHz, acetone-$d_6$, 25ºC)

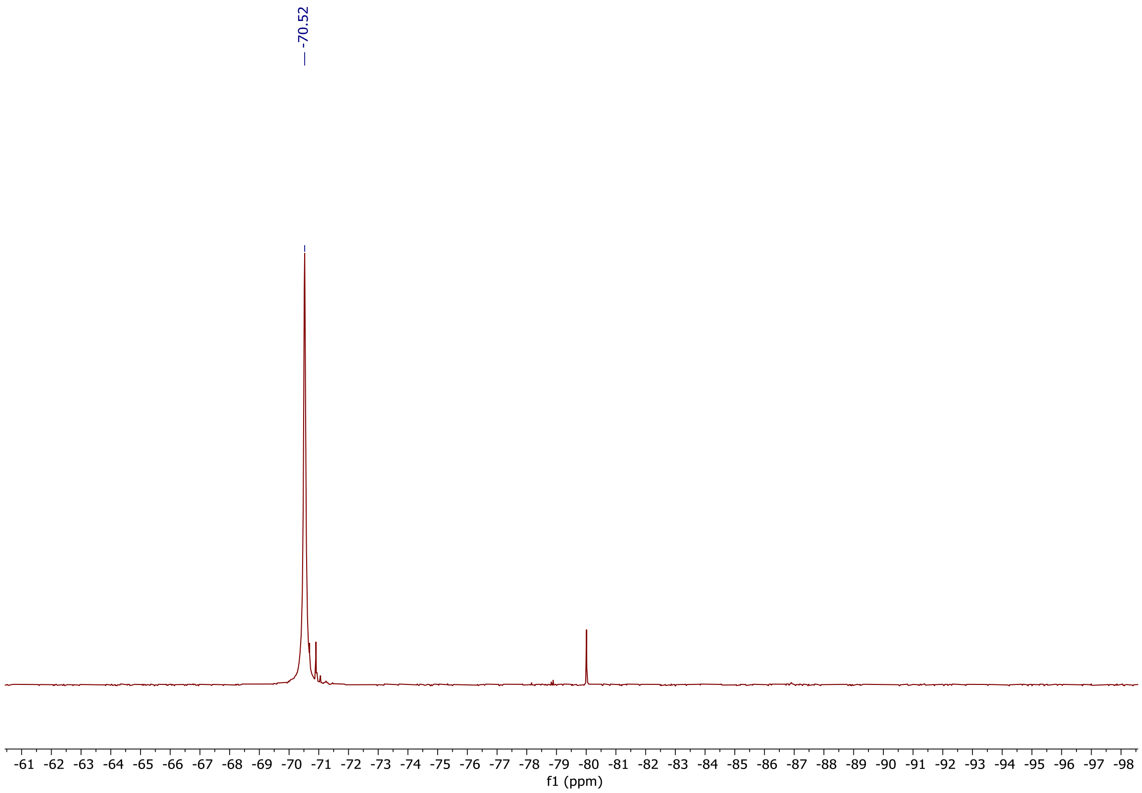

$^{1}$H NMR of compound **3o** (300 MHz, acetone-$d_6$, 25ºC)

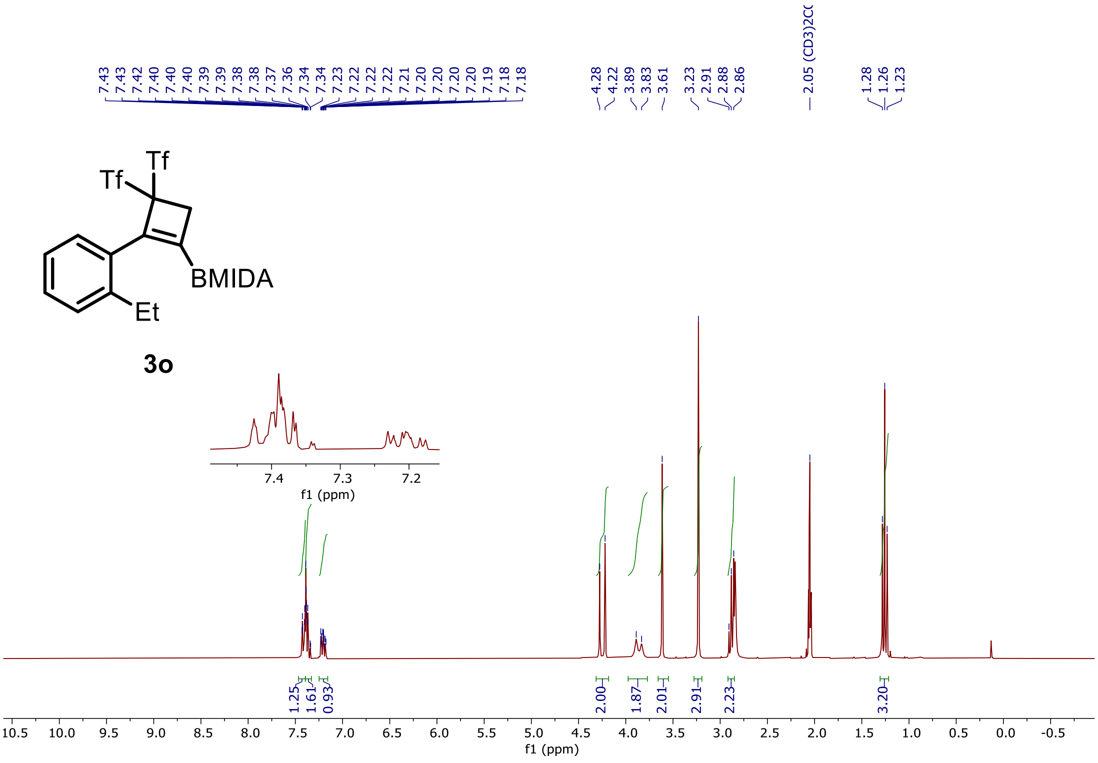


$^{13}$C NMR of compound **3o** (75 MHz, acetone-$d_6$, 25ºC)

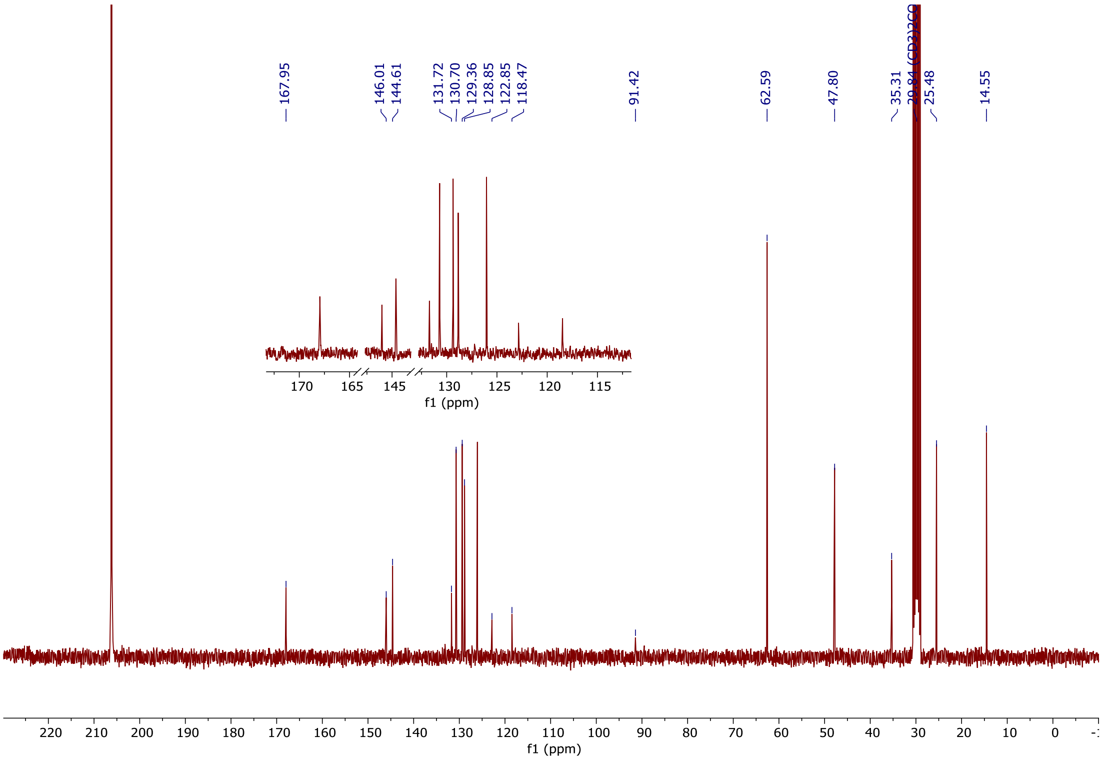

$^{19}F$ NMR of compound **3o** (282 MHz, acetone-$d_6$, 25ºC)

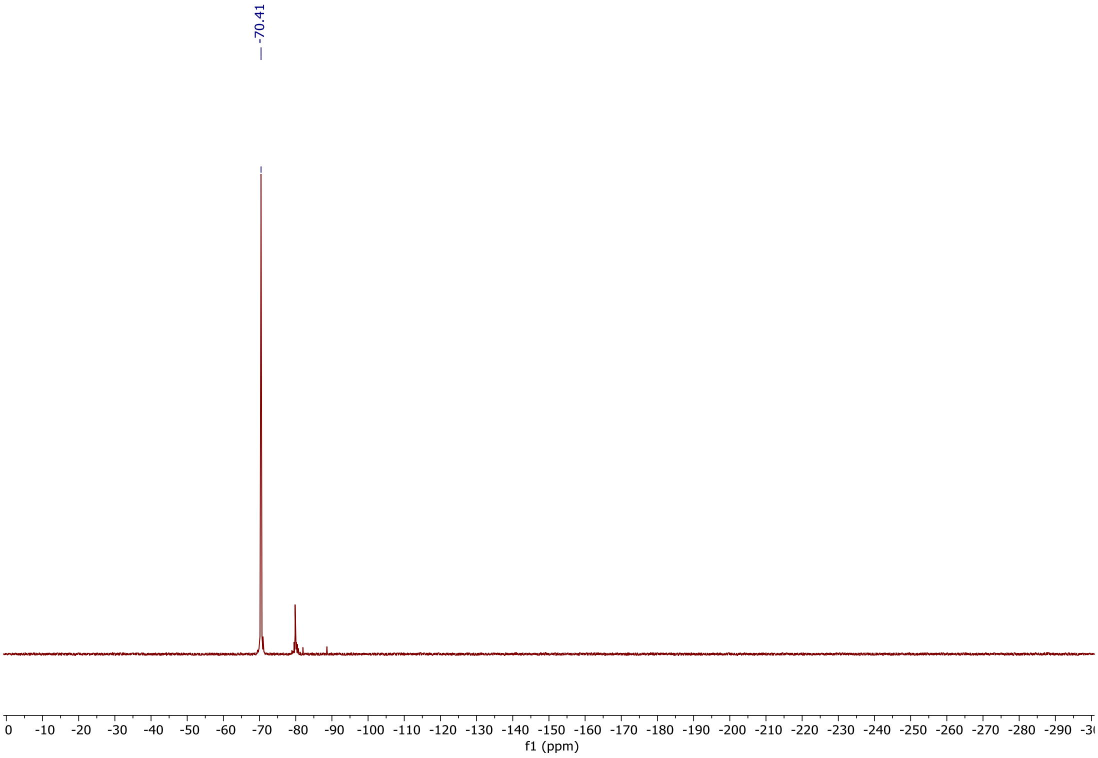

$^{1}$H NMR of compound **3p** (300 MHz, acetone-$d_6$, 25ºC)

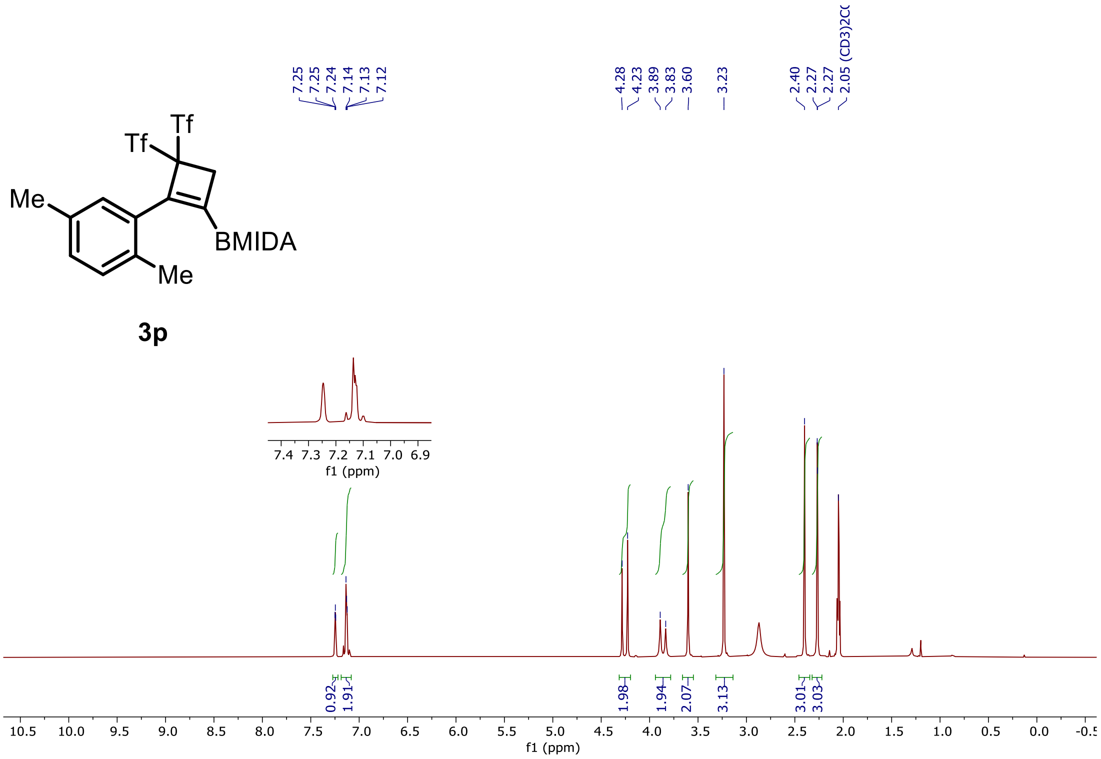


$^{13}$C NMR of compound **3p** (176 MHz, acetone-$d_6$, 25ºC)

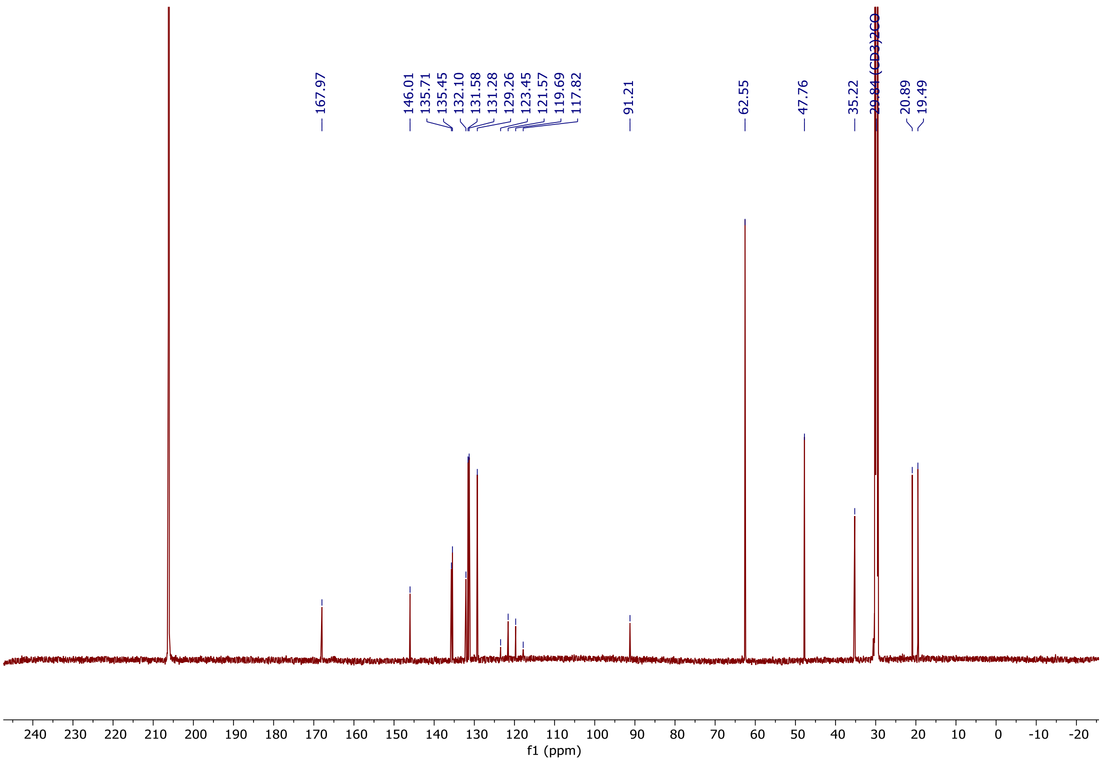

$^{19}F$ NMR of compound **3p** (282 MHz, acetone-d$_6$, 25ºC)

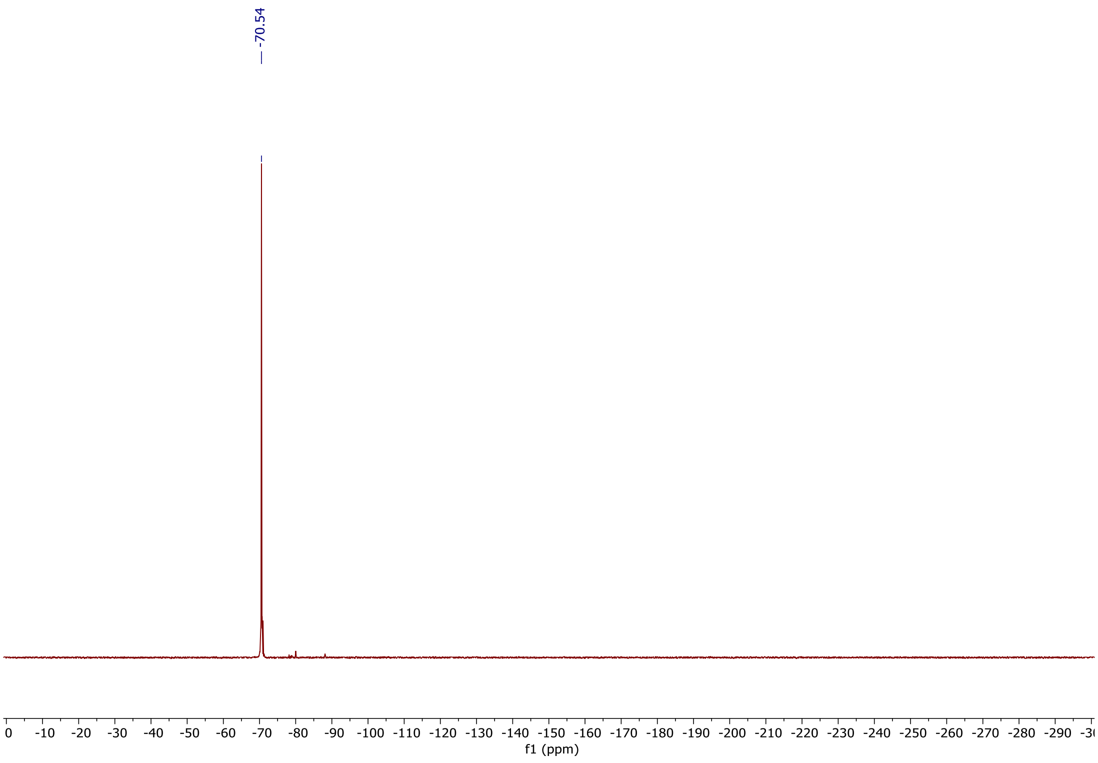


$^{1}H$-$^{13}C$ HSQC NMR of compound **3p** (176 MHz /700 MHz, acetone-d$_6$, 25ºC)

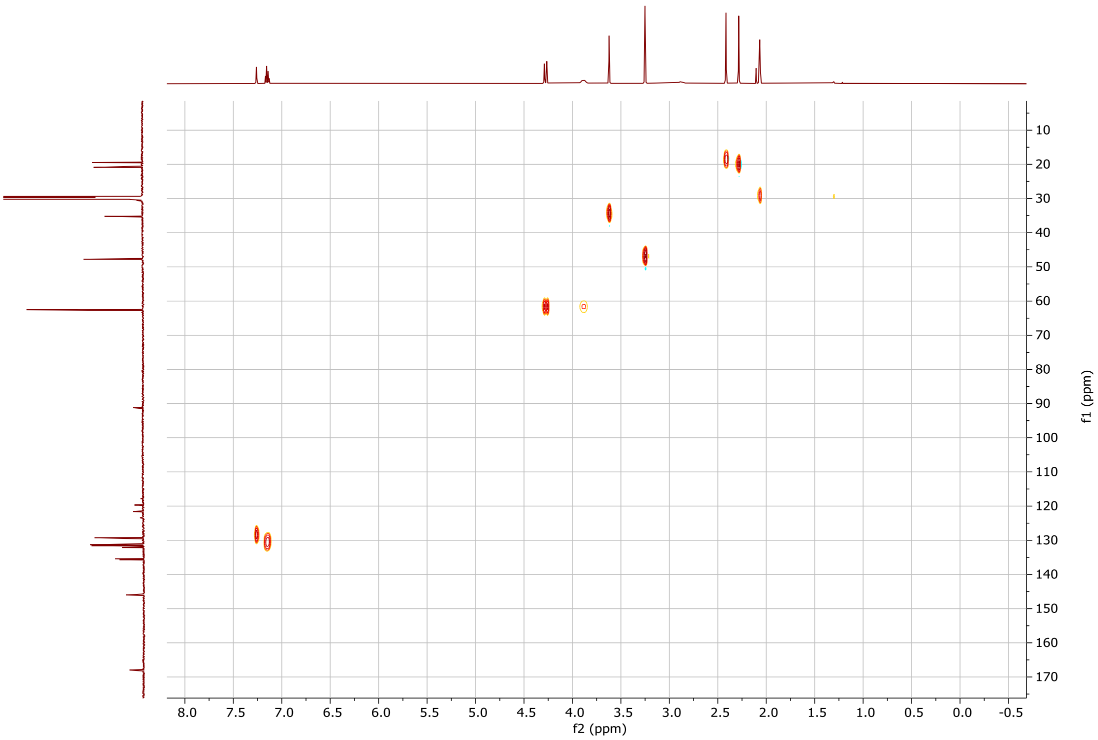

$^{1}H$-$^{13}C$ HMBC NMR of compound **3p** (176 MHz /700 MHz, acetone-$d_6$, 25ºC)

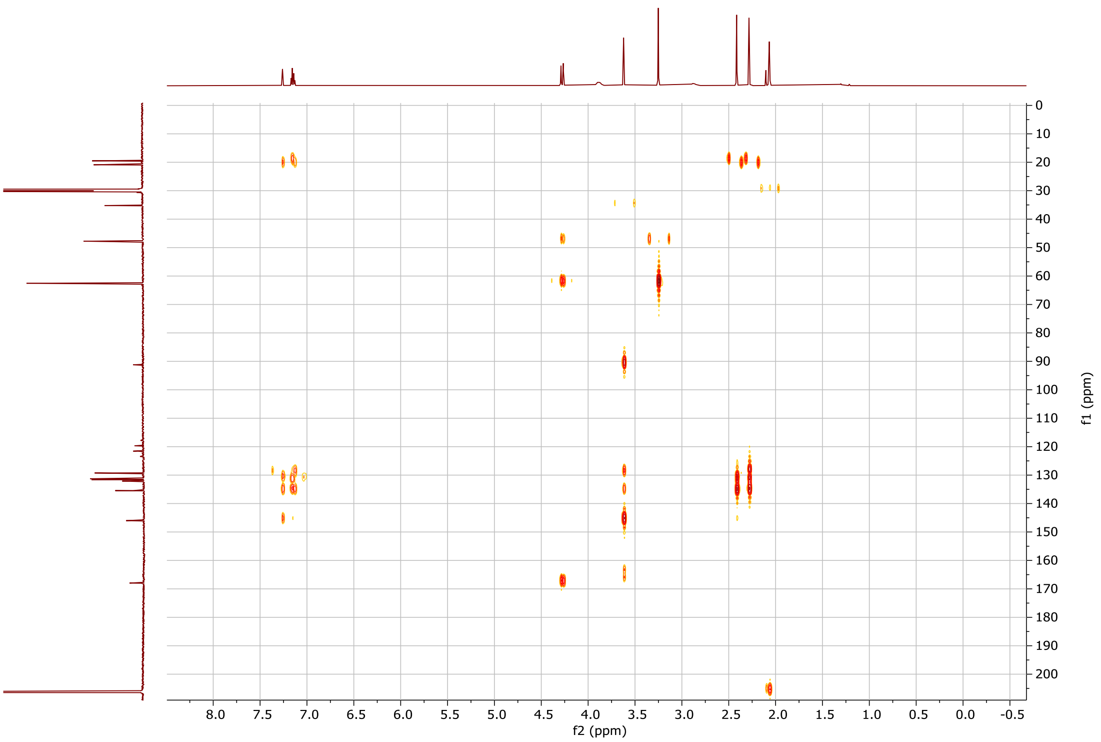

$^{1}$H NMR of compound **3q** (300 MHz, acetone-$d_6$, 25ºC)

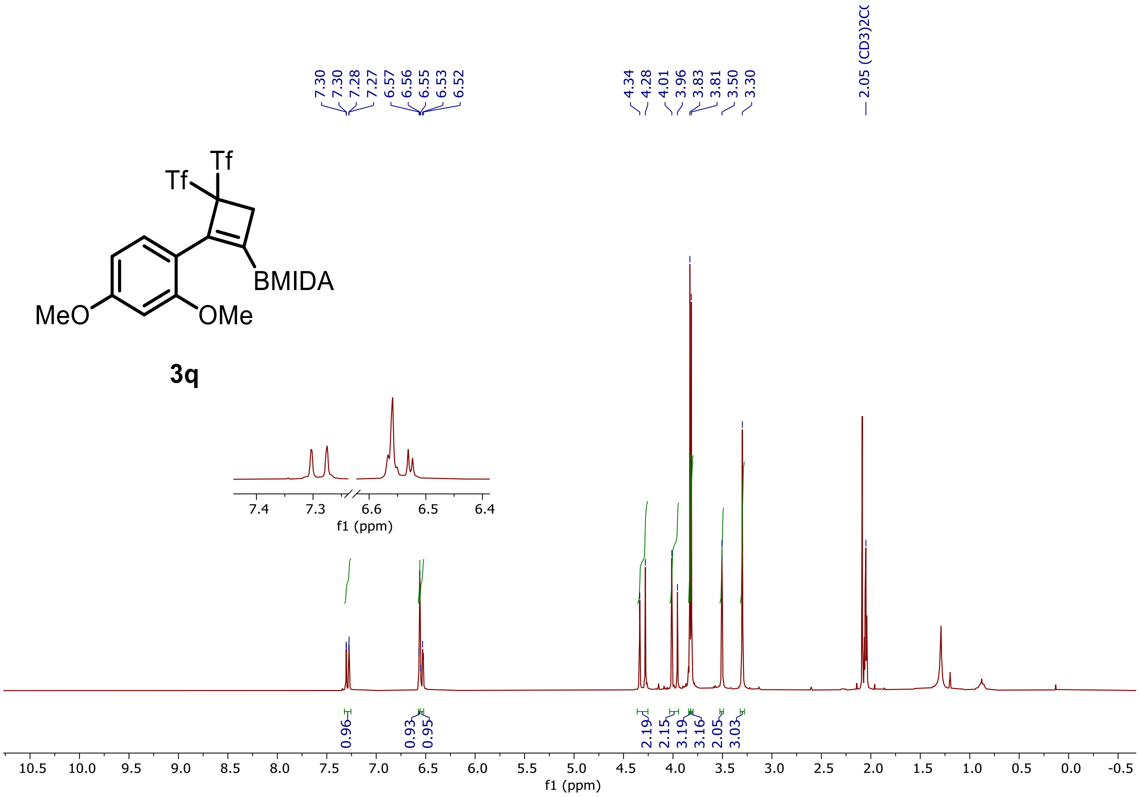


$^{13}$C NMR of compound **3q** (75 MHz, acetone-$d_6$, 25ºC)

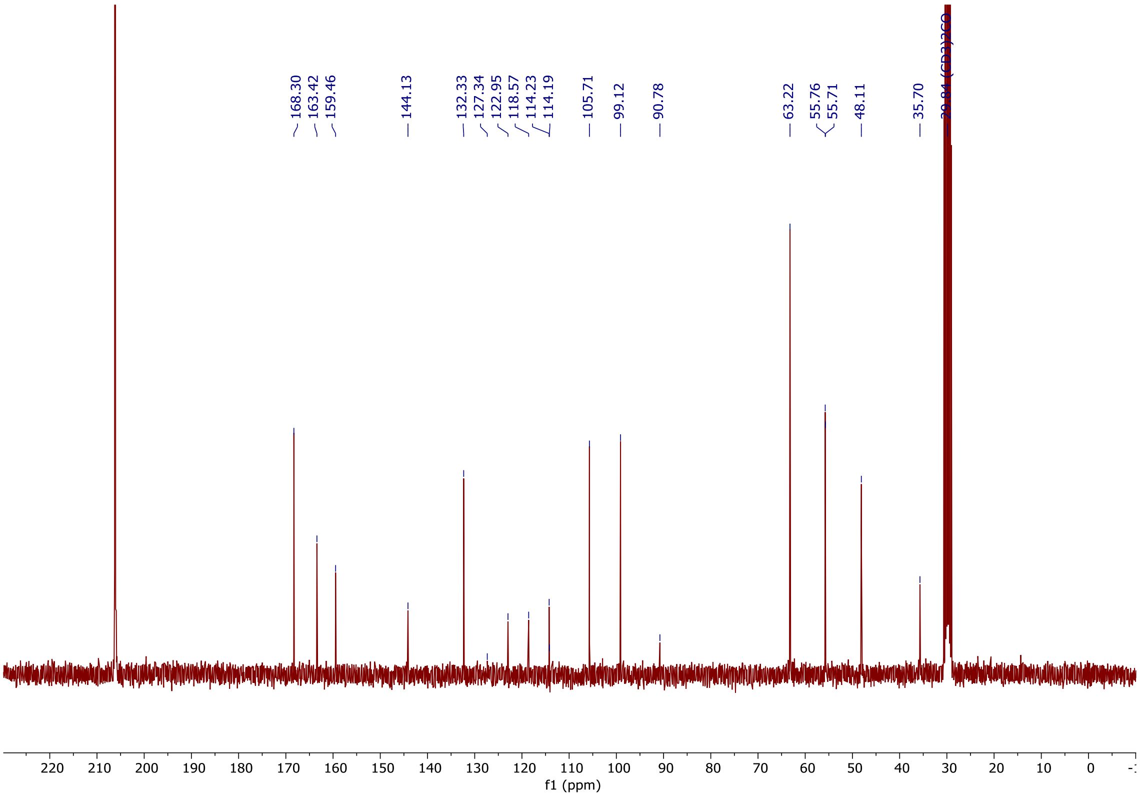

$^{19}F$ NMR of compound **3q** (282 MHz, acetone-$d_6$, 25ºC)

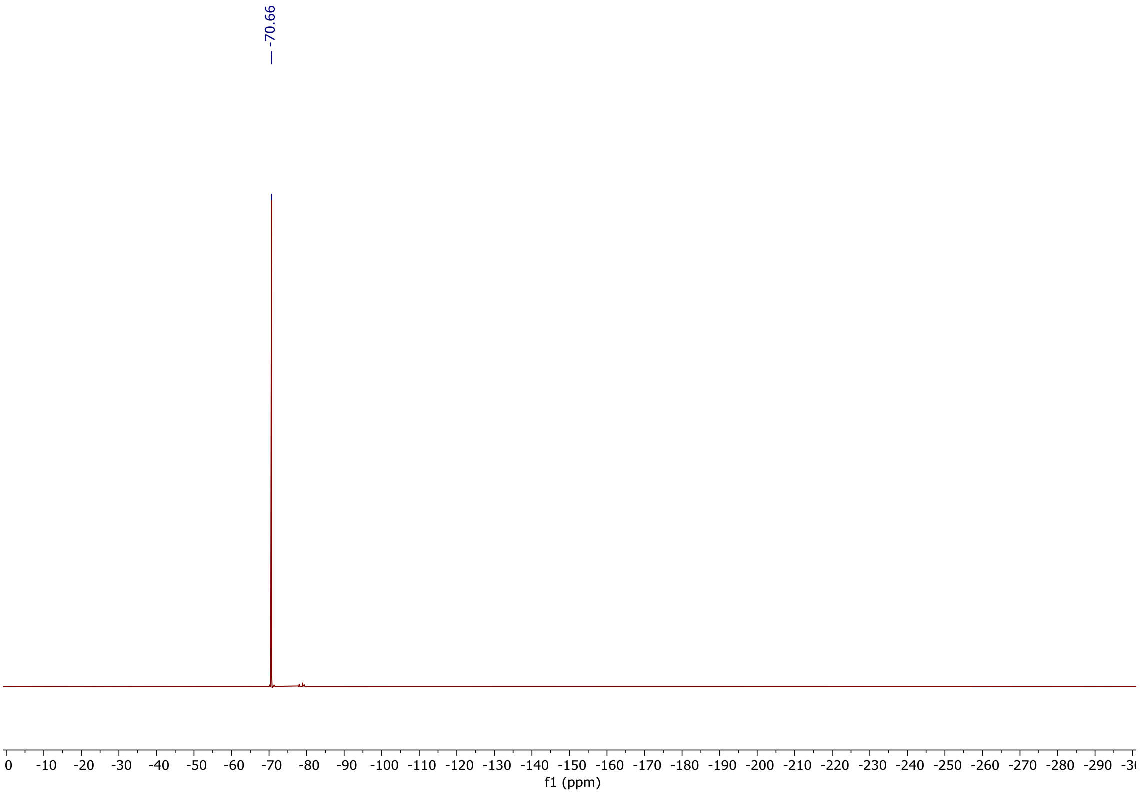

$^{1}H$ NMR of compound **3r** (300 MHz, acetone-$d_6$, 25ºC)

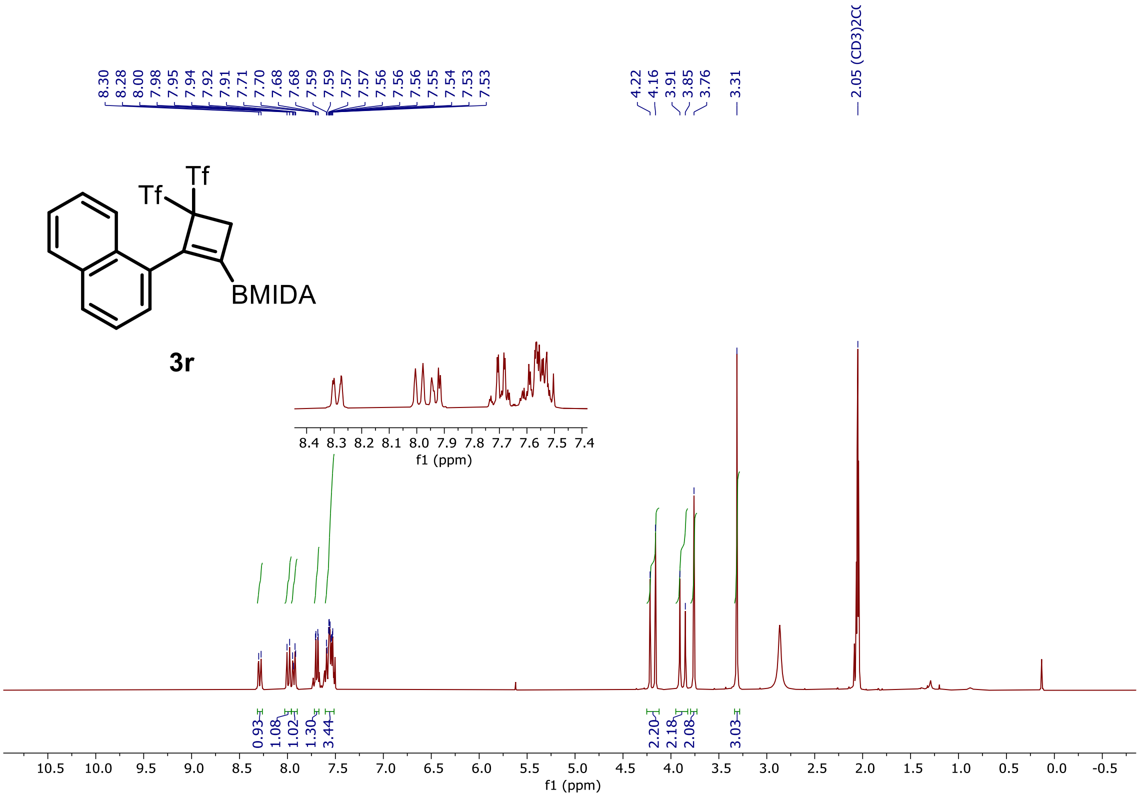

**3r**

$^{13}C$ NMR of compound **3r** (75 MHz, acetone-$d_6$, 25ºC)

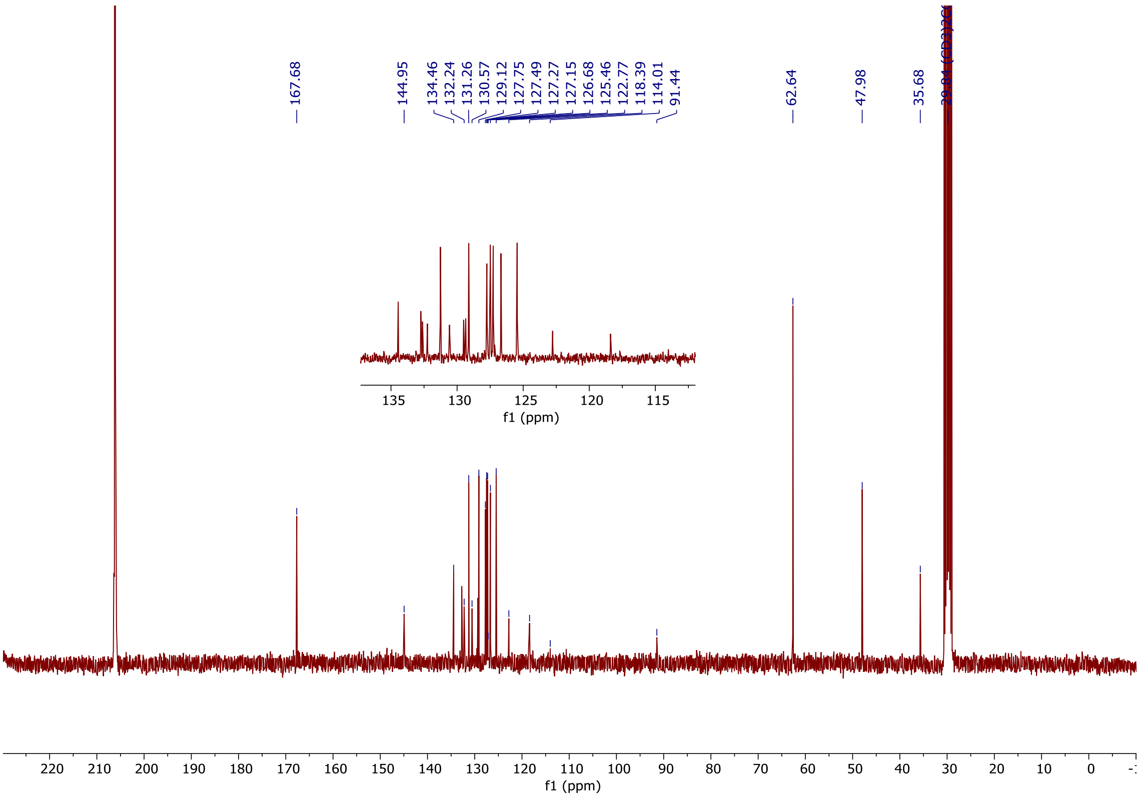

$^{19}$F NMR of compound **3r** (282 MHz, acetone-$d_6$, 25ºC)

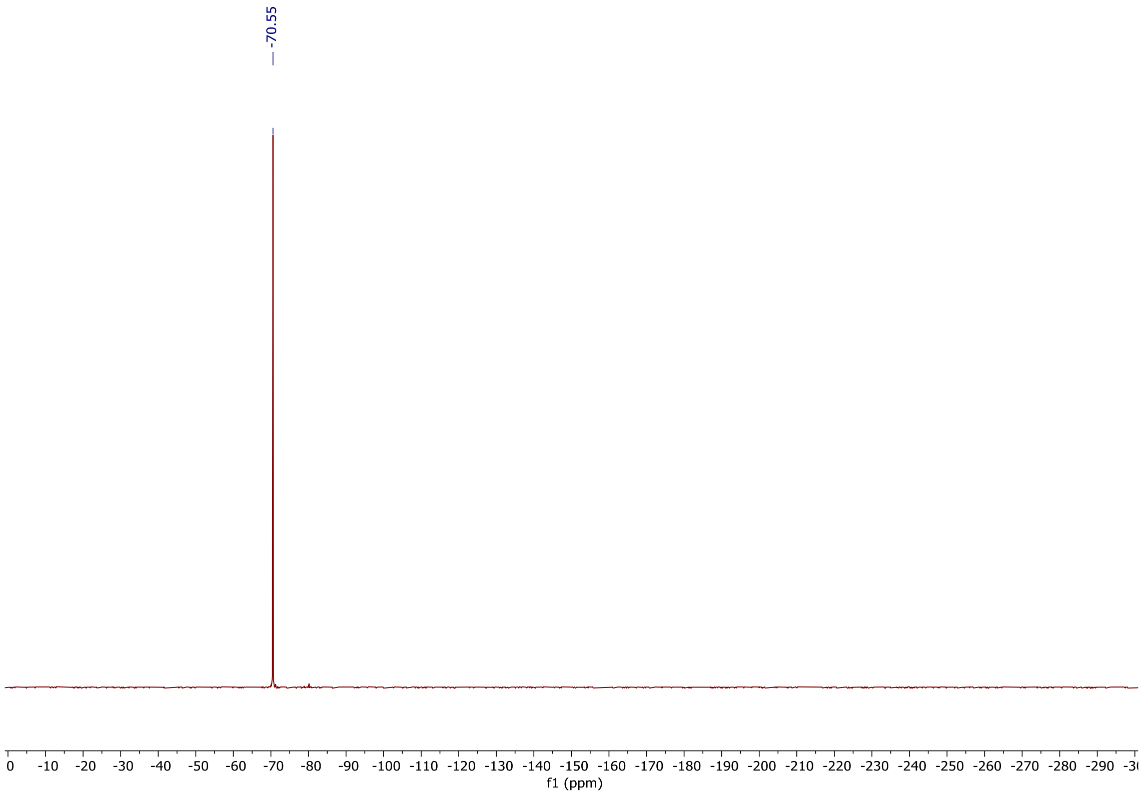

$^{1}$H NMR of compound **3s** (700 MHz, acetone-$d_6$, 25ºC)

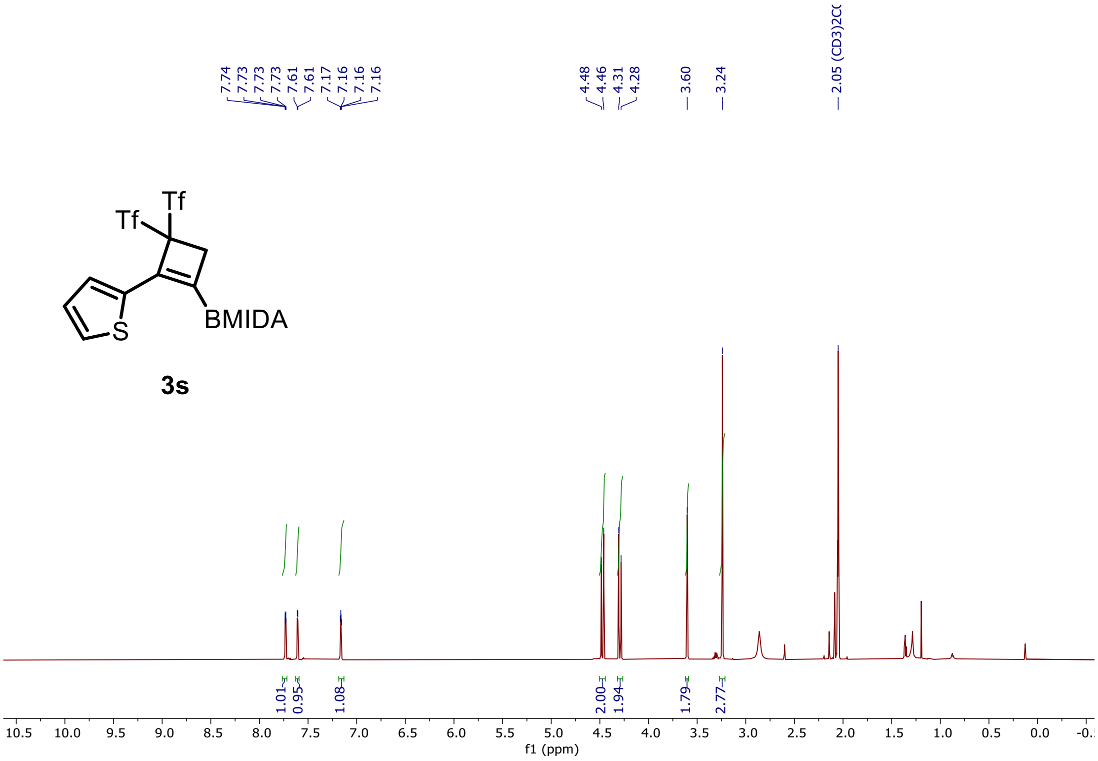


$^{13}$C NMR of compound **3s** (176 MHz, acetone-$d_6$, 25ºC)

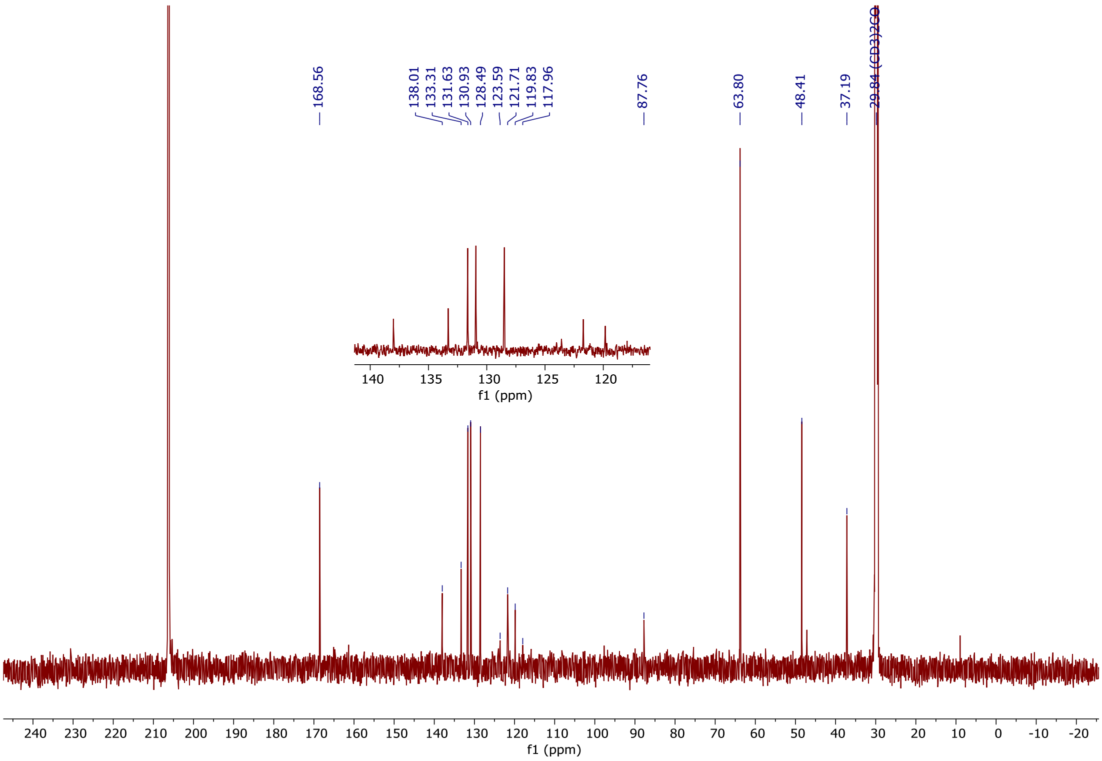

$^{19}$F NMR of compound **3s** (282 MHz, acetone-$d_6$, 25ºC)

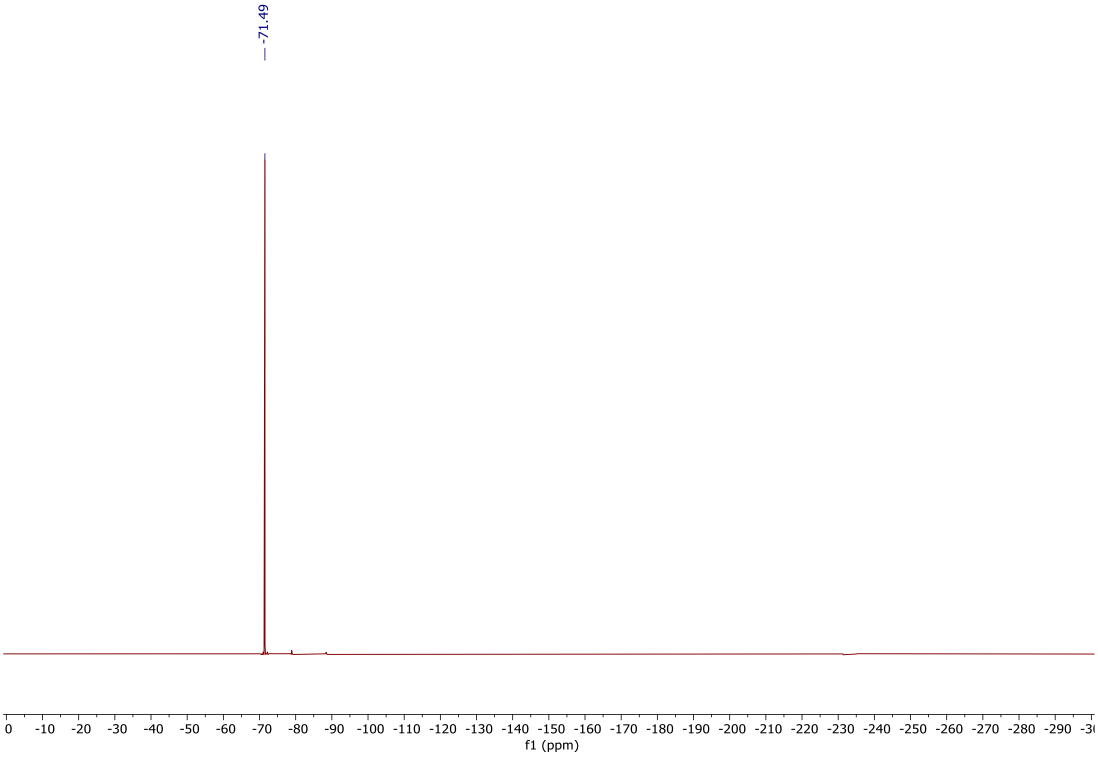

$^{1}$H NMR of compound **3t** (300 MHz, acetone-d$_6$, 25ºC)

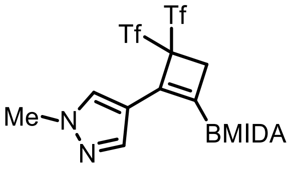


**3t**

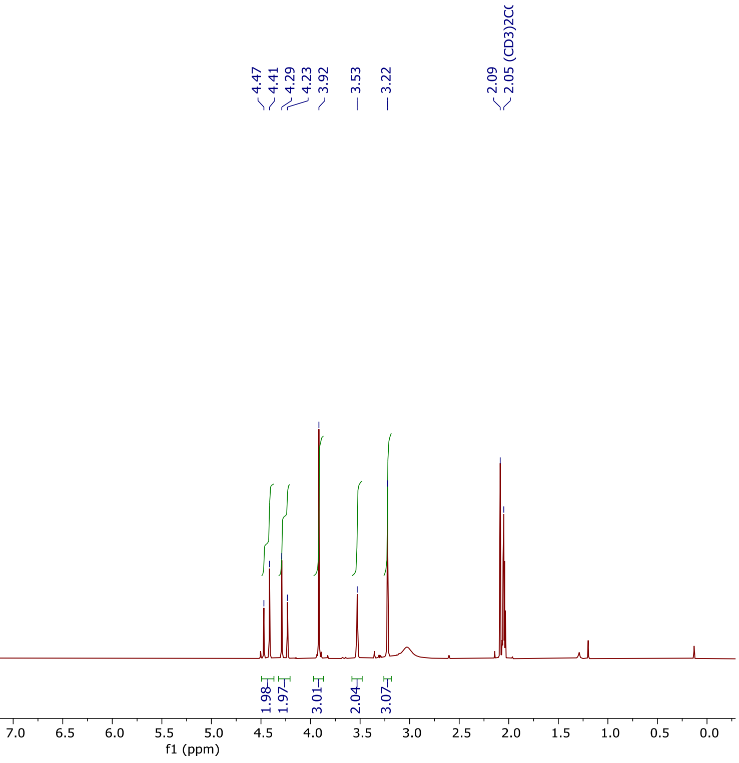


$^{13}$C NMR of compound **3t** (75 MHz, acetone-d$_6$, 25ºC)

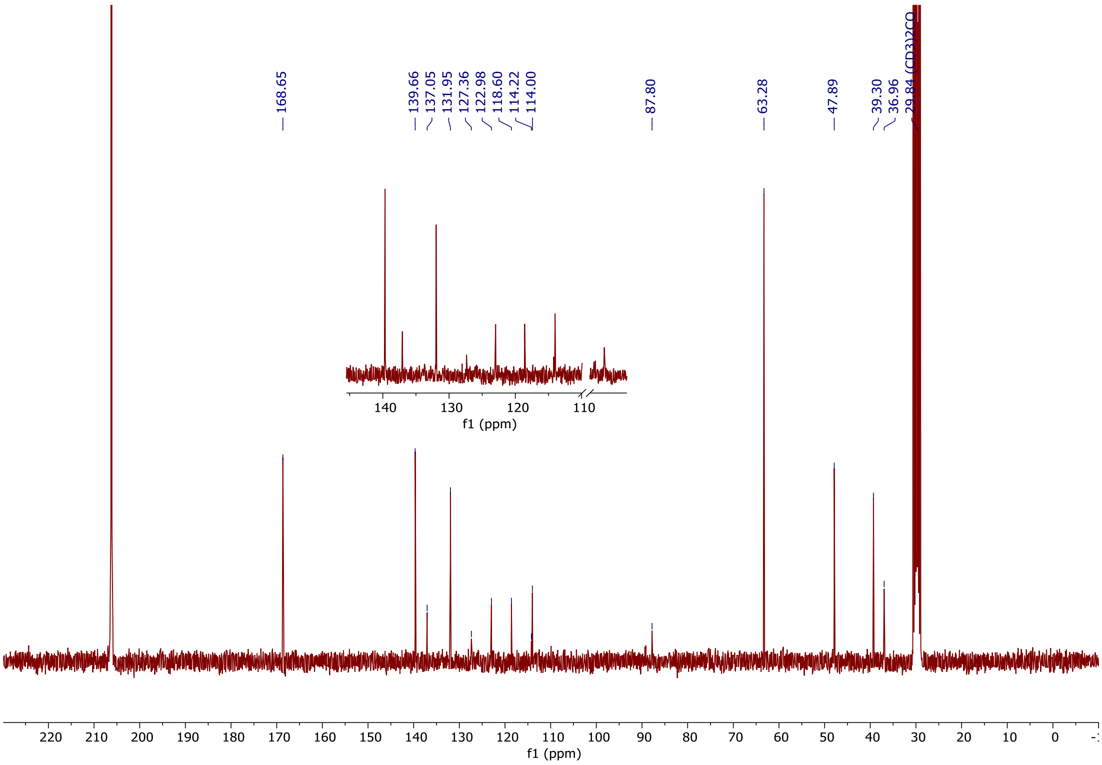

$^{19}F$ NMR of compound **3t** (282 MHz, acetone-$d_6$, 25ºC)

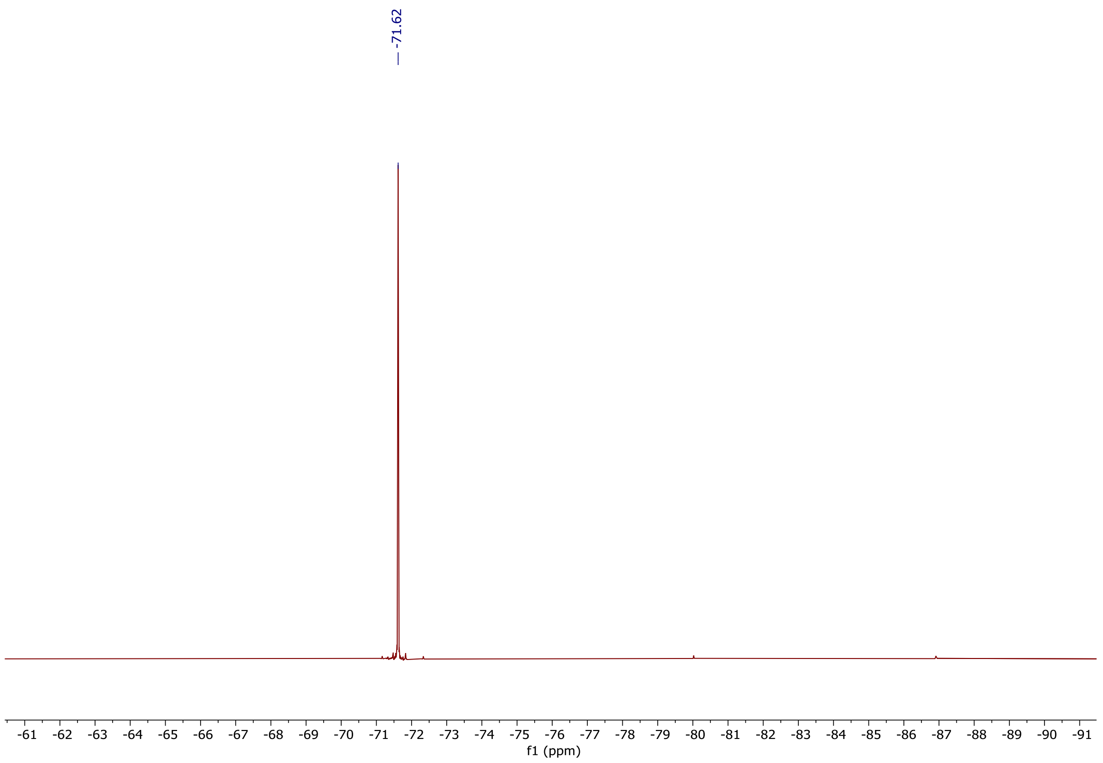

$^{1}$H NMR of compound **3u** (300 MHz, acetone-$d_6$, 25ºC)

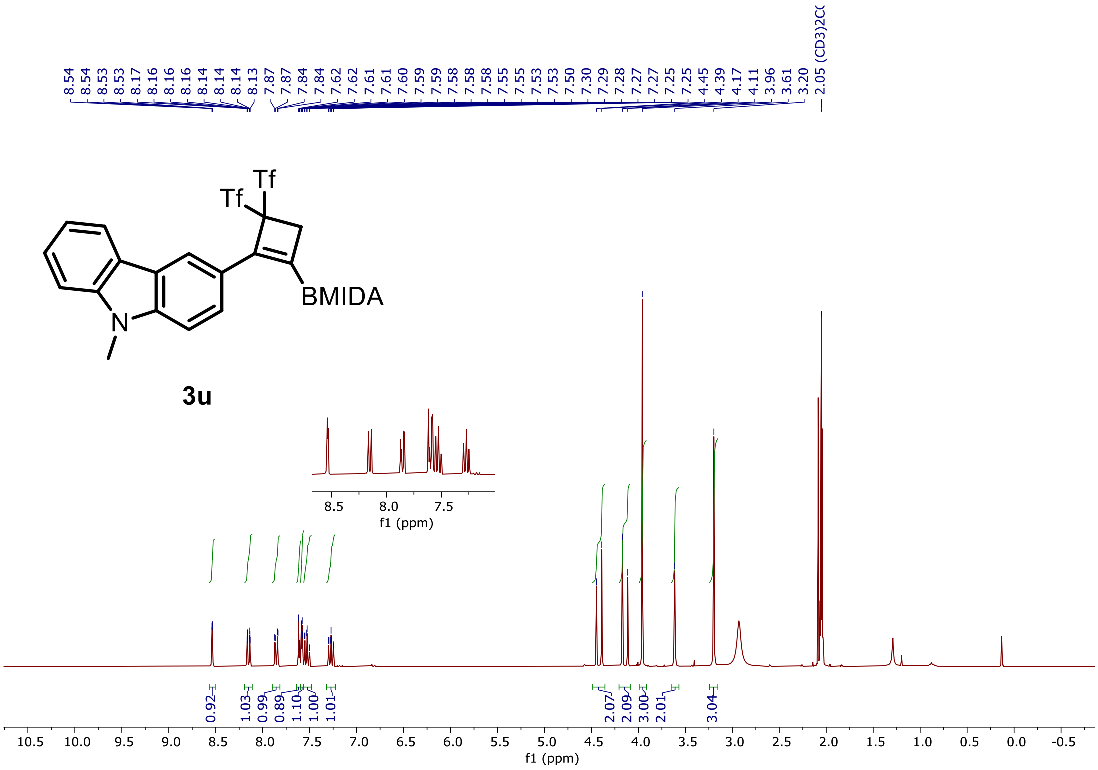


$^{13}$C NMR of compound **3u** (75 MHz, acetone-$d_6$, 25ºC)

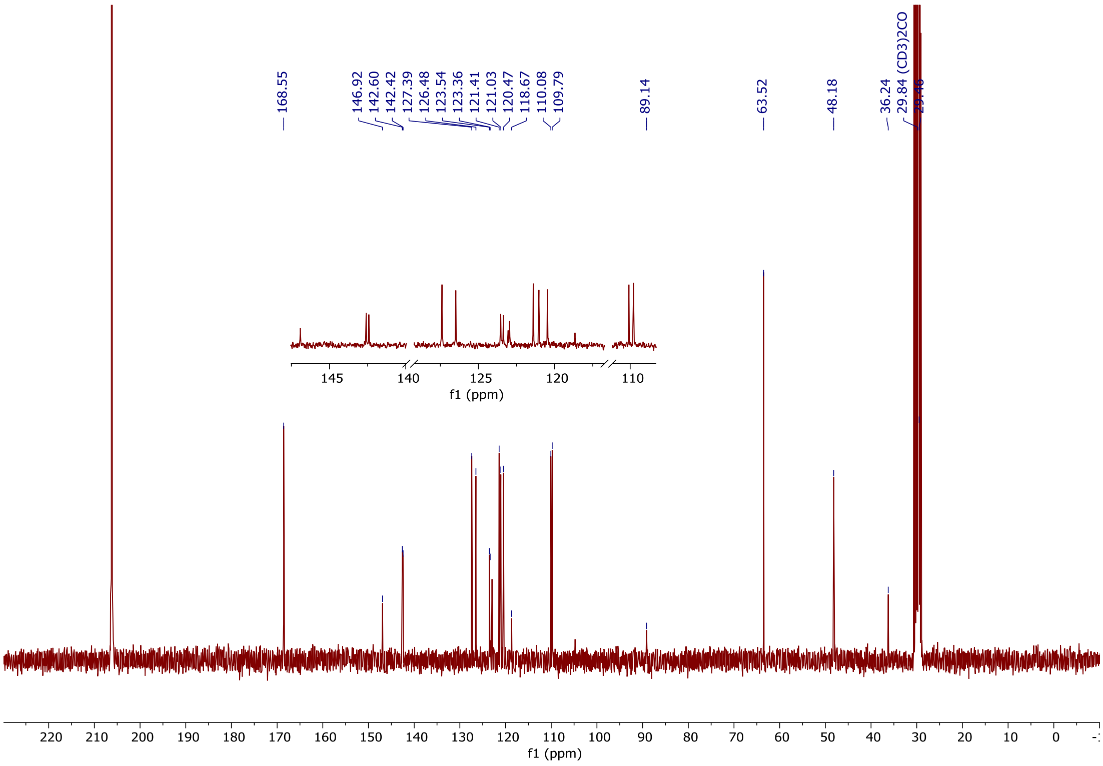

$^{19}F$ NMR of compound **3u** (282 MHz, acetone-$d_6$, 25ºC)

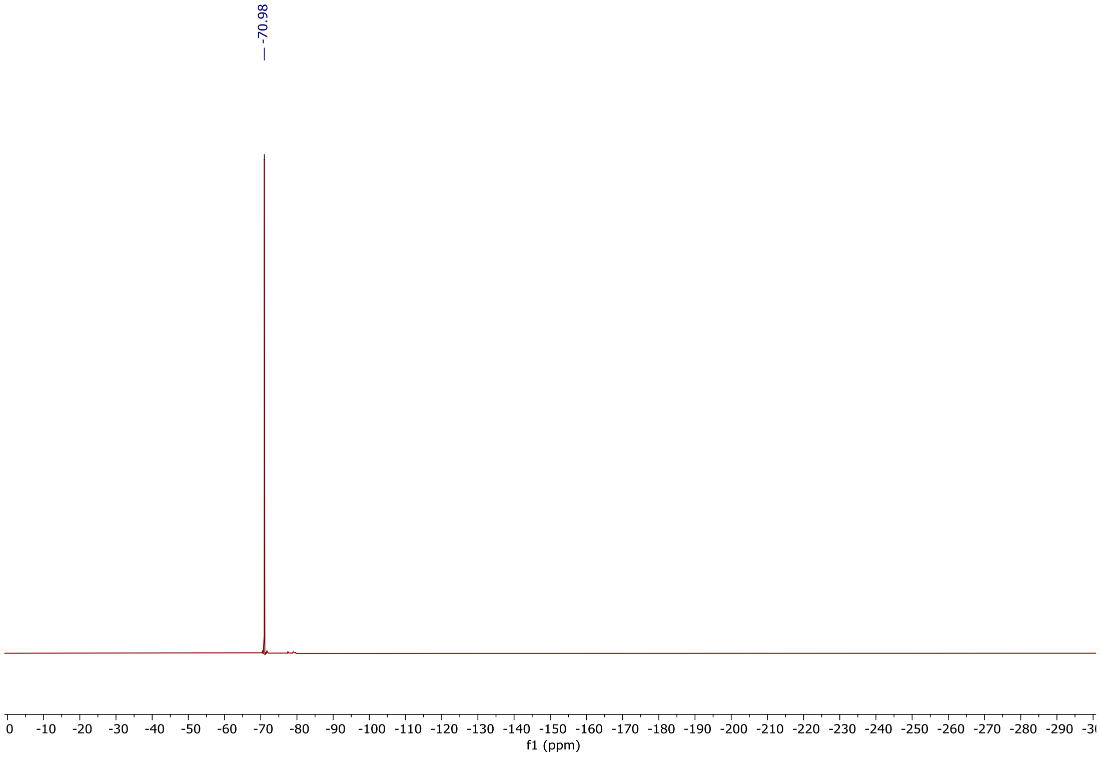

$^{1}$H NMR of compound **3v** (300 MHz, acetone-$d_6$, 25ºC)

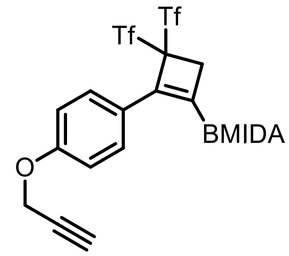


**3v**

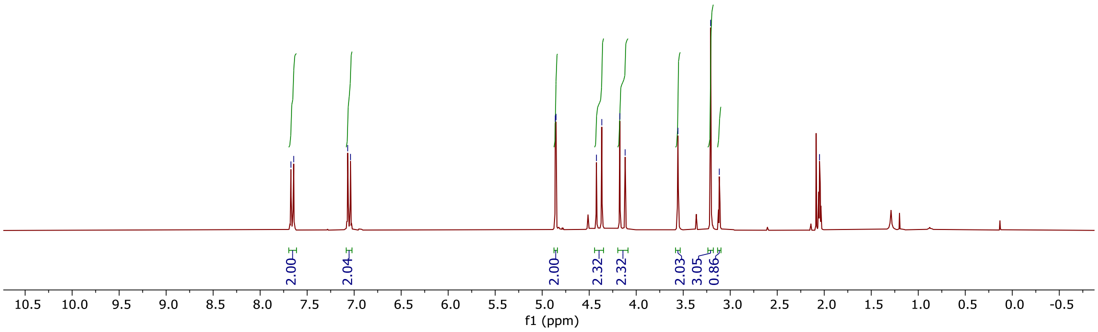


$^{13}$C NMR of compound **3v** (75 MHz, acetone-$d_6$, 25ºC)

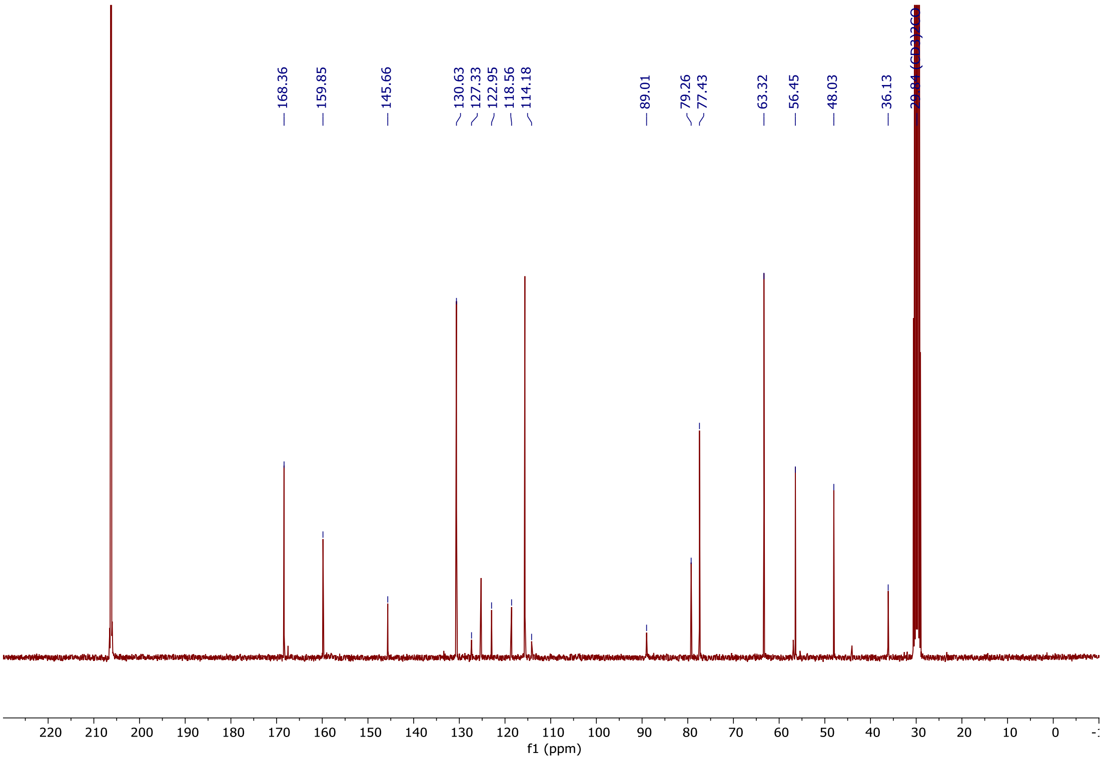

$^{19}$F NMR of compound **3v** (282 MHz, acetone-d$_6$, 25ºC)

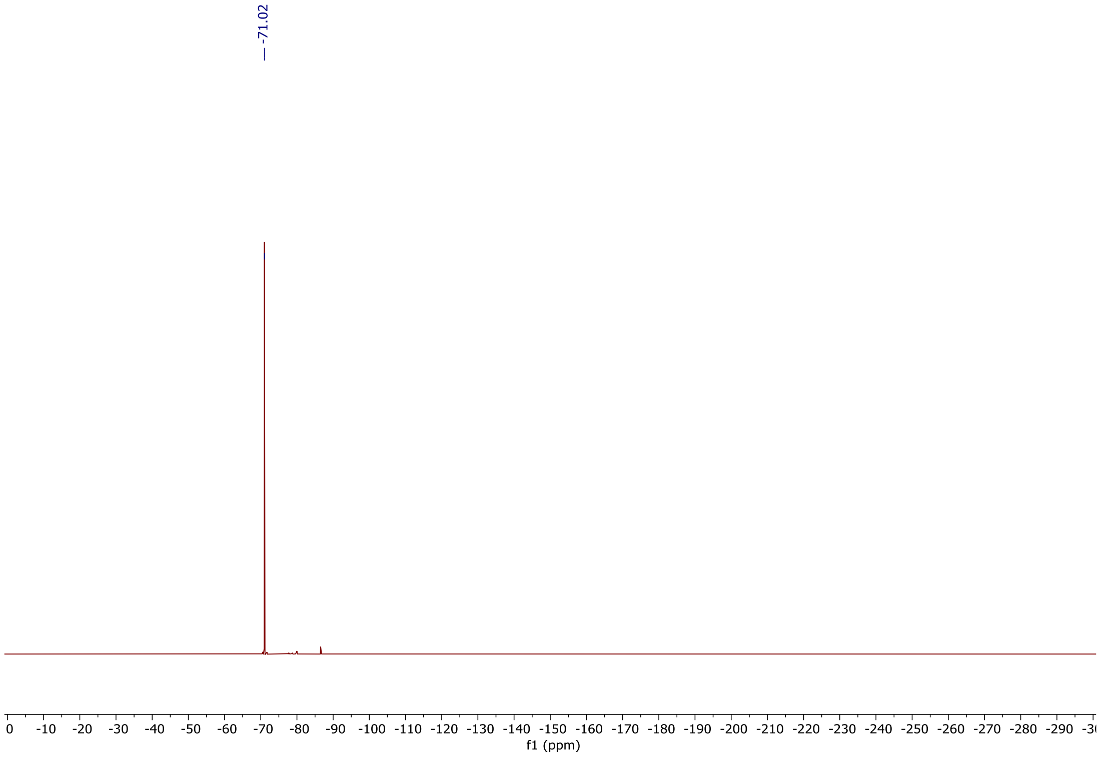

$^{1}$H NMR of compound **3a**-*d*$_2$ (300 MHz, acetone-d$_6$, 25ºC)

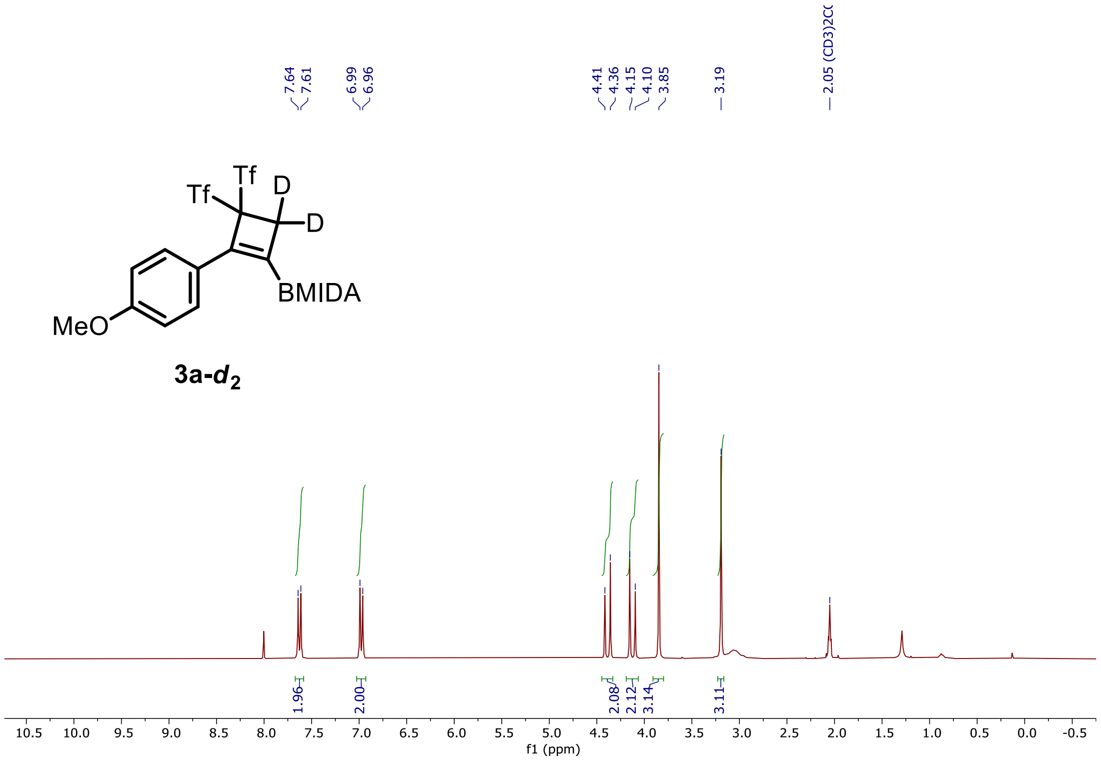


$^{13}$C NMR of compound **3a**-*d*$_2$ (75 MHz, acetone-d$_6$, 25ºC)

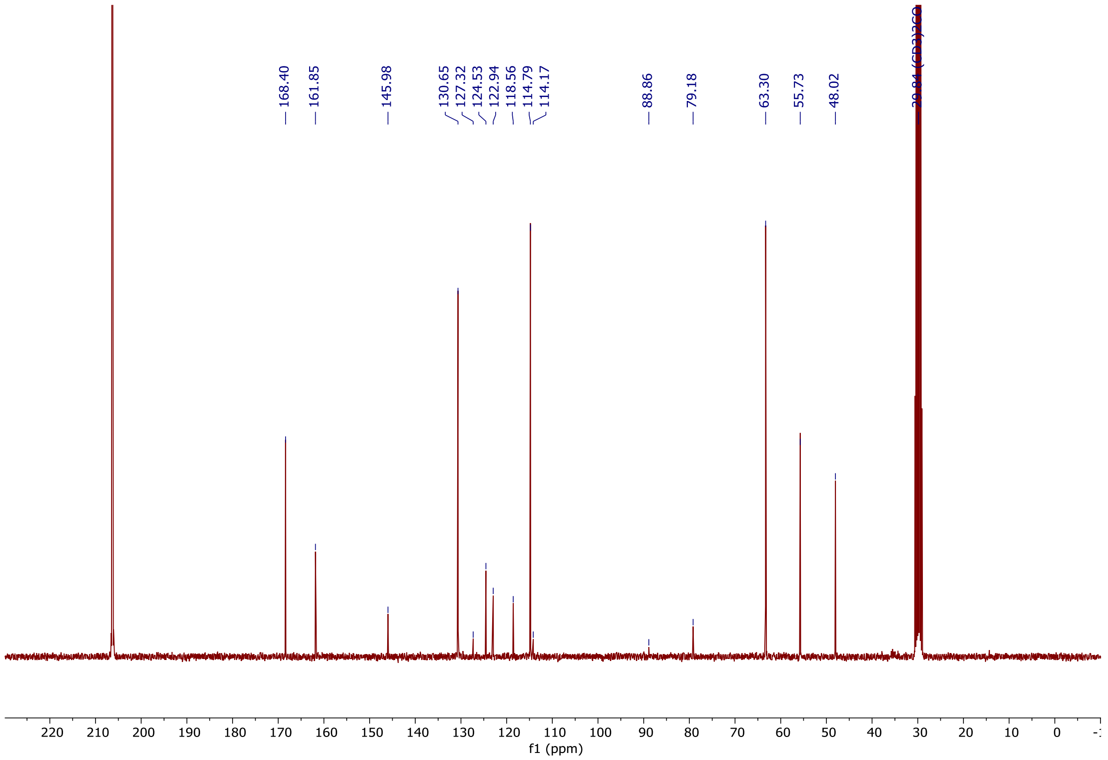

$^{19}$F NMR of compound **3a**-$d_2$ (282 MHz, acetone-d$_6$, 25ºC)

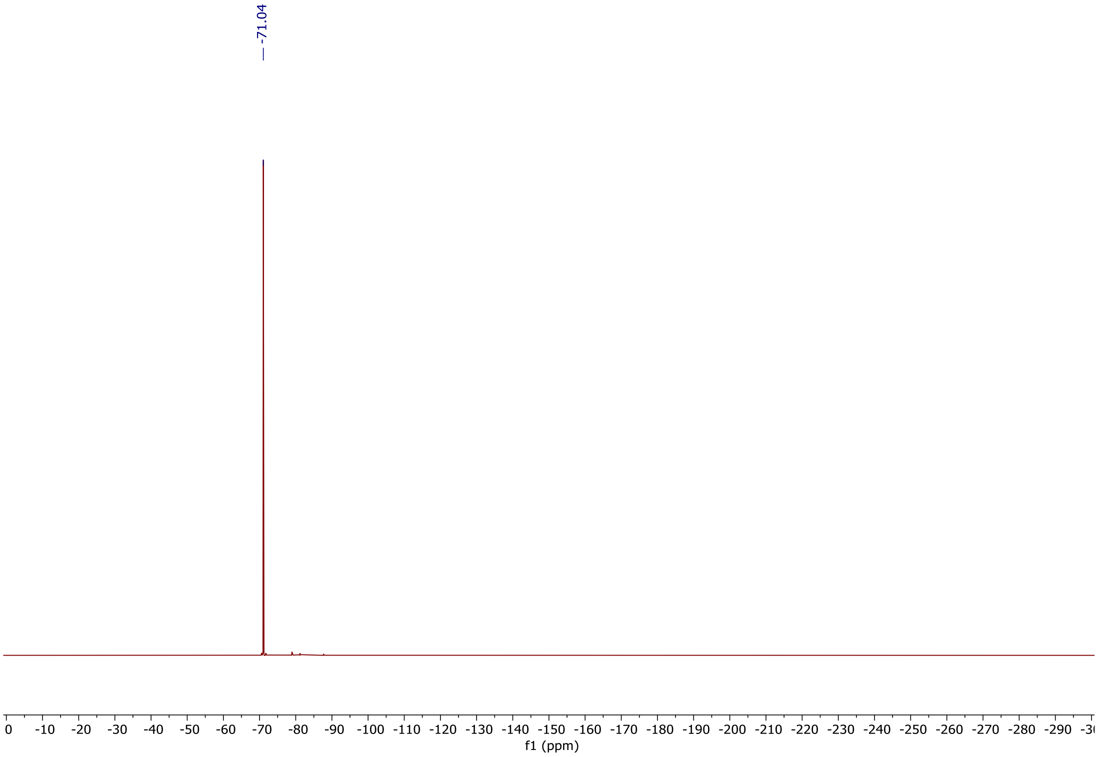

$^{1}$H NMR of compound **3n**-$d_2$ (300 MHz, acetone-d$_6$, 25ºC)

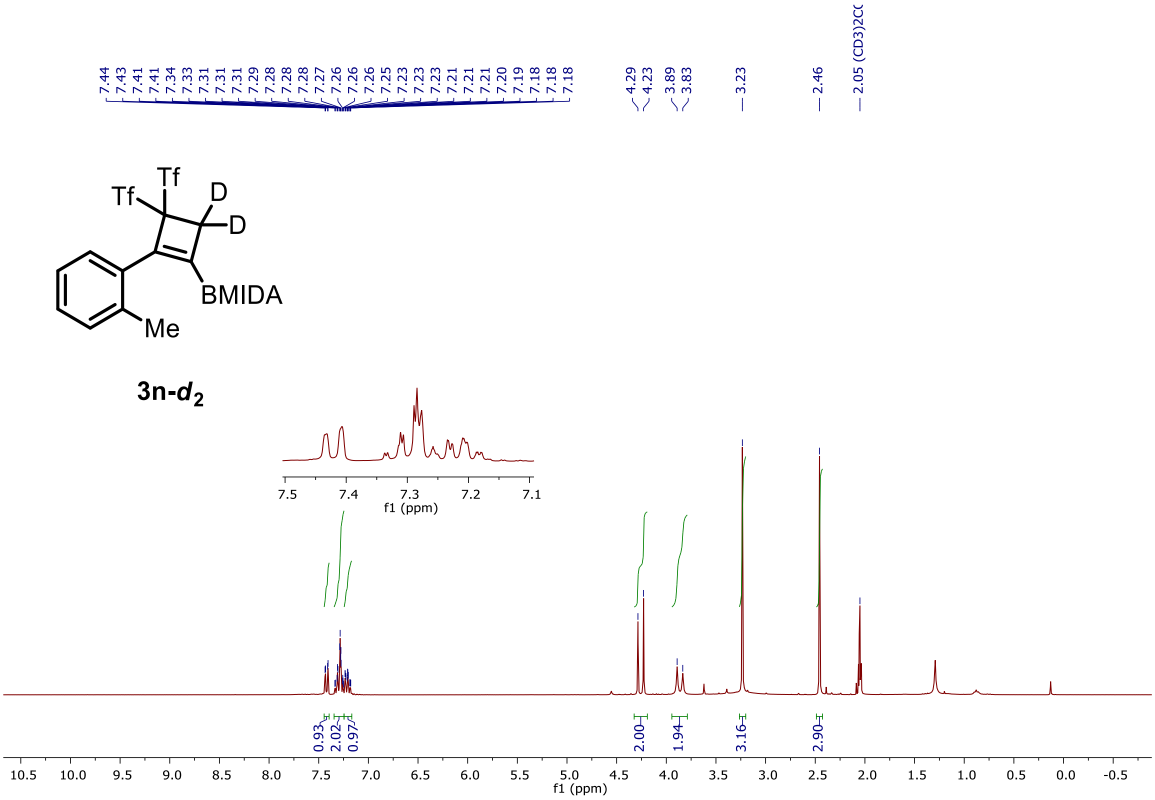


$^{13}$C NMR of compound **3n**-$d_2$ (75 MHz, acetone-d$_6$, 25ºC)

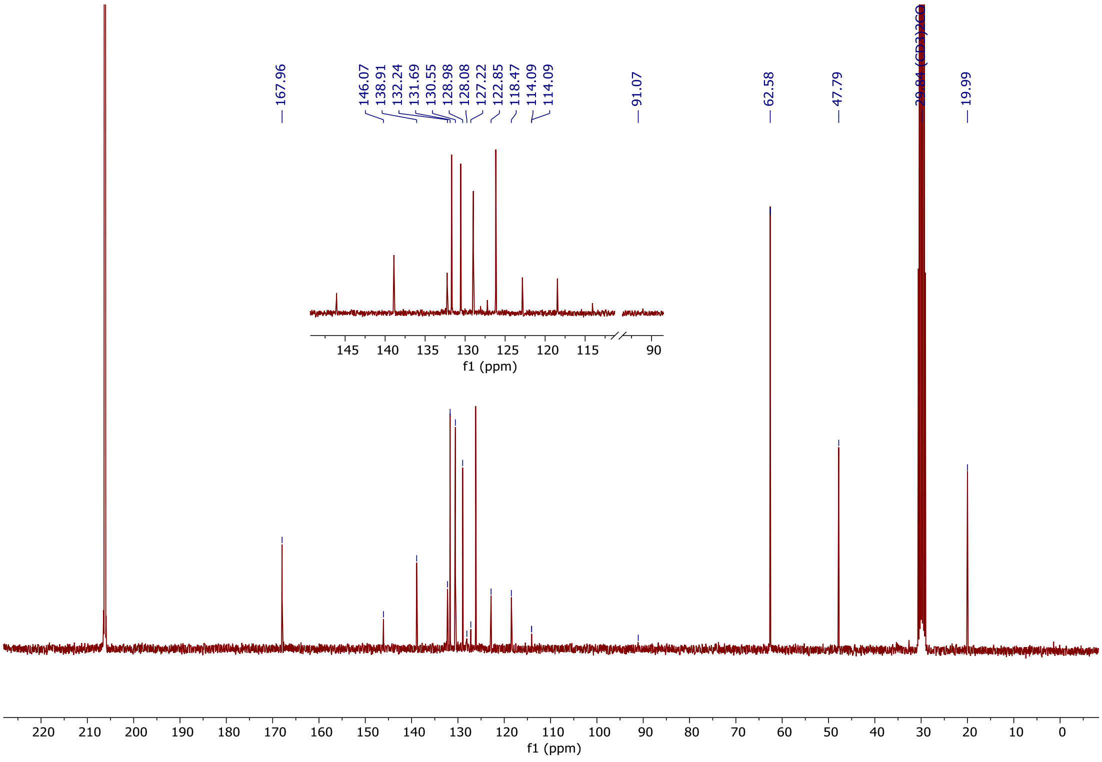

$^{19}F$ NMR of compound **3n**-$d_2$ (282 MHz, acetone-$d_6$, 25ºC)

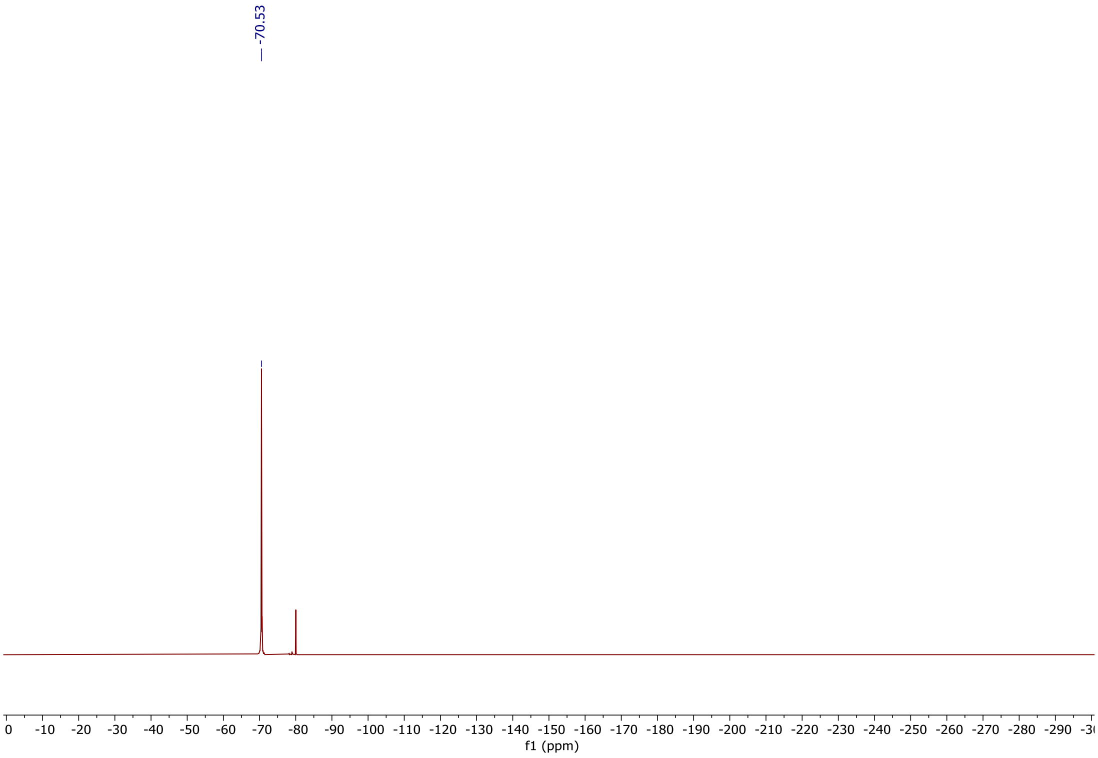

$^{1}H$ NMR of compound **3p**-$d_2$ (300 MHz, acetone-$d_6$, 25ºC)

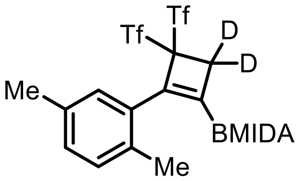


**3p-$d_2$**

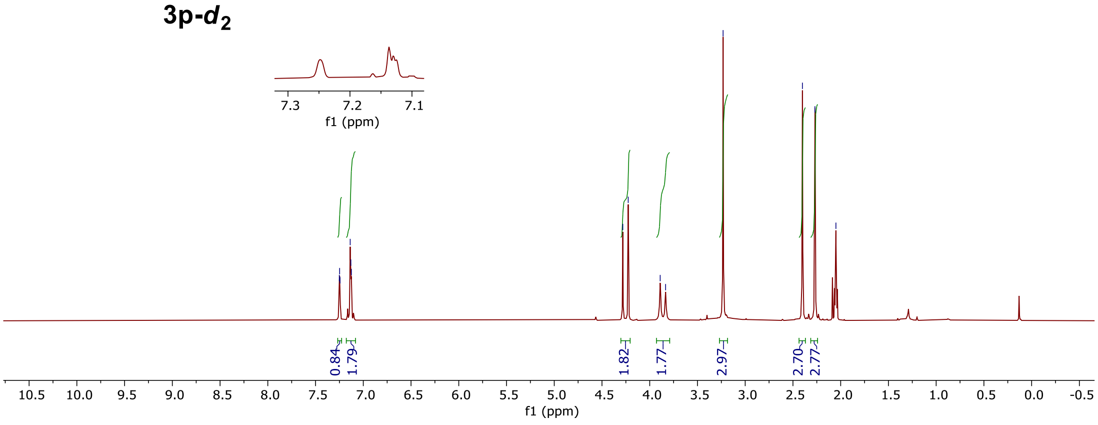


$^{13}C$ NMR of compound **3p**-$d_2$ (75 MHz, acetone-$d_6$, 25ºC)

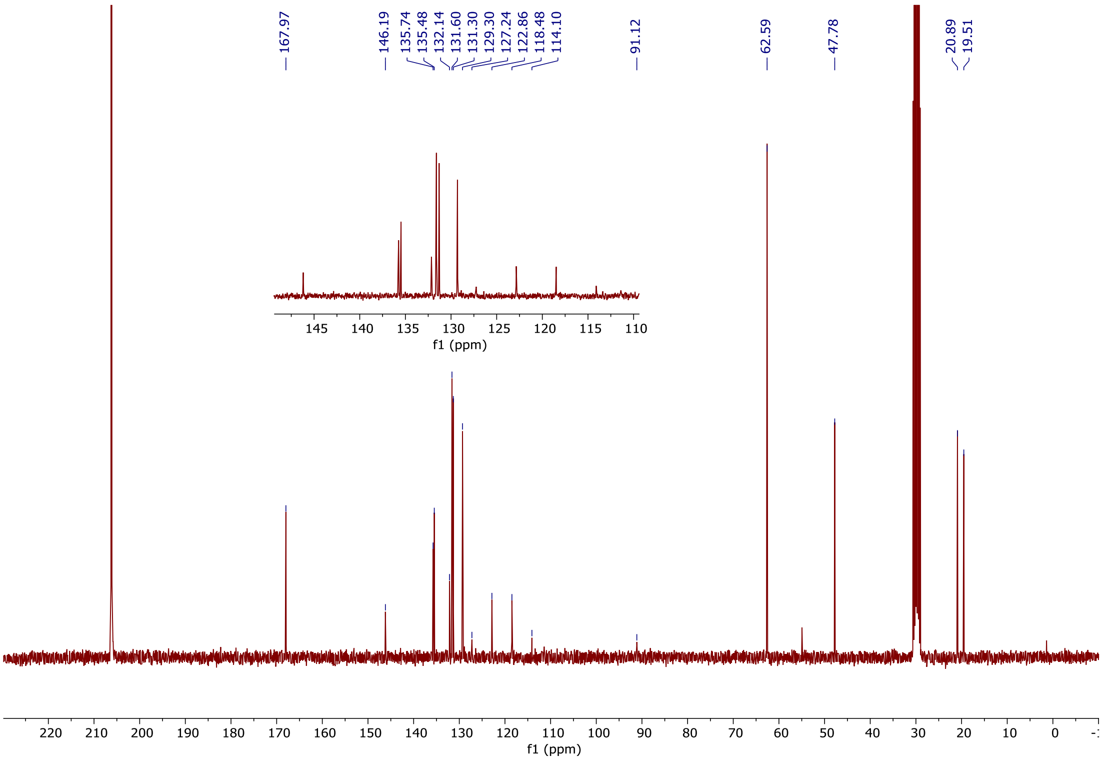

$^{19}$F NMR of compound **3p**-$d_2$ (282 MHz, acetone-d$_6$, 25ºC)

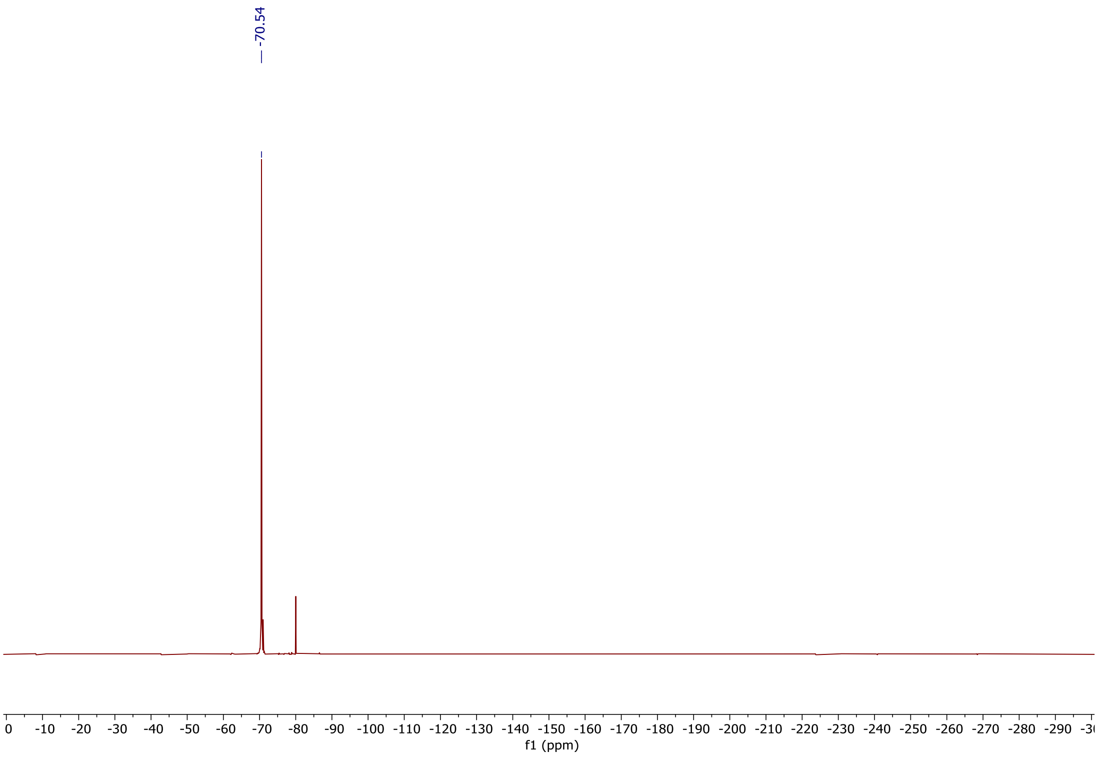

$^{1}H$ NMR of compound **3q**-$d_2$ (300 MHz, acetone-$d_6$, 25ºC)

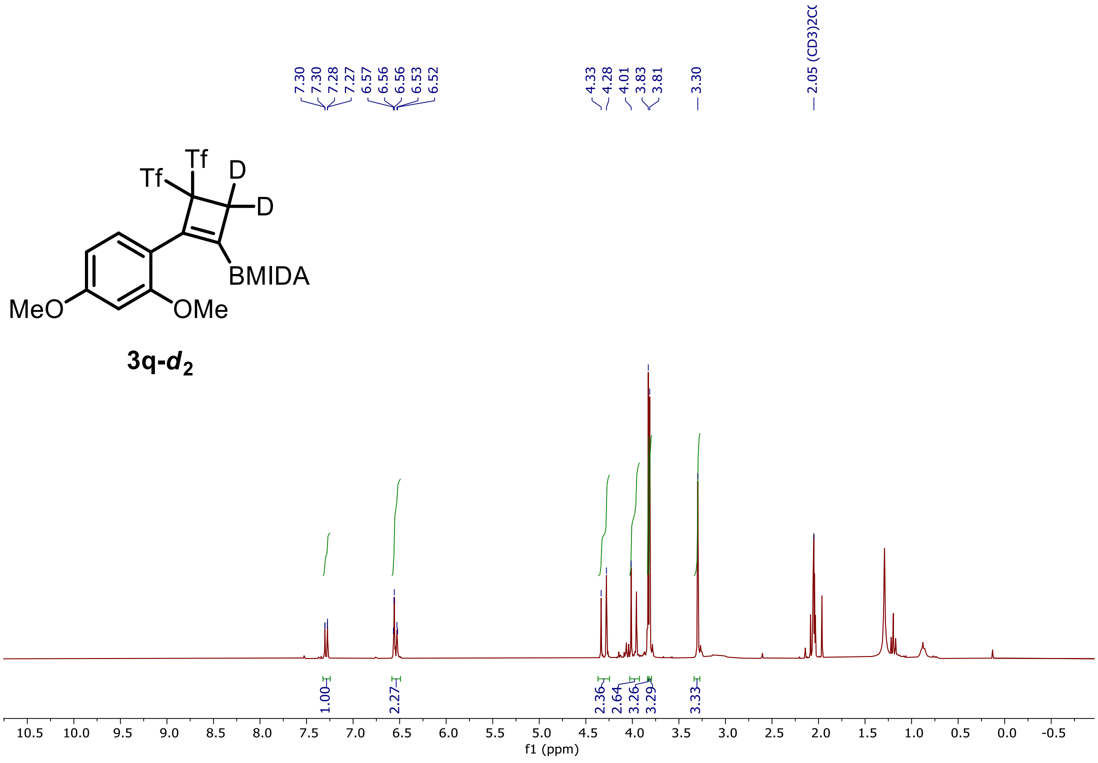


$^{13}C$ NMR of compound **3q**-$d_2$ (75 MHz, acetone-$d_6$, 25ºC)

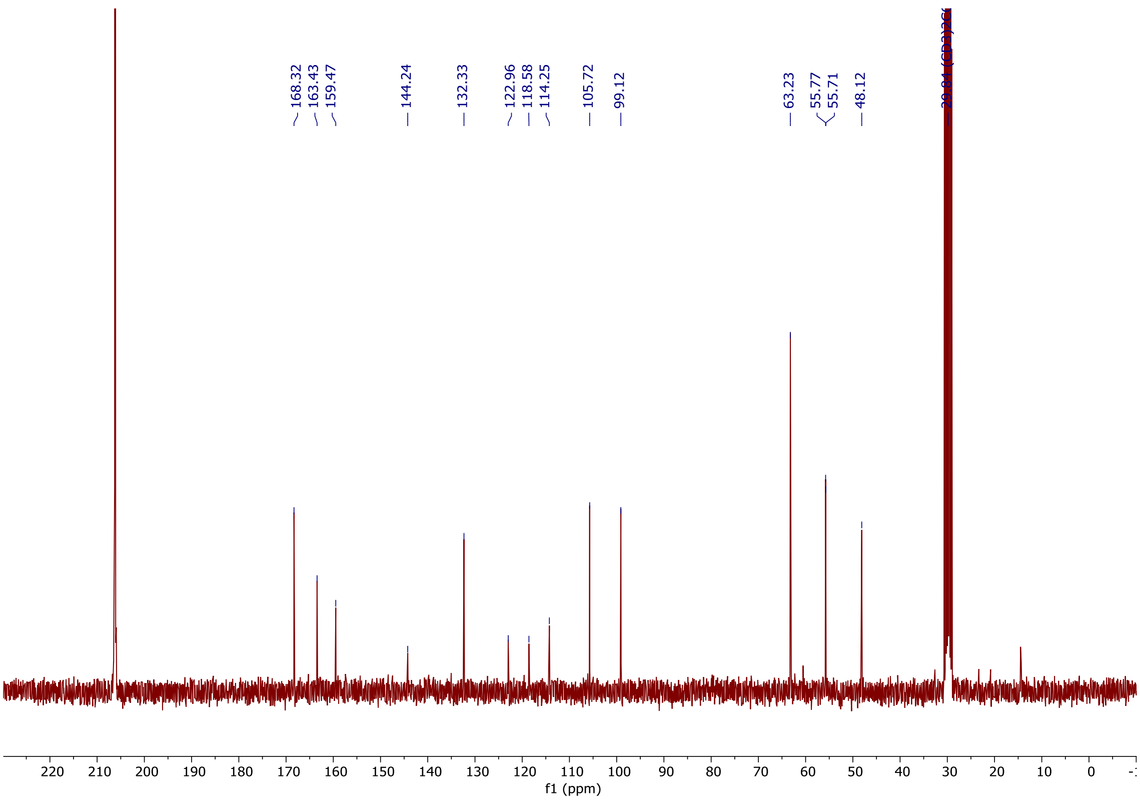

$^{19}$F NMR of compound **3q**-$d_2$ (282 MHz, acetone-$d_6$, 25ºC)

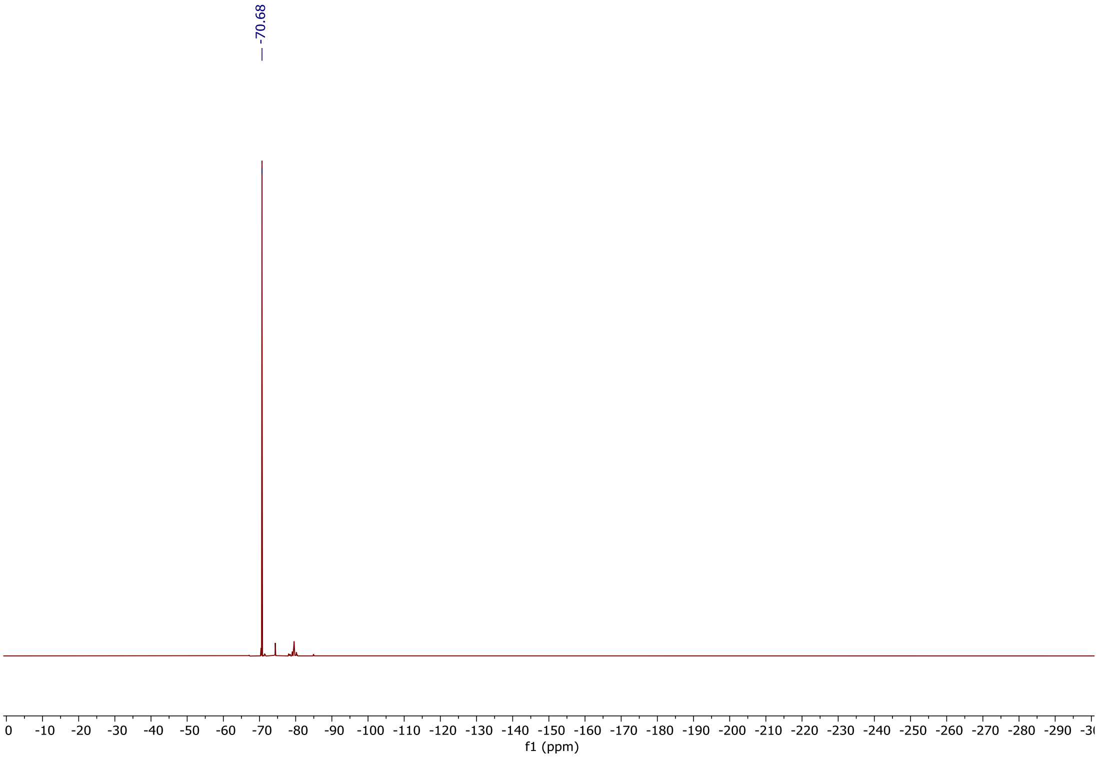

$^{1}H$ NMR of compound **4a** (300 MHz, acetone-$d_6$, 25ºC)

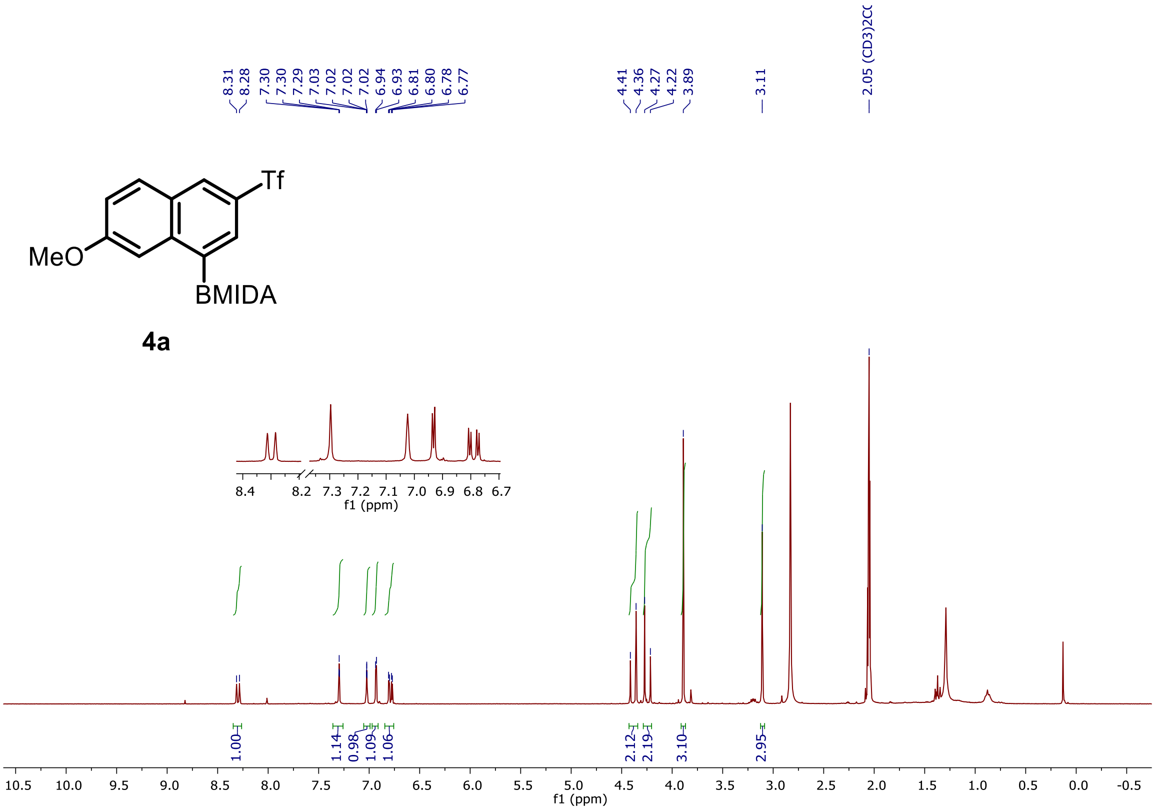


$^{1}H$-$^{1}H$ COSY NMR of compound **4a** (700 MHz, acetone-$d_6$, 25ºC)

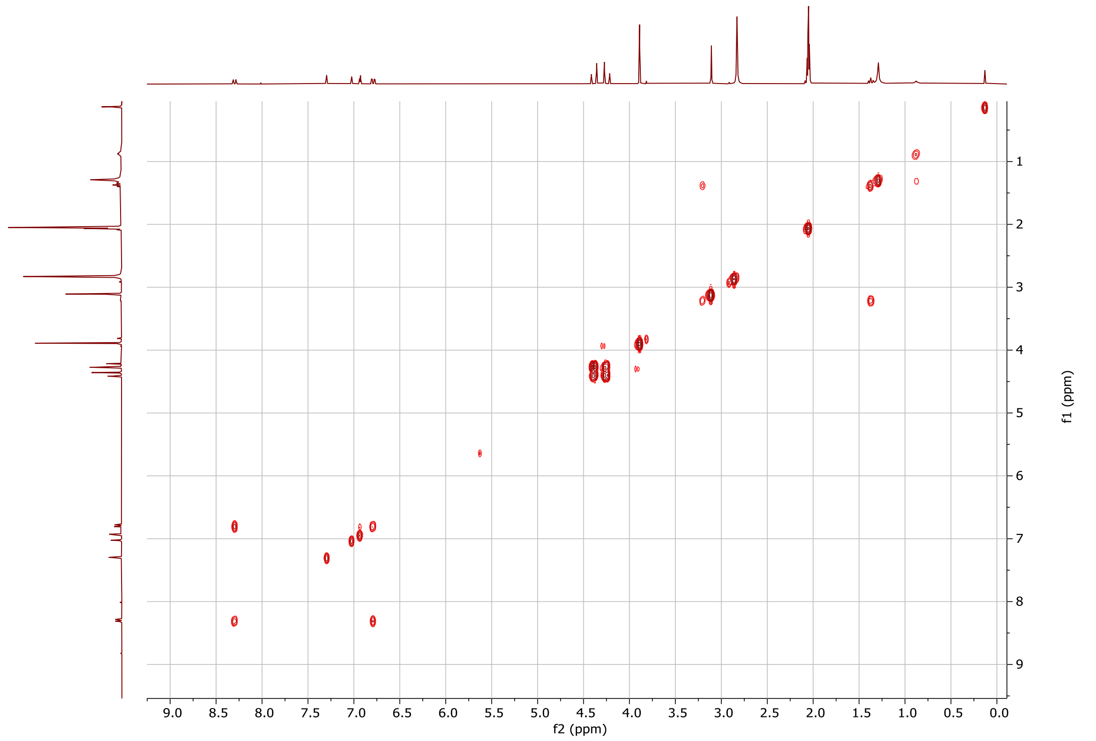

## $^{1}H$-$^{1}H$ TOCSY NMR of compound **4a** (400 MHz, acetone-$d_6$, 25ºC)

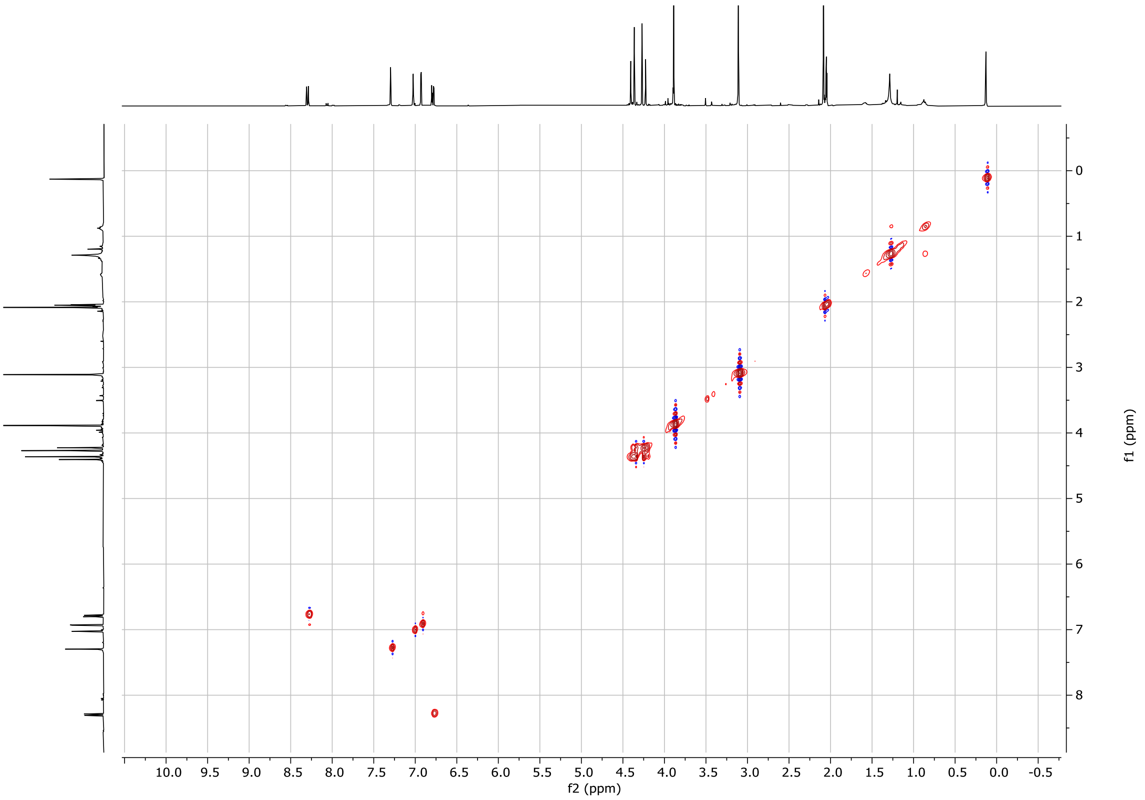


## $^{13}C$ NMR of compound **4a** (176 MHz, acetone-$d_6$, 25ºC)

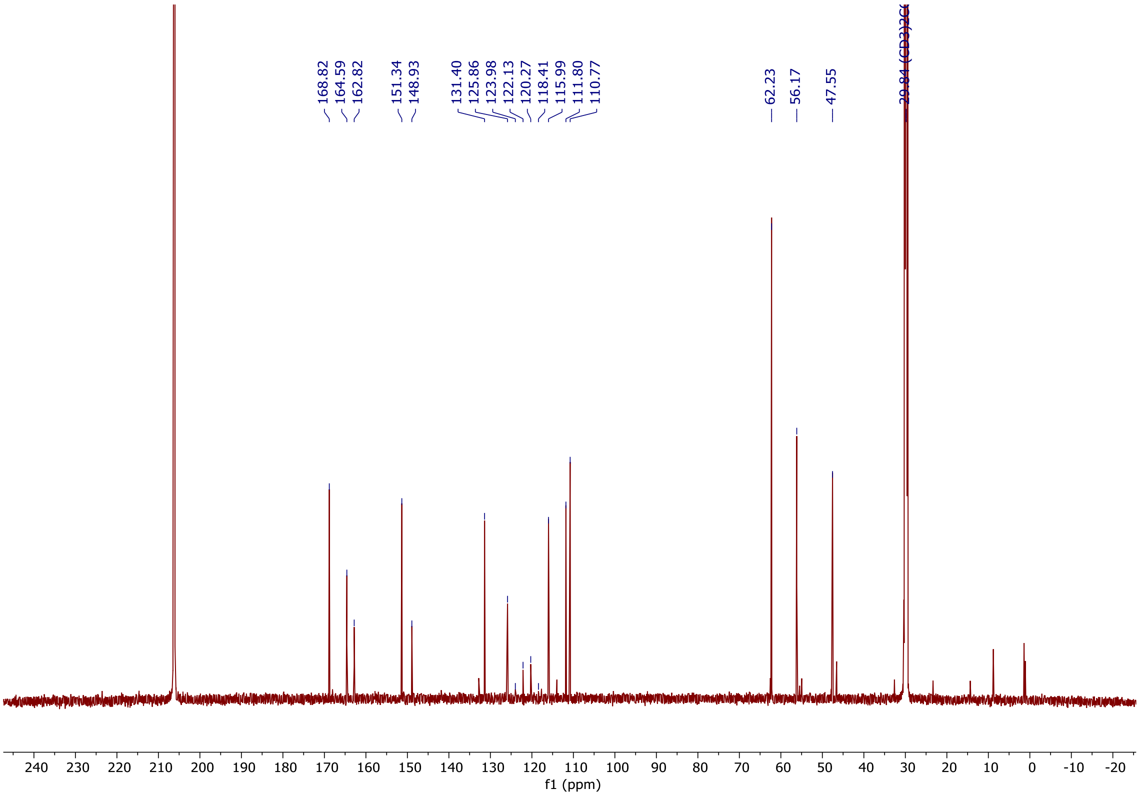

$^{19}$F NMR of compound **4a** (282 MHz, acetone-d$_6$, 25ºC)

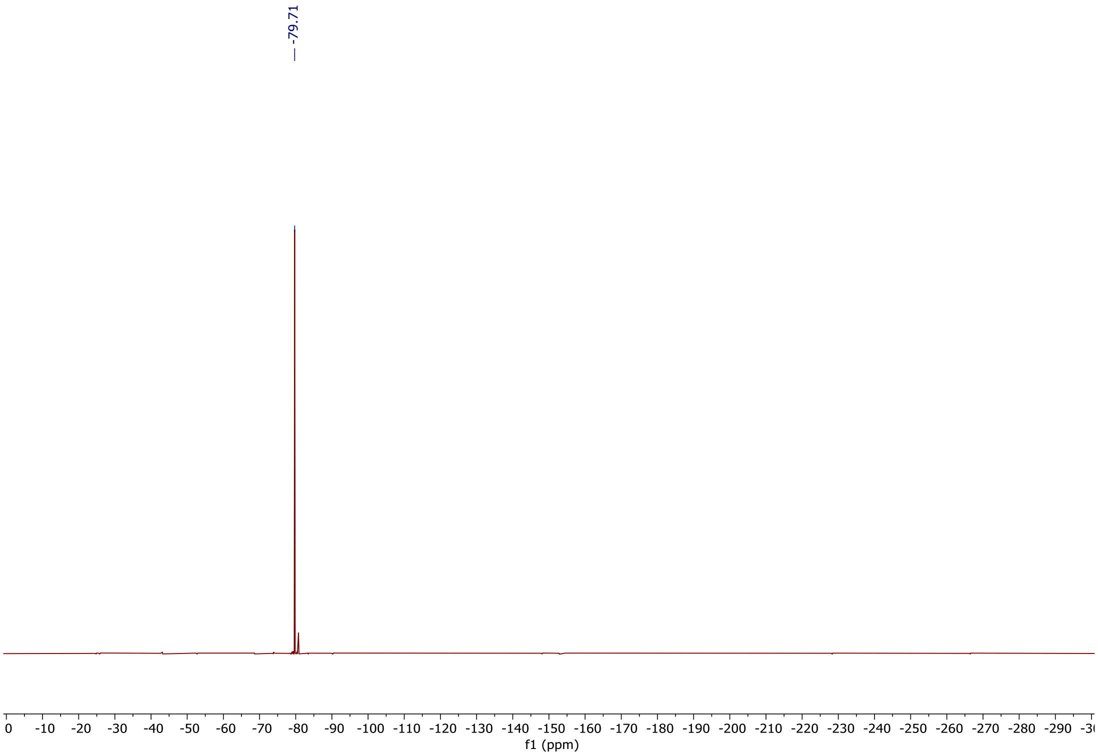


$^1$H-$^{13}$C HMBC NMR of compound **4a** (176 MHz /700 MHz, acetone-d$_6$, 25ºC)

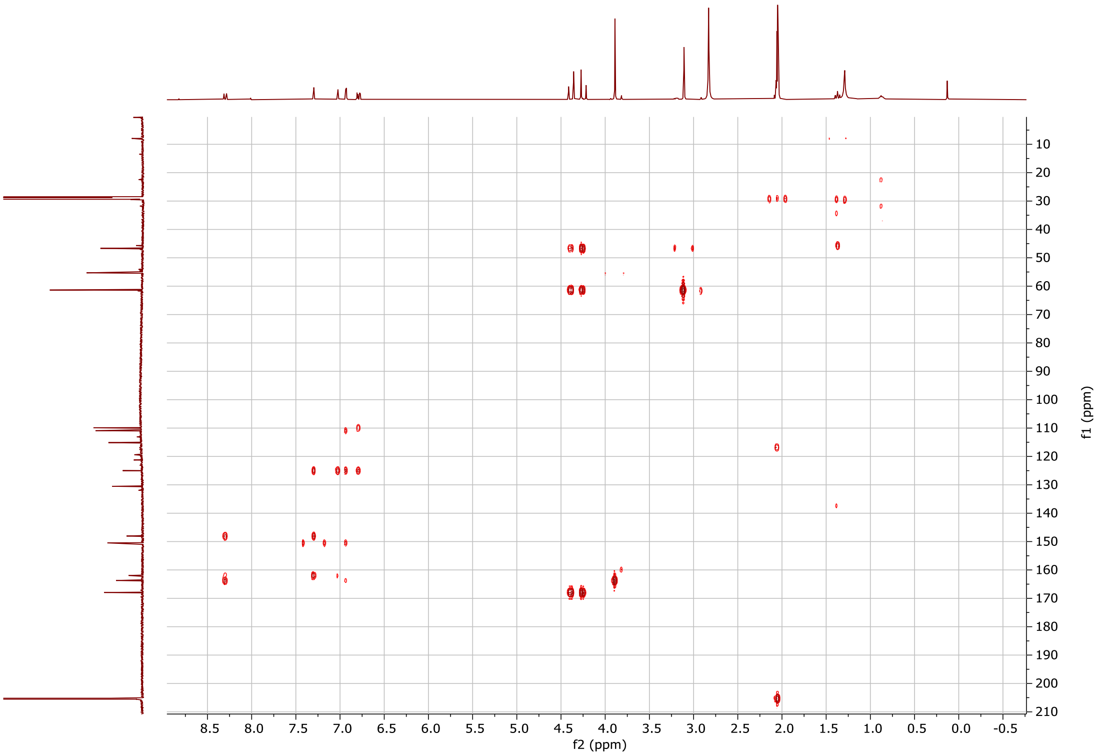

$^{1}H$-$^{13}C$ HSQC NMR of compound **4a** (176 MHz /700 MHz, acetone-$d_6$, 25ºC)

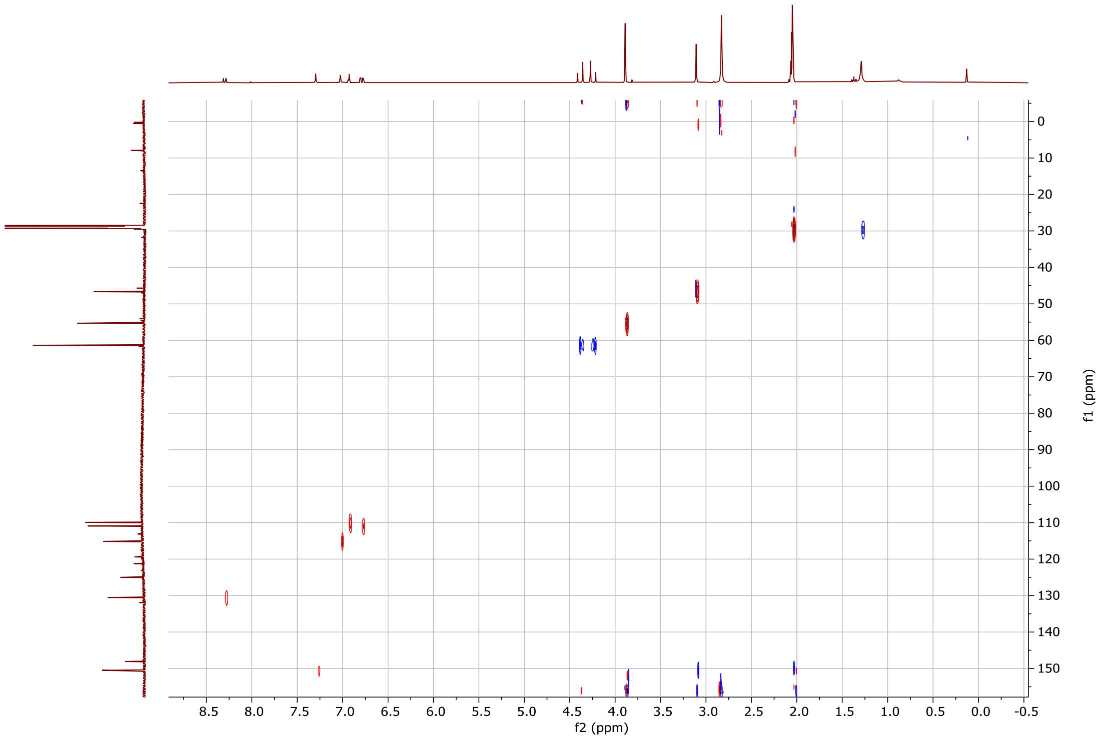

$^{1}H$ NMR of compound **4a**-*d* (700 MHz, acetone-$d_6$, 25ºC)

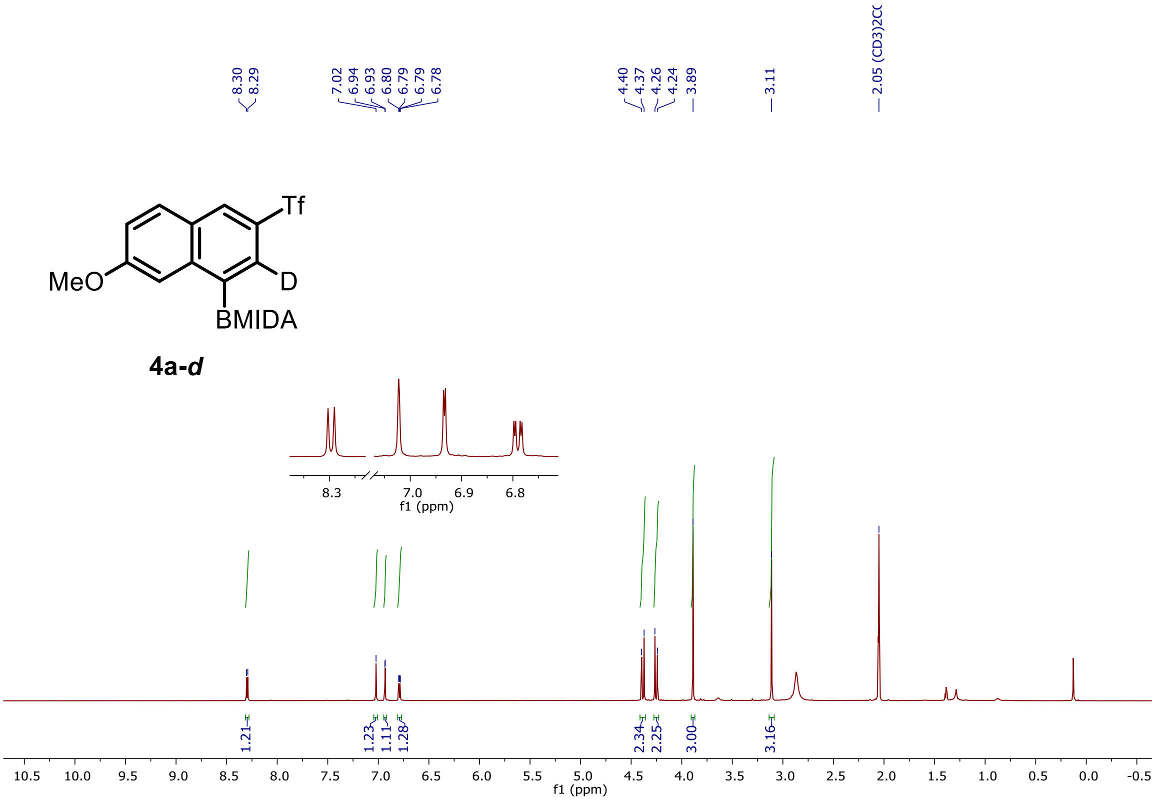


$^{13}C$ NMR of compound **4a**-*d* (176 MHz, acetone-$d_6$, 25ºC)

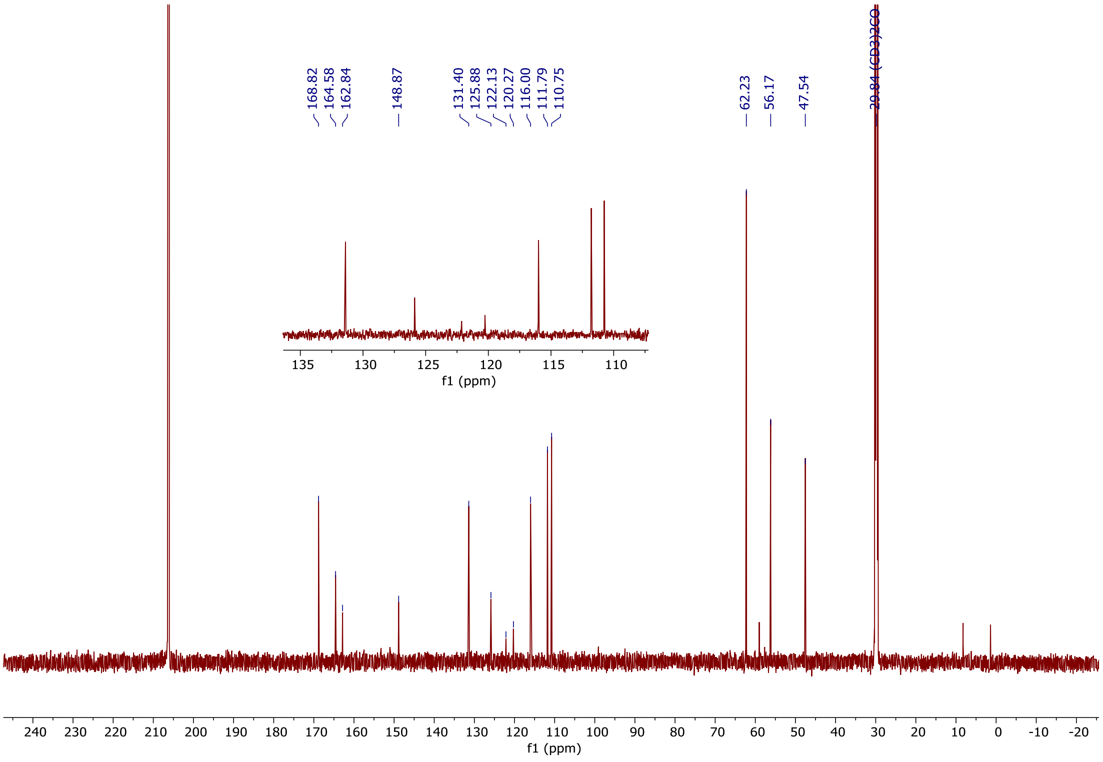

$^{19}$F NMR of compound **4a**-*d* (282 MHz, acetone-d$_6$, 25ºC)

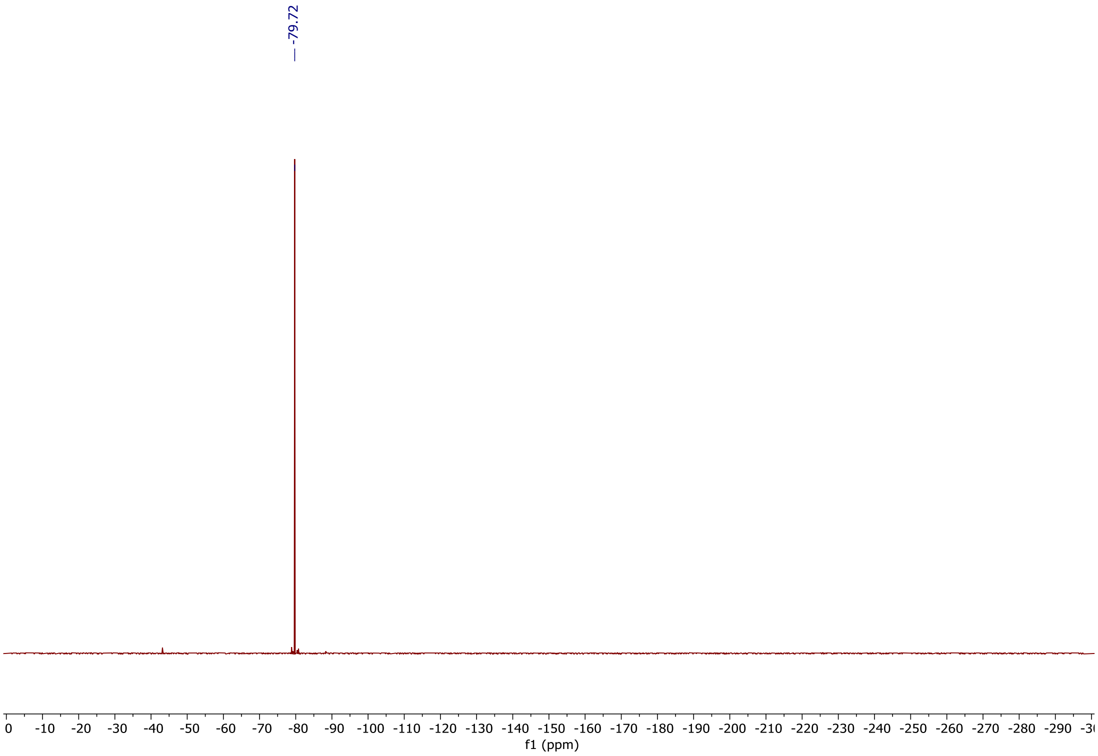


$^1$H-$^{13}$C HMBC NMR of compound **4a**-*d* (176 MHz /700 MHz, acetone-d$_6$, 25ºC)

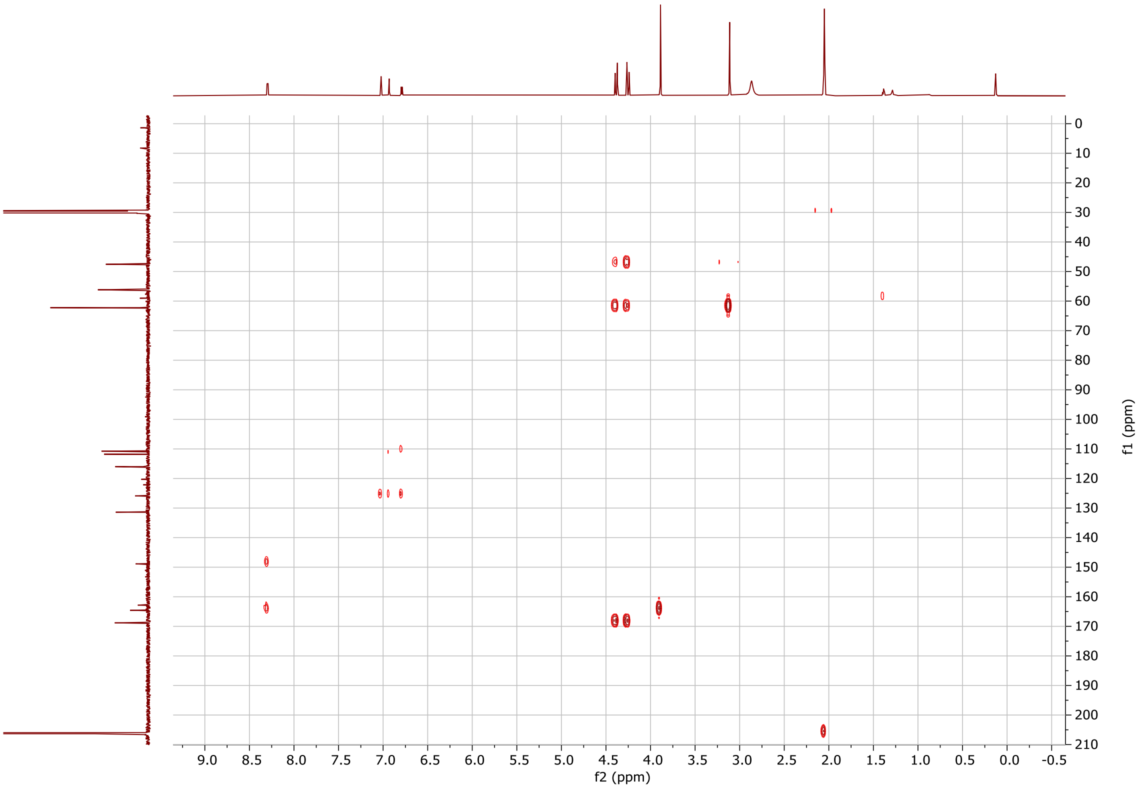

$^{1}H$-$^{13}C$ HSQC NMR of compound **4a**-*d* (176 MHz /700 MHz, acetone-$d_6$, 25ºC)

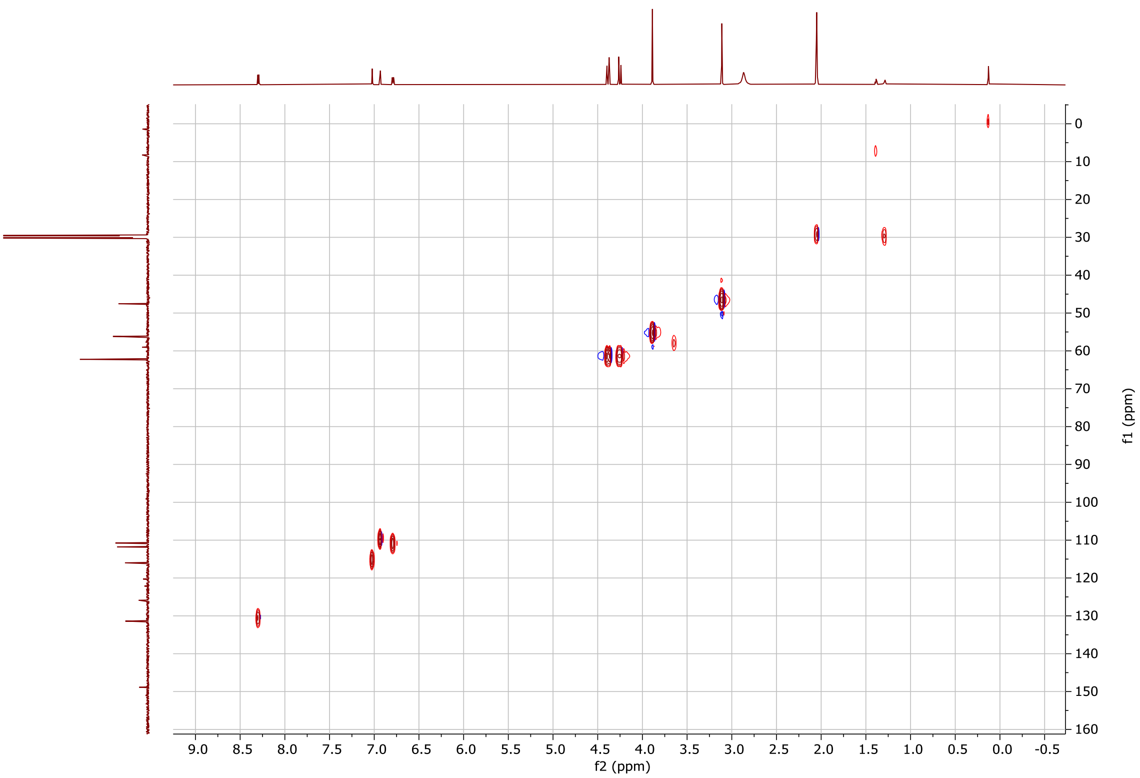

$^{1}$H NMR of compound **4b** (700 MHz, acetone-$d_6$, 25ºC)

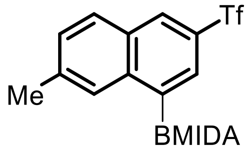


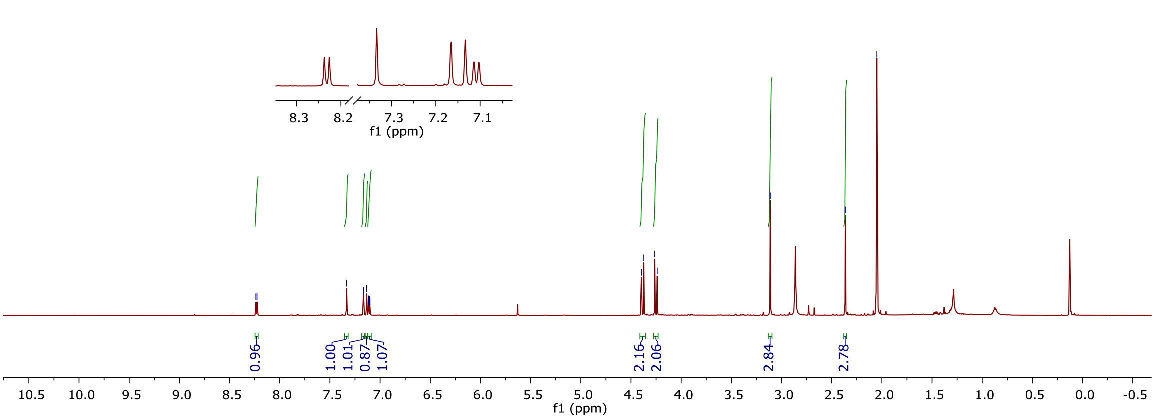


$^{13}$C NMR of compound **4b** (176 MHz, acetone-$d_6$, 25ºC)

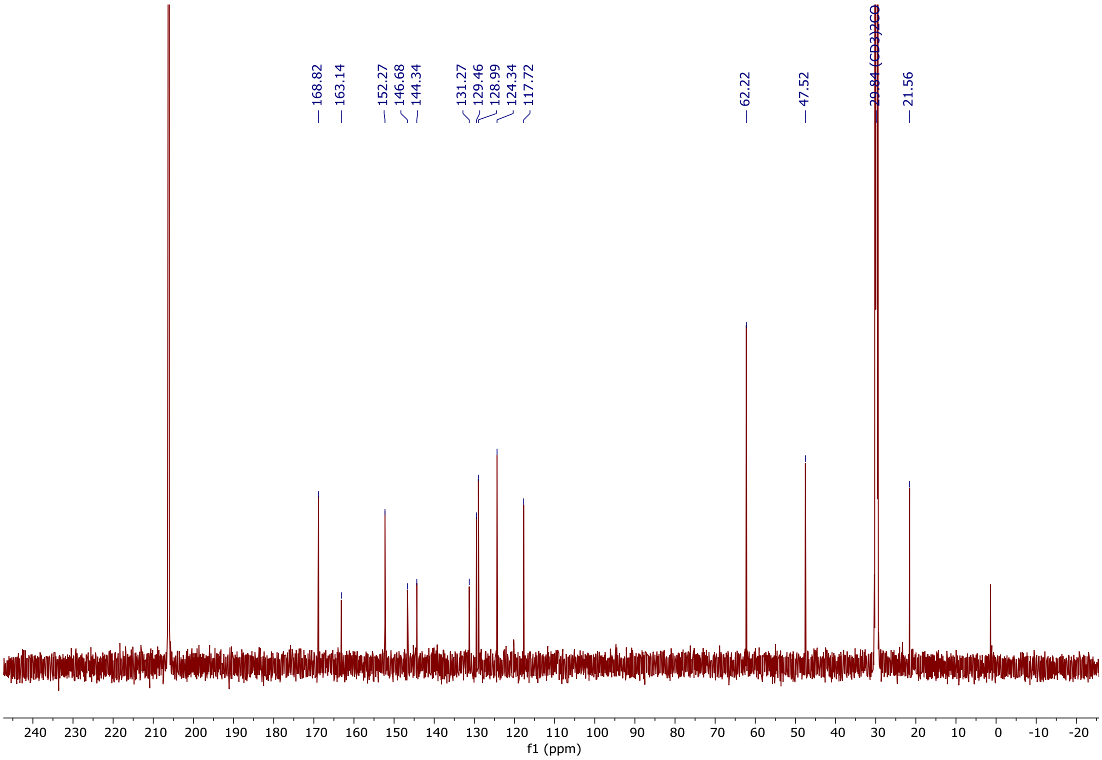

$^{19}$F NMR of compound **4b** (282 MHz, acetone-$d_6$, 25ºC)

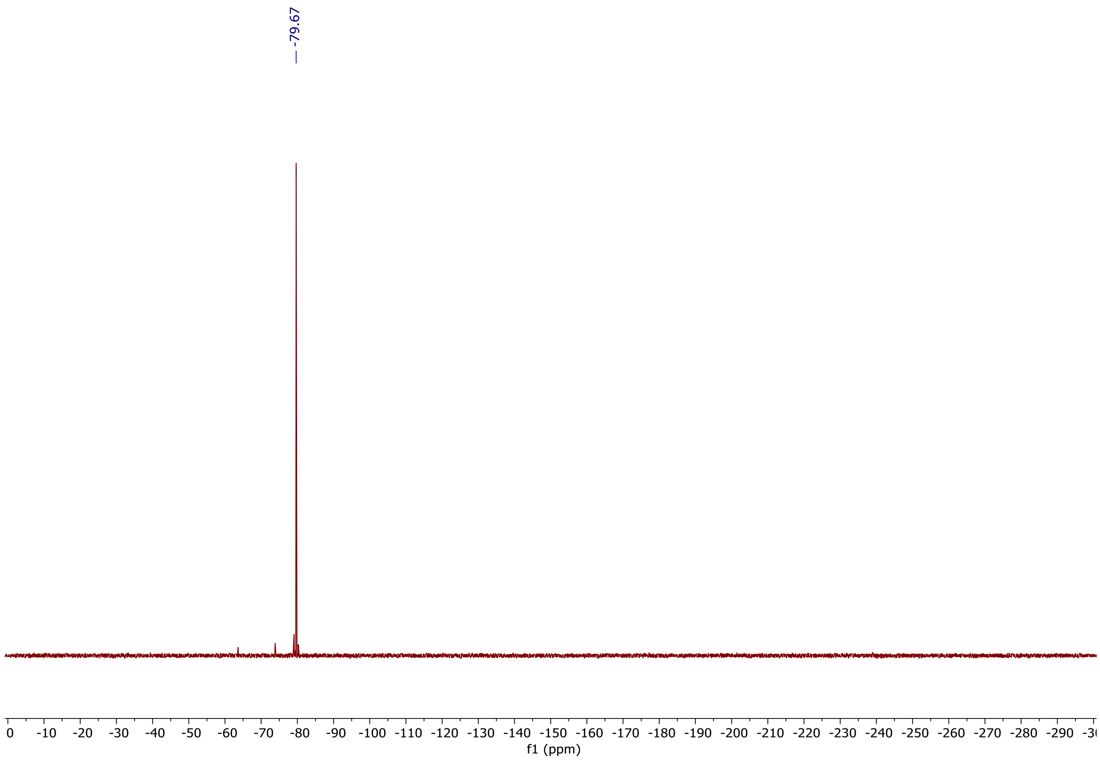

$^{1}$H NMR of compound **4e** (700 MHz, acetone-d$_6$, 25ºC)

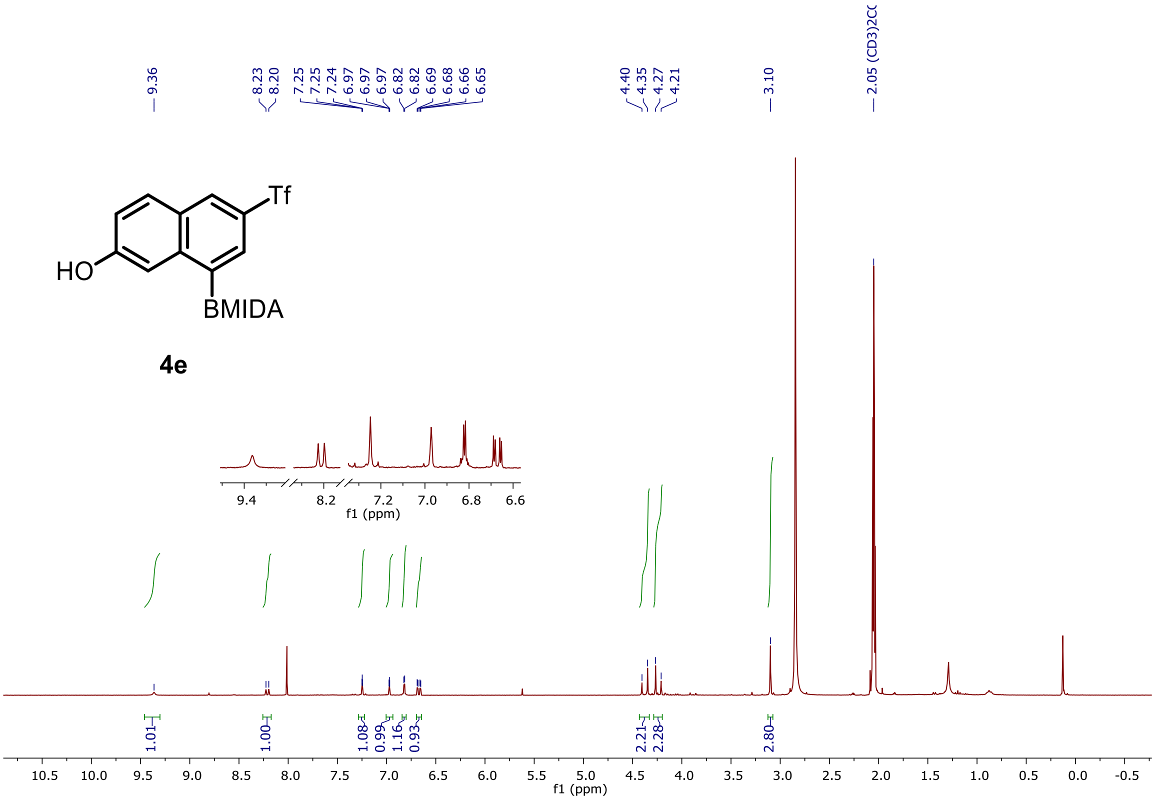


$^{1}$H-$^{1}$H COSY NMR of compound **4e** (700 MHz, acetone-d$_6$, 25ºC)

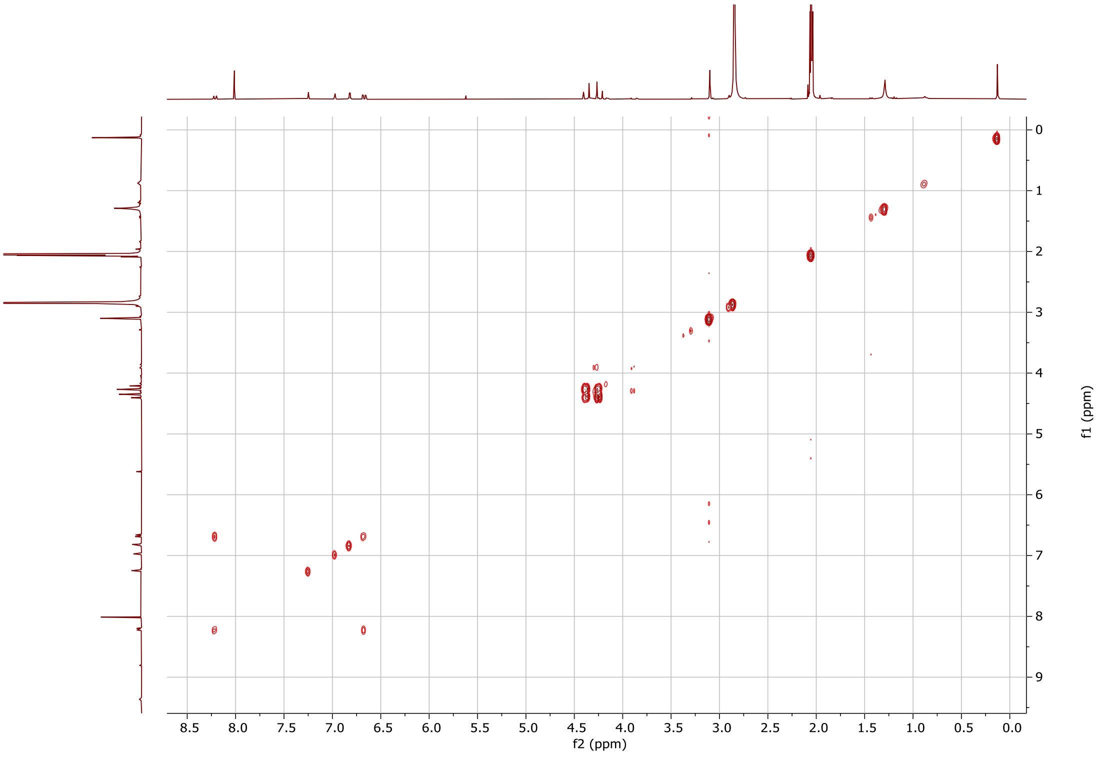

$^{13}C$ NMR of compound **4e** (176 MHz, acetone-$d_6$, 25ºC)

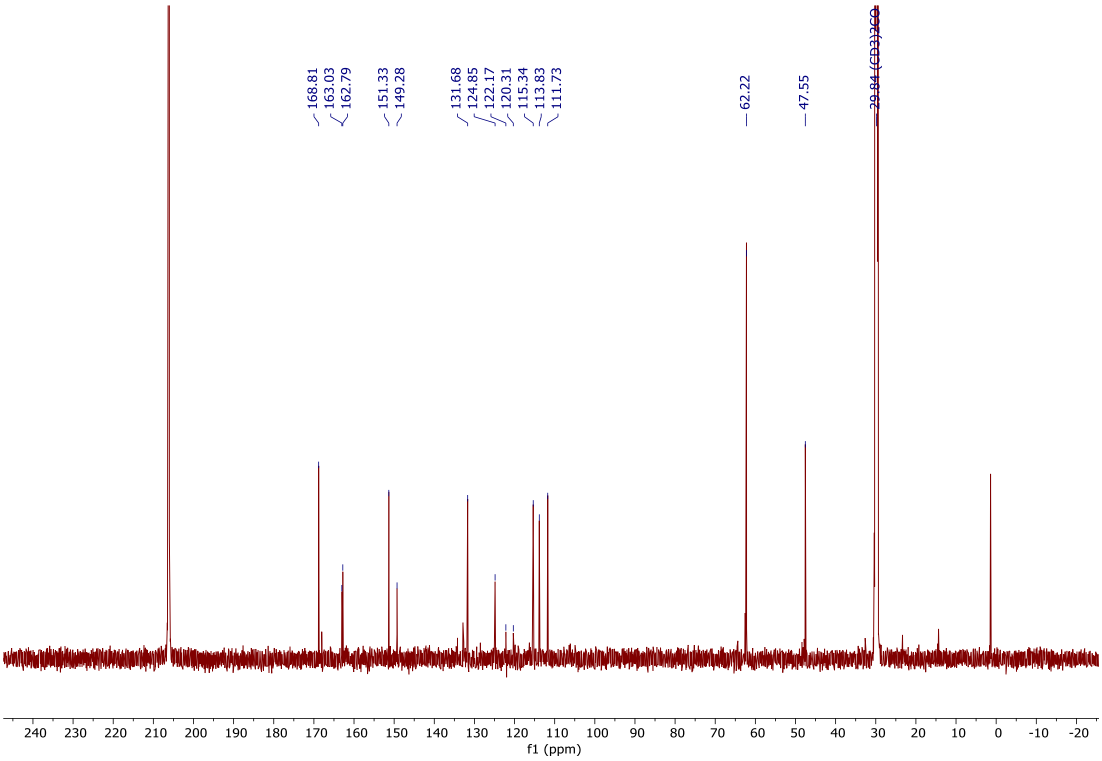


$^{19}F$ NMR of compound **4e** (282 MHz, acetone-$d_6$, 25ºC)

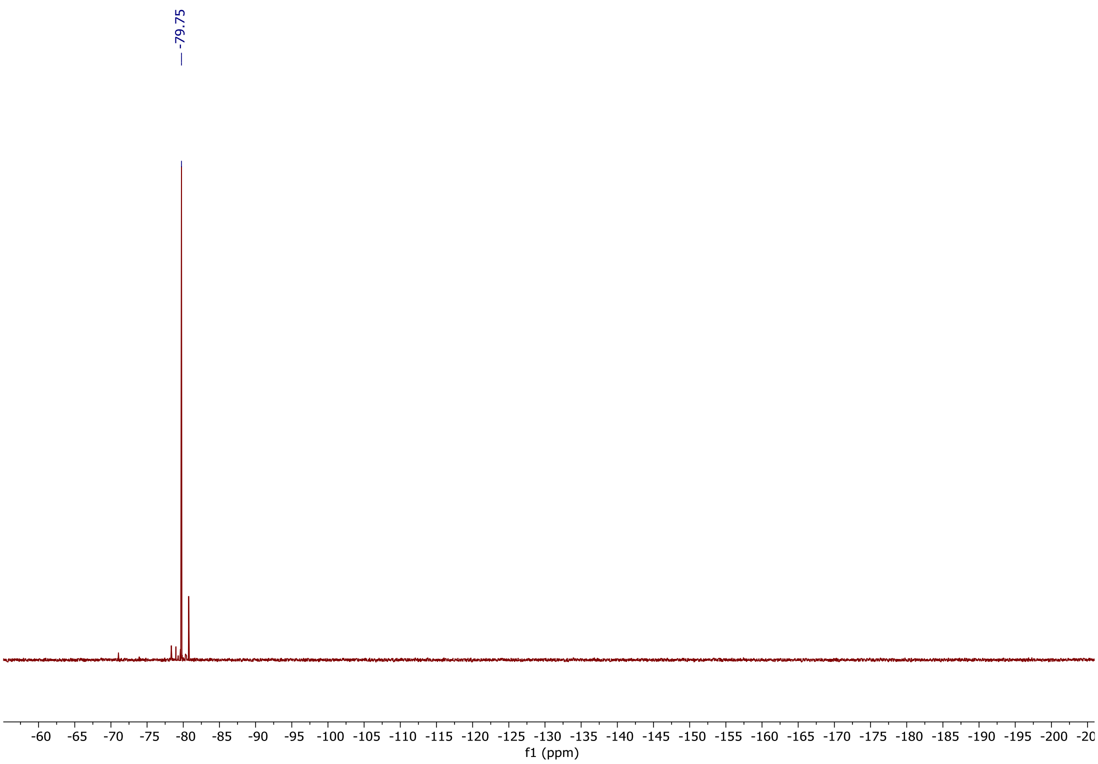

$^{1}$H NMR of compound **4f** (300 MHz, acetone-$d_6$, 25ºC)

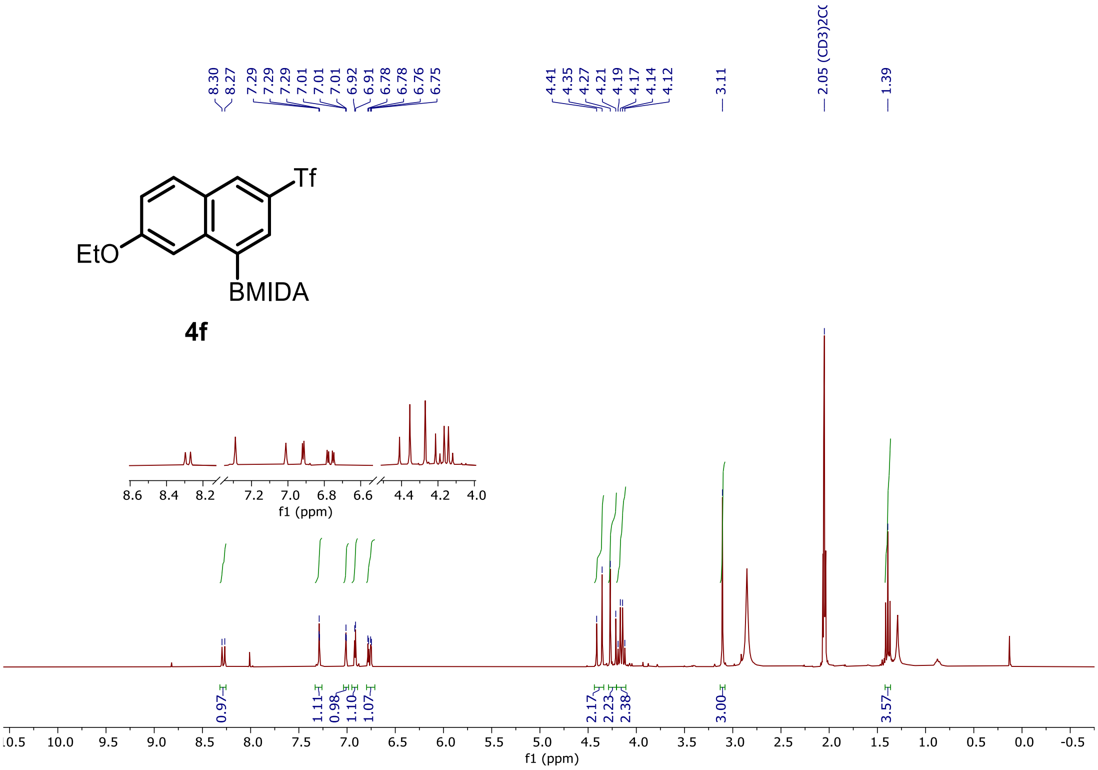


$^{13}$C NMR of compound **4f** (75 MHz, acetone-$d_6$, 25ºC)

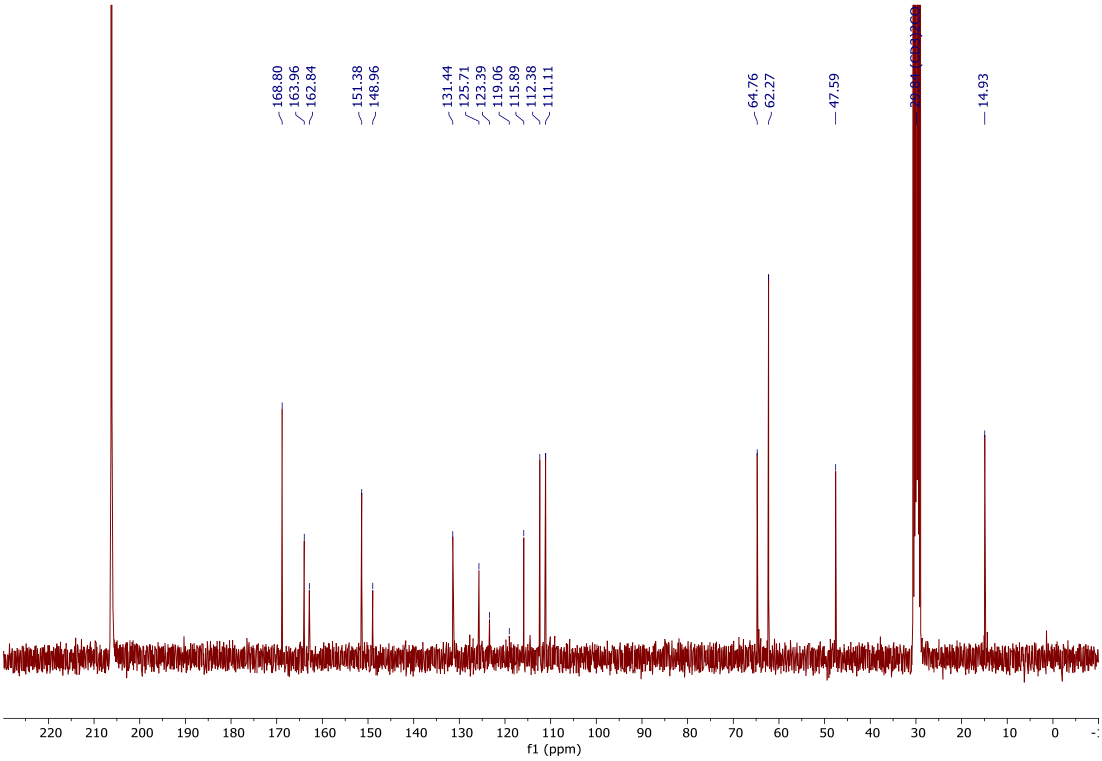

$^{19}F$ NMR of compound **4f** (282 MHz, acetone-$d_6$, 25ºC)

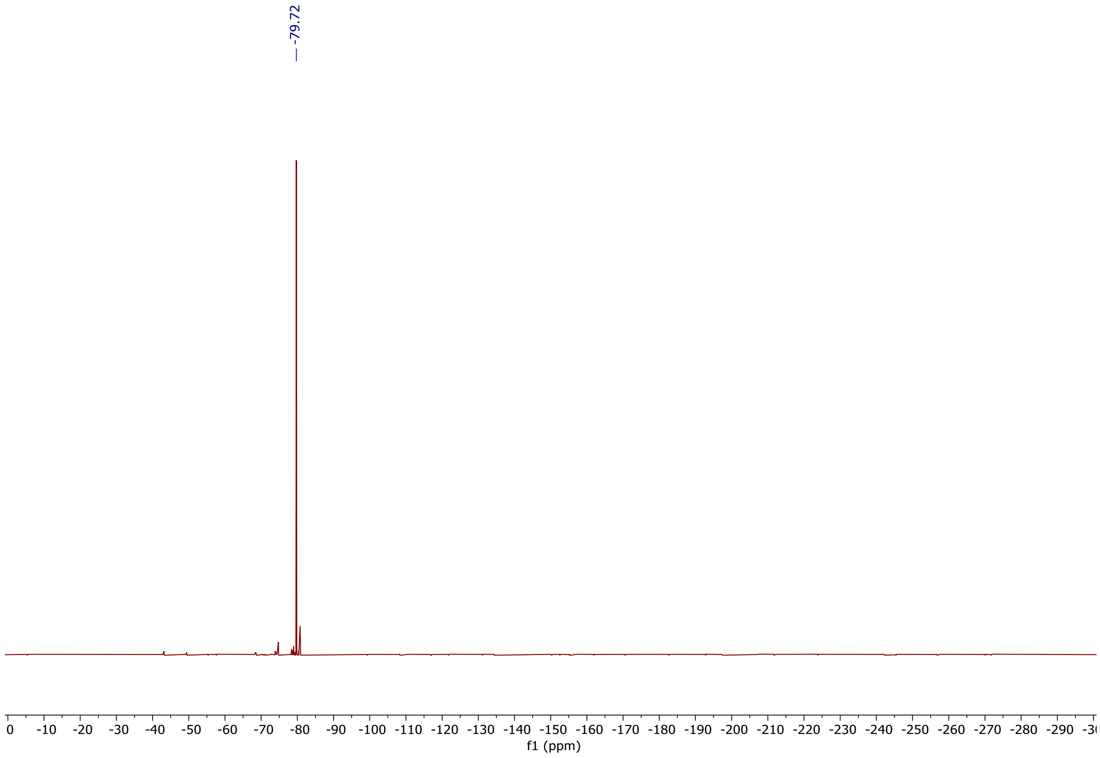

$^1$H NMR of compound **4h** (700 MHz, acetone-d$_6$, 25ºC)

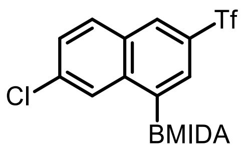


**4h**

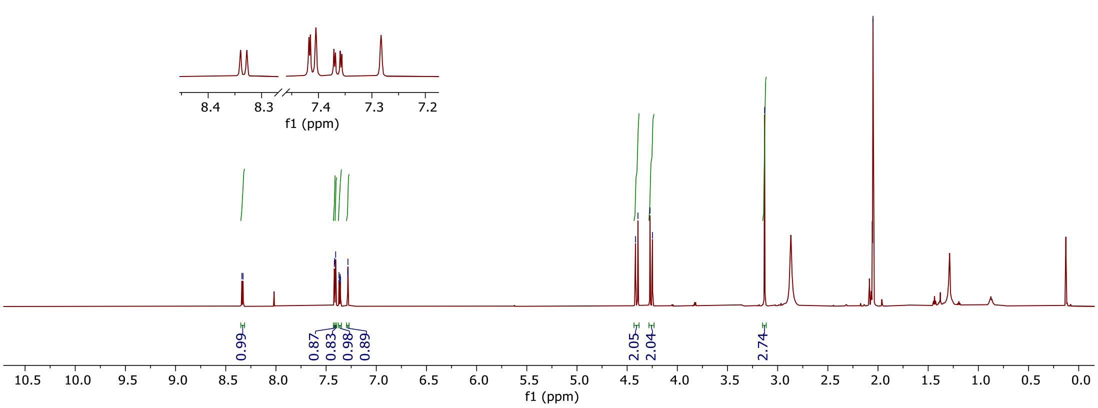


$^{13}$C NMR of compound **4h** (176 MHz, acetone-d$_6$, 25ºC)

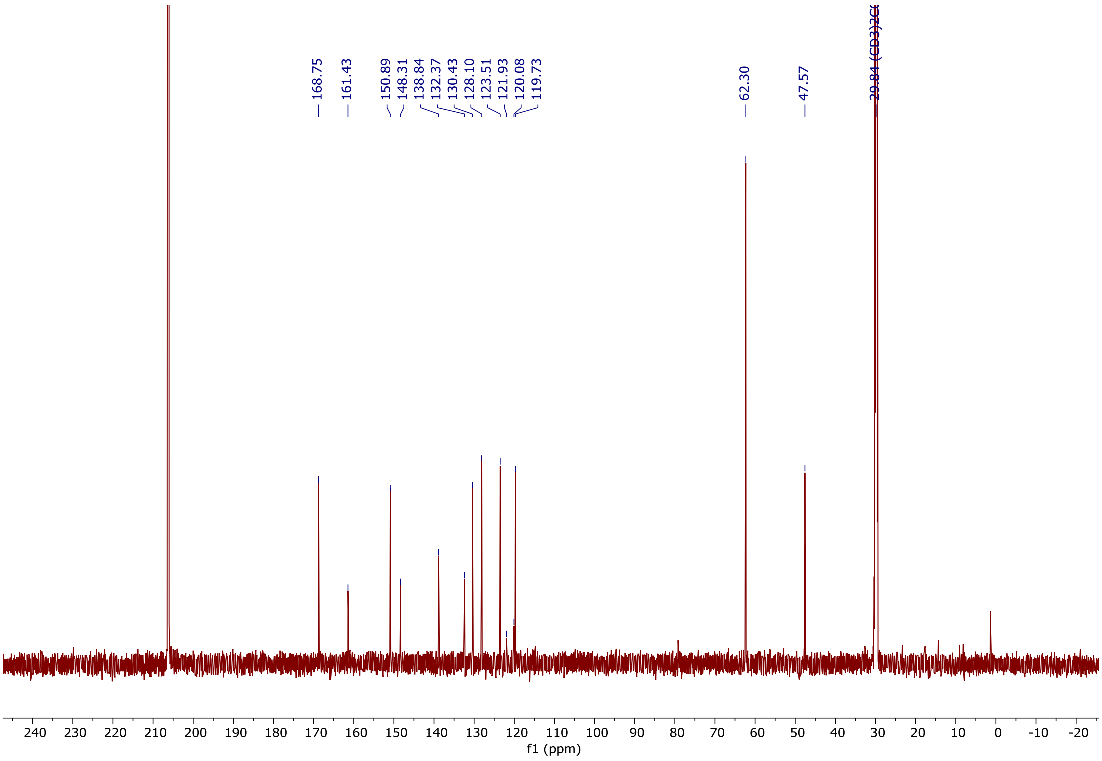

$^{19}$F NMR of compound **4h** (282 MHz, acetone-d$_6$, 25ºC)

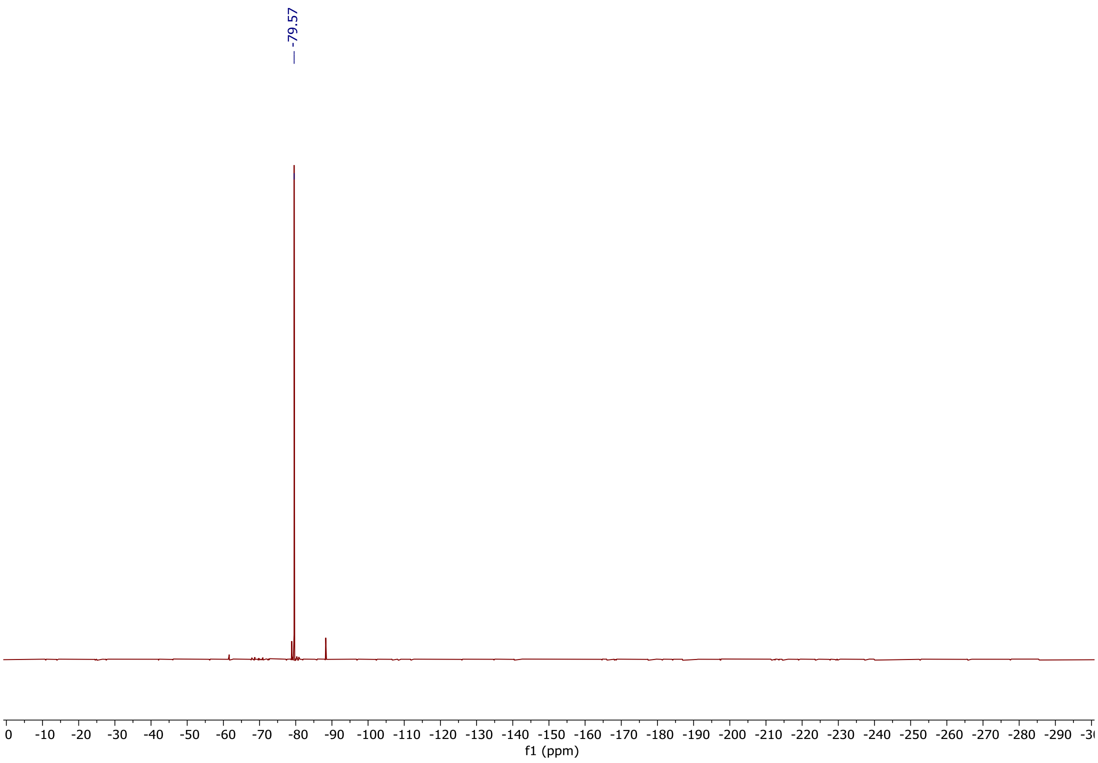


$^1$H-$^{13}$C HMBC NMR of compound **4h** (176 MHz /700 MHz, acetone-d$_6$, 25ºC)

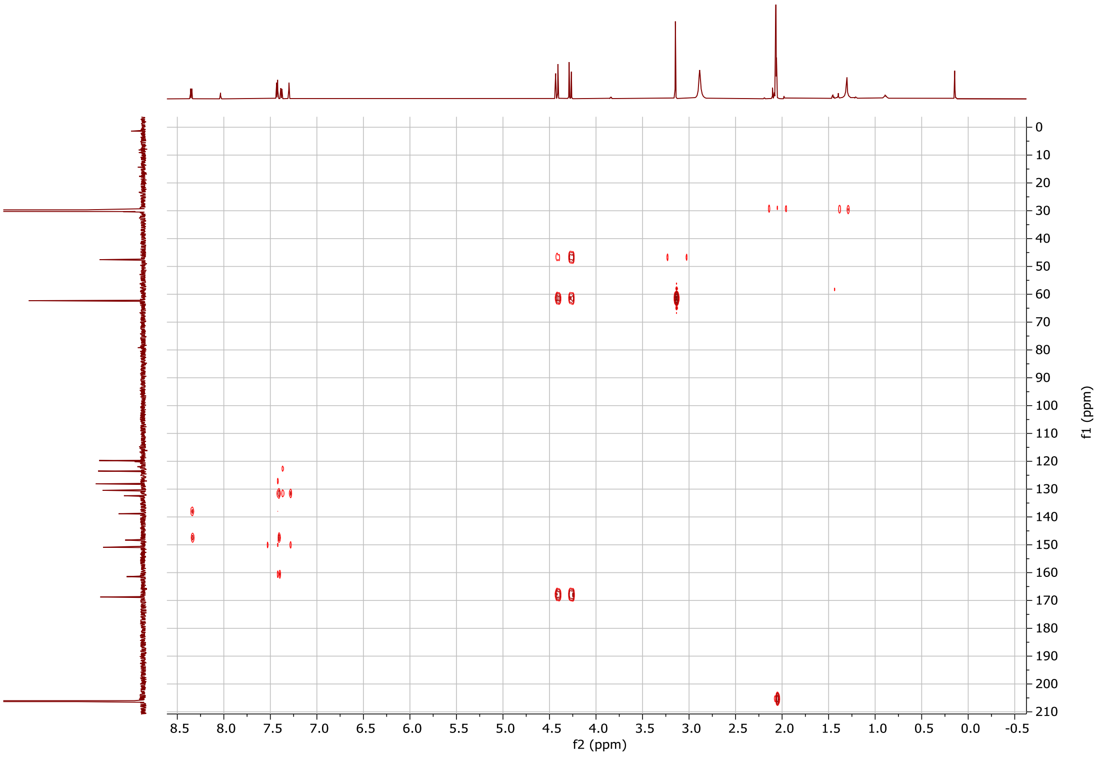

$^{1}H$-$^{13}C$ HSQC NMR of compound **4h** (176 MHz /700 MHz, acetone-$d_6$, 25ºC)

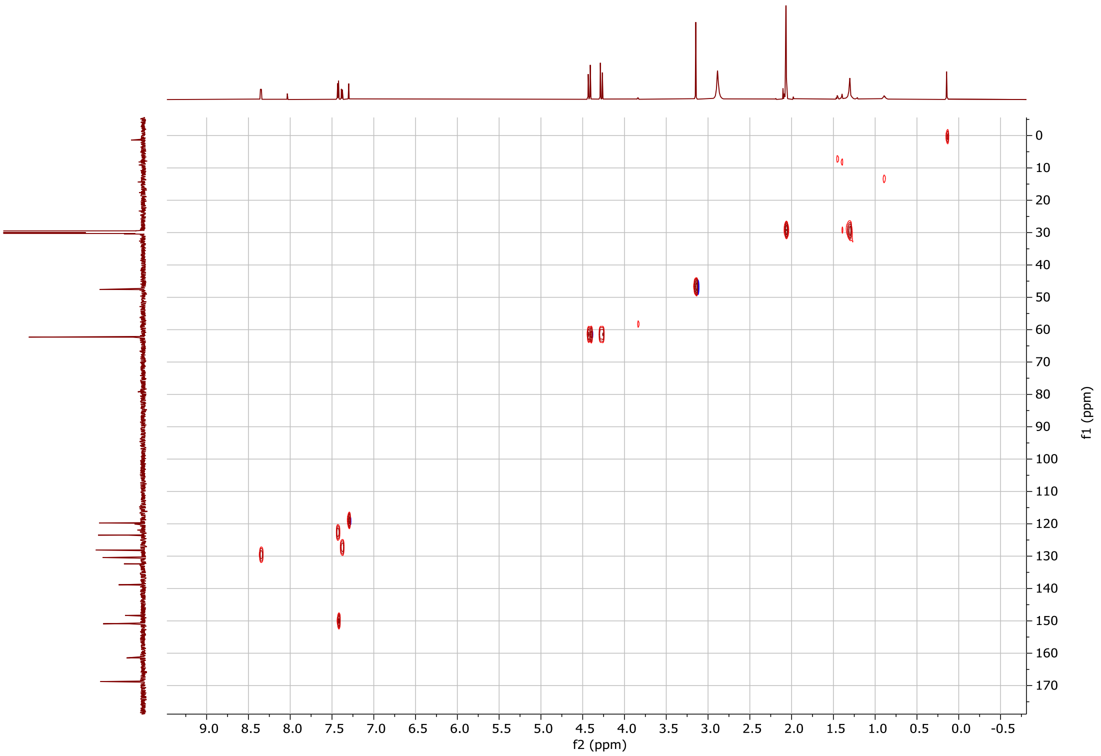

$^{1}$H NMR of compound **4k** (700 MHz, acetone-$d_6$, 25ºC)

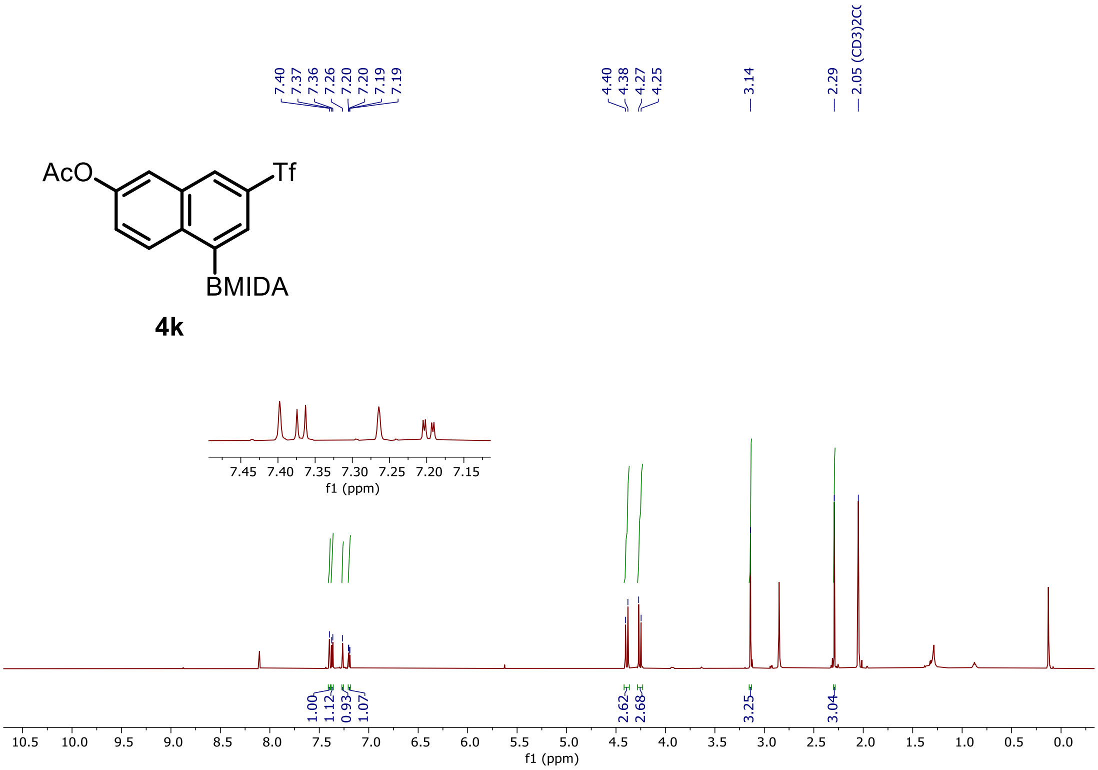


$^{13}$C NMR of compound **4k** (176 MHz, acetone-$d_6$, 25ºC)

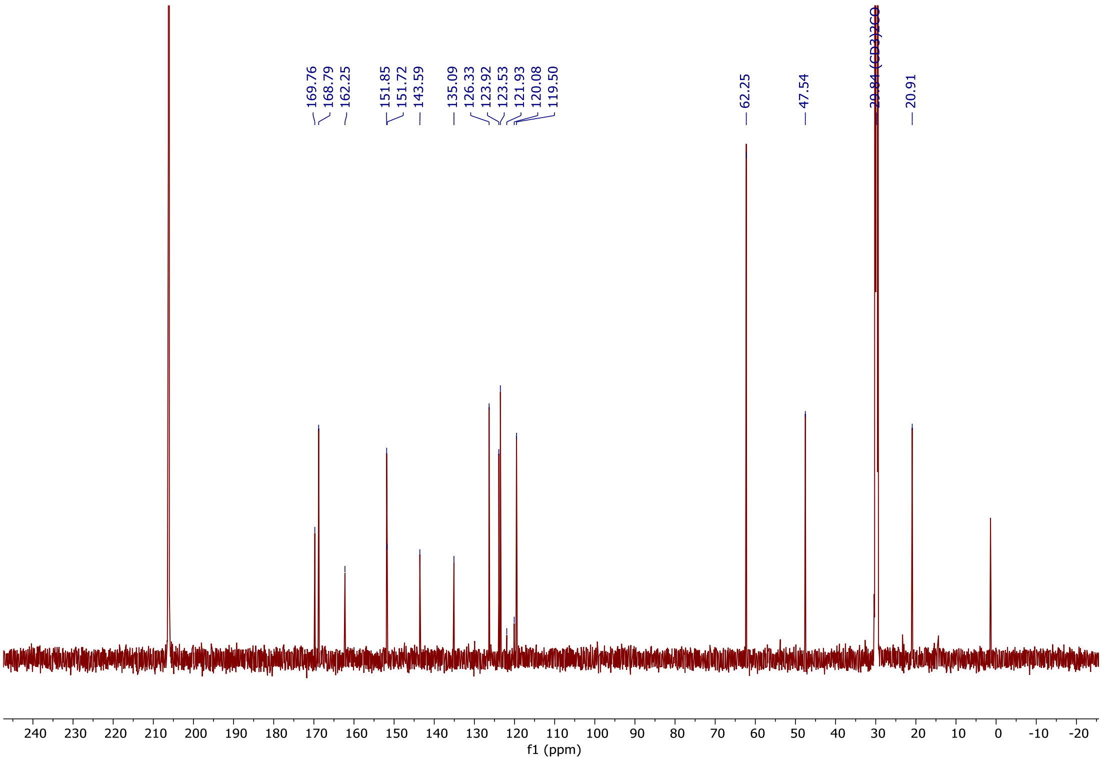

$^{19}F$ NMR of compound **4k** (282 MHz, acetone-$d_6$, 25ºC)

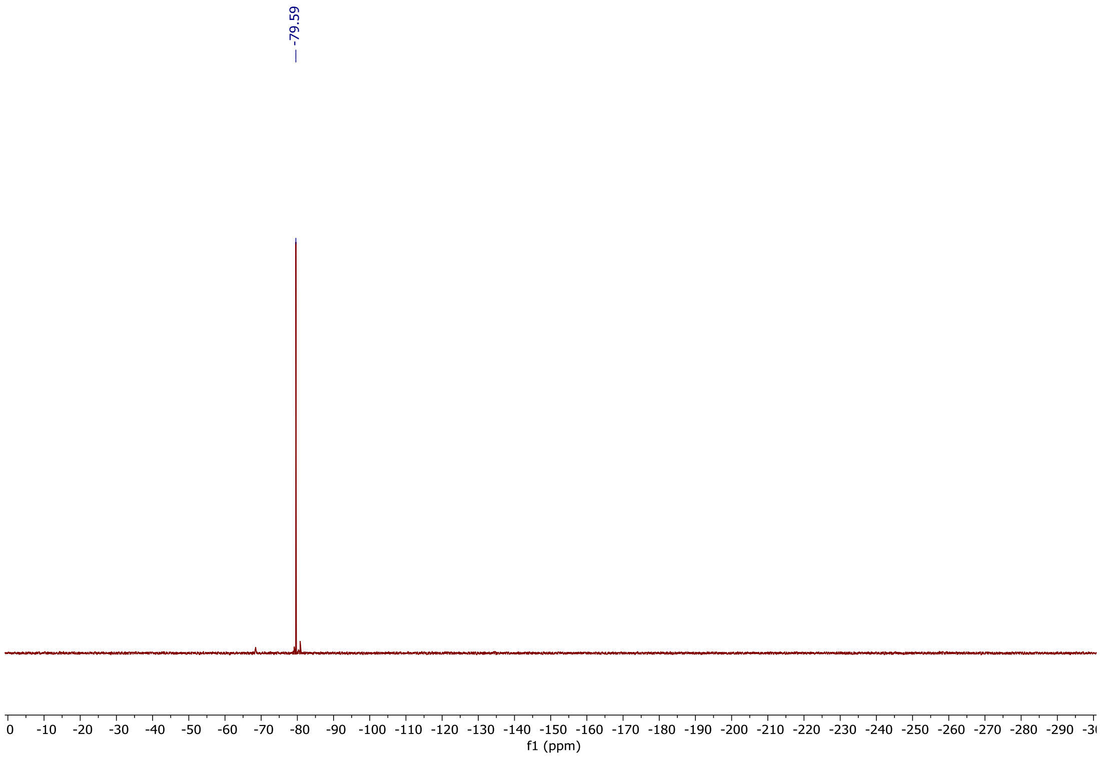

$^{1}$H NMR of compound **4l** (700 MHz, acetone-d$_6$, 25ºC)

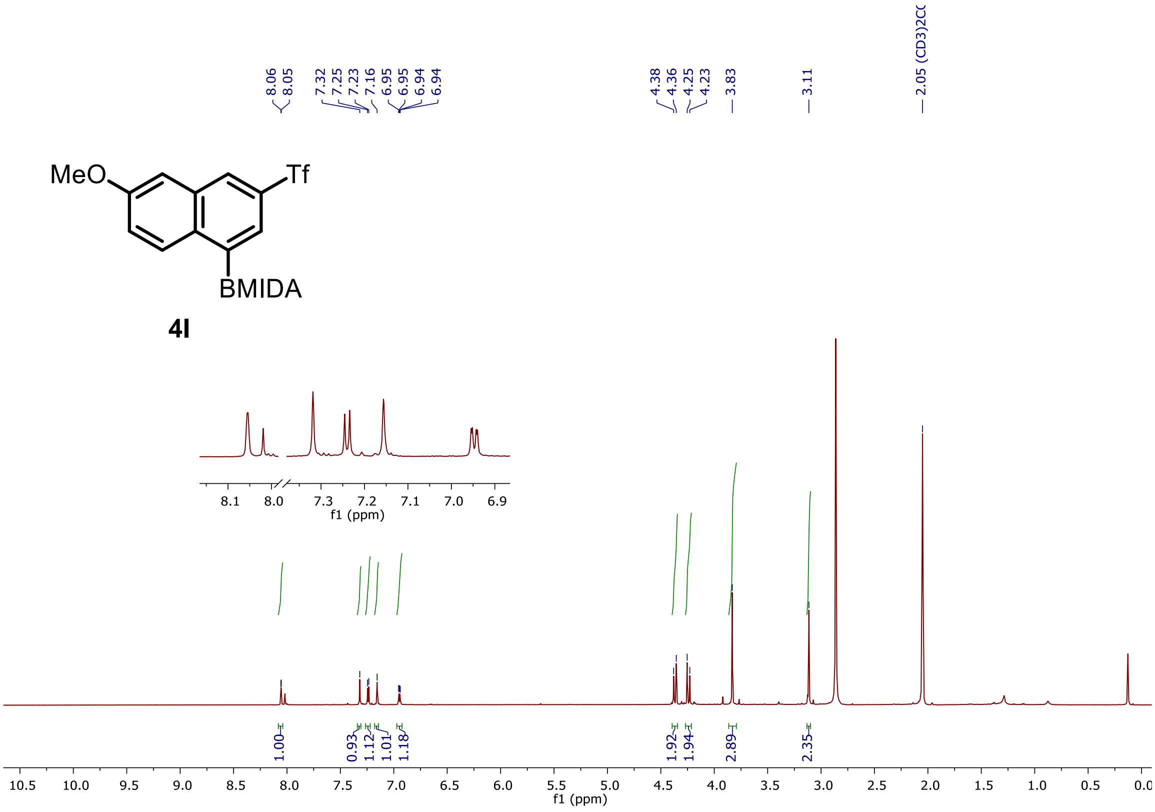


$^{13}$C NMR of compound **4l** (176 MHz, acetone-d$_6$, 25ºC)

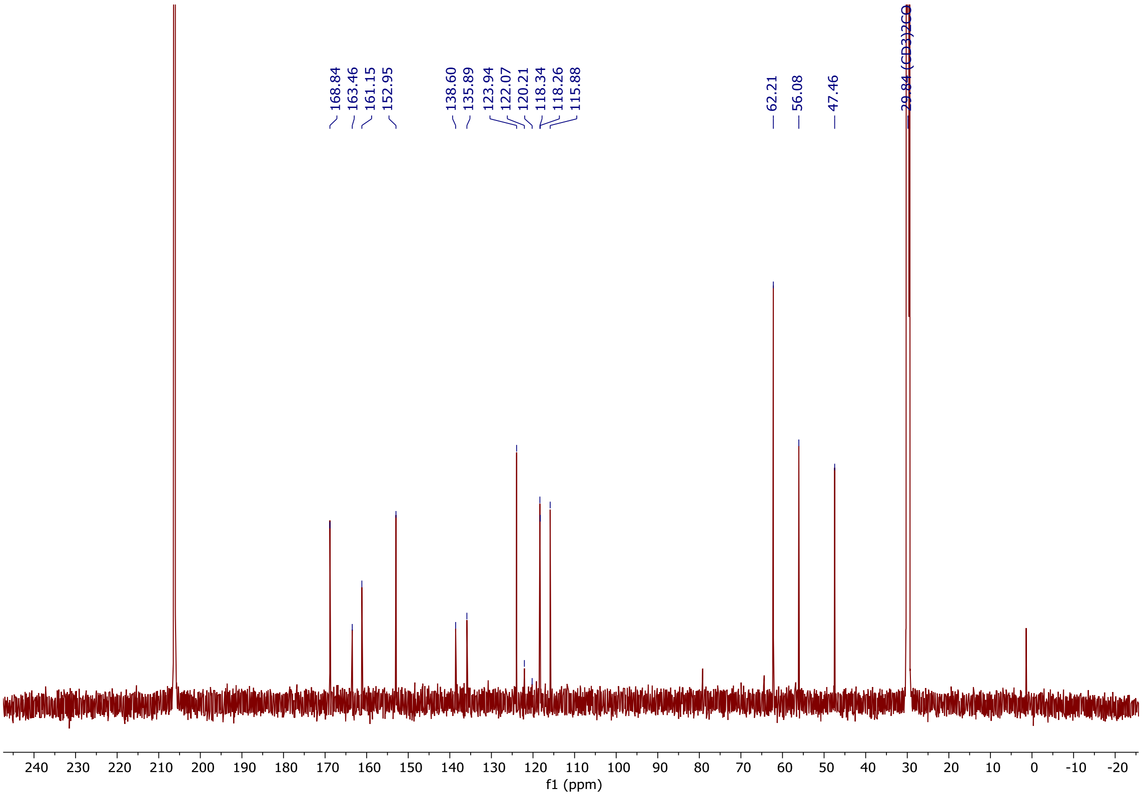

$^{19}F$ NMR of compound **4l** (282 MHz, acetone-d$_6$, 25ºC)

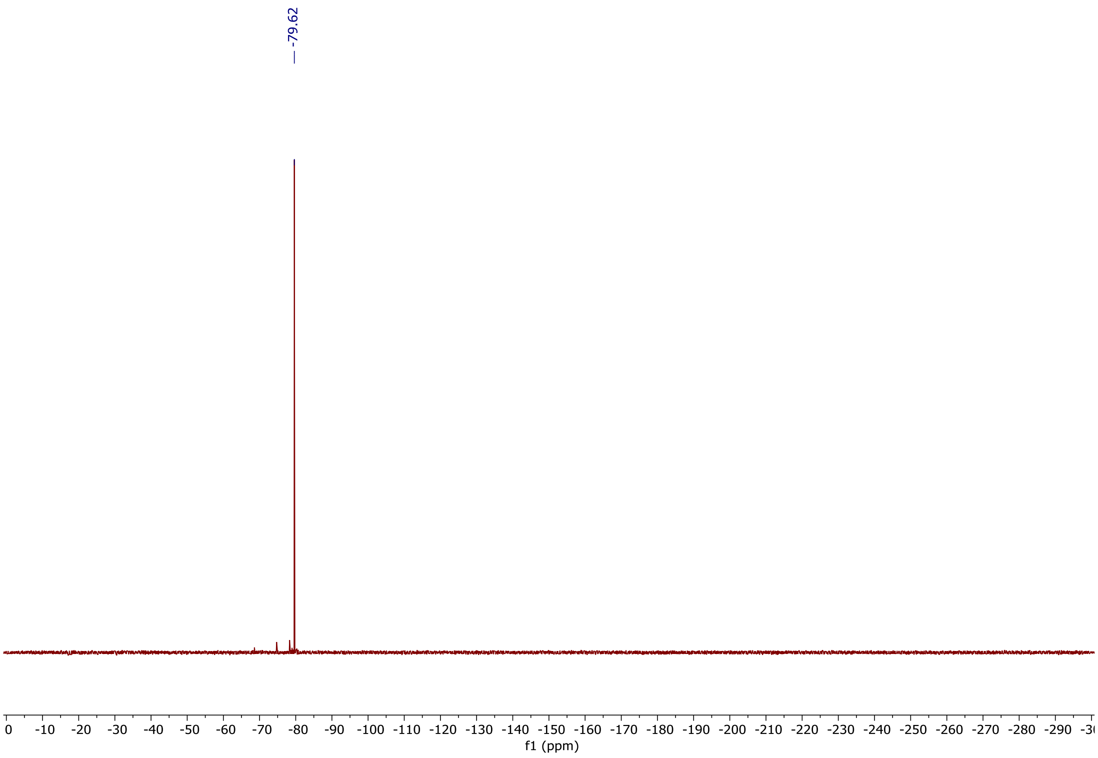


$^{1}H$-$^{13}C$ HMBC NMR of compound **4l** (176 MHz /700 MHz, acetone-d$_6$, 25ºC)

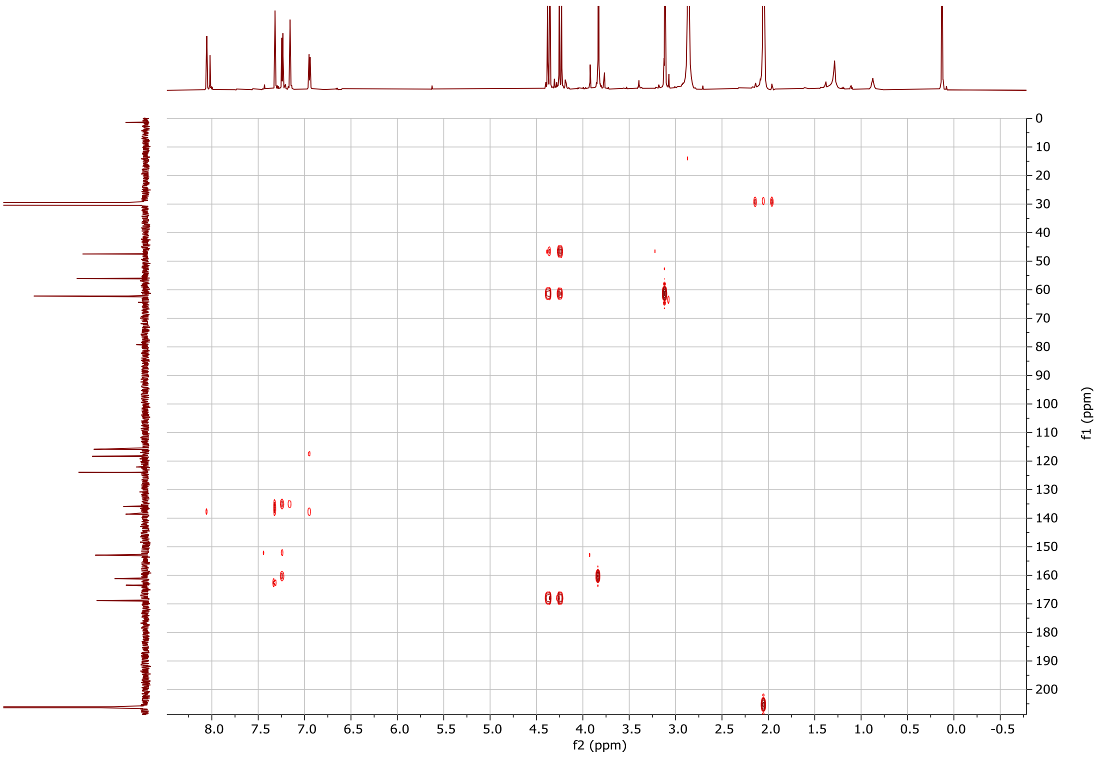

$^{1}H$-$^{13}C$ HSQC NMR of compound **4l** (176 MHz /700 MHz, acetone-$d_6$, 25ºC)

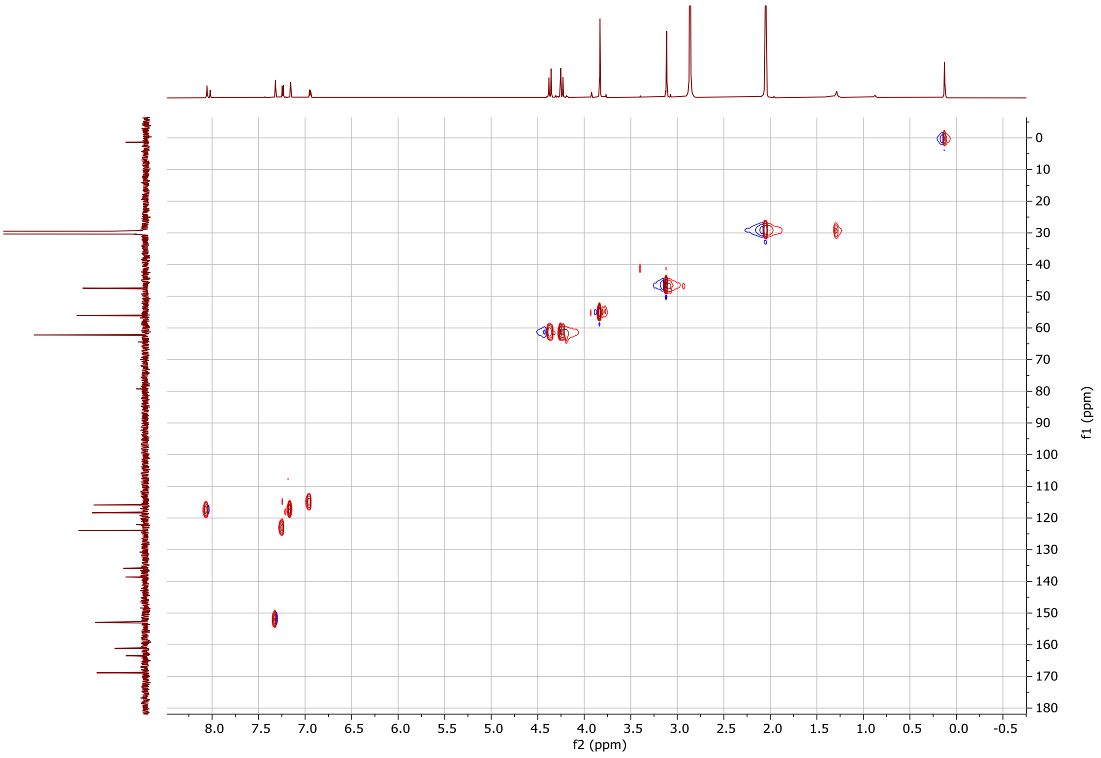


NOESY NMR of compound **4l** (300 MHz, acetone-$d_6$, 25ºC)

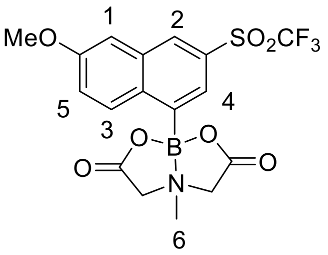

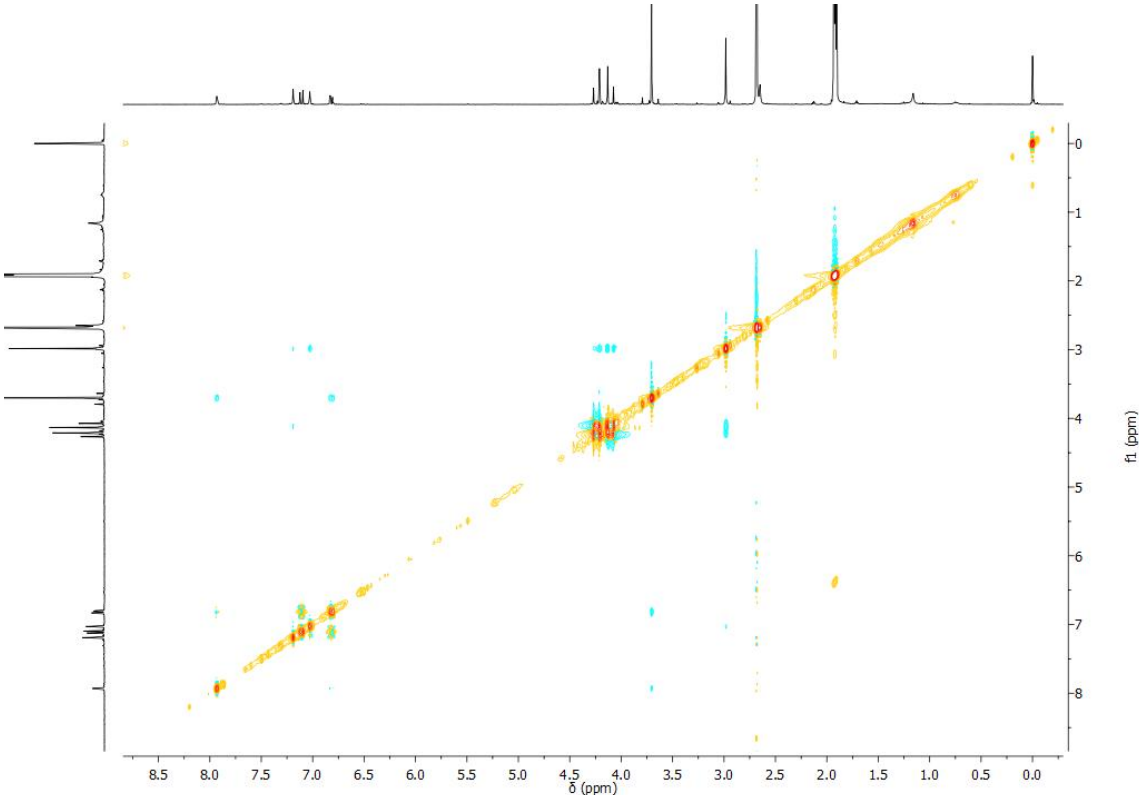
δ (ppm)
f1 (ppm)

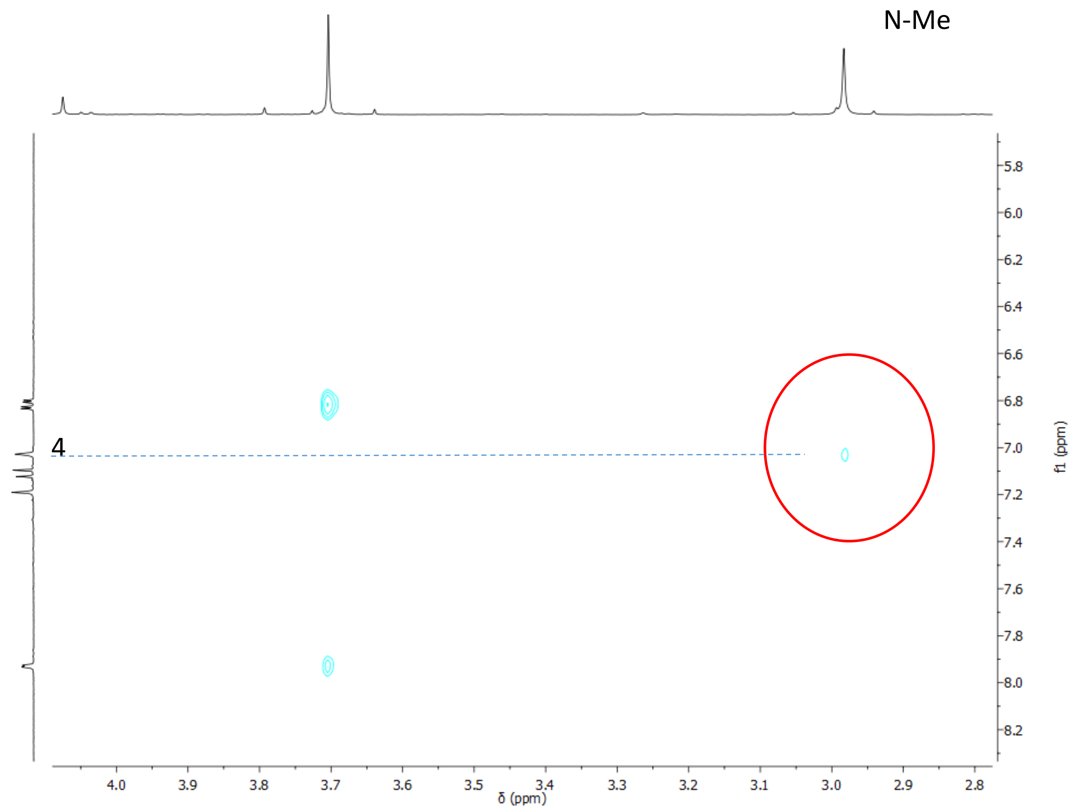
N-Me
4
δ (ppm)
f1 (ppm)

$^1H$ NMR of compound **4s** (300 MHz, acetone-$d_6$, 25ºC)

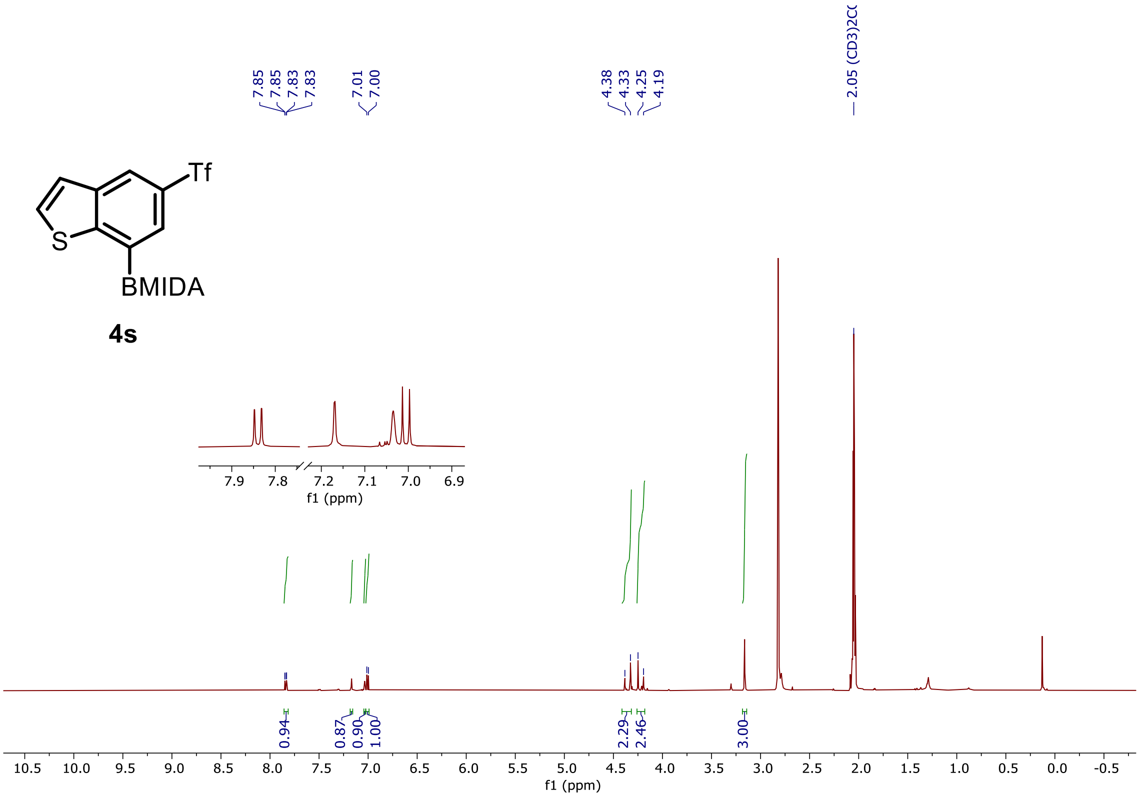


$^{13}C$ NMR of compound **4s** (176 MHz, acetone-$d_6$, 25ºC)

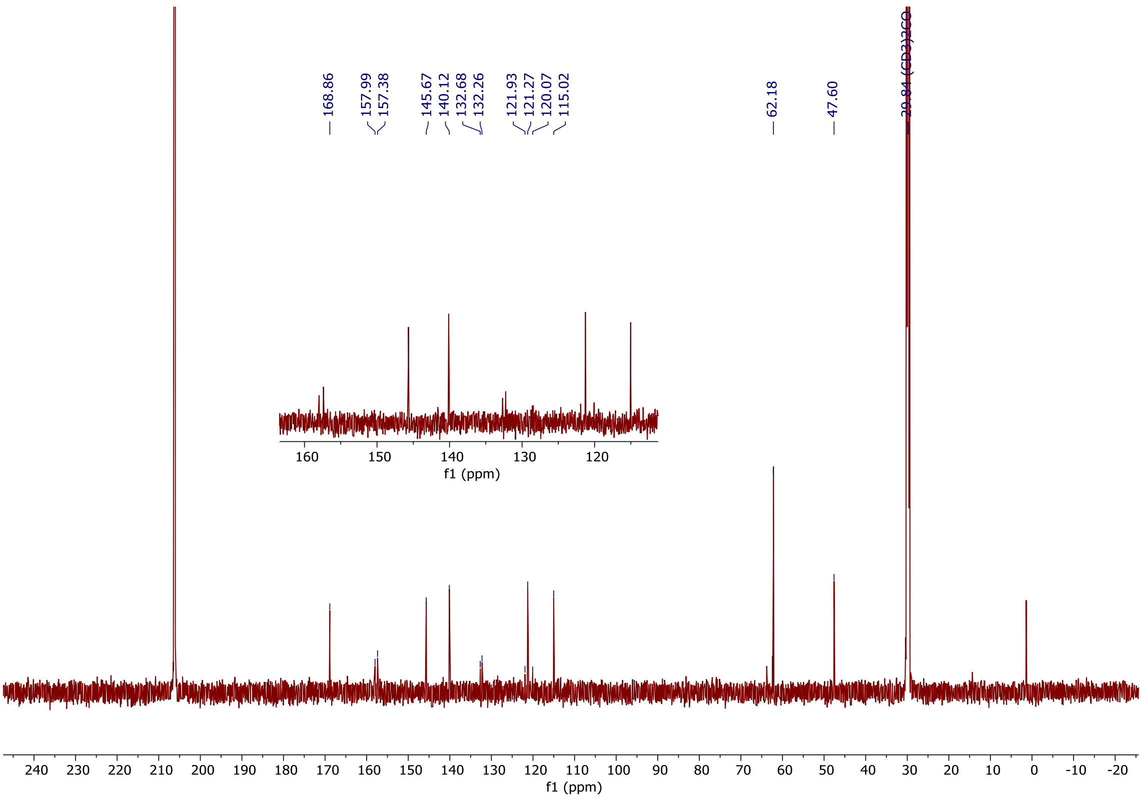

$^{19}F$ NMR of compound **4s** (282 MHz, acetone-$d_6$, 25°C)

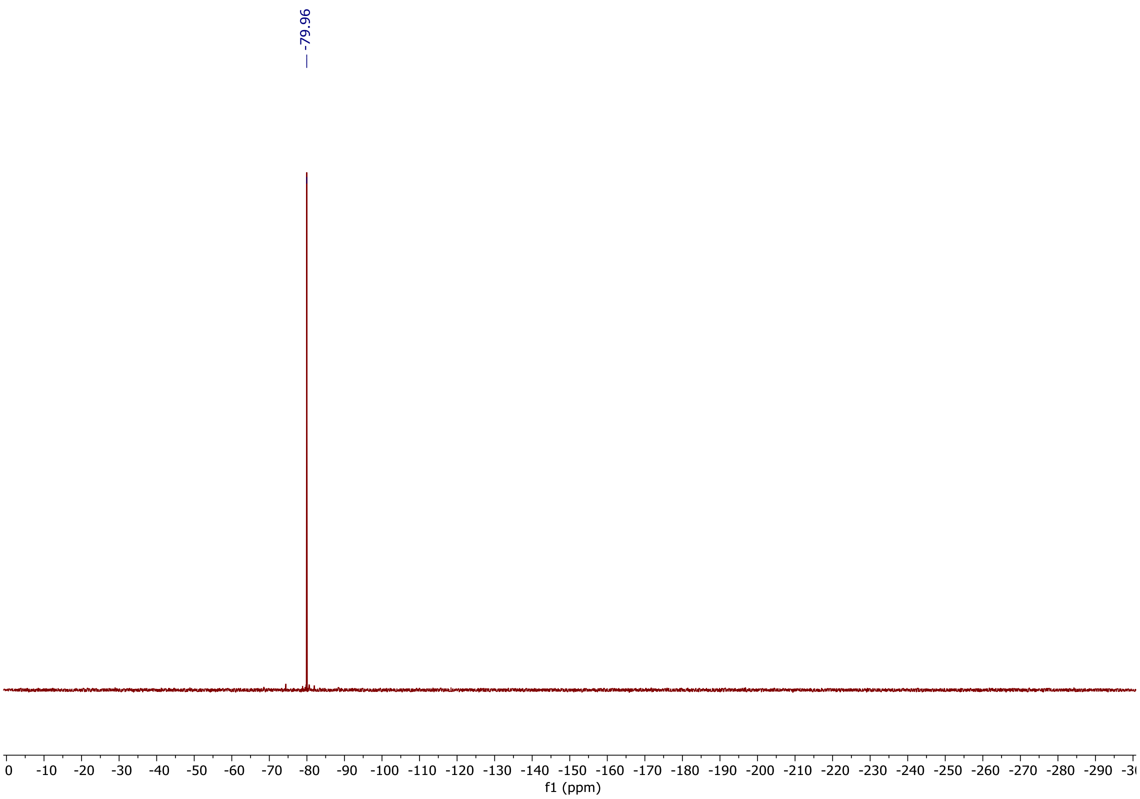


$^{1}H$-$^{13}C$ HMBC- NMR of compound **4s** (176 MHz /700 MHz, acetone-$d_6$, 25°C)

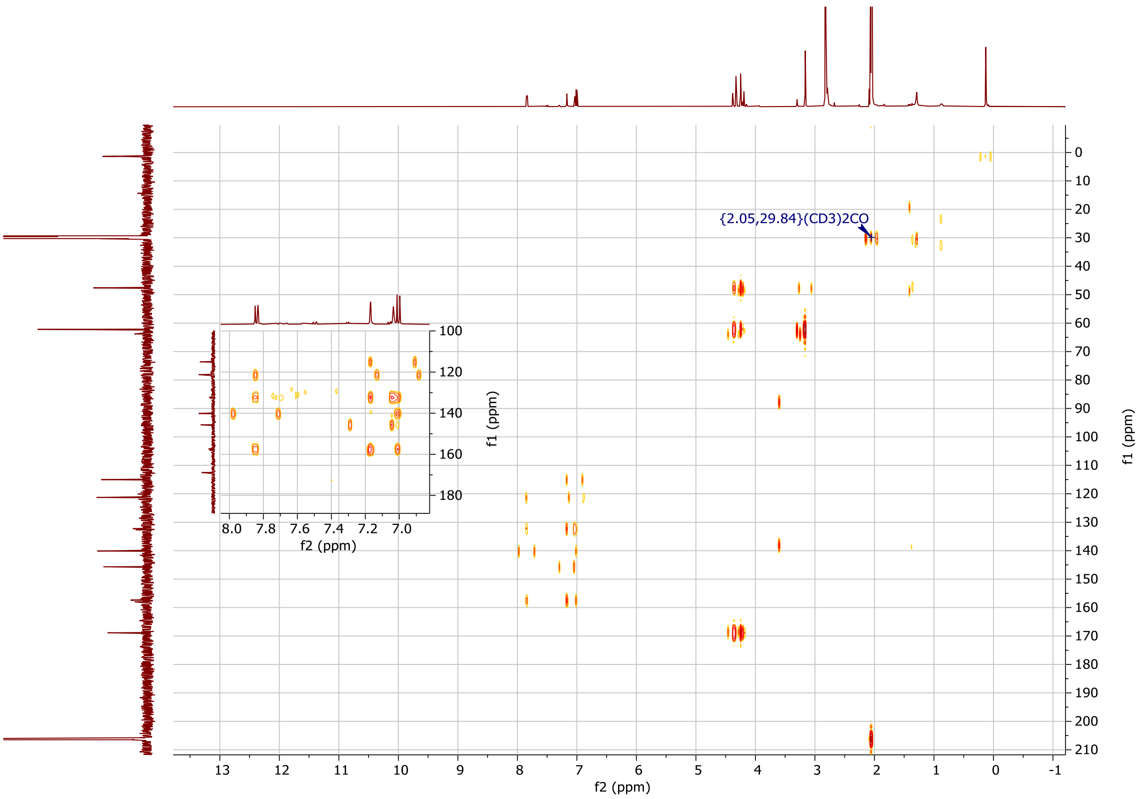

$^{1}H$-$^{13}C$ HSQC NMR of compound **4s** (176 MHz /700 MHz, acetone-$d_6$, 25ºC)

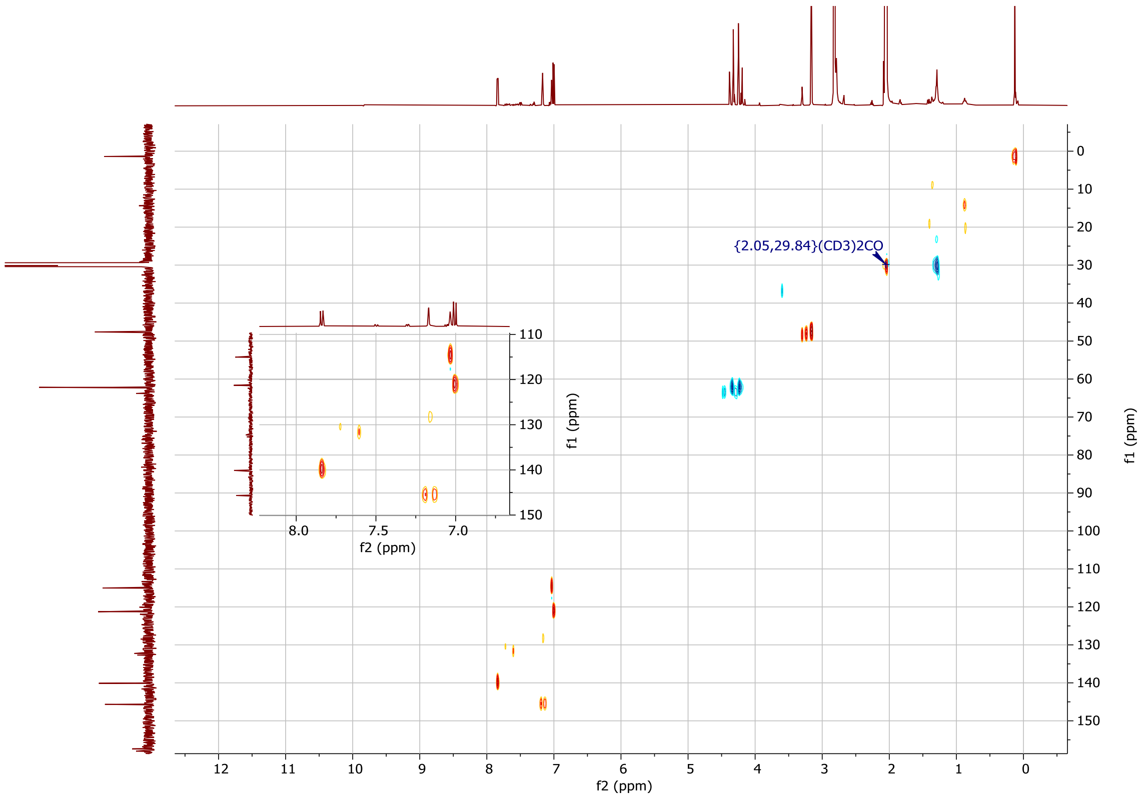

## DFT calculations

All calculations were conducted by using a *Gaussian 09* program package, revision D.01.[i] Molecular geometries were optimized at PCM(toluene)-UM06-2X/6-31+G(d,p) level of theory. Free energies $\Delta G$ are obtained by the following frequency calculations. Single imaginary frequency was obtained for the transition state, which was supported by the intrinsic reaction coordinate (IRC) calculations using the local quadratic approximation (lqa) algorithm. Each geometry of reaction intermediates was obtained by structural optimization of the IRC geometries.

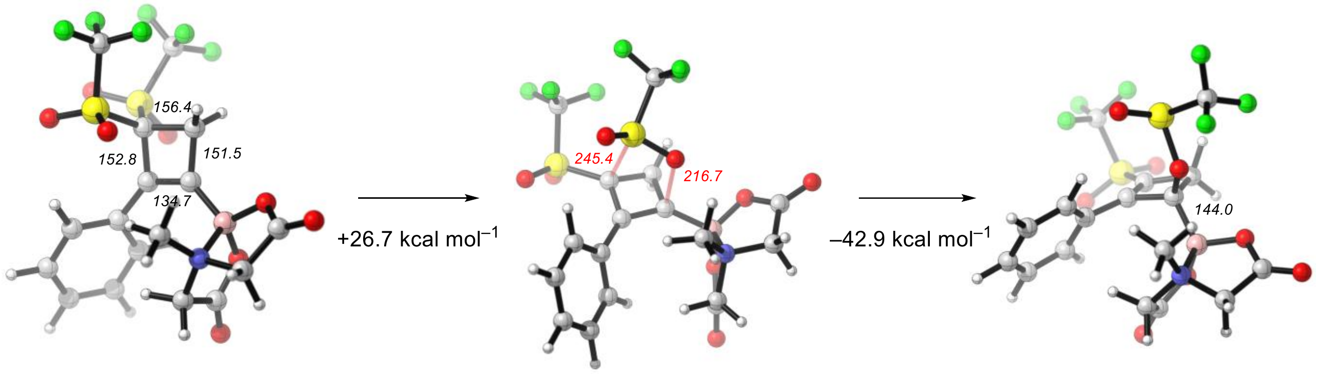


**3-H** 0 kcal mol$^{-1}$ *TS-1* +28.7 kcal mol$^{-1}$ Int **A** –14.2 kcal mol$^{-1}$

**Table S1.** Molecular geometries and energies

| **3-H** | | | | | ***TS-1*** | | | | |
|---|---|---|---|---|---|---|---|---|---|
| Center Number | Atomic Number | Coordinates (Angstroms) X | Y | Z | Center Number | Atomic Number | Coordinates (Angstroms) X | Y | Z |
| 1 | 6 | 0.069019 | 0.278145 | 0.464046 | 1 | 6 | 0.058181 | -0.861523 | -0.625298 |
| 2 | 6 | 0.907703 | -0.748105 | 0.729271 | 2 | 6 | 0.994614 | 0.132896 | -1.012531 |
| 3 | 6 | -0.198939 | -1.779994 | 0.837087 | 3 | 6 | 0.049710 | 0.687629 | -2.083616 |
| 4 | 6 | -1.162686 | -0.605351 | 0.455709 | 4 | 6 | -0.941269 | -0.162001 | -1.294369 |
| 5 | 6 | 0.261579 | 1.718936 | 0.256124 | 5 | 6 | 0.125762 | -2.007122 | 0.291527 |
| 6 | 6 | 1.154823 | 2.400866 | 1.094839 | 6 | 6 | 1.061207 | -3.019291 | 0.041025 |
| 7 | 1 | 1.615971 | 1.874572 | 1.926799 | 7 | 1 | 1.671921 | -2.980102 | -0.859587 |
| 8 | 6 | 1.423177 | 3.751316 | 0.886817 | 8 | 6 | 1.179412 | -4.093516 | 0.920344 |
| 9 | 1 | 2.120433 | 4.266153 | 1.541053 | 9 | 1 | 1.899407 | -4.880605 | 0.716900 |
| 10 | 6 | 0.786302 | 4.436623 | -0.147856 | 10 | 6 | 0.363478 | -4.159269 | 2.050082 |
| 11 | 6 | -0.129243 | 3.770820 | -0.963271 | 11 | 6 | -0.578429 | -3.158674 | 2.292646 |
| 12 | 1 | -0.642500 | 4.305593 | -1.756593 | 12 | 1 | -1.219236 | -3.213583 | 3.167195 |
| 13 | 6 | -0.396311 | 2.419003 | -0.763600 | 13 | 6 | -0.704854 | -2.083855 | 1.416423 |
| 14 | 1 | -1.121256 | 1.917129 | -1.398047 | 14 | 1 | -1.433417 | -1.300304 | 1.607390 |

| | | | | | | | | | |
|---|---|---|---|---|---|---|---|---|---|
| 15 | 5 | 2.484103 | -0.971669 | 0.786696 | 15 | 5 | 2.568577 | 0.409264 | -0.864623 |
| 16 | 8 | 2.788587 | -2.378796 | 0.999445 | 16 | 8 | 2.872171 | 1.785363 | -1.182363 |
| 17 | 6 | 3.834215 | -2.805114 | 0.295625 | 17 | 6 | 3.850682 | 2.295444 | -0.439568 |
| 18 | 8 | 4.317493 | -3.902001 | 0.365298 | 18 | 8 | 4.301161 | 3.402389 | -0.547424 |
| 19 | 6 | 4.344796 | -1.690566 | -0.616213 | 19 | 6 | 4.352823 | 1.250315 | 0.553341 |
| 20 | 1 | 4.572166 | -2.062801 | -1.616524 | 20 | 1 | 4.522782 | 1.677177 | 1.542656 |
| 21 | 1 | 5.255675 | -1.274142 | -0.175713 | 21 | 1 | 5.294076 | 0.844027 | 0.171635 |
| 22 | 7 | 3.270624 | -0.655471 | -0.648343 | 22 | 7 | 3.311414 | 0.175789 | 0.597551 |
| 23 | 6 | 2.433912 | -0.794421 | -1.868814 | 23 | 6 | 2.485681 | 0.308354 | 1.833134 |
| 24 | 1 | 2.011328 | -1.801765 | -1.895283 | 24 | 1 | 2.108850 | 1.326899 | 1.902443 |
| 25 | 1 | 1.618249 | -0.072583 | -1.829106 | 25 | 1 | 1.643116 | -0.382747 | 1.776927 |
| 26 | 1 | 3.053144 | -0.623694 | -2.752874 | 26 | 1 | 3.114760 | 0.067568 | 2.693754 |
| 27 | 6 | 3.801073 | 0.715670 | -0.455972 | 27 | 6 | 3.907724 | -1.180512 | 0.485087 |
| 28 | 1 | 3.068394 | 1.442939 | -0.822494 | 28 | 1 | 3.228272 | -1.910210 | 0.936105 |
| 29 | 1 | 4.752994 | 0.869421 | -0.967766 | 29 | 1 | 4.878808 | -1.244091 | 0.979300 |
| 30 | 6 | 3.922673 | 0.874851 | 1.058347 | 30 | 6 | 4.002546 | -1.460214 | -1.011056 |
| 31 | 8 | 3.229683 | -0.075580 | 1.679150 | 31 | 8 | 3.300232 | -0.560642 | -1.695319 |
| 32 | 8 | 4.530418 | 1.756064 | 1.605554 | 32 | 8 | 4.598497 | -2.384931 | -1.494685 |
| 33 | 1 | -0.164992 | -2.597420 | 0.110533 | 33 | 1 | -0.092363 | 1.769342 | -2.115656 |
| 34 | 1 | -0.365815 | -2.205225 | 1.829310 | 34 | 1 | 0.250724 | 0.285538 | -3.079541 |
| 35 | 16 | -1.873455 | -0.856414 | -1.226575 | 35 | 16 | -1.087148 | 1.582873 | 0.424663 |
| 36 | 8 | -0.744160 | -1.152264 | -2.095544 | 36 | 8 | 0.419164 | 1.686195 | 0.384753 |
| 37 | 8 | -2.816505 | 0.204290 | -1.534548 | 37 | 8 | -1.685356 | 1.176681 | 1.712662 |
| 38 | 6 | -2.876104 | -2.468137 | -1.123947 | 38 | 6 | -1.484233 | 3.404131 | 0.228617 |
| 39 | 9 | -4.158294 | -2.188865 | -1.198602 | 39 | 9 | -2.801480 | 3.569926 | 0.227055 |
| 40 | 9 | -2.608269 | -3.124850 | -0.002177 | 40 | 9 | -0.986940 | 3.834164 | -0.926590 |
| 41 | 9 | -2.516909 | -3.213530 | -2.158663 | 41 | 9 | -0.960269 | 4.101303 | 1.225328 |
| 42 | 16 | -2.538331 | -0.082422 | 1.542523 | 42 | 16 | -2.584403 | -0.726215 | -1.697771 |
| 43 | 8 | -3.684390 | -0.942416 | 1.293910 | 43 | 8 | -3.108665 | -1.524276 | -0.606077 |
| 44 | 8 | -2.623027 | 1.364565 | 1.520868 | 44 | 8 | -2.535274 | -1.204314 | -3.069763 |
| 45 | 6 | -1.931017 | -0.511679 | 3.262817 | 45 | 6 | -3.592863 | 0.846770 | -1.769256 |
| 46 | 9 | -0.671003 | -0.121688 | 3.415412 | 46 | 9 | -2.851373 | 1.818637 | -2.303625 |
| 47 | 9 | -2.021398 | -1.818276 | 3.470790 | 47 | 9 | -3.977041 | 1.203051 | -0.554202 |
| 48 | 9 | -2.704722 | 0.128604 | 4.121371 | 48 | 9 | -4.650795 | 0.628042 | -2.528547 |
| 49 | 1 | 0.990177 | 5.491104 | -0.308355 | 49 | 1 | 0.454439 | -4.996102 | 2.736164 |

| | |
|---|---|
| E(UM062X) = -2732.30733090 | E(UM062X) = -2732.25914674 |
| Zero-point correction = 0.330136 (Hartree/Particle) | Zero-point correction = 0.328050 (Hartree/Particle) |
| Sum of electronic and thermal Energies = -2731.945941 | Sum of electronic and thermal Energies = -2731.899799 |
| Sum of electronic and thermal Enthalpies = -2731.944996 | Sum of electronic and thermal Enthalpies = -2731.898855 |
| Sum of electronic and thermal Free Energies = -2732.040675 | Sum of electronic and thermal Free Energies = -2731.994960 |

Int **A**

| Center | Atomic | Coordinates (Angstroms) | | |
|---|---|---|---|---|
| Number | Number | X | Y | Z |
| 1 | 6 | -0.153263 | -0.854228 | -0.218090 |
| 2 | 6 | 0.455667 | 0.550028 | -0.293296 |
| 3 | 6 | -0.404087 | 0.782694 | -1.587951 |
| 4 | 6 | -0.825598 | -0.644924 | -1.365860 |
| 5 | 6 | 0.151456 | -1.988785 | 0.659713 |
| 6 | 6 | 0.567395 | -3.205996 | 0.099699 |
| 7 | 1 | 0.589902 | -3.323340 | -0.978961 |
| 8 | 6 | 0.979092 | -4.246525 | 0.928614 |
| 9 | 1 | 1.315083 | -5.180186 | 0.488314 |
| 10 | 6 | 0.963624 | -4.088547 | 2.314445 |
| 11 | 6 | 0.532015 | -2.885455 | 2.875704 |
| 12 | 1 | 0.501194 | -2.765285 | 3.954465 |
| 13 | 6 | 0.134057 | -1.833998 | 2.054458 |
| 14 | 1 | -0.221423 | -0.904725 | 2.492329 |
| 15 | 5 | 2.035234 | 0.631246 | -0.640562 |
| 16 | 8 | 2.385197 | 1.978886 | -1.053195 |
| 17 | 6 | 3.620978 | 2.326706 | -0.704120 |
| 18 | 8 | 4.161492 | 3.359766 | -0.989263 |
| 19 | 6 | 4.268910 | 1.202428 | 0.100540 |
| 20 | 1 | 4.839953 | 1.581286 | 0.949631 |
| 21 | 1 | 4.939030 | 0.647803 | -0.563203 |
| 22 | 7 | 3.146584 | 0.319811 | 0.546422 |
| 23 | 6 | 2.758029 | 0.652835 | 1.943615 |
| 24 | 1 | 2.516272 | 1.715325 | 2.000678 |
| 25 | 1 | 1.880396 | 0.069797 | 2.217732 |

| | | | | |
|---|---|---|---|---|
| 26 | 1 | 3.592483 | 0.415111 | 2.608099 |
| 27 | 6 | 3.464706 | -1.119541 | 0.361105 |
| 28 | 1 | 2.820031 | -1.724333 | 1.007638 |
| 29 | 1 | 4.509665 | -1.339689 | 0.587784 |
| 30 | 6 | 3.120839 | -1.406500 | -1.100764 |
| 31 | 8 | 2.408544 | -0.403821 | -1.613164 |
| 32 | 8 | 3.440439 | -2.401030 | -1.693572 |
| 33 | 1 | -1.191746 | 1.533833 | -1.480724 |
| 34 | 1 | 0.164612 | 0.972669 | -2.502272 |
| 35 | 16 | -1.362224 | 1.641926 | 1.297515 |
| 36 | 8 | 0.206056 | 1.446506 | 0.805016 |
| 37 | 8 | -1.549286 | 0.939077 | 2.582577 |
| 38 | 6 | -0.949248 | 3.396168 | 1.802831 |
| 39 | 9 | -2.005159 | 3.901618 | 2.432757 |
| 40 | 9 | -0.691998 | 4.123309 | 0.721879 |
| 41 | 9 | 0.095925 | 3.421727 | 2.618563 |
| 42 | 16 | -1.867467 | -1.676870 | -2.339707 |
| 43 | 8 | -1.508105 | -3.080501 | -2.213589 |
| 44 | 8 | -2.087688 | -1.039563 | -3.627593 |
| 45 | 6 | -3.462230 | -1.491332 | -1.394371 |
| 46 | 9 | -3.801090 | -0.205102 | -1.343811 |
| 47 | 9 | -3.305428 | -1.946031 | -0.156026 |
| 48 | 9 | -4.415346 | -2.178854 | -2.004656 |
| 49 | 1 | 1.282135 | -4.903996 | 2.957084 |

E(UM062X) = -2732.32594828

Zero-point correction = 0.328887 (Hartree/Particle)

Sum of electronic and thermal Energies = -2731.965248

Sum of electronic and thermal Enthalpies = -2731.964304

Sum of electronic and thermal Free Energies = -2732.063286

## References

[i]. For *Gaussian 09*, Revision D.01, M. J. Frisch, G. W. Trucks, H. B. Schlegel, G. E. Scuseria, M. A. Robb, J. R. Cheeseman, G. Scalmani, V. Barone, B. Mennucci, G. A. Petersson, H. Nakatsuji, M. Caricato, X. Li, H. P. Hratchian, A. F. Izmaylov, J. Bloino, G. Zheng, J. L. Sonnenberg, M. Hada, M. Ehara, K. Toyota, R. Fukuda, J. Hasegawa, M. Ishida, T. Nakajima, Y. Honda, O. Kitao, H. Nakai, T. Vreven, J. A. Montgomery, Jr., J. E. Peralta, F. Ogliaro, M. Bearpark, J. J. Heyd, E. Brothers, K. N. Kudin, V. N. Staroverov, T. Keith, R. Kobayashi, J. Normand, K. Raghavachari, A. Rendell, J. C. Burant, S. S. Iyengar, J. Tomasi, M. Cossi, N. Rega, J. M. Millam, M. Klene, J. E. Knox, J. B. Cross, V. Bakken, C. Adamo, J. Jaramillo, R. Gomperts, R. E. Stratmann, O. Yazyev, A. J. Austin, R. Cammi, C. Pomelli, J. W. Ochterski, R. L. Martin, K. Morokuma, V. G. Zakrzewski, G. A. Voth, P. Salvador, J. J. Dannenberg, S. Dapprich, A. D. Daniels, O. Farkas, J. B. Foresman, J. V. Ortiz, J. Cioslowski, D. J. Fox, Gaussian, Inc., Wallingford CT, 2013.
